# General Synthesis of Secondary Alkylamines by Reductive Alkylation of Nitriles by Aldehydes and Ketones

**DOI:** 10.1002/chem.202004755

**Published:** 2020-12-21

**Authors:** Timon Schönauer, Sabrina L. J. Thomä, Leah Kaiser, Mirijam Zobel, Rhett Kempe

**Affiliations:** ^1^ Inorganic Chemistry II—Catalyst Design University of Bayreuth 95440 Bayreuth Germany; ^2^ Mesostructured Materials Department of Chemistry University of Bayreuth 95440 Bayreuth Germany

**Keywords:** aldehydes, amines, catalysis, cobalt, hydrogenation, nitriles

## Abstract

The development of C−N bond formation reactions is highly desirable due to their importance in biology and chemistry. Recent progress in 3d metal catalysis is indicative of unique selectivity patterns that may permit solving challenges of chemical synthesis. We report here on a catalytic C−N bond formation reaction—the reductive alkylation of nitriles. Aldehydes or ketones and nitriles, all abundantly available and low‐cost starting materials, undergo a reductive coupling to form secondary alkylamines and inexpensive hydrogen is used as the reducing agent. The reaction has a very broad scope and many functional groups, including hydrogenation‐sensitive examples, are tolerated. We developed a novel cobalt catalyst, which is nanostructured, reusable, and easy to handle. The key seems the earth‐abundant metal in combination with a porous support material, N‐doped SiC, synthesized from acrylonitrile and a commercially available polycarbosilane.

## Graphical abstract

**A cobalt catalyst** was developed, which is nanostructured, reusable, and easy to handle. It mediates the selective reductive alkylation of nitriles with carbonyl compounds. The key to a broad scope and the tolerance of numerous functional groups, including hydrogenation sensitive examples, is the earth‐abundant metal in combination with a porous N‐doped SiC catalyst support material.

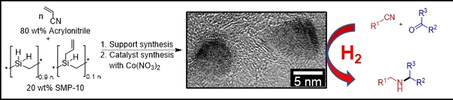

C−N bond formation reactions are of fundamental interest in chemistry and biology, and amines are very important compounds and key functional groups in many bulk and fine chemicals,^[1]^ drugs^[2]^ and materials.^[3]^ Differently substituted secondary alkylamines, examples of pharmaceuticals are shown in Scheme 1 A, are challenging to synthesize. Most of the existing catalytic methods, such as borrowing hydrogen or hydrogen autotransfer,[^4^, ^5^] reductive amination,[^6^, ^7^] hydroaminomethylation^[8]^ and hydroamination,[^9^, ^10^] albeit intensively investigated, start already from an amine (Scheme 1 B) and are restricted regarding the synthesis of secondary alkylamines.^[11]^ The hydrogenation of amides is an alternative (Scheme 1 C), since it does not require an amine as starting material,[^12^, ^13^] but again the synthesis of differently substituted secondary alkylamines is rarely reported.^[14]^ The use of catalysts based on earth‐abundant metals such as Mn, Fe or Co in reactions classically associated with rare noble metals is also of fundamental interest.[^15^, ^16^, ^17^, ^18^, ^19^, ^20^, ^21^, ^22^, ^23^]

**Scheme 1:**
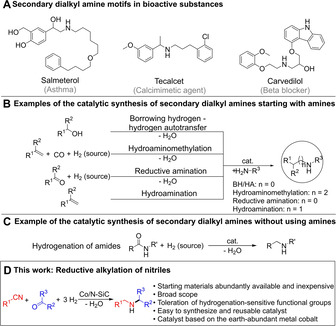
A) Examples of secondary dialkylamine motifs in pharmaceuticals. B/C) Examples of the catalytic synthesis of secondary alkylamines. These methods can be used to synthesize secondary alkylamines but the scopes are mostly demonstrated for aryl‐alkylamines or tertiary alkylamines (borrowing hydrogen/hydrogen autotransfer, reductive alkylation and hydroaminomethylation, hydrogenation of amides). The intermolecular hydroamination of alkylamines and olefins is challenging. D) Catalytic reaction introduced herein.

We report herein that the reductive alkylation of nitriles by aldehydes or ketones permits the general synthesis of secondary alkylamines. The reaction has a broad scope. Aromatic or aliphatic nitriles and benzylic or aliphatic aldehydes or ketones ‐dialkyl, diaryl or arylalkyl substituted—can be employed. In addition, we demonstrated the synthesis and modification of bioactive compounds and pharmaceuticals. Many functional groups, including hydrogenation‐sensitive examples, are tolerated. We had to develop a novel catalyst which is nanostructured, reusable, and easy to handle. The key is the earth‐abundant metal cobalt (Co) in combination with a porous support material, N‐doped SiC synthesized from acrylonitrile and a commercially available polycarbosilane. The catalyst has a distinct selectivity pattern; it permits the selective hydrogenation of aliphatic or aromatic nitriles while barely reducing aldehydes or ketones. In addition, the transiently formed imine intermediate is efficiently hydrogenated.

Attempts to reductively link nitriles and carbonyl compounds in the gas phase^[24]^ indicate that this high‐temperature approach is extremely limited in conversion, scope, and functional group tolerance. Based on the inspiring development of Co catalysts for the selective hydrogenation of nitriles,[^25^, ^26^, ^27^, ^28^, ^29^, ^30^] we expected that a Co catalyst could also be the key to develop a broadly applicable catalytic process for the reductive alkylation of nitriles. Co catalysts have also been employed successfully in reductive amination reactions, the coupling of amines or ammonia and aldehydes or ketones in the presence of hydrogen as the reducing agent.[^27^, ^31^, ^32^, ^33^, ^34^, ^35^, ^36^, ^37^, ^38^, ^39^, ^40^, ^41^] In addition, the Co catalyzed transfer hydrogenation and coupling of nitriles has been described.^[42]^

Our Co catalyst (Co/*N*‐SiC) was synthesized as shown in Figure 1 A. Firstly, we synthesized the N‐doped SiC support (*N*‐SiC), which contains 8 atom% (at %) nitrogen, as determined by elemental analysis, using a modified literature procedure.[^43^, ^44^] Secondly, the *N*‐SiC material was wet impregnated with a solution of Co(NO_3_)_2_ in water. After the evaporation of the solvent, the sample was pyrolyzed under nitrogen flow at 700 °C followed by a reduction step (N_2_/H_2_, 90/10) at 550 °C (Figure 1 A). Inductively coupled plasma optical emission spectrometry indicated 4.7 weight% (wt %) Co in the catalyst material. Homogeneously distributed Co nanoparticles with an average particle size of 6 nm were determined with transmission electron microscopy (Figure 1 B). The catalyst has a specific surface area (Brunauer‐Emmet‐Teller) of 367 m^2^ g^−1^ with primarily micropores and about 9 % mesopores. X‐ray photoelectron spectroscopy analysis confirms the presence of metallic Co species and oxi(hydroxide) Co species at the surface of the Co nanoparticles (Figure 1 C). High resolution transmission electron microscopy of the Co nanoparticles confirms that the core of these particles is metallic Co (cubic phase, Figure 1 D) and indicate an embedding of the Co nanoparticles into the *N*‐SiC support. In the diffraction data, I(Q), of the catalyst (Co/*N*‐SiC), the broad reflexes of graphite are still visible (Figure 1 E). Additionally, some reflexes due to Cobalt arise in the black curve. The first reflex (002) of the graphitic support is shifted slightly to lower Q values in the catalyst material due to the loading with Cobalt compared to the bare *N*‐SiC support, indicating expansion of the graphite interlayer spacing upon catalyst loading. A biphasic refinement of the d‐PDF data (Co/*N*‐SiC‐*N*‐SiC) reveals the coexistence of crystalline Co fcc nanoparticles with diameters of 5.4 nm and smaller CoO fcc domains, with a phase ratio of Co particles to CoO domains of about 6:1.

**Figure 1:**
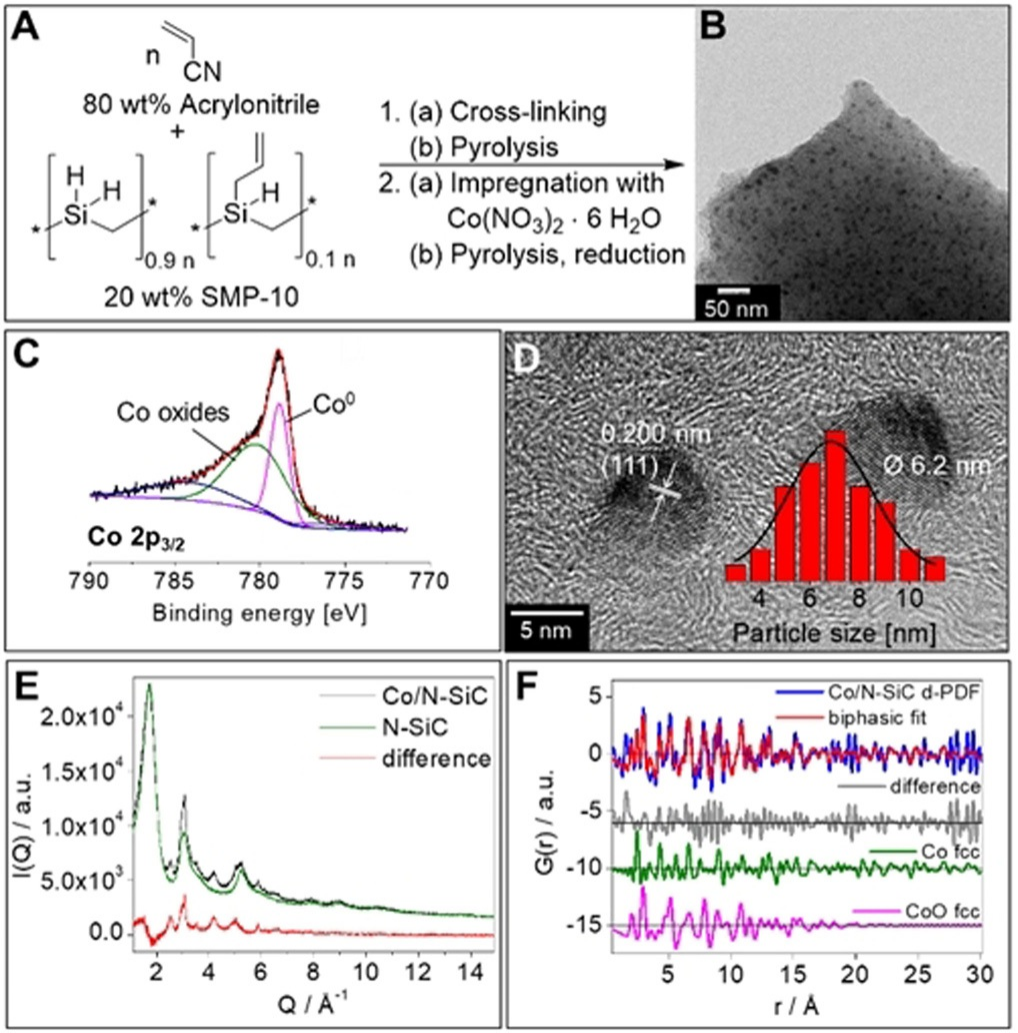
Synthesis and characterization of the Co catalyst denoted as Co/*N*‐SiC. A) Preparation of the cobalt (Co) catalyst. (a) Wet impregnation of *N*‐SiC with an aqueous solution of Co(NO_3_)_2_. (b) Pyrolysis of the impregnated *N*‐SiC under a nitrogen atmosphere at 700 °C and reduction of the pyrolyzed sample in the presence of hydrogen at 550 °C. B) Transmission electron microscopy analysis of the Co/*N*‐SiC catalyst and Co nanoparticle size distribution. Homogeneously dispersed Co nanoparticles with an average particle size of 6 nm. C) X‐ray photoelectron spectroscopy analysis verifies the presence of metallic Co and oxi(hydroxide) Co species (about 70 %) at the surface of the Co nanoparticles. D) Characterization of the Co nanoparticles by high resolution transmission electron microscopy indicates that they are embedded in the *N*‐SiC support and metallic E) XRD data of catalyst Co/*N*‐SiC (black) and support *N*‐SiC material (green), together with their difference (catalyst–support, in red). F) PDF refinement of the d‐PDF (Co/*N*‐SiC–*N*‐SiC), showing the contributions of the Co fcc phase (green) and of the CoO fcc phase (pink) to the biphasic fit (in offset for clarity), together with the difference curve (grey) only containing high frequency noise.

The reductive alkylation of benzonitrile with 4‐methylbenzaldehyde was chosen as a benchmark reaction for the optimization of the reaction conditions (Table 1, top). The most active catalyst based on the yield of product in a given time was obtained by wet impregnation of *N*‐SiC with Co(NO_3_)_2_ and a pyrolysis temperature of 700 °C (Table 1, entry 3). Varying the solvent (2‐methyltetrahydrofuran, diglyme, dioxane, triethylamine, methylcyclohexane, ethanol, toluene, water, isopropanol, pyridine), temperature, hydrogen pressure and ratio of nitrile to aldehyde led to a 90 % formation of [*N*‐benzyl‐1‐(*p*‐tolyl)methanamine] in 2‐methyltetrahydrofuran at 100 °C at a hydrogen pressure of 1.5 MPa and a nitrile‐to‐aldehyde ratio of 1:2 (Table S6–S9). The replacement of the metal source with other Co compounds led to a decrease in the yield of secondary amine (Table 1, entries 1 and 2). A minor decrease in activity was observed by using catalysts with Co(OAc)_2_ (OAcH=acetic acid) and Co(acac)_2_ (acacH=pentane‐2,4‐dione). Pyrolysis temperatures of 600 and 800 °C led to a significant decrease in the catalytic activity of the catalysts synthesized from Co(NO_3_)_2_ and *N*‐SiC (Table 1, entries 4 and 6). In addition, we investigated different catalyst supports (Table 1, entries 8 till 10; Table S13), which are all significantly less active.

**Table 1:** Catalyst screening.

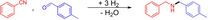				
Entry	Metal source	Support	Pyrolysis temp. [°C]	Yield [%]
1^[a]^	Co(OAc)_2_⋅6 H_2_O	*N*‐SiC	700	71
2^[a]^	Co(acac)_2_	*N*‐SiC	700	70
**3^[a]^**	**Co(NO_3_)_2_**⋅**6 H_2_O**	***N*** **‐SiC**	**700**	**87**
4^[b]^	Co(NO_3_)_2_⋅6 H_2_O	*N*‐SiC	600	48
**5^[b]^**	**Co(NO_3_)_2_**⋅**6 H_2_O**	***N*** **‐SiC**	**700**	**90**
6^[b]^	Co(NO_3_)_2_⋅6 H_2_O	*N*‐SiC	800	60
7^[c]^	–	*N*‐SiC	–	0
8^[b]^	Co(NO_3_)_2_⋅6 H_2_O	Pyrolyzed polyacrylonitrile	700	0
9^[b]^	Co(NO_3_)_2_⋅6 H_2_O	Activated charcoal	700	0
10^[b]^	Co(NO_3_)_2_⋅6 H_2_O	γ‐Al_2_O_3_	700	0

[a] Reaction conditions: 5.0 mol % Co (37 mg catalyst, 4.0 wt % Co, 0.025 mmol Co, 1.47 mg Co), 0.5 mmol benzonitrile, 1.0 mmol 4‐methylbenzaldehyde, 2 mL 2‐methyltetrahydrofuran (2‐MTHF), 90 °C, 1.5 MPa H_2_, 16 h. [b] conditions as above with only 3 mL 2‐methyltetrahydrofuran instead of 2 mL. Yields were determined by gas chromatography using *n*‐dodecane as an internal standard. Abbreviations in entries 1 and 2 are explained in the text.

The other supports led to inactive catalysts for which we could not observe any product formation under these conditions. The combination of Co(NO_3_)_2_ and the porous support material *N*‐SiC seem essential for the activity in the reductive alkylation of nitriles with carbonyl compounds under the optimized and mild conditions. We expected mild conditions to be crucial in order to achieve a desirable level of tolerance of functional groups.

We were interested in exploring the substrate scope and the functional group tolerance of our Co/*N*‐SiC catalyst next. Isolated yields of the secondary amines were given for the corresponding hydrochloride salts. For the reductive alkylation of benzonitrile with various aldehydes (Figure 2, products **1**–**19**), the secondary amines using 4‐methylbenzaldehyde (electron‐donating group) and 4‐chlorobenzaldehyde (electron‐withdrawing group) were isolated in 99 % yield (Figure 2, product **1**). In addition, the influence of the substituent position was investigated by converting 2‐, 3‐ or 4‐methylbenzaldehyde and 2‐, 3‐ or 4‐chlorobenzaldehyde. *meta*‐Substituted benzaldehydes showed no influence on the catalytic activity (Figure 2, products **2**, **5**), but a decrease in the product yields were observed for sterically more demanding 2‐methylbenzaldehyde and 2‐chlorobenzaldehyde (Figure 2, products **3**, **7**). In addition, other electron‐withdrawing substituents, such as fluorides and bromides, were tolerated well; this was indicative, especially for bromide **8**, that dehalogenation is of minor relevance (Figure 2, products **4**–**8**). The *tert*‐butyl substituent in the *para* position of benzaldehyde showed no influence on the product formation and the corresponding amine **9** was isolated in 99 % yield. Boronic esters, such as 4,4,5,5‐tetramethyl‐1,3,2‐dioxaborolan‐2‐yl)benzaldehyde, are of special importance, since they are common compounds for cross‐coupling reactions and one example could be isolated in excellent yield (Figure 2, **10**). Secondary amines with methoxy and benzyloxy substituents, which are used to protect hydroxyl groups, were also isolated in nearly quantitative yields (Figure 2, products **11**, **12**). Cyclic acetals, such as piperonal, which are common protective groups for carbonyl groups, were also tolerated (Figure 2, product **13**). In addition, purely aliphatic aldehydes can be employed (Figure 2, products **14**–**19**) albeit with lower isolated yields.

**Figure 2:**
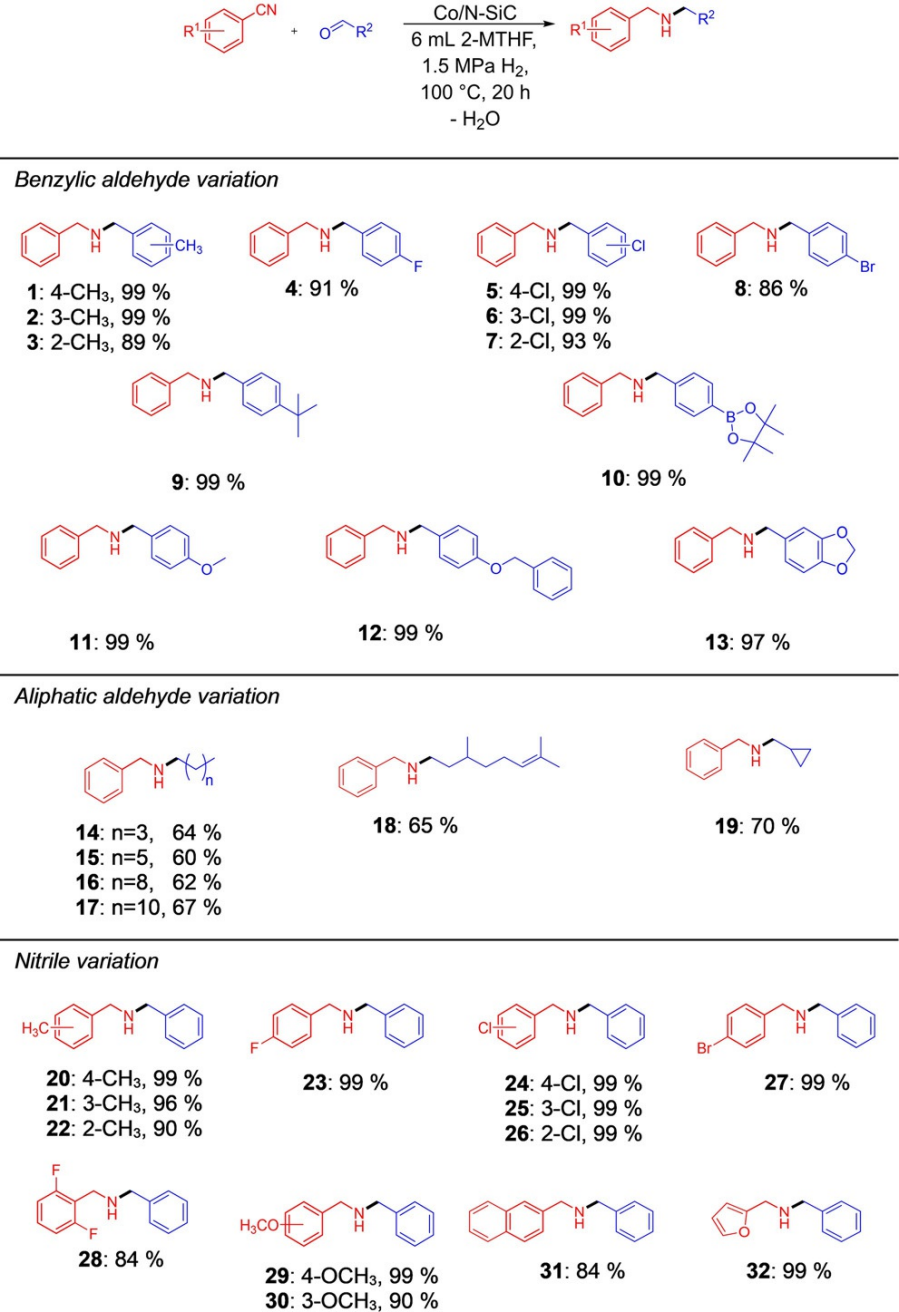
Scope of secondary amines using aromatic nitriles and aldehydes. Reaction conditions: 5.0 mol % Co (74 mg Co/*N*‐SiC, 4.0 wt % Co, 0.05 mmol Co, 2.95 mg Co), 1.0 mmol nitrile, 3.0 mmol aldehyde, 6 mL 2‐methyltetrahydrofuran, 100 °C, 1.5 MPa H_2_, 20 h. Isolated yields are given for the corresponding hydrochloride salts.

For the reductive alkylation of various benzonitriles and benzaldehyde, methyl group and chloro‐substituted benzonitriles were used to investigate the substituent position effect for both electron‐donating and ‐withdrawing substituents (Figure 2, products **20**–**22** and **24**–**26**). By converting 2‐, 3‐ or 4‐methylbenzonitrile, the yield decreases gradually from *para*‐ via *meta*‐ to *ortho*‐substituted nitriles (Figure 2, products **20**–**22**). This trend could not be observed at the chloro‐substituted nitriles, as the corresponding amines were isolated in 99 % yield (Figure 2, products **24**–**26**). A decrease of the product yield was observed by converting 2,6‐difluorobenzonitrile, which we believe is due to the two electron‐withdrawing fluoro substituents (Figure 2, product **28**). The electron‐donating methoxy substituent in the *para* position had no negative influence on the yield of the corresponding amine, which was isolated in 99 % yield (Figure 2, product **29**).

However, a slight decrease in the yield could be observed for the methoxy substituent in the *meta* position (Figure 2, product **30**). The naphthalene‐based nitrile can be converted in good yields to the corresponding secondary amine **31**. To our delight, the reductive alkylation of an aromatic *O*‐heterocycle proceeded very well and the corresponding amine **32** was isolated in nearly quantitative yield.

For the reductive alkylation of aliphatic nitriles with various aldehydes (Figure 3), as before, the influence of the substituent position was investigated with the methyl‐ and chloro‐substituted benzaldehydes (Figure 3, products **33**–**35**, **37**–**39**). The same trend was observed, but the yields of the methyl‐substituted secondary amines **33**, **34** and **35** were slightly lower, due to the lower reactivity of the aliphatic nitrile. The secondary amines with the electron‐withdrawing chloro substituents at the 2‐, 3‐ or 4‐position were obtained in 96–99 % isolated yield (Figure 3, products **37**–**39**). Fluorinated and brominated benzaldehydes were smoothly converted in the corresponding amines **36** and **40** in good yields. The secondary amine **41** with the electron‐donating methoxy substituent in the *para* position was isolated in 96 % yield. 4‐Benzyloxybenzaldehyde was converted selectively to product **42** in nearly quantitative yield without a significant amount of hydrogenolytic ether cleavage. Piperonal was converted to the corresponding amine **43** in very good yield, no cleavage of the acetal was observed and a yield of 99 % was observed for 4‐*tert*‐butylbenzaldehyde (Figure 3, product **44**). We then varied the aliphatic nitrile and combined it with numerous, mostly purely aliphatic aldehydes. Purely aliphatic nitriles of different chain lengths were linked smoothly with benzaldehyde and the secondary amines were isolated in yields of 99 % (Figure 3, products **45**, **46**). The synthesis of such alkyl‐benzylamines seems more efficient if an aliphatic nitrile is used instead of an aliphatic aldehyde. A nitrile with a cyclohexane substituent was converted to the product **47** in good yields too. The sterically demanding pivalonitrile reacts well and the coupling product with benzaldehyde was obtained in 82 % yield (Figure 3, product **48**). The unsaturated secondary amine **49**, which was synthesized from valeronitrile and citronellal, was still isolated in an acceptable isolated yield of 62 %. Various pure aliphatic nitriles and aldehydes with different chain lengths were smoothly converted to the corresponding amines (Figure 3, products **50**–**54**); no influence of the chain length on the yield were observed.

**Figure 3:**
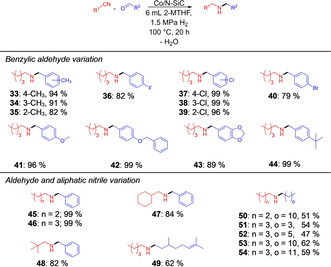
Scope of secondary amines using aromatic nitriles and aldehydes. Reaction conditions: 5.0 mol % Co (74 mg Co/*N*‐SiC, 4.0 wt % Co, 0.05 mmol Co, 2.95 mg Co), 1.0 mmol nitrile, 3.0 mmol aldehyde, 6 mL 2‐methyltetrahydrofuran, 100 °C, 1.5 MPa H_2_, 20 h. Isolated yields are given for the corresponding hydrochloride salts.

For reductive alkylation of nitriles with ketones, the imine intermediate formed after the condensation is sterically more protected and, thus, more difficult to hydrogenate. Higher reaction temperatures (110 °C) and catalyst loadings of 8 mol % Co were required to obtain good yields. We systematically explored the coupling of an aromatic nitrile, namely, benzonitrile or a purely aliphatic nitrile, namely, pentanenitrile with a diaryl, aryl‐alkyl, dialkyl or cyclic ketone. Isolated yields between 57 and 81 % were obtained (Figure 4, product **55** till **62**). The yield of the secondary amines in the reductive alkylation of benzonitrile decreases gradually from diaryl via aryl‐alky to dialkyl ketones (Figure 4, products **55**–**57**). The cyclic ketone was converted smoothly in the secondary amine **58**. The yield (85 %) was, compared to the other ketones, higher due to a smaller steric demand. The same trends but lower isolated yields were observed for the reaction of the aliphatic nitrile pentanenitrile with the same ketones (Figure 4, products **59**–**62**).

**Figure 4:**
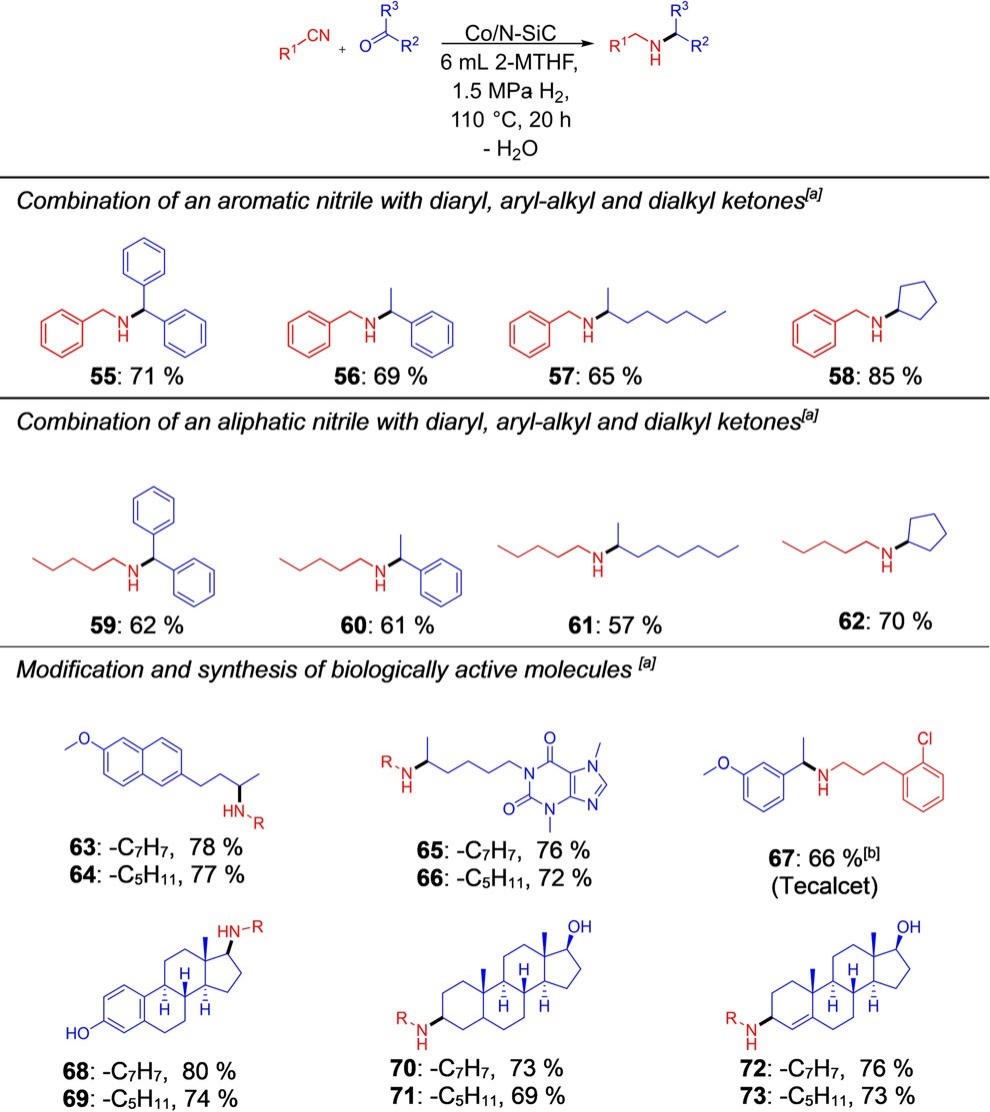
Reductive alkylation of nitriles using ketones and modifications and synthesis of biologically active molecules. [a] Reaction conditions: 8.0 mol % Co (118 mg Co/*N*‐SiC, 4.0 wt % Co, 0.08 mmol Co, 4.71 mg Co), 1.0 mmol nitrile, 3.0 mmol ketone, 6 mL 2‐methyltetrahydrofuran, 110 °C, 1.5 MPa H_2_, 20 h. Isolated yields are given for the corresponding hydrochloride salts. [b] 130 °C.

For the modification and synthesis of biologically active molecules, nabumentone and pentoxifylline, common drug molecules, were converted to the respective amines in up to 78 % isolated yield (Figure 4, products **63**–**66**). To our delight, pentoxifylline was converted without hydrogenation of the C=O bond. Our synthesis protocol may also be applied to synthesize biologically active molecules. Tecalcet, which is used as a calcimimetic agent, can be synthesized from the two commercially available educts 2‐chlorohydrocinnamonitrile and 3‐methoxyacetophenone in 66 % isolated yield (Figure 4, product **67**). Nitriles can also be alkylated with various steroid derivatives. The reductive alkylation of benzonitrile and valeronitrile with estrone, stanolone and testosterone proceeded smoothly, and the products were obtained in yields up to 80 % (Figure 4, products **68**–**73**). Testosterone, which contains the C−C double bond, was converted into the corresponding unsaturated amines **72** and **73**. The yields of the products, which were synthesized with an aliphatic nitrile, were slightly lower than those obtained with an aromatic nitrile. Various tests were performed to demonstrate the catalyst stability and leaching of cobalt species (see Supporting Information). The reductive alkylation of benzonitrile with 4‐methylbenzaldehyde at about 65 % yield of product was chosen to demonstrate the reusability of the catalyst and to establish its efficiency. Five consecutive runs showed no decrease in the catalytic activity (Figure S10). An upscaling of the reaction was performed using various substrates. Therefore, 5 mmol of the nitriles was converted into the secondary alkylamines on a gram scale. No decrease of the yield obtained was observed as the reaction was upscaled (Table S14).

We propose a hydrogenation–condensation–hydrogenation pathway regarding the reductive alkylation of nitriles with carbonyl compounds as shown in Scheme 2, top. Mechanistic investigations were carried out under the given conditions (Scheme 2, bottom), and product and intermediates are obtained in the yields listed. We used milder reaction conditions to better distinguish between the rates of the individual reaction steps. The hydrogenation of the nitrile proceeds slowly in comparison to condensation and imine hydrogenation and is probably the rate‐determining step in the product formation sequence. The benzaldehyde is slowly converted to the corresponding alcohol (23 % under the conditions given) if no nitrile is present. No hydrogenation of benzaldehyde to the corresponding alcohol was observed if the reaction is performed in the presence of nitriles.

**Scheme 2:**
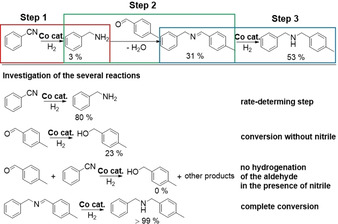
Proposed hydrogenation–condensation–hydrogenation pathway regarding the reductive alkylation of nitriles with carbonyl compounds (top). Mechanistic investigations (bottom). Reaction conditions: 5.0 mol % Co (37 mg catalyst, 4.0 wt % Co, 0.025 mmol Co, 1.47 mg Co), 0.5 mmol benzonitrile, 1.5 mmol 4‐methylbenzaldehyde, 3 mL 2‐methyltetrahydrofuran, 80 °C, 1.5 MPa H_2_, 16 h. Under these conditions, products and intermediates are obtained in the yields given. These yields were determined by gas chromatography using *n*‐dodecane as an internal standard.

## Conflict of interest

The authors declare no conflict of interest.

## Supporting information

As a service to our authors and readers, this journal provides supporting information supplied by the authors. Such materials are peer reviewed and may be re‐organized for online delivery, but are not copy‐edited or typeset. Technical support issues arising from supporting information (other than missing files) should be addressed to the authors.

Supplementary
