# Supplementary material for: General Synthesis of Secondary Alkylamines by Reductive Alkylation of Nitriles by Aldehydes and Ketones
Source: Chemistry. 2020 Dec 21;27(5):1609–14. doi: 10.1002/chem.202004755 (PMC7898800; doi:10.1002/chem.202004755)
Supplement: Supplementary file 1 — Supplementary [file CHEM-27-1609-s001.pdf]

Chemistry–A European Journal

Supporting Information

General Synthesis of Secondary Alkylamines by Reductive Alkylation of Nitriles by Aldehydes and Ketones

Timon Schönauer,[a] Sabrina L. J. Thomä,[b] Leah Kaiser,[a] Mirijam Zobel,[b] and Rhett Kempe*[a]

Table of Contents

1. General considerations 2
2. Experimental procedures 4
   Catalyst synthesis 4
   Catalyst characterization 4
3. Catalytic studies 13
   Screening of reaction parameters 13
   Reductive alkylation of nitriles with aldehydes – general procedure 17
   Reductive alkylation of nitriles with ketones – general procedure 17
   Evaluation of the catalyst stability 18
   Catalyst recycling 20
   Up-scaling 21
4. Additional literature 22
   Hydrogenation of amides 22
5. Characterization of isolated products 23
6. NMR spectra 48
7. References 122

1. General considerations

Air- and moisture sensitive reactions were carried out under dry argon or nitrogen atmosphere using standard Schlenk or glove box techniques. Solvents were dried and distilled from sodium benzophenone, stored over molecular sieves (3 Å) before use. All chemicals were purchased from commercial sources with purity over 95 % and used without further purification. StarPCS^{TM} SMP-10 was purchased from Starfire and used without further purifications. Acrylonitrile was purchased from Sigma-Aldrich and before using the inhibitor was removed with Al_2O_3.

NMR-Spectra were collected on Varian INOVA 300 (300 MHz for ^{1}H, 75 MHz for ^{13}C), Varian INOVA 400 (400 MHz for ^{1}H, 100 MHz for ^{13}C) or Bruker Avance III HD 500 (500 MHz for ^{1}H, 125 MHz for ^{13}C) instruments at 298 K. Chemical shifts are reported in ppm relative to the residual solvent signal (DMSO-D_6: 2.50 ppm (^{1}H), 39.51 ppm (^{13}C)). Coupling constants (*J*) are reported in Hz (coupling patterns: s = singlet, d = doublet, t = triplet, q = quartet, quint. = quintet, sxt = sextet, spt = septet, m = multiplet).

GC analyses were carried out on an Agilent 6850 GC system equipped with an Optima 17 column (30 m x 0.32 mm x 0.25 µm) or an Agilent 6890N GC system equipped with a HP-5 column (30 m x 0.32 mm x 0.25 µm). GC-MS analyses were carried out on an Agilent 7890A GC system equipped with a HP-5MS column (30 m x 0.32 mm x 0.25 µm) and a 5975C inert MSD detector (EI, 70 eV).

Transmission electron microscopy (TEM) was carried out by using a LEO 922o (200 kV) instrument and a JEOL JEM 2200FS (200 kV). The sample was suspended in chloroform and sonicated for 5 min. Subsequently 2 µL of the suspended sample was placed on a CF200-Cu-grid or a LC200-Cu-grid (Electron Microscopy Sciences) and allowed to dry.

Scanning electron microscopy (SEM) and energy dispersive X-ray spectroscopy (EDX) measurements were carried out by using a Zeiss Ultra plus. The acceleration voltage was 20 kV.

The carbon amount was determined by combustion analysis with a carbon analyser Leco C-200 using SiC as standard and the nitrogen and oxygen content by hot gas extraction with a Leco TC-436 N/O analyser using TiN and WO_3 as standards. Elemental analyses of the hydrochloride salts were performed using the Elementar UNICUBE®.

The cobalt content was determined by ICP-OES. The fusion of the catalyst was carried out in a Berghof SpeedWave 4 microwave, for the ICP-OES measurement a Varian Vista Pro was used.

The X-ray photoelectron spectroscopy (XPS) measurements were conducted in a PHI Vesa Probe III apparatus. A monochromatic Al K*α* x-ray source (1486.6 eV) was used for excitation. The analysis was conducted along the lines of Refs.

X-ray powder diffractograms were received by a PANalytical X'Pert EMPYREAN (CuKα-radiation 1.54178 Å) in Bragg-Brentano-geometry and with a PIXcel^{1D} detector. The reference codes are 00-015-0806 for the cubic cobalt and 00-041-1487 for hexagonal Graphite-2*H*.

N_2 physisorption measurements were determined at -196 °C using a Nova2000e (Quantachrome) apparatus. The specific surface areas were calculated using p/p_0 values from 0.05-0.3 (BET). The pore width and average pore volume were calculated by DFT calculations [N_2 at -196.15 °C on carbon (slit pore model, NLDFT equilibrium model)].

Pyrolysis was carried out under nitrogen atmosphere in a high temperature furnace (Gero, Berlin, Germany) and reduction under nitrogen atmosphere and forming gas (N_2/H_2, 90/10) using ChemBET Pulsar TPR/TPD. Milling of the support material was performed in a ball mill "Pulverisette 0" (Fritsch, Germany) for 40 min.

Powder X-ray diffraction (XRD) data was collected at room temperature on a STOE STADI P Mythen2 4K diffractometer (Ge(111) monochromator; Ag Kα_1 radiation, λ = 0.5594 Å), optimized for PDF data collection using four Dectris MYTHEN2 R 1K detectors in Debye–Scherrer geometry.[1] Samples were measured in 0.5 mm glass capillaries (Hilgenberg special-purpose glass) for 24 h. For PDF processing, the employed Q-range was 1.0 - 15.0 Å^{-1}. Here, Q is the momentum transfer calculated from the wavelength λ and the scattering angle 2θ according to $Q = \frac{4\pi}{\lambda}\sin(\theta)$. PDF calculation was done with PDFgetX3,[2] using the composition of $SiC_{9.36}N_{0.99}O_{1.62}$ for the support (as determined by CHN analytics, and an empty glass capillary as background) and a nominal composition of CoNSiC for the calculation of the difference-PDF of the catalyst using the unloaded support as background. For PDF modelling we used PDFgui.[3] Cif files for PDF modelling were downloaded from the American Mineralogist Crystal Structure Database (AMCSD)[4] or created from published structures.[5]

2. Experimental procedures

Catalyst synthesis

Support synthesis

The applied support material was modified according to known literature.[6] 0.200 g StarPCS^{TM} SMP-10, 0.988 mL (0.800 g, 15.08 mmol) acrylonitrile (AN) and 0.075 g (0.46 mmol) azobisisobutyronitrile (AIBN) were dissolved in 4 mL dimethylformamide (DMF) and heated up to 75 °C for 5 h under aerobic conditions. After removal of the solvent, the yellowish solid was pyrolyzed under N_2 atmosphere with the following heating program:

$$\text{RT} \xrightarrow{\text{5 K/min}} \text{200 °C} \xrightarrow{\text{1 K/min}} \text{400 °C} \xrightarrow{\text{5 K/min}} \text{1000 °C (1 h)} \xrightarrow{\text{5 K/min}} \text{RT}$$

The mass loss after pyrolysis was 45 %. After ball milling for 40 minutes, 500 mg support material were washed by stirring in an aqueous solution of 6.7 mL NaOH (c = 1 mol/l) and 5 mL MeOH at 80 °C for 20 h under aerobic conditions. Other mass ratios of AN/SMP-10 (100/0, 50/50, 80/20, 0/100) were synthesized equally.

Wet impregnation

To an aqueous solution (10 mL H_2O) of 102.9 mg (0.35 mmol) $Co(NO_3)_2 \cdot 6\ H_2O$, 500 mg support N-SiC were added and the suspension was stirred at 105 °C. After evaporation of the solvent, the sample was pyrolyzed under nitrogen atmosphere at 700 °C followed by reduction at 550 °C (N_2/H_2, 90/10) with the following heating program:

$$\text{RT} \xrightarrow[N_2]{\text{2 K/min}} \text{300 °C} \xrightarrow[N_2]{\text{10 K/min}} \text{700 °C (0.5 h)} \xrightarrow[N_2]{\text{20 K/min}} \text{100 °C} \xrightarrow[N_2/H_2]{\text{5 K/min}} \text{550 °C (3 h)} \xrightarrow[N_2/H_2]{\text{20 K/min}} \text{40 °C}$$

The catalysts used for the screening reactions were synthesized using equivalent amounts of support material (silica, activated charcoal (Norit CA1), γ-Al_2O_3) or metal salt.

Catalyst characterization

ICP-OES

25 mg of the sample were solved in 1.5 mL HNO_3 (65 %, distilled), 4.5 mL HCl (32 %, p.a.) and 1 mL HF (40 %) and heated in the microwave at 170 °C for 7 min (80 % power), at 180 °C for 7 min (85 % power) and at 195 °C for 20 min (90 % power).

Theoretical Co content: 4.0 wt%
Measured Co content: 4.7 wt%

Leaching experiment: A mixture of 37 mg Co catalyst, 3 mL 2-methyltetrahydrofuran were stirred at 100 °C and 15 bar H_2 pressure for 20 hours. The catalyst was removed and the resulting solution was analyzed by ICP-OES. 0.05 % of the total Co amount were found.

Elemental analysis

Table S1: Elemental analysis of the support material N-SiC and pyrolyzed polyacrylonitrile.

	**Si [at%]**	**C [at%]**	**N [at%]**	**O [at%]**	**Resulting chemical formula**
N-SiC	7.7	72.2	7.6	12.5	$SiC_{9.36}N_{0.99}O_{1.62}$
Pyrolyzed polyacrylonitrile	0.0	79.7	14.0	6.3	$C_{5.68}NO_{0.45}$

The silicon content was calculated as the difference of the abovementioned elements to 100%.

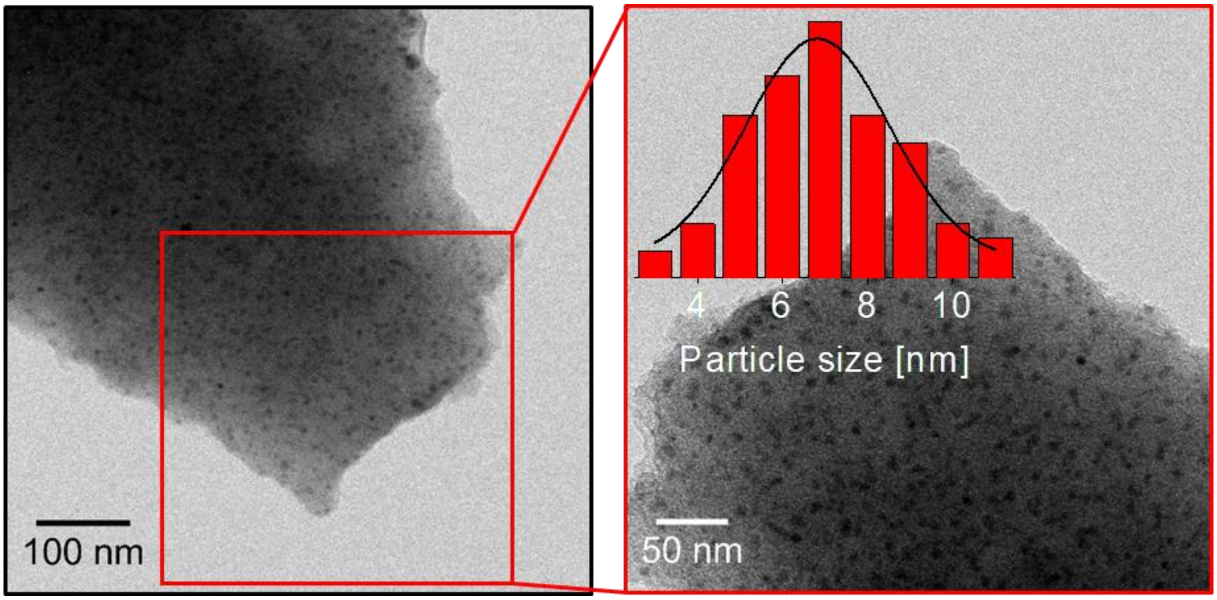

Figure S1: Transmission electron microscopy (TEM) analysis of the Co/N-SiC catalyst and Co nanoparticle size distribution. Homogeneously dispersed cobalt nanoparticles with a particle size distribution centered at 6.2 nm were observed.

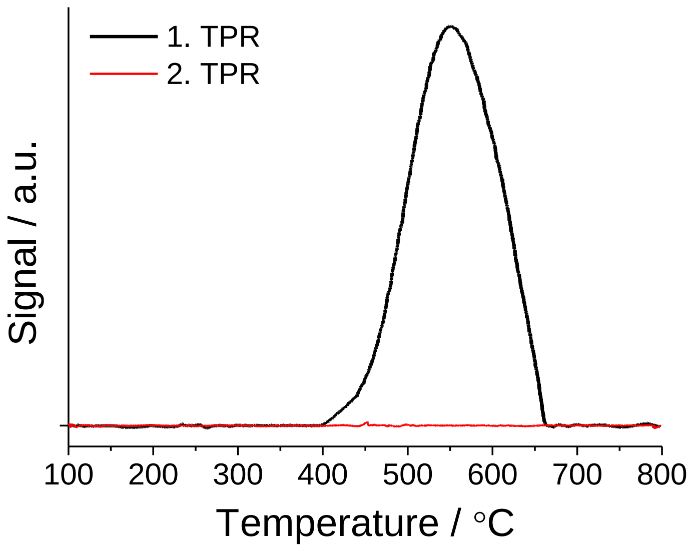

Figure S2: Temperature programmed reduction (TPR) after pyrolysis of the wet impregnated N-SiC support. The N-SiC support was wet impregnated with $Co(NO_3)_2 \cdot 6\ H_2O$ and heated up to 700 °C with 1 K/min under reductive atmosphere (N_2/H_2, 90/10). The first TPR (black) was performed after the pyrolysis. A broad signal between 400 and 650 °C indicates the presence of reducible cobalt species on the support material. The second TPR (red) was accomplished subsequently after the first TPR (black). In the second run, no hydrogen uptake was recorded, which indicates a complete reduction of the cobalt oxide species.

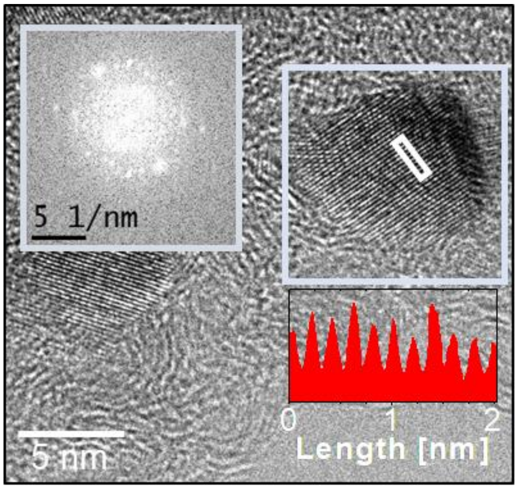

Figure S3: Characterization of the cobalt nanoparticles by high resolution TEM. The image indicates the presence of Co nanoparticles on the N-SiC support. The characteristic lattice planes - (111) reflex of cubic crystalline Co - indicates the presence of metallic cobalt in the core of the particles. The analysis resulted in a d-spacing of 0.200 nm, which is in accordance with the expected value of 0.204 nm of the (111) reflex of cubic crystalline Co.

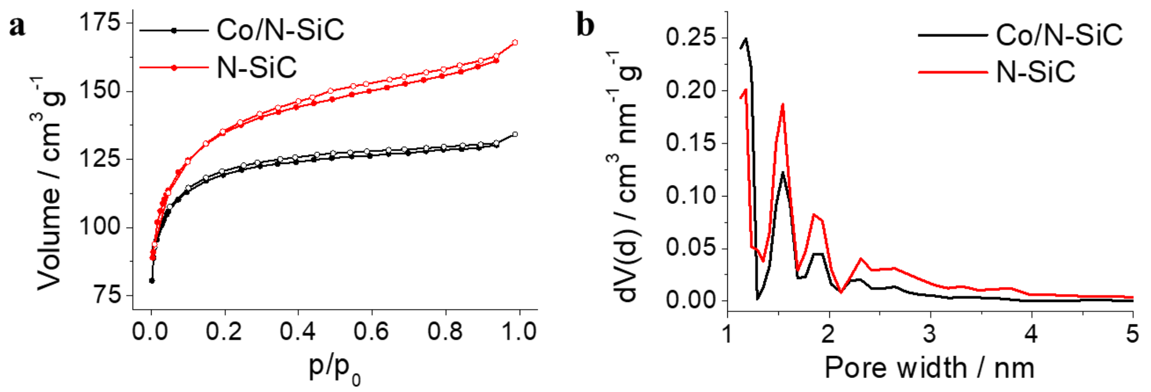

Figure S4: Nitrogen physisorption measurements of the catalyst and the support material N-SiC. (a) Isotherms of the Co/N-SiC catalyst (black) and N-SiC support (red). Both materials show the typical hysteresis of porous materials. Due to the impregnation of the N-SiC support with $Co(NO_3)_2 \cdot 6\ H_2O$, only a slight decrease from 423 to 367 $m^2\ g^{-1}$ of the surface area can be observed. **(b)** Corresponding calculated NLDFT pore size distributions {[N_2 at -196.15 °C on carbon (slit pore model, NLDFT equilibrium model)]: Co/N-SiC (black), N-SiC (red)} show no significant differences for both samples.

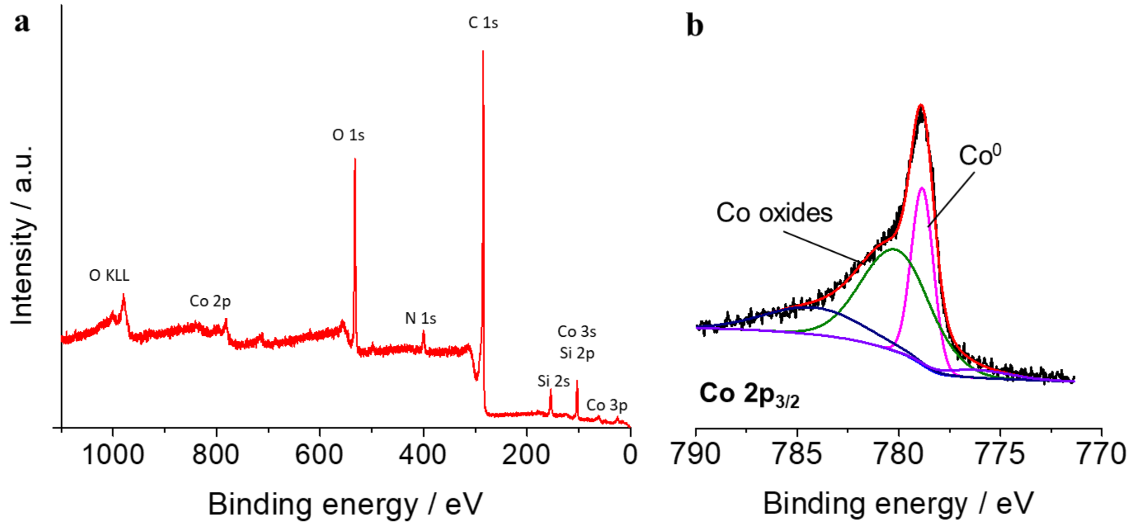

Figure S5: X-ray photoelectron spectroscopy (XPS) analysis of the Co/N-SiC catalyst. (a) The Co/N-SiC catalyst contains Si, C, N, O and Co. **(b)** An analysis of the Co $2p_{3/2}$ region verifies the presence of metallic cobalt, which can be identified due to its sharp line and its binding energy of 779 eV (28 % of the signal). At higher binding energies, different oxides and maybe also hydroxides are found that cannot be distinguished.

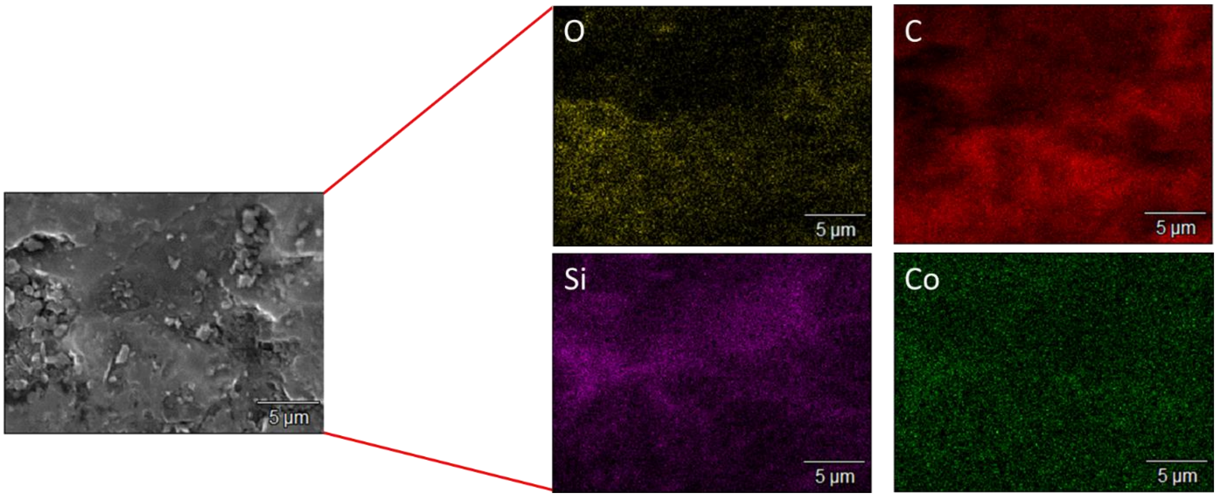

Figure S6: Scanning electron microscopy (SEM) in combination with energy-dispersive X-ray (EDX) element maps of the Co/N-SiC catalyst. The cobalt nanoparticles were homogeneously distributed over the entire support material which indicates a smooth impregnation process. The homogenous distribution of Si, C and O verifies a homogeneous support material.

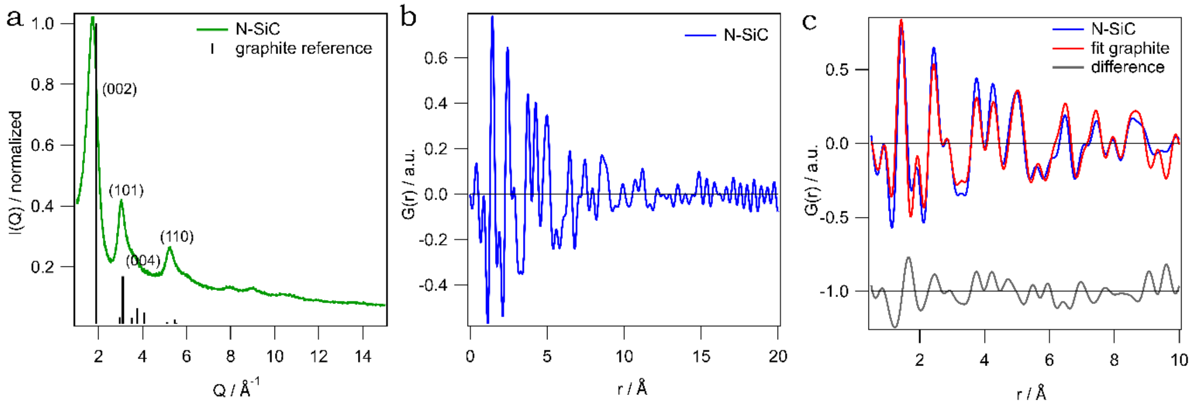

Figure S7: PXRD and PDF data of N-SiC support material. (a) PXRD indexed with hexagonal graphite (structure from AMCSD database code: 0011247). **(b)** PDF data showing sharp interatomic distances from short-range order over ca. 10 Å, with only high frequeny noise beyond. **(c)** Refinement of N-SiC PDF data (blue) with a model of hexagonal graphite (red) and the difference (grey, in offset).

The reference pattern of graphite indexes several peaks of the PXRD data reasonably well. The (002) reflex of N-SiC is slightly shifted compared to hexagonal graphite. To evaluate this further, the peak positions in Q and corresponding d-spacings ($d = 2\pi/Q$) of the reflexes (002) and (110) are compared for the N-SiC support and the Co/N-SiC catalyst from the experimental data, and for bulk graphite from its known structure (code amcsd: 0011247) in Table S2. The in-plane reflex (110) of our support material N-SiC is only slightly shifted to higher values compared to graphite, resulting in slightly smaller lattice spacings, but not affected strongly. The (002) reflex contains information about the c-axis, i.e. the interlayer spacings, and is shifted to smaller Q-values by 0.147 $Å^{-1}$ for N-SiC, and by 0.217 $Å^{-1}$ for the catalyst Co/N-SiC. This reflects an expansion of the interlayer spacing compared to graphite. It can be explained by the fact that the N-SiC catalyst is a N- and Si-doped graphitic material. It is known, that high doping levels with nitrogen (above

20 %)[7] and Si lead to corrugated graphite sheets and that e.g. 10.1 % nitrogen-doping in graphite can be directly achieved during synthesis from organic starting materials such as Melamin[8] For Si, the different radii for Si and C result in rippled surfaces as well.[9] For instance, NiC nanotubes feature interlayer spacings of ca. 0.38 nm compared to only 0.34 nm for pure carbon nanotubes.[9] We see exactly the same trend here, that our N-SiC support features an interlayer spacing of 0.36 nm. For the Co/N-SiC catalyst, this interlayer spacing increases further to 0.38 nm, while the in-plane structure (i.e. (110) reflex) is not affected. This points to the fact, that the individual graphite sheets remain rather intact during the synthesis of the catalyst, which mainly affects the ordering along the crystallographic c-axis, i.e. the interlayer spacing. In Figure 2f, the difference curve of the catalyst minus support is displayed in red, featuring a broad dip between 1.5 – 2.4 Å^{-1}. This dip again corroborates, that structural signal is lost in the catalyst compared to the support in this Q-range, which corresponds to the interlayer spacing.

Table S2: Positions in Q and corresponding lattice spacings d of the two prominent reflexes (002) and (110) of the graphite structure as given by theory for graphite[4] and experimentally determined for our support and catalyst material. For positions and lattice spacings for support and catalyst material only three valid digits in comparison to the reference material (five valid digits) are given, because the experimental reflexes of the not perfectly crystalline support are broader and asymmetric.

	**Q (A^{-1})**		**d (nm)**	
	**(002)**	**(110)**	**(002)**	**(110)**
Graphite	1.8770	5.1164	0.33474	0.12280
N-SiC	1.73	5.26	0.363	0.119
Co/N-SiC	1.67	5.26	0.376	0.119

To understand the short-range order of the support and the catalyst materials beyond, PDF analysis was carried out. The support material N-SiC features short-range order up to about 10 Å, displayed by sharp peaks corresponding to interatomic distances within the material, see Figure S7, panel B. Above, only high frequency ripples due to noise are present. The N-SiC PDF data was fitted with the graphite structure up to 10 Å in Figure S7, panel C. The fit of pure graphite to the N-SiC catalyst describes the data astonishingly well, pointing towards a rather isostructural doping of the N and Si into the graphite lattice. Nitrogen scatters quite comparably to carbon (only one electron difference) and the discrepancies between data and fit are thus either due to the Silicon atoms (more electrons, resulting in higher PDF peaks) and / or structural changes induced in the graphite due to the N- and Si-dopants. The resulting fit parameters, are listed in Table S3 together with initial lattice parameters in brackets. The refined lattice parameter for the c-axis supports again the expansion of the interlayer spacing compared to bulk graphite as derived above based on the interpretation of the (002) reflex in reciprocal space. The large isotropic atomic displacement parameter u_{33} corroborates also higher disorder along c, and was used before to describe reduced interlayer correlations and simulate turbostratic disorder.[9] For graphitic structures in wood, Poulain et al.[10] found that higher inorganic fractions such as $Ca(OH)_2$ result in higher average interlayer spacings. Here, we observe the same effect that Cobalt species lead to a further increase in the interlayer spacing of the catalyst support as well.

Table S3: Results of PDF refinement of graphite model to N-SiC PDF data. a, b, c: unit cell parameter (starting values in brackets); scale factor to match theoretical and experimental PDF data in intensity; u_{11}, u_{22}, u_{33}: isotropic atomic displacement parameters with $B_{iso} = 8\pi^2 u_{iso}$,[11] delta2: parameter to account for correlated atomic motion; R_w: goodness-of-fit

a,b	2.4608 (2.546)
c	7.0012 (6.696)
scale factor	0.1022
u_{11}, u_{22}	0.0071
u_{33}	0.2533
delta 2	2.0058
**R_w**	**0.35**

Subsequently, a PDF refinement of the difference-PDF of the Co/N-SiC catalyst materials after subtraction of the N-SiC support was carried out, fitting spherical Co fcc particles (red curve) to the experimental d-PDF (blue), see Figure S8, panel A. The resulting fit parameters are contained in Table S4. The fit refined a Co fcc particle diameter of ca. 6.5 nm, which coincides very well with Co fcc particle diameters as retrieved from TEM analysis (average diameter of 6.2 nm). The difference curve (grey, in offset undoubtedly contains remaining structural signal in three peaks at 3.02, 5.16 and 7.82 Å, which lie above the rather high frequency noise. The latter stems from the rather low concentration of Co on the support material and having subtracted the large signal from the support material from laboratory PDF data only.

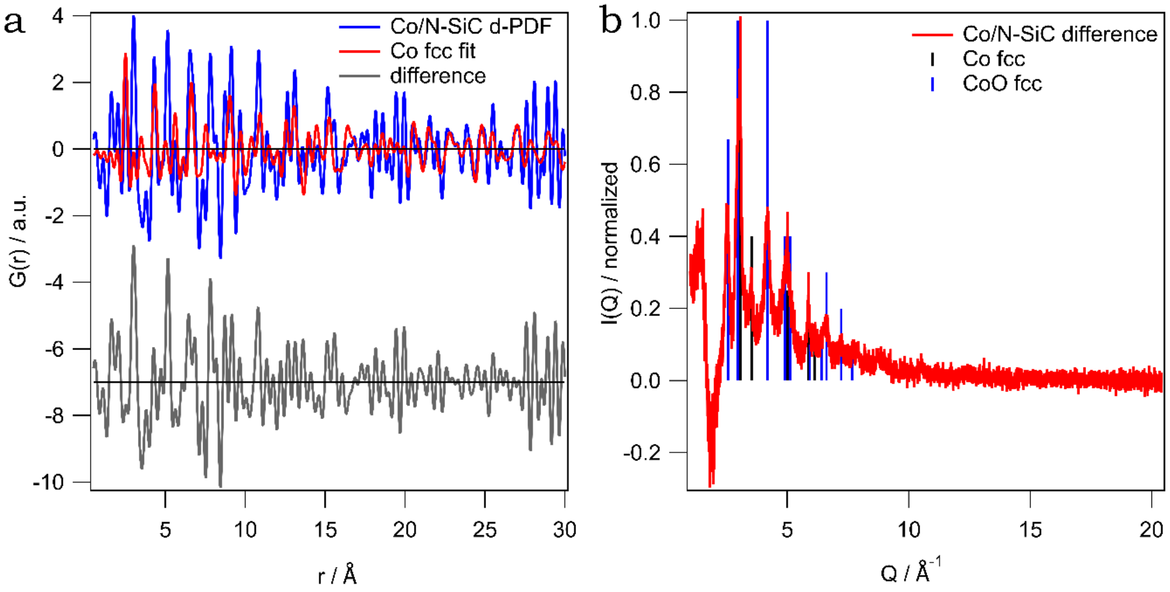

Figure S8: Difference-PDF and difference-XRD data showing existence of Co phases. (a) PDF refinement of Co fcc (red) to experimental d-PDF of Co/N-SiC (blue) with difference (grey, in offset). The difference curve reveals three peaks at < 10 Å containing remaining structural signal. **(b)** Difference XRD pattern (taken from Figure 2f) after subtraction of N-SiC support from Co/N-SiC catalyst, indexed with Co fcc and CoO fcc structure.

To find a structural match to the remaining structural signal in the difference curve of the PDF fit, the red difference curve of the PXRD data from Figure 2f is indexed further in Figure S8, panel B. Also here, indexing only with the reference pattern of the Co fcc structure (black lines), the reflexes at 2.5, 4.1 and above 5.8 $Å^{-1}$ are not indexed. Since XPS confirmed the presence of Co oxides, reference patterns for different crystalline cobalt oxide species were indexed with the software X-Pert Highscore. Only with a reference pattern of CoO (cubic, $Fm\overline{3}m$, space group 225, fcc)[7] all reflexes could be indexed (blue lines in Figure S8b).

Table S4: Fit results from refinement of Co fcc structure to d-PDF data of Co/N-SiC (spdiameter: finite particle size).

a, b, c	3.5493 (3.548)
a, b, c	3.5493 (3.548)
scale factor	0.1640
spdiameter	65.4309
u_{11}, u_{22}, u_{33}	0.0088
delta 2	5.5786
**R_w**	**0.87**

The simultaneous PDF refinement of a CoO phase alongside the Co fcc phase (see Figure 2g) can explicitly describe the three remaining PDF peaks and improves the goodness-of-fit value R_w from 0.87 to 0.64. The results from this simultaneous fit are contained in Table S5. Given the high noise in the data, the refinement is very satisfactory and further addition of phases or more detailed modelling would overinterpret the data. The refined CoO domain sizes feature a diameter of 2.5 nm and are significantly smaller than the Co fcc nanoparticles, whose size reduces to ca. 5.4 nm for the biphasic refinement.

Table S5: Results from biphasic simultaneous refinement of Co fcc and CoO fcc phases to d-PDF data of Co/N-SiC.

**Co fcc**		**CoO fcc**	
a, b, c	3.5515 (3.548)	a, b, c	4.2451 (4.24)
scale factor	0.1735	scale factor	0.4795
spdiameter	53.6189	spdiameter	24.6943
u_{11}, u_{22}, u_{33}	0.0082	Co u_{11}, u_{22}, u_{33}	0.01485
		O u_{11}, u_{22}, u_{33}	0.01648
delta 2	6.2951	delta 2	1.1143
**R_w**	**0.64**		

To evaluate the ratio of the two Co phases (Co:CoO), the two scale factors can be converted using the number of Co atoms per unit cell (4 each in Co fcc and CoO), the unit cell volume $V_{unitcell} = a^3$, and the volume of the spherical nanoparticles V_{NP} (spdiameters corresponds to particle diameter).

The number of unit cells per nanoparticles (#unit $cell_{NP}$) can be determined as:

$$\#unit\ cell_{NP} = \frac{V_{nanoparticle}}{V_{unit\ cell}}$$

$$\frac{\#Co_{\ Co-NP}}{\#Co_{CoO-NP}} = \frac{\#unit\ cell_{Co-NP}}{\#unit\ cell_{CoO-NP}}$$

It results that the number of Co atoms in the Co nanoparticles is 17.48 times the number of Co atoms in CoO domains. The final ratio of the two phases is calculated with the scale factors determined by PDFgui:

$$ratio = \frac{scale\ factor\ Co \cdot 17.48}{scale\ factor\ CoO} = 6.33$$

The Co/N-SiC catalyst thus contains about the 6-fold amount of Co fcc phase in comparison to the CoO fcc phase.

3. Catalytic studies

Screening of reaction parameters

Table S6: Screening of reaction parameters – solvent.

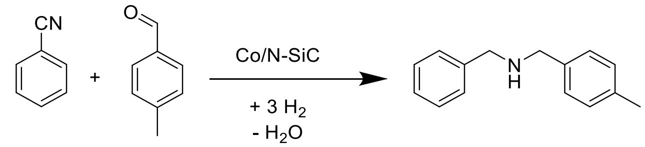

Entry	Solvent	Yield [%]
1	2-methyltetrahydrofuran	87
2	diglyme	81
3	dioxane	12
4	triethylamine	66
5	methylcyclohexane	79
6	ethanol	77
7	toluene	77
8	water	40
9	isopropanol	10
10	pyridine	33

Reaction conditions: 0.5 mmol benzonitrile, 1.0 mmol 4-methylbenzaldehyde, 37 mg catalyst (5 mol% Co), 90 °C, 1.5 MPa H_2, 2 mL solvent, 16 h; yields were determined by GC using *n*-dodecane as an internal standard.

Table S7: Screening of reaction parameters – reaction temperature.

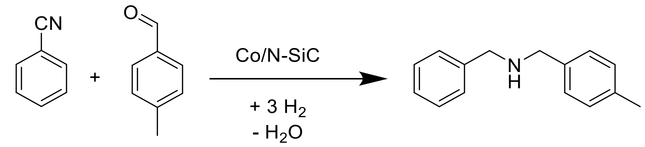

Entry	Temperature [°C]	Yield [%]
1	70	12
2	80	65
3	90	87
4	100	84
5	110	88

Reaction conditions: 0.5 mmol benzonitrile, 1.0 mmol 4-methylbenzaldehyde, 37 mg catalyst (5 mol% Co), 1.5 MPa H_2, 2 mL 2-methyltetrahydrofuran, 16 h; yields were determined by GC using *n*-dodecane as an internal standard.

Table S8: Screening of reaction parameters – H_2-pressure.

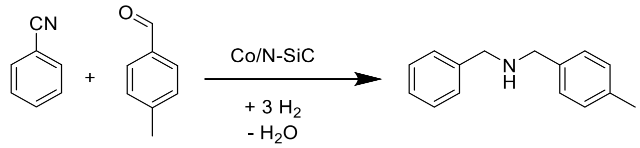

Entry	H_2-pressure [MPa]	Yield [%]
1	1.0	76
2	1.5	90
3	3.0	85
4	4.5	86

Reaction conditions: 0.5 mmol benzonitrile, 1.0 mmol 4-methylbenzaldehyde, 37 mg catalyst (5 mol% Co), 90 °C, 2 mL 2-methyltetrahydrofuran, 16 h; yields were determined by GC using *n*-dodecane as an internal standard.

Table S9: Screening of reaction parameters – amount of 4-methylbenzaldehyde.

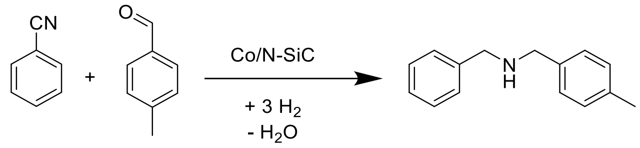

Entry	4-methylbenzaldehyde [mmol]	Yield [%]
1	0.5	71
2	1.0	90
3	1.5	69
4	2.0	60
5	3.0	10

Reaction conditions: 0.5 mmol benzonitrile, 37 mg catalyst (5 mol% Co), 90 °C, 1.5 MPa H_2, 3 mL 2-methyl-tetrahydrofuran, 16 h; Yields were determined by GC using *n*-dodecane as an internal standard.

Table S10: Screening of reaction parameters – mass ratio AN/SMP-10.

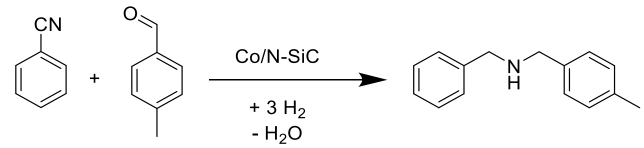

Entry	Mass ratio AN/SMP-10	Yield [%]	Surface area
1	100/0	16	16
2	80/20	90	527
3	50/50	79	362
4	20/80	1	13
5	0/100	2	13

Reaction conditions: 0.5 mmol benzonitrile, 1.0 mmol 4-methylbenzaldehyde, 37 mg catalyst (5 mol% Co), 90 °C, 1.5 MPa H_2, 3 mL 2-methyltetrahydrofuran, 16 h; yields were determined by GC using *n*-dodecane as an internal standard.

Table S11: Screening of reaction parameters – metal source.

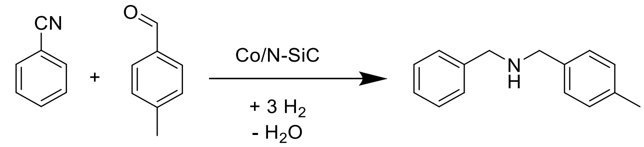

Entry	Metal source	Yield [%]
1	$Co(OAc)_2 \cdot 6\ H_2O$	71
2	$Co(acac)_2$	70
3	$Co(NO_3)_2 \cdot 6\ H_2O$	87
4	$Co(Cl)_2 \cdot 6\ H_2O$	5
5	Co(II) stearate	3
6	$[Co(NH_3)_6]Cl_3$	2

Reaction conditions: 0.5 mmol benzonitrile, 37 mg catalyst (5 mol% Co), 90 °C, 1.5 MPa H_2, 2 mL 2-methyl-tetrahydrofuran, 16 h; yields were determined by GC using *n*-dodecane as an internal standard.

Table S12: Screening of reaction parameters – pyrolysis temperature of the catalyst.

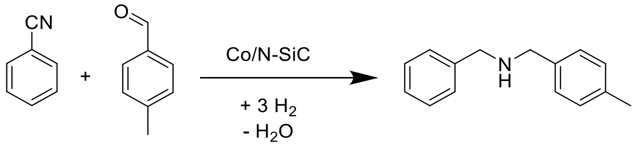

Entry	Pyrolysis temperature [°C]	Yield [%]
1	500	37
2	600	48
3	700	90
4	800	60
5	900	5

Reaction conditions: 0.5 mmol benzonitrile, 1.0 mmol 4-methylbenzaldehyde, 37 mg catalyst (5 mol% Co), 90 °C, 1.5 MPa H_2, 3 mL 2-methyltetrahydrofuran, 16 h; yields were determined by GC using *n*-dodecane as an internal standard.

Table S13: Screening of reaction parameters – support material.

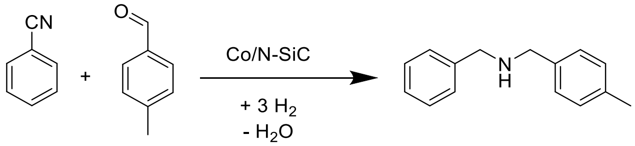

Entry	Support material	Yield [%]	Surface area [m^2/g]
1	N-SiC	90	527
2	Pyrolyzed polyacrylonitrile	0	5
3	Activated charcoal	0	893
4	γ-Al_2O_3	0	220
5	SiO_2	12	415

Reaction conditions: 0.5 mmol benzonitrile, 1.0 mmol 4-methylbenzaldehyde, 22 mg catalyst (3 mol% Co), 90 °C, 1.5 MPa H_2, 3 mL 2-methyltetrahydrofuran, 16 h; yields were determined by GC using *n*-dodecane as an internal standard.

Reductive alkylation of nitriles with aldehydes – general procedure

A 10 mL reaction vial was charged with a magnetic stirring bar, 1.0 mmol nitrile, 3.0 mmol aldehyde, 6 ml 2-methyltetrahydrofuran and 74 mg Co/N-SiC (5.0 mol% Co). The vial was placed in a 300 mL high-pressure autoclave (Parr Instruments) and the autoclave was flushed three times with 1.5 MPa hydrogen. Afterwards, 1.5 MPa hydrogen was applied and the reaction was stirred at 100 °C for 20 h. After completion of the reaction time, the autoclave was cooled to room temperature and the hydrogen was released. The catalyst was removed by centrifugation and the organic phase was separated. The secondary amine was converted to the corresponding hydrochloride salt by adding 1.2 mmol of HCl in ether. The solvent was removed and the residue washed twice with acetone. After purification the solid was analysed by ^{1}H and ^{13}C NMR spectroscopy. Elemental analysis was carried out for products with incomplete spectroscopic literature data.

Reductive alkylation of nitriles with ketones – general procedure

A 10 mL reaction vial was charged with a magnetic stirring bar, 1.0 mmol nitrile, 3.0 mmol aldehyde, 6 ml 2-methyltetrahydrofuran and 118 mg Co/N-SiC (8.0 mol% Co). The vial was placed in a 300 mL high-pressure autoclave (Parr Instruments) and the autoclave was flushed three times with 1.5 MPa hydrogen. Afterwards, 1.5 MPa hydrogen was applied and the reaction was stirred at the desired temperature for 20 h. After completion of the reaction time, the autoclave was cooled to room temperature and the hydrogen was released. The catalyst was removed by centrifugation and the organic phase was separated. The secondary amine was converted to the corresponding hydrochloride salt by adding 1.2 mmol of HCl in ether. The solvent was removed and the residue washed twice with acetone. After purification the solid was analysed by ^{1}H and ^{13}C NMR spectroscopy. Elemental analysis was carried out for products with incomplete spectroscopic literature data.

Evaluation of the catalyst stability

<u>Investigation of the used catalyst</u>

The Co/N-SiC catalyst was investigated with TEM measurements after the catalysis. The general procedure for the reductive alkylation of nitriles with aldehydes was applied. A 10 mL reaction vial was charged with a magnetic stirring bar, 1.0 mmol nitrile, 3.0 mmol aldehyde, 6 ml 2-methyltetrahydrofuran and 74 mg Co/N-SiC (5.0 mol% Co). The vial was placed in a 300 mL high-pressure autoclave (Parr Instruments) and the autoclave was flushed three times with 1.5 MPa hydrogen. Afterwards, 1.5 MPa hydrogen was applied and the reaction was stirred at 100 °C for 20 h. After completion of the reaction time, the autoclave was cooled to room temperature and the hydrogen was released. The catalyst was removed by centrifugation and the organic phase was separated. Afterwards, the catalyst was washed three times with 2-methyltetrahydrofuran and dried under vacuum. The catalyst was characterized with TEM measruements (Figure S9).

catalyst before catalysis

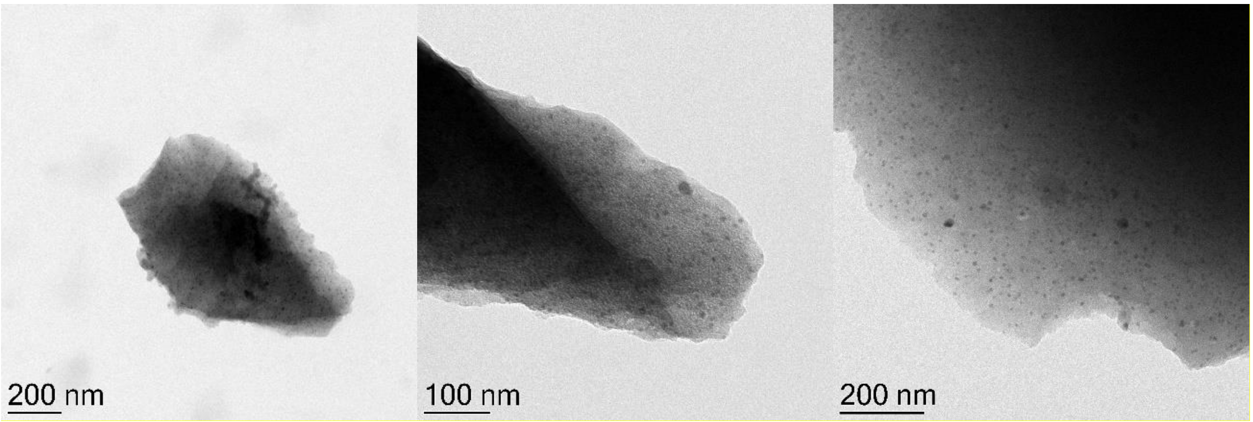

catalyst after catalysis

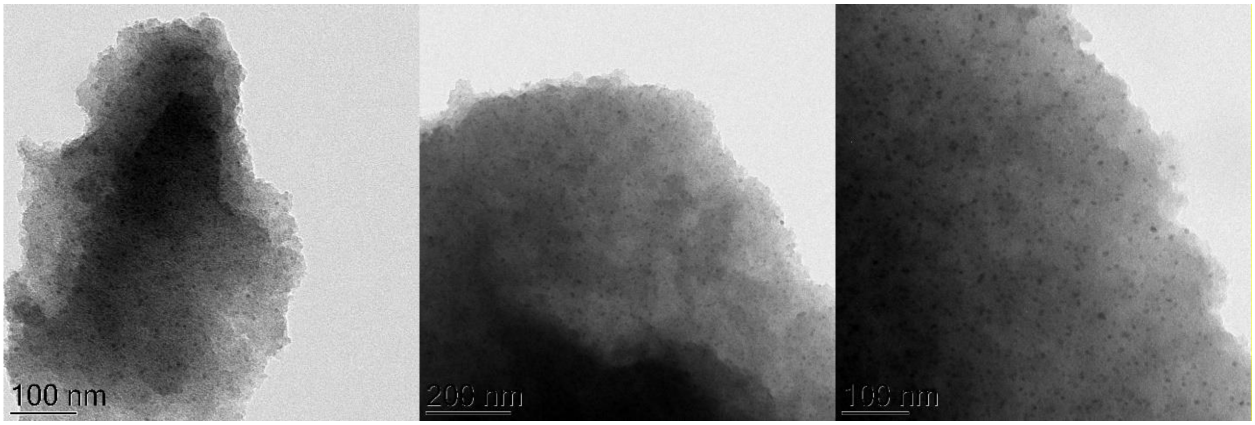

Figure S9: TEM characerization of the Co/N-SiC catalyst before and after the catalysis. No agglomerates or growth of the cobalt nanoparticles (~ 6 nm) were observed.

Hot filtration test

To demonstrate the catalyst stability a hot filtration test was performed. Therefore, a 10 mL reaction vial was charged with a magnetic stirring bar, 0.5 mmol benzonitrile, 1.0 mmol 4-methylbenzaldehyde, 3 ml 2-methyltetrahydrofuran and 37 mg Co/N-SiC (5.0 mol% Co). The vial was placed in a 300 mL high-pressure autoclave (Parr Instruments) and the autoclave was flushed three times with 1.5 MPa hydrogen. Afterwards, the 1.5 MPa hydrogen was applied and the reaction was stirred at the desired 90 °C. The hot mixture was filtered to remove the solid catalyst from the solution. 36 % benzonitrile was converted and 12 % secondary amine were generated after a reaction time of 3 h. 0.5 mmol benzonitrile und 1.0 mmol 4-methylbenzaldehyde were added to the solution and the solution was stirred for 16 h at the catalytic conditions. The filtrate did not show any activity. No educts or intermediate products were converted to the secondary amine.

Leaching test

A leaching test was performed to demonstrate, that our catalyst does not form homogeneous cobalt species. A 10 mL reaction vial was charged with a magnetic stirring bar, 1.0 mmol nitrile, 3.0 mmol aldehyde, 6 ml 2-methyltetrahydrofuran and 74 mg Co/N-SiC (5.0 mol% Co). The vial was placed in a 300 mL high-pressure autoclave (Parr Instruments) and the autoclave was flushed three times with 1.5 MPa hydrogen. Afterwards, 1.5 MPa hydrogen was applied and the reaction was stirred at 100 °C for 20 h. After completion of the reaction time, the autoclave was cooled to room temperature and the hydrogen was released. The reaction mixture was extracted with 5 mL H_2O and 10 mL 4.5 % nitric acid solution was added. The leaching amount of the Co/N-SiC catalyst was determined via ICP-AES. The leaching rate of cobalt is 0.7 %.

Catalyst recycling

The reductive alkylation of benzonitrile with 4-methylbenzaldehyde was chosen to investigate the recyclability of the novel cobalt nanocomposite catalyst. A 5 mL reaction vial was charged with a magnetic stirring bar, 0.5 mmol benzonitrile, 1.0 mmol 4-methylbenzaldehyde, 3 mL 2-methyltetrahydrofuran and 74 mg Co catalyst (10 mol%). The vial was placed in a high-pressure autoclave (Parr Instruments) and the autoclave was flushed five times with 2.0 MPa hydrogen. The autoclave was pressured with 1.5 MPa of hydrogen and the reaction was stirred for 6 h at 100 °C. After 6 h the autoclave was cooled to room temperature and the hydrogen pressure was released. The autoclave was opened in the glove box under N_2-atmosphere and there the catalyst was washed three times with 2-methyltetrahydrofuran. To accelerate the separation between solid and liquid a magnet was used. Therefore, the catalyst could be completely recovered. The yield of the secondary amine was determined by GC using *n*-dodecane as an internal standard (Figure S9).

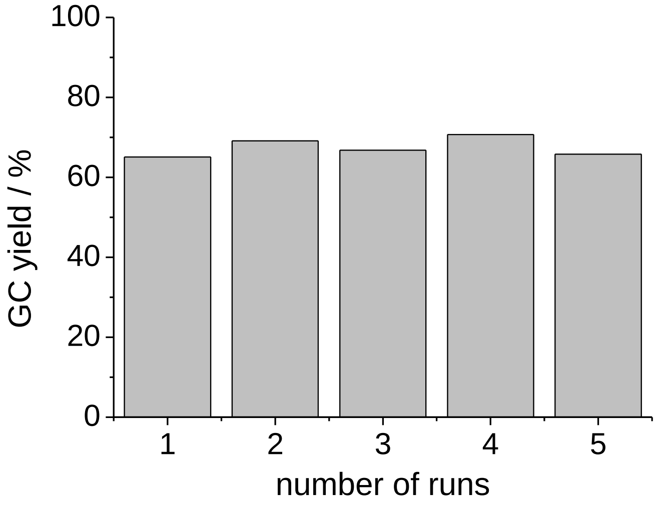

Figure S10: Results of the recycling experiment. No decrease of catalytic activity in five consecutive runs.

Up-scaling

The reaction were scaled up to 5 mmol substrate. The reaction was performed in a 50 mL glass vial equipped with a magnetic stirring bar. This vial was placed in a 300 mL high-pressure autoclave (Parr Instruments) and the autoclave was flushes three times with 1.5 MPa hydrogen. After pressurizing the autoclave with the desired pressure of 1.5 MPa hydrogen, the reaction was stirred for 20 h at 100 °C. The autoclave was cooled to room temperature and the hydrogen was released. The workup was identical to the 1.0 mmol reactions.

Table S14: Up-scaling of the reaction.

Entry	Product	Isolated yield of the hydrochloride salt [g]	Yield [%]
1	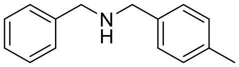	1.226	99
2	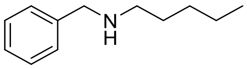	0.684	65
3	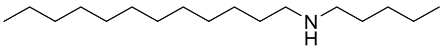	0.890	61
4	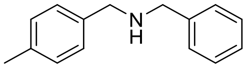	1.224	99
5	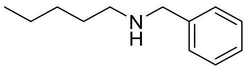	1.058.	99
6	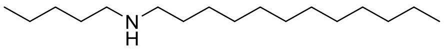	0.942	60

Reaction conditions: 5.0 mol% Co (370 mg Co/N-SiC, 4.0 wt% Co, 0.25 mmol Co, 14.7 mg Co), 5.0 mmol nitrile, 15.0 mmol aldehyde, 30 mL 2-methytetrahydrofuran, 100 °C, 1.5 MPa H_2, 20 h. Isolated yields are given for the corresponding hydrochloride salts.

4. Additional literature

Hydrogenation of amides

The selective hydrogenation of amides can be used for the synthesis of secondary alkyl amines. Recently a review has been published about the hydrogenation of amides.[12] This reaction is often catalyzed by homogeneous, rarely by heterogeneous catalysts (Table S18). Homogeneous catalysts with Ir[13], Ru[14, 15, 16, 17,18], Fe[19, 20, 21] and Mn[22] were used for the catalysis. Heterogeneous catalysts use only precious metals like Pd[23], Pt[24] and Ag[25]. All these catalysts are generally limited in the selective synthesis of aryl-alkyl amines.

Table S15: Synthesis of secondary alkyl amines via hydrogenation of amides.

**Entry**	**Metal**	**Catalysis**	**Secondary alkyl amines/products**	**Reference**
1	Ir	homogeneous	2/25	Yuan et al.[13]
2	Ru	homogeneous	5/23	Shi et al.[14]
3	Ru	homogeneous	1/5	John et al.[15]
4	Ru	homogeneous	3/24	Coetzee et al.[16]
5	Ru	homogeneous	1/20	Cabrero-Antonio et al.[17]
6	Ru	homogeneous	3/15	Kar et al.[18]
7	Fe	homogeneous	1/9	Schneck et al.[19]
8	Fe	homogeneous	1/11	Rezayee et al.[20]
9	Mn	homogeneous	1/25	Papa et al.[21]
10	Mn	homogeneous	5/15	Zou et al.[22]
11	Pd, Re	heterogeneous	4/18	Stein et al.[23]
12	Pt, V	heterogeneous	3/24	Mitsudome et al.[24]
13	Ag	heterogeneous	8/14	Xie et al.[25]

5. Characterization of isolated products

_N_-benzyl-1-(_p_-tolyl)methanaminium chloride (1)

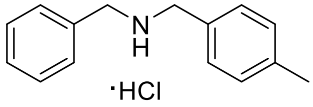

FW ($C_{15}H_{18}ClN$) = 247.77 g mol^{-1}
^{1}H-NMR (300 MHz, DMSO-d$_6$, 298 K): δ = 9.89 (s, 2H), 7.57 (dd, *J* = 6.4, 3.0 Hz, 2H), 7.51 – 7.36 (m, 5H), 7.22 (d, *J* = 7.9 Hz, 2H), 4.05 (dt, *J* = 9.1, 6.2 Hz, 4H), 2.31 (s, 3H) ppm.
^{13}C-NMR (75 MHz, DMSO-d$_6$, 298 K): δ = 138.26, 131.93, 130.16, 130.14, 129.29, 128.82, 128.80, 128.55, 49.45, 49.36, 20.81 ppm.
Yield: 99 % (0.99 mmol, 245 mg) as a white solid.

_N_-benzyl-1-(_m_-tolyl)methanaminium chloride (2)

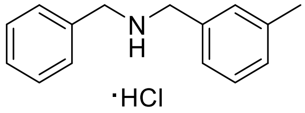

FW ($C_{15}H_{18}ClN$) = 247.77 g mol^{-1}
^{1}H-NMR (300 MHz, DMSO-d$_6$, 298 K): δ = 9.94 (s, 2H), 7.56 (dd, *J* = 14.7, 5.4 Hz, 2H), 7.40 (s, 5H), 7.34 – 7.18 (m, 2H), 4.21 – 3.91 (m, 4H), 2.31 (s, 3H) ppm.
^{13}C-NMR (75 MHz, DMSO-d$_6$, 298 K): δ = 137.71, 131.89, 131.77, 130.71, 130.17, 129.41, 128.82, 128.54, 128.47, 127.16, 49.61, 20.93 ppm.
Yield: 99 % (0.99 mmol, 244 mg) as a white solid.

_N_-benzyl-1-(_o_-tolyl)methanaminium chloride (3)

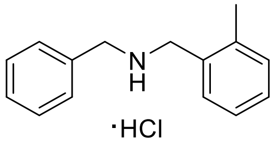

FW ($C_{15}H_{18}ClN$) = 247.77 g mol^{-1}
^{1}H-NMR (300 MHz, DMSO-d$_6$, 298 K): δ = 9.79 (s, 2H), 7.68 – 7.50 (m, 3H), 7.43 (s, 2H), 7.25 (d, *J* = 6.3 Hz, 4H), 4.13 (m, 4H), 2.28 (d, *J* = 14.4 Hz, 3H) ppm.
^{13}C-NMR (75 MHz, DMSO-d$_6$, 298 K): δ = 137.40, 131.80, 130.60, 130.43, 130.32, 130.16, 128.93, 128.59, 126.02, 49.97, 46.46, 18.93 ppm.
Yield: 89 % (0.89 mmol, 246 mg) as a white solid.

N-benzyl-1-(4-fluorophenyl)methanaminium chloride (4)

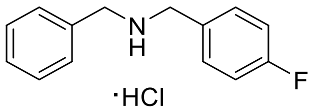

FW ($C_{14}H_{15}ClFN$) = 251.73 g mol^{-1}

^{1}H-NMR (300 MHz, DMSO-d$_6$, 298 K): δ = 10.02 (s, 2H), 7.79 – 7.51 (m, 4H), 7.51 – 7.32 (m, 3H), 7.24 (t, *J* = 8.8 Hz, 2H), 4.11 (s, 4H) ppm.

^{13}C-NMR (75 MHz, DMSO-d$_6$, 298 K): δ = 163.95, 160.70, 132.74, 132.63, 131.93, 130.20, 128.81, 128.53, 128.23, 128.18, 115.49, 115.20, 49.56, 48.86 ppm.

Yield: 91 % (0.91 mmol, 229 mg) as a white solid.

N-benzyl-1-(4-chlorophenyl)methanaminium chloride (5)

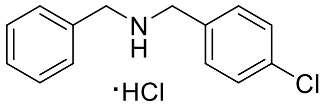

FW ($C_{14}H_{15}Cl_2N$) = 268.18 g mol^{-1}

^{1}H-NMR (300 MHz, DMSO-d$_6$, 298 K): δ = 9.99 (s, 2H), 7.60 (dd, *J* = 12.9, 6.1 Hz, 4H), 7.48 (d, *J* = 8.1 Hz, 2H), 7.41 (d, *J* = 3.3 Hz, 2H), 4.12 (s, 4H) ppm.

^{13}C-NMR (75 MHz, DMSO-d$_6$, 298 K): δ = 133.61, 132.22, 131.88, 130.91, 130.18, 128.84, 128.54, 128.49, 49.65, 48.88 ppm.

Yield: 99 % (0.99 mmol, 266 mg) as a white solid.

N-benzyl-1-(3-chlorophenyl)methanaminium chloride (6)

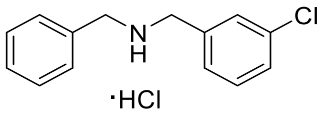

FW ($C_{14}H_{15}Cl_2N$) = 268.18 g mol^{-1}

^{1}H-NMR (300 MHz, DMSO-d$_6$, 298 K): δ = 9.97 (s, 2H), 7.73 (s, 1H), 7.56 (d, *J* = 6.7 Hz, 3H), 7.50 – 7.29 (m, 5H), 4.15 (s, 4H) ppm.

^{13}C-NMR (75 MHz, DMSO-d$_6$, 298 K): δ = 134.34, 133.00, 131.85, 130.37, 130.18, 130.09, 128.95, 128.86, 128.77, 128.54, 49.86, 49.05 ppm.

Yield: 99 % (0.99 mmol, 264 mg) as a white solid.

N-benzyl-1-(2-chlorophenyl)methanaminium chloride (7)

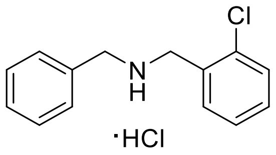

FW ($C_{14}H_{15}Cl_2N$) = 268.18 g mol^{-1}
^{1}H-NMR (300 MHz, DMSO-d$_6$, 298 K): δ = 10.04 (s, 2H), 7.89 – 7.75 (m, 1H), 7.60 (dd, *J* = 8.2, 6.8 Hz, 2H), 7.43 (d, *J* = 2.9 Hz, 6H), 4.22 (s, 4H) ppm.
^{13}C-NMR (75 MHz, DMSO-d$_6$, 298 K): δ = 133.51, 131.97, 131.88, 130.74, 130.32, 130.16, 129.82, 129.49, 128.96, 128.84, 128.57, 127.44, 50.01, 46.20 ppm.
Yield: 93 % (0.93 mmol, 266 mg) as a white solid.

_N_-benzyl-1-(4-bromophenyl)methanaminium chloride (8)

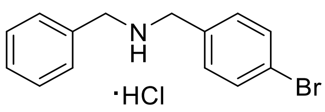

FW ($C_{14}H_{15}ClBrN$) = 312.64 g mol^{-1}
^{1}H-NMR (300 MHz, DMSO-d$_6$, 298 K): δ = 8.55 (s, 2H), 7.84 (d, *J* = 1.1 Hz, 1H), 7.69 – 7.26 (m, 8H), 4.11 (t, *J* = 5.6 Hz, 2H), 4.00 (dd, *J* = 10.7, 4.9 Hz, 2H) ppm.
^{13}C-NMR (75 MHz, DMSO-d$_6$, 298 K): δ = 134.10, 132.50, 131.42, 131.28, 130.17, 128.94, 128.54, 128.37, 122.28, 121.68, 49.66, 48.96 ppm.
Yield: 86 % (0.86 mmol, 269 mg) as a green solid.

_N_-benzyl-1-(4-(tert-butyl)phenyl)methanaminium chloride (9)

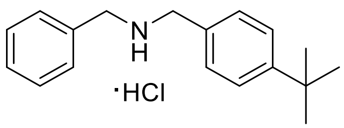

FW ($C_{18}H_{24}ClN$) = 289.85 g mol^{-1}
^{1}H-NMR (400 MHz, DMSO-d$_6$, 298 K): δ = 9.91 (s, 2H), 7.64 – 7.56 (m, 2H), 7.55 – 7.38 (m, 6H), 7.28 (dd, *J* = 38.9, 8.0 Hz, 1H), 4.09 (d, *J* = 21.3 Hz, 4H), 1.28 (s, 9H).
^{13}C-NMR (100 MHz, DMSO-d$_6$, 298 K): δ = 151.35, 131.93, 130.16, 129.96, 128.97, 128.79, 128.53, 126.29, 125.30, 124.70, 49.62, 49.28, 39.52, 34.38, 31.04.
Yield: 99 % (0.99 mmol, 287 mg) as a white solid.

_N_-benzyl-1-(4-(4,4,5,5-tetramethyl-1,3,2-dioxaborolan-2-yl)phenyl)methanaminium chloride (10)

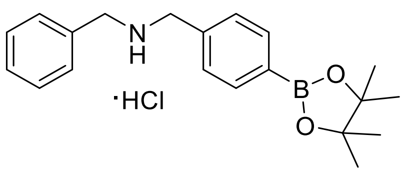

FW ($C_{20}H_{27}BClNO_2$) = 359.70 g mol^{-1}
^{1}H-NMR (300 MHz, DMSO-d$_6$, 298 K): δ = 8.68 (s, 2H), 7.68 (dd, *J* = 7.8, 3.6 Hz, 1H), 7.64 – 7.56 (m, 1H), 7.56 – 7.47 (m, 2H), 7.47 – 7.28 (m, 5H), 4.03 (dt, *J* = 17.2, 5.6 Hz, 4H), 1.30 (d, *J* = 6.1 Hz, 12H).
^{13}C-NMR (75 MHz, DMSO-d$_6$, 298 K): δ = 137.36, 134.49, 134.15, 131.91, 131.87, 130.23, 129.63, 128.99, 128.80, 128.52, 128.50, 128.33, 83.81, 83.76, 49.55, 42.08, 39.52, 24.69.
Yield: 83 % (0.83 mmol, 299 mg) as a white solid.

_N_-benzyl-1-(4-methoxyphenyl)methanaminium chloride (11)

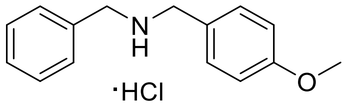

FW ($C_{15}H_{18}ClNO$) = 263.77 g mol^{-1}
^{1}H-NMR (300 MHz, DMSO-d$_6$, 298 K): δ = 9.81 (s, 2H), 7.65 – 7.32 (m, 7H), 6.97 (d, *J* = 8.3 Hz, 2H), 4.20 – 3.92 (m, 4H), 3.76 (s, 3H) ppm.
^{13}C-NMR (75 MHz, DMSO-d$_6$, 298 K): δ = 159.63, 131.98, 131.77, 130.11, 128.80, 128.55, 123.64, 113.90, 55.19, 49.35, 49.15 ppm.
Yield: 99 % (0.99 mmol, 261 mg) as a white solid.

_N_-benzyl-1-(4-(benzyloxy)phenyl)methanaminium chloride (12)

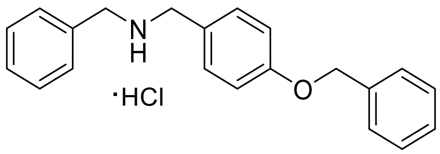

FW ($C_{21}H_{22}ClNO$) = 339.87 g mol^{-1}
^{1}H-NMR (300 MHz, DMSO-d$_6$, 298 K): δ = 9.72 (s, 2H), 7.68 – 7.18 (m, 12H), 7.05 (dd, *J* = 8.7, 2.0 Hz, 2H), 5.13 (s, 2H), 4.07 (q, *J* = 10.8 Hz, 4H) ppm.
^{13}C-NMR (75 MHz, DMSO-d$_6$, 298 K): δ = 158.68, 136.89, 131.97, 130.09, 128.83, 128.57, 128.45, 127.87, 127.67, 123.89, 114.80, 69.17, 49.45, 49.21 ppm.
Yield: 99 % (0.99 mmol, 336 mg) as a white solid.

1-(benzo[d][1,3]dioxol-5-yl)-_N_-benzylmethanaminium chloride (13)

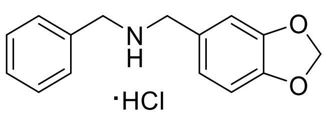

FW ($C_{15}H_{16}ClNO_2$) = 277.75 g mol^{-1}
^{1}H-NMR (300 MHz, DMSO-d$_6$, 298 K): δ = 9.92 (s, 2H), 7.58 (dd, *J* = 6.5, 3.0 Hz, 2H), 7.51 – 7.17 (m, 4H), 7.08 – 6.86 (m, 2H), 6.04 (d, *J* = 1.1 Hz, 2H), 4.19 – 3.82 (m, 4H).
^{13}C-NMR (75 MHz, DMSO-d$_6$, 298 K): δ = 147.63, 147.24, 131.99, 130.14, 128.77, 128.51, 125.37, 125.29, 124.31, 124.23, 110.50, 108.19, 101.27, 49.45, 49.28, 39.52.
Yield: 97 % (0.97 mmol, 269 mg) as a white solid.

***N*-benzylpentan-1-aminium chloride (14)**

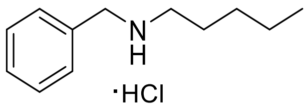

FW ($C_{12}H_{20}ClN$) = 213.75 g mol^{-1}
^{1}H-NMR (300 MHz, DMSO-d$_6$, 298 K): δ = 9.43 (s, 2H), 7.67 – 7.50 (m, 2H), 7.50 – 7.29 (m, 3H), 4.09 (t, *J* = 5.6 Hz, 2H), 2.82 (d, *J* = 4.5 Hz, 2H), 1.80 – 1.49 (m, 2H), 1.27 (td, *J* = 7.0, 3.9 Hz, 4H), 0.86 (td, *J* = 6.6, 3.3 Hz, 3H) ppm.
^{13}C-NMR (75 MHz, DMSO-d$_6$, 298 K): δ = 132.12, 131.88, 130.15, 130.06, 128.78, 128.55, 49.73, 46.27, 28.13, 24.84, 21.60, 13.70 ppm.
Yield: 64 % (0.64 mmol, 137 mg) as a white solid.

***N*-benzylheptan-1-aminium chloride (15)**

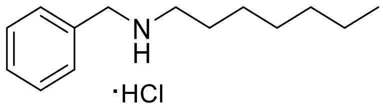

FW ($C_{14}H_{24}ClN$) = 241.81 g mol^{-1}
^{1}H-NMR (300 MHz, DMSO-d$_6$, 298 K): δ = 9.49 (s, 2H), 7.59 (dt, *J* = 4.1, 2.3 Hz, 2H), 7.49 – 7.33 (m, 3H), 4.10 (d, *J* = 5.0 Hz, 2H), 2.81 (d, *J* = 4.3 Hz, 2H), 1.68 (d, *J* = 6.9 Hz, 2H), 1.24 (s, 8H), 0.85 (t, *J* = 6.7 Hz, 3H) ppm.
^{13}C-NMR (75 MHz, DMSO-d$_6$, 298 K): δ = 132.12, 131.89, 130.18, 130.08, 128.76, 128.54, 49.72, 46.29, 31.00, 28.17, 25.99, 25.17, 21.97, 13.92 ppm.
Yield: 60 % (0.60 mmol, 145 mg) as a grey solid.

***N*-benzyldecan-1-aminium chloride (16)**

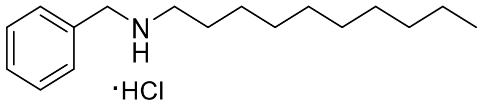

FW ($C_{17}H_{30}ClN$) = 283.89 g mol^{-1}
^{1}H-NMR (300 MHz, DMSO-d$_6$, 298 K): δ = 9.35 (s, 1H), 7.62 – 7.51 (m, 2H), 7.45 – 7.36 (m, 3H), 4.09 (t, *J* = 5.8 Hz, 2H), 2.82 (q, *J* = 11.8 Hz, 2H), 1.71 – 1.52 (m, 2H), 1.24 (s, 14H), 0.85 (t, *J* = 6.6 Hz, 3H) ppm.

^{13}**C-NMR** (75 MHz, DMSO-d$_6$, 298 K): δ = 132.12, 130.15, 130.05, 128.81, 128.58, 49.76, 46.34, 31.30, 28.99, 28.94, 28.79, 28.72, 25.20, 22.11, 13.98 ppm.
Yield: 62 % (0.62 mmol, 176 mg) as a white solid.

_N_-benzyldodecan-1-aminium chloride (17)

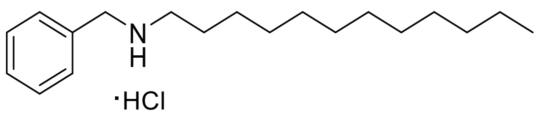

FW ($C_{19}H_{34}ClN$) = 311.94 g mol^{-1}
^{1}**H-NMR** (300 MHz, DMSO-d$_6$, 298 K): δ = 9.31 (s, 2H), 7.56 (s, 2H), 7.41 (s, 3H), 4.10 (s, 2H), 2.82 (s, 2H), 1.64 (s, 2H), 1.24 (s, 16H), 0.85 (s, 3H) ppm.
^{13}**C-NMR** (75 MHz, DMSO-d$_6$, 298 K): δ = 132.11, 130.14, 130.03, 128.82, 128.59, 49.78, 46.36, 31.30, 29.03, 28.93, 28.79, 28.72, 28.50, 22.11, 13.97 ppm.
Yield: 67 % (0.67 mmol, 209 mg) as a white solid.

_N_-benzyl-3,7-dimethyloct-6-en-1-aminium chloride (18)

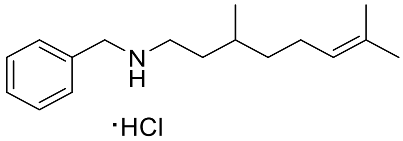

FW ($C_{17}H_{28}ClN$) = 281.87 g mol^{-1}
^{1}**H-NMR** (300 MHz, DMSO-d$_6$, 298 K): δ = 9.36 (s, 2H), 7.58 (dd, *J* = 6.7, 2.0 Hz, 2H), 7.50 – 7.31 (m, 3H), 5.07 (dd, *J* = 7.1, 6.0 Hz, 1H), 4.05 (d, *J* = 30.0 Hz, 2H), 2.87 (d, *J* = 7.9 Hz, 2H), 1.93 (d, *J* = 5.6 Hz, 2H), 1.79 – 1.38 (m, 9H), 1.35 – 0.99 (m, 2H), 0.84 (d, *J* = 5.9 Hz, 3H).
^{13}**C-NMR** (75 MHz, DMSO-d$_6$, 298 K): δ = 132.16, 130.73, 130.15, 130.05, 128.81, 128.58, 124.36, 49.79, 44.70, 39.52, 36.24, 31.92, 29.75, 25.52, 24.78, 19.13, 17.57.
Yield: 65 % (0.65 mmol, 183 mg) as a white solid.

_N_-benzyl-1-cyclopropylmethanaminium chloride (19)

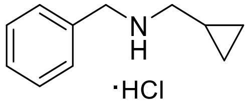

FW ($C_{11}H_{16}ClN$) = 197.71 g mol^{-1}
^{1}**H-NMR** (300 MHz, DMSO-d$_6$, 298 K): δ = 9.42 (s, 2H), 7.63 – 7.51 (m, 2H), 7.50 – 7.35 (m, 3H), 4.12 (s, 2H), 2.78 (d, J = 7.2 Hz, 2H), 1.21 – 1.03 (m, 1H), 0.60 – 0.51 (m, 2H), 0.39 – 0.32 (m, 2H) ppm.
^{13}**C-NMR** (75 MHz, DMSO-d$_6$, 298 K): δ = 132.18, 130.14, 129.99, 128.80, 128.58, 50.94, 49.45, 6.92, 4.08,

3.98 ppm.
Yield: 70 % (0.70 mmol, 138 mg) as a white solid.

N-benzyl-1-(*p*-tolyl)methanaminium chloride (20)

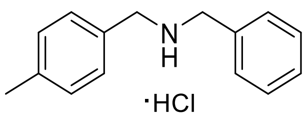

FW ($C_{15}H_{18}ClN$) = 247.77 g mol^{-1}
^{1}H-NMR (300 MHz, DMSO-d$_6$, 298 K): δ = 9.86 (s, 2H), 7.67 – 7.52 (m, 2H), 7.52 – 7.36 (m, 5H), 7.22 (d, *J* = 7.8 Hz, 2H), 4.09 (dt, *J* = 18.2, 6.0 Hz, 4H), 2.32 (s, 3H) ppm.
^{13}C-NMR (75 MHz, DMSO-d$_6$, 298 K): δ = 138.26, 131.92, 130.15, 129.09, 128.83, 128.78, 128.55, 49.47, 49.38, 39.52, 20.80 ppm.
Yield: 99 % (0.99 mmol, 245 mg) as a white solid.

N-benzyl-1-(*m*-tolyl)methanaminium chloride (21)

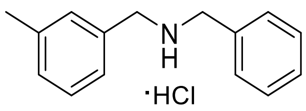

FW ($C_{15}H_{18}ClN$) = 247.77 g mol^{-1}
^{1}H-NMR (300 MHz, DMSO-d$_6$, 298 K): δ = 9.89 (s, 2H), 7.58 (dd, *J* = 4.8, 2.3 Hz, 2H), 7.46 – 7.34 (m, 5H), 7.30 (t, *J* = 7.7 Hz, 1H), 7.21 (d, *J* = 7.4 Hz, 1H), 4.18 – 3.97 (m, 4H), 2.31 (s, 3H) ppm.
^{13}C-NMR (75 MHz, DMSO-d$_6$, 298 K): δ = 137.71, 131.89, 131.77, 130.71, 130.17, 129.41, 128.82, 128.54, 128.47, 127.16, 49.61, 20.93 ppm.
Yield: 96 % (0.96 mmol, 238 mg) as a white solid.

N-benzyl-1-(*o*-tolyl)methanaminium chloride (22)

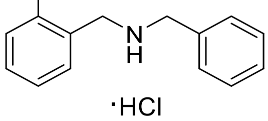

FW ($C_{15}H_{18}ClN$) = 247.77 g mol^{-1}
^{1}H-NMR (300 MHz, DMSO-d$_6$, 298 K): δ = 9.79 (s, 2H), 7.68 – 7.50 (m, 3H), 7.43 (s, 2H), 7.25 (d, *J* = 6.3 Hz, 4H), 4.13 (m, 4H), 2.28 (d, *J* = 14.4 Hz, 3H) ppm.
^{13}C-NMR (75 MHz, DMSO-d$_6$, 298 K): δ = 137.40, 131.80, 130.60, 130.43, 130.32, 130.16, 128.93, 128.59,

126.02, 49.97, 46.46, 18.93 ppm.
Yield: 90 % (0.90 mmol, 223 mg) as a white solid.

***N*-benzyl-1-(4-fluorophenyl)methanaminium chloride (23)**

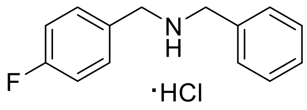

FW ($C_{14}H_{15}ClFN$) = 251.73 g mol^{-1}
^{1}H-NMR (300 MHz, DMSO-d$_6$, 298 K): δ = 9.96 (s, 2H), 7.75 – 7.51 (m, 4H), 7.40 (s, 3H), 7.25 (t, *J* = 8.8 Hz, 2H), 4.12 (s, 4H) ppm.
^{13}C-NMR (75 MHz, DMSO-d$_6$, 298 K): δ = 163.95, 160.70, 132.71, 132.59, 131.92, 130.16, 128.83, 128.54, 128.22, 128.18, 115.50, 115.22, 49.58, 48.88 ppm.
Yield: 99 % (0.99 mmol, 249 mg) as a white solid.

***N*-benzyl-1-(4-chlorophenyl)methanaminium chloride (24)**

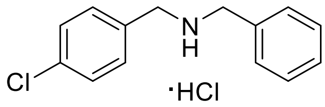

FW ($C_{14}H_{15}Cl_2N$) = 268.18 g mol^{-1}
^{1}H-NMR (300 MHz, DMSO-d$_6$, 298 K): δ = 9.97 (s, 2H), 7.68 – 7.54 (m, 4H), 7.49 (d, *J* = 8.4 Hz, 2H), 7.41 (dd, *J* = 5.0, 1.3 Hz, 3H), 4.12 (d, *J* = 4.3 Hz, 4H) ppm.
^{13}C-NMR (75 MHz, DMSO-d$_6$, 298 K): δ = 133.61, 132.22, 131.89, 130.91, 130.18, 128.85, 128.55, 128.50, 49.66, 48.90 ppm.
Yield: 99 % (0.99 mmol, 266 mg) as a white solid.

***N*-benzyl-1-(3-chlorophenyl)methanaminium chloride (25)**

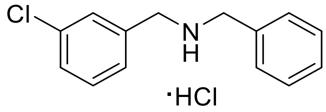

FW ($C_{14}H_{15}Cl_2N$) = 268.18 g mol^{-1}
^{1}H-NMR (300 MHz, DMSO-d$_6$, 298 K): δ = 10.02 (s, 2H), 7.73 (s, 1H), 7.56 (d, *J* = 7.6 Hz, 3H), 7.43 (d, *J* = 16.2 Hz, 5H), 4.14 (d, *J* = 4.2 Hz, 4H) ppm.
^{13}C-NMR (75 MHz, DMSO-d$_6$, 298 K): δ = 134.33, 132.99, 131.85, 130.35, 130.19, 130.10, 128.96, 128.84, 128.75, 128.52, 49.83, 49.01 ppm.
Yield: 99 % (0.99 mmol, 264 mg) as a white solid.

_N_-benzyl-1-(2-chlorophenyl)methanaminium chloride (26)

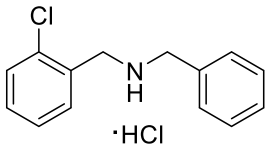

FW ($C_{14}H_{15}Cl_2N$) = 268.18 g mol^{-1}
^{1}H-NMR (300 MHz, DMSO-d$_6$, 298 K): δ = 10.04 (s, 2H), 7.89 – 7.75 (m, 1H), 7.60 (dd, *J* = 8.2, 6.8 Hz, 2H), 7.43 (d, *J* = 2.9 Hz, 6H), 4.22 (s, 4H) ppm.
^{13}C-NMR (75 MHz, DMSO-d$_6$, 298 K): δ = 133.51, 131.97, 131.88, 130.74, 130.32, 130.16, 129.82, 129.49, 128.96, 128.84, 128.57, 127.44, 50.01, 46.20 ppm.
Yield: 99 % (0.99 mmol, 264 mg) as a white solid.

_N_-benzyl-1-(4-bromophenyl)methanaminium chloride (27)

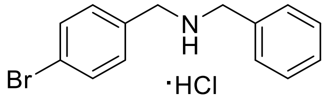

FW ($C_{14}H_{15}ClBrN$) = 312.64 g mol^{-1}
^{1}H-NMR (300 MHz, DMSO-d$_6$, 298 K): δ = 9.94 (s, 2H), 7.68 – 7.49 (m, 5H), 7.41 (d, *J* = 4.4 Hz, 4H), 4.12 (s, 4H) ppm.
^{13}C-NMR (75 MHz, DMSO-d$_6$, 298 K): δ = 132.49, 131.88, 131.44, 131.30, 130.16, 128.86, 128.56, 122.29, 49.68, 48.98 ppm.
Yield: 99 % (0.99 mmol, 310 mg) as a green solid.

_N_-benzyl-1-(4-methoxyphenyl)methanaminium chloride (28)

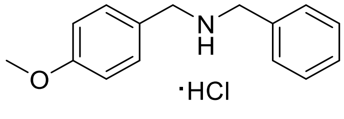

FW ($C_{15}H_{18}ClNO$) = 263.77 g mol^{-1}
^{1}H-NMR (300 MHz, DMSO-d$_6$, 298 K): δ = 9.79 (s, 2H), 7.57 (dt, *J* = 5.6, 2.8 Hz, 2H), 7.49 (dd, *J* = 8.5, 3.9 Hz, 2H), 7.45 – 7.36 (m, 3H), 6.97 (d, *J* = 8.3 Hz, 2H), 4.17 – 3.96 (m, 4H), 3.76 (s, 3H) ppm.
^{13}C-NMR (75 MHz, DMSO-d$_6$, 298 K): δ = 159.63, 131.98, 131.89, 131.76, 131.70, 130.17, 130.11, 128.85, 128.80, 128.55, 123.72, 123.63, 113.90, 55.19, 49.65, 49.36 ppm.
Yield: 99 % (0.99 mmol, 261 mg) as a white solid.

_N_-benzyl-1-(3-methoxyphenyl)methanaminium chloride (29)

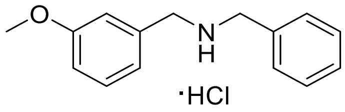

FW ($C_{15}H_{18}ClNO$) = 263.77 g mol^{-1}
^{1}H-NMR (300 MHz, DMSO-d$_6$, 298 K): δ = 9.91 (s, 2H), 7.58 (dd, *J* = 6.5, 3.0 Hz, 2H), 7.47 – 7.37 (m, 3H), 7.33 (t, *J* = 7.9 Hz, 1H), 7.26 (s, 1H), 7.11 (d, *J* = 7.6 Hz, 1H), 6.96 (dd, *J* = 8.3, 2.5 Hz, 1H), 4.10 (d, *J* = 4.0 Hz, 4H), 3.77 (s, 3H) ppm.
^{13}C-NMR (75 MHz, DMSO-d$_6$, 298 K): δ = 159.26, 133.28, 131.88, 130.16, 129.66, 128.85, 128.55, 122.12, 115.48, 114.56, 55.20, 49.62 ppm.
Yield: 90 % (0.90 mmol, 235 mg) as a white solid.

_N_-benzyl-1-(2,6-difluorophenyl)methanaminium chloride (30)

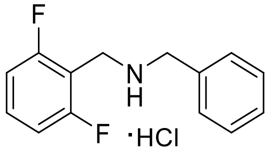

FW ($C_{14}H_{14}ClF_2N$) = 269.72 g mol^{-1}
^{1}H-NMR (300 MHz, DMSO-d$_6$, 298 K): δ = 9.96 (s, 2H), 7.66 – 7.51 (m, 3H), 7.46 – 7.37 (m, 3H), 7.19 (t, *J* = 8.1 Hz, 2H), 4.22 (s, 2H), 4.12 (s, 2H) ppm.
^{13}C-NMR (75 MHz, DMSO-d$_6$, 298 K): δ = 162.88, 159.57, 132.42, 131.57, 130.20, 128.96, 128.83, 128.58, 128.54, 111.97, 111.64, 108.22, 50.03, 36.73 ppm.
Yield: 84 % (0.84 mmol, 227 mg) as a white solid.

_N_-benzyl-1-(maphthalen-2-yl)methanaminium chloride (31)

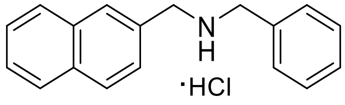

FW ($C_{18}H_{18}ClN$) = 283.80 g mol^{-1}
^{1}H-NMR (300 MHz, DMSO-d$_6$, 298 K): δ = 10.06 (s, 2H), 8.09 (d, *J* = 6.1 Hz, 1H), 8.01 – 7.87 (m, 3H), 7.82 – 7.73 (m, 1H), 7.59 (ddd, *J* = 9.4, 6.8, 3.5 Hz, 4H), 7.47 – 7.35 (m, 3H), 4.32 (dt, *J* = 15.5, 5.2 Hz, 2H), 4.14 (dt, *J* = 16.8, 5.5 Hz, 2H) ppm.
^{13}C-NMR (75 MHz, DMSO-d$_6$, 298 K): δ = 132.79, 132.50, 131.93, 131.89, 130.21, 130.17, 129.58, 129.46, 128.83, 128.54, 128.13, 127.86, 127.64, 127.40, 126.73, 126.57, 49.79, 49.71 ppm.
Yield: 84 % (0.84 mmol, 238 mg) as a white solid.

_N_-benzyl-1-(furan-2-yl)methanaminium chloride (32)

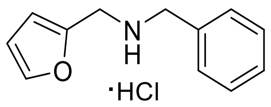

FW ($C_{12}H_{14}ClNO$) = 223.70 g mol^{-1}
^{1}H-NMR (300 MHz, DMSO-d$_6$, 298 K): δ = 9.91 (s, 2H), 7.75 (d, *J* = 9.9 Hz, 1H), 7.55 (d, *J* = 6.3 Hz, 2H), 7.41 (s, 3H), 6.66 (d, *J* = 5.2 Hz, 1H), 6.53 (s, 1H), 4.14 (d, *J* = 20.3 Hz, 4H) ppm.
^{13}C-NMR (75 MHz, DMSO-d$_6$, 298 K): δ = 145.73, 144.21, 144.11, 131.89, 131.71, 130.15, 128.91, 128.61, 112.31, 111.10, 49.36, 41.81 ppm.
Yield: 99 % (0.99 mmol, 221 mg) as a white solid.

***N*-(4-methylbenzyl)pentan-1-aminium chloride (33)**

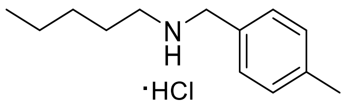

FW ($C_{13}H_{22}ClN$) = 227.78 g mol^{-1}
^{1}H-NMR (300 MHz, DMSO-d$_6$, 298 K): δ = 9.35 (s, 2H), 7.53 – 7.36 (m, 2H), 7.22 (d, *J* = 7.8 Hz, 2H), 4.04 (s, 2H), 2.79 (s, 2H), 2.32 (s, 3H), 1.64 (d, *J* = 5.7 Hz, 2H), 1.26 (s, 4H), 0.85 (d, *J* = 6.7 Hz, 3H).
^{13}C-NMR (75 MHz, DMSO-d$_6$, 298 K): δ = 138.21, 130.05, 129.10, 49.46, 46.10, 39.52, 28.15, 24.86, 21.63, 20.80, 13.73.
Yield: 94 % (0.94 mmol, 214 mg) as a white solid.

***N*-(3-methylbenzyl)pentan-1-aminium chloride (34)**

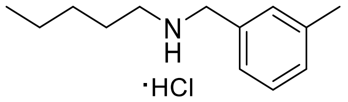

FW ($C_{13}H_{22}ClN$) = 227.78 g mol^{-1}
^{1}H-NMR (300 MHz, DMSO-d$_6$, 298 K): δ = 9.44 (s, 2H), 7.49 – 7.10 (m, 5H), 4.05 (s, 2H), 2.80 (s, 2H), 2.32 (s, 3H), 1.66 (d, *J* = 6.2 Hz, 2H), 1.27 (d, *J* = 6.8 Hz, 4H), 0.85 (d, *J* = 6.5 Hz, 3H).
^{13}C-NMR (75 MHz, DMSO-d$_6$, 298 K): δ = 137.72, 132.03, 130.62, 129.36, 128.48, 127.10, 49.72, 46.26, 39.52, 28.15, 24.85, 21.63, 20.94, 13.72.
Yield: 91 % (0.91 mmol, 207 mg) as a white solid.

***N*-(2-methylbenzyl)pentan-1-aminium chloride (35)**

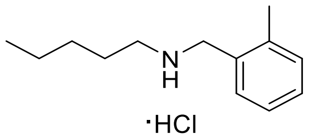

FW ($C_{13}H_{22}ClN$) = 227.78 g mol^{-1}
^{1}H-NMR (300 MHz, DMSO-d$_6$, 298 K): δ = 9.29 (s, 2H), 7.68 – 7.47 (m, 1H), 7.32 – 7.16 (m, 3H), 4.13 (dt, *J* = 11.9, 5.6 Hz, 2H), 3.04 – 2.81 (m, 2H), 2.35 (d, *J* = 24.2 Hz, 3H), 1.80 – 1.61 (m, 2H), 1.40 – 1.16 (m, 4H), 0.88 (dd, *J* = 9.2, 4.3 Hz, 3H).
^{13}C-NMR (75 MHz, DMSO-d$_6$, 298 K): δ = 137.35, 130.64, 130.46, 128.86, 126.01, 47.12, 46.96, 39.52, 28.21, 24.85, 21.66, 19.12, 13.76.
Yield: 82 % (0.82 mmol, 187 mg) as a white solid.

***N*-(4-fluorobenzyl)pentan-1-aminium chloride (36)**

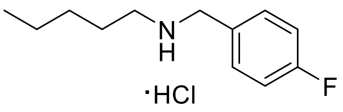

FW ($C_{12}H_{19}ClFN$) = 231.74 g mol^{-1}
^{1}H-NMR (300 MHz, DMSO-d$_6$, 298 K): δ = 9.42 (s, 2H), 7.65 (dd, *J* = 8.3, 5.7 Hz, 2H), 7.26 (t, *J* = 8.8 Hz, 2H), 4.10 (dd, *J* = 12.0, 6.2 Hz, 2H), 2.97 – 2.66 (m, 2H), 1.80 – 1.51 (m, 2H), 1.39 – 1.15 (m, 4H), 0.86 (t, *J* = 6.7 Hz, 3H).
^{13}C-NMR (75 MHz, DMSO-d$_6$, 298 K): δ = 163.93, 160.68, 132.56, 132.44, 128.45, 115.55, 115.26, 48.91, 46.21, 39.52, 28.14, 24.88, 21.62, 13.72.
Yield: 82 % (0.82 mmol, 190 mg) as a white solid.

***N*-(4-chlorobenzyl)pentan-1-aminium chloride (37)**

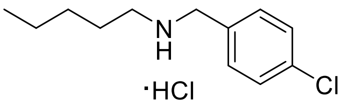

FW ($C_{12}H_{19}Cl_2N$) = 248.19 g mol^{-1}
^{1}H-NMR (300 MHz, DMSO-d$_6$, 298 K): δ = 9.48 (s, 2H), 7.62 (d, *J* = 6.9 Hz, 2H), 7.55 – 7.41 (m, 2H), 4.10 (s, 2H), 2.81 (s, 2H), 1.76 – 1.55 (m, 2H), 1.27 (dd, *J* = 4.9, 2.2 Hz, 4H), 0.86 (dt, *J* = 7.0, 3.3 Hz, 3H).
^{13}C-NMR (75 MHz, DMSO-d$_6$, 298 K): δ = 133.58, 132.20, 132.09, 131.14, 130.90, 128.53, 48.91, 46.29, 39.52, 28.13, 24.88, 21.62, 13.72.
Yield: 99 % (0.99 mmol, 247 mg) as a white solid.

***N*-(3-chlorobenzyl)pentan-1-aminium chloride (38)**

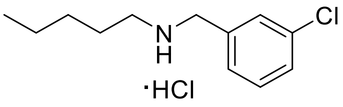

FW ($C_{12}H_{19}Cl_2N$) = 248.19 g mol^{-1}
^{1}H-NMR (300 MHz, DMSO-d$_6$, 298 K): δ = 9.51 (s, 2H), 7.73 (d, *J* = 1.4 Hz, 1H), 7.62 – 7.52 (m, 1H), 7.52 – 7.35 (m, 2H), 4.14 (d, *J* = 14.5 Hz, 2H), 2.83 (dd, *J* = 32.6, 15.7 Hz, 2H), 1.65 (dd, *J* = 13.7, 6.6 Hz, 2H),

1.28 (dd, *J* = 4.0, 3.1 Hz, 4H), 0.87 (dd, *J* = 9.0, 4.6 Hz, 3H).
^{13}C-NMR (75 MHz, DMSO-d$_6$, 298 K): δ = 134.56, 133.05, 130.41, 129.97, 128.85, 128.73, 49.03, 46.44, 39.52, 28.12, 24.88, 21.62, 13.72.
Yield: 99 % (0.99 mmol, 246 mg) as a white solid.

***N*-(2-chlorobenzyl)pentan-1-aminium chloride (39)**

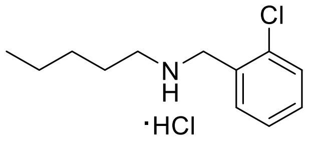

FW ($C_{12}H_{19}Cl_2N$) = 248.19 g mol^{-1}
^{1}H-NMR (300 MHz, DMSO-d$_6$, 298 K): δ = 9.49 (s, 2H), 7.89 – 7.74 (m, 1H), 7.64 – 7.50 (m, 1H), 7.50 – 7.39 (m, 2H), 4.23 (s, 2H), 2.93 (s, 2H), 1.68 (d, *J* = 7.6 Hz, 2H), 1.45 – 1.17 (m, 4H), 0.88 (dd, *J* = 9.4, 4.3 Hz, 3H).
^{13}C-NMR (75 MHz, DMSO-d$_6$, 298 K): δ = 133.52, 131.95, 130.77, 129.95, 129.55, 127.50, 46.86, 46.79, 39.52, 28.11, 24.86, 21.63, 13.73.
Yield: 96 % (0.96 mmol, 238 mg) as a white solid.

***N*-(4-bromobenzyl)pentan-1-aminium chloride (40)**

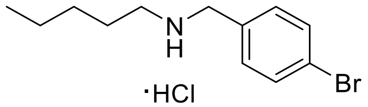

FW ($C_{12}H_{19}BrClN$) = 292.65 g mol^{-1}
^{1}H-NMR (300 MHz, DMSO-d$_6$, 298 K): δ = 9.40 (s, 2H), 7.59 (dt, *J* = 8.2, 6.4 Hz, 4H), 4.10 (d, *J* = 5.1 Hz, 3H), 2.82 (s, 2H), 1.65 (s, 2H), 1.38 – 1.14 (m, 4H), 1.06 – 0.73 (m, 3H).
^{13}C-NMR (75 MHz, DMSO-d$_6$, 298 K): δ = 132.47, 132.35, 131.49, 49.00, 46.32, 39.52, 28.11, 24.90, 21.62, 13.72.
Yield: 79 % (0.79 mmol, 231 mg) as a white solid.

***N*-(4-methoxybenzyl)pentan-1-aminium chloride (41)**

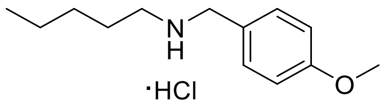

FW ($C_{13}H_{22}ClNO$) = 243.78 g mol^{-1}
^{1}H-NMR (300 MHz, DMSO-d$_6$, 298 K): δ = 9.26 (s, 2H), 7.53 – 7.45 (m, 2H), 7.03 – 6.92 (m, 2H), 4.02 (t, *J* = 5.2 Hz, 2H), 3.77 (dd, *J* = 3.9, 1.4 Hz, 3H), 2.79 (d, *J* = 3.5 Hz, 2H), 1.78 – 1.53 (m, 2H), 1.36 – 1.16 (m, 4H), 0.86 (q, *J* = 5.6 Hz, 3H).
^{13}C-NMR (75 MHz, DMSO-d$_6$, 298 K): δ = 159.60, 131.63, 123.88, 113.92, 55.20, 49.23, 45.98, 39.52,

28.16, 24.89, 21.62, 13.72.
Yield: 96 % (0.96 mmol, 234 mg) as a white solid.

***N*-(4-(benzyloxy)benzyl)pentan-1-aminium chloride (42)**

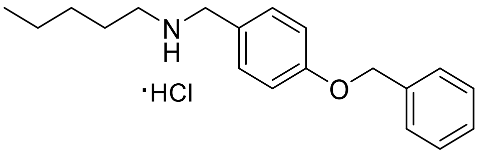

FW ($C_{19}H_{26}ClNO$) = 319.87 g mol^{-1}
^{1}H-NMR (300 MHz, DMSO-d$_6$, 298 K): δ = 9.29 (s, 2H), 7.54 – 7.27 (m, 7H), 7.04 (d, *J* = 8.7 Hz, 2H), 5.13 (s, 3H), 4.01 (s, 2H), 2.78 (d, *J* = 4.1 Hz, 2H), 1.74 – 1.54 (m, 2H), 1.26 (dd, *J* = 8.6, 5.5 Hz, 4H), 0.86 (t, *J* = 6.6 Hz, 3H).
^{13}C-NMR (75 MHz, DMSO-d$_6$, 298 K): δ = 158.62, 136.89, 131.64, 128.44, 127.86, 127.67, 124.14, 114.79, 69.17, 49.21, 46.00, 39.52, 28.15, 24.87, 21.62, 13.72.
Yield: 99 % (0.99 mmol, 317 mg) as a white solid.

***N*-(benzo[d][1,3]dioxol-5-ylmethyl)pentan-1-aminium chloride (43)**

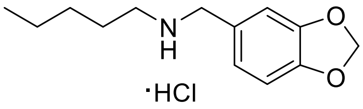

FW ($C_{13}H_{20}ClNO_2$) = 257.76 g mol^{-1}
^{1}H-NMR (300 MHz, DMSO-d$_6$, 298 K): δ = 9.41 (s, 2H), 7.24 (s, 1H), 7.07 – 6.89 (m, 2H), 6.04 (s, 2H), 3.99 (s, 2H), 2.92 – 2.60 (m, 2H), 1.65 (s, 2H), 1.39 – 1.12 (m, 4H), 0.85 (t, *J* = 6.2 Hz, 3H).
^{13}C-NMR (75 MHz, DMSO-d$_6$, 298 K): δ = 147.58, 147.24, 125.54, 124.13, 110.37, 108.20, 101.26, 49.46, 45.89, 39.52, 28.16, 24.86, 21.62, 13.72.
Yield: 89 % (0.89 mmol, 229 mg) as a white solid.

***N*-(4-(tert-butyl)benzyl)pentan-1-aminium chloride (44)**

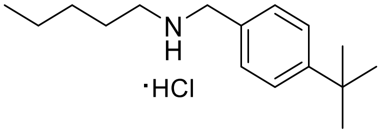

FW ($C_{16}H_{28}ClN$) = 269.86 g mol^{-1}
^{1}H-NMR (400 MHz, DMSO-d$_6$, 298 K): δ = 9.44 (s, 2H), 7.47 (dd, *J* = 36.5, 7.9 Hz, 4H), 4.04 (s, 2H), 2.87 – 2.74 (m, 2H), 1.67 (s, 2H), 1.27 (d, *J* = 5.2 Hz, 13H), 0.86 (s, 3H).
^{13}C-NMR (100 MHz, DMSO-d$_6$, 298 K): δ = 151.29, 129.89, 129.19, 126.29, 125.29, 124.69, 49.44, 46.28, 39.52, 34.37, 31.04, 28.17, 24.86, 21.63, 13.72.
Yield: 99 % (0.99 mmol, 267 mg) as a white solid.

N-benzylbutan-1-aminium chloride (45)

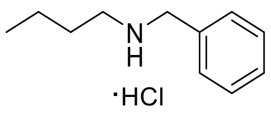

FW ($C_{11}H_{18}ClN$) = 199.72 g mol^{-1}
^{1}H-NMR (300 MHz, DMSO-d_6, 298 K): δ = 9.48 (s, 1H), 7.64 – 7.53 (m, 2H), 7.48 – 7.33 (m, 3H), 4.09 (t, *J* = 5.5 Hz, 2H), 2.82 (q, *J* = 11.3 Hz, 2H), 1.65 (dt, *J* = 21.9, 7.6 Hz, 2H), 1.42 – 1.17 (m, 2H), 0.87 (dd, *J* = 9.8, 4.9 Hz, 3H) ppm.
^{13}C-NMR (75 MHz, DMSO-d_6, 298 K): δ = 132.12, 131.89, 130.17, 130.09, 128.78, 128.55, 49.72, 46.05, 27.25, 19.38, 13.51 ppm.
Yield: 99 % (0.99 mmol, 198 mg) as a white solid.

N-benzylpentanaminium chloride (46)

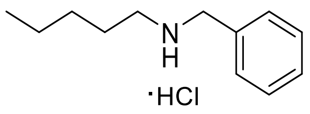

FW ($C_{12}H_{20}ClN$) = 213.75 g mol^{-1}
^{1}H-NMR (300 MHz, DMSO-d_6, 298 K): δ = 9.47 (s, 2H), 7.59 (dt, *J* = 4.2, 2.4 Hz, 2H), 7.46 – 7.34 (m, 3H), 4.09 (s, 2H), 2.81 (t, *J* = 24.1 Hz, 2H), 1.66 (d, *J* = 6.9 Hz, 2H), 1.26 (dd, *J* = 8.9, 5.2 Hz, 4H), 0.86 (t, *J* = 6.8 Hz, 3H) ppm.
^{13}C-NMR (75 MHz, DMSO-d_6, 298 K): δ = 132.12, 131.88, 130.16, 130.07, 128.77, 128.54, 49.72, 46.26, 28.14, 24.83, 21.60, 13.70 ppm.
Yield: 99 % (0.99 mmol, 211 mg) as a white solid.

N-benzyl-1-cyclohexylmethanaminium chloride (47)

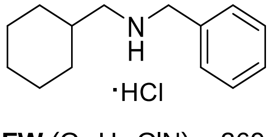

FW ($C_{14}H_{22}ClN$) = 269.79 g mol^{-1}
^{1}H-NMR (300 MHz, DMSO-d_6, 298 K): δ = 9.39 (s, 2H), 7.59 (td, *J* = 7.3, 2.9 Hz, 2H), 7.50 – 7.33 (m, 3H), 4.10 (t, *J* = 5.2 Hz, 2H), 2.68 (t, *J* = 11.0 Hz, 2H), 1.79 – 1.58 (m, 6H), 1.29 – 0.76 (m, 5H) ppm.
^{13}C-NMR (75 MHz, DMSO-d_6, 298 K): δ = 131.90, 130.25, 130.16, 128.80, 128.52, 51.98, 50.19, 34.14, 30.14, 25.53, 25.03 ppm.
Yield: 84 % (0.84 mmol, 227 mg) as a white solid.

N-benzyl-2,2-dimethylpropan-1-aminium chloride (48)

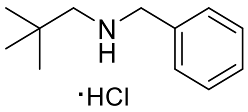

FW ($C_{12}H_{20}ClN$) = 213.75 g mol^{-1}
^{1}H-NMR (300 MHz, DMSO-d$_6$, 298 K): δ = 9.20 (s, 2H), 7.61 (ddd, *J* = 19.2, 6.6, 3.0 Hz, 2H), 7.47 – 7.35 (m, 3H), 4.19 – 4.06 (m, 2H), 2.65 – 2.54 (m, 2H), 0.95 (s, 9H) ppm.
^{13}C-NMR (75 MHz, DMSO-d$_6$, 298 K): δ = 131.84, 131.47, 130.40, 130.12, 128.90, 128.52, 50.65, 49.64, 30.19, 27.22 ppm.
Yield: 82 % (0.82 mmol, 175 mg) as a green solid.

3,7-dimethyl-N-pentyloct-6-en-1-aminium chloride (49)

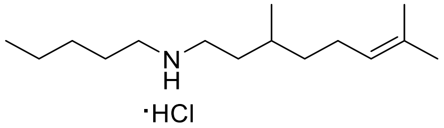

FW ($C_{15}H_{32}ClN$) = 261.88 g mol^{-1}
^{1}H-NMR (300 MHz, DMSO-d$_6$, 298 K): δ = 8.91 (s, 2H), 5.08 (td, *J* = 7.1, 1.2 Hz, 1H), 2.82 (s, 4H), 1.93 (d, *J* = 14.1 Hz, 2H), 1.76 – 1.54 (m, 8H), 1.44 (d, *J* = 14.4 Hz, 2H), 1.38 – 1.21 (m, 6H), 1.21 – 1.00 (m, 1H), 0.87 (t, *J* = 6.3 Hz, 6H).
^{13}C-NMR (75 MHz, DMSO-d$_6$, 298 K): δ = 130.71, 124.39, 46.63, 44.98, 39.52, 36.27, 32.15, 29.74, 28.15, 25.52, 25.06, 24.81, 21.64, 19.11, 17.57, 13.74.
Yield: 62 % (0.62 mmol, 162 mg) as a white solid.

N-butyldodecan-1-aminium chloride (50)

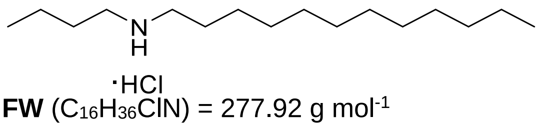

FW ($C_{16}H_{36}ClN$) = 277.92 g mol^{-1}
^{1}H-NMR (300 MHz, DMSO-d$_6$, 298 K): δ = 8.68 (s, 2H), 2.87 – 2.76 (m, 4H), 1.67 – 1.47 (m, 4H), 1.36 – 1.20 (m, 21H), 0.87 (dt, *J* = 9.2, 7.1 Hz, 6H) ppm.
^{13}C-NMR (75 MHz, DMSO-d$_6$, 298 K): δ = 46.66, 46.39, 31.30, 29.77, 29.02, 28.71, 27.79, 27.46, 26.90, 25.39, 24.15, 22.10, 19.32, 13.97, 13.51 ppm.
Yield: 51 % (0.51 mmol, 142 mg) as a white solid.

Dipentylaminium chloride (51)

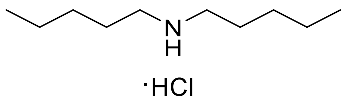

FW ($C_{10}H_{24}ClN$) = 157.30 g mol^{-1}
^{1}H-NMR (300 MHz, DMSO-d$_6$, 298 K): δ = 8.86 (s, 2H), 2.93 – 2.68 (m, 4H), 1.71 – 1.51 (m, 4H), 1.40 – 1.15 (m, 8H), 0.87 (t, *J* = 6.7 Hz, 6H) ppm.
^{13}C-NMR (75 MHz, DMSO-d$_6$, 298 K): δ = 46.59, 28.13, 25.03, 21.63, 13.73 ppm.
Yield: 54 % (0.54 mmol, 85 mg) as a white solid.

***N*-pentylheptan-1-aminium chloride (52)**

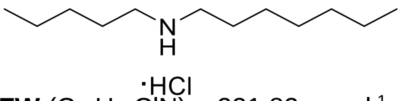

FW ($C_{12}H_{28}ClN$) = 221.82 g mol^{-1}
^{1}H-NMR (300 MHz, DMSO-d$_6$, 298 K): δ = 8.86 (s, 2H), 2.90 – 2.70 (m, 4H), 1.69 – 1.52 (m, 4H), 1.36 – 1.16 (m, 12H), 0.87 (td, *J* = 6.7, 2.3 Hz, 6H) ppm.
^{13}C-NMR (75 MHz, DMSO-d$_6$, 298 K): δ = 46.60, 31.03, 28.19, 28.13, 25.97, 25.03, 21.99, 21.63, 13.93, 13.73 ppm.
Yield: 47 % (0.47 mmol, 104 mg) as a white solid.

***N*-pentyldecan-1-aminium chloride (53)**

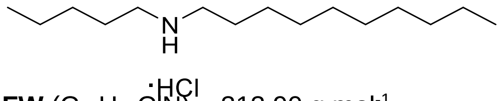

FW ($C_{15}H_{34}ClN$) = 313.90 g mol^{-1}
^{1}H-NMR (300 MHz, DMSO-d$_6$, 298 K): δ = 8.70 (s, 2H), 2.87 – 2.75 (m, 4H), 1.58 (d, *J* = 6.9 Hz, 4H), 1.36 – 1.16 (m, 22H), 0.86 (q, *J* = 6.7 Hz, 6H) ppm.
^{13}C-NMR (75 MHz, DMSO-d$_6$, 298 K): δ = 46.62, 31.30, 28.99, 28.72, 28.11, 25.07, 22.11, 21.63, 13.98, 13.73 ppm.
Yield: 62 % (0.62 mmol, 195 mg) as a white solid.

***N*-pentyldodecan-1-aminium chloride (54)**

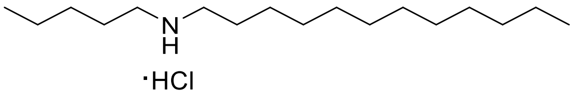

FW ($C_{17}H_{38}ClN$) = 291.95 g mol^{-1}
^{1}H-NMR (300 MHz, DMSO-d$_6$, 298 K): δ = 8.81 (s, 2H), 2.81 (td, *J* = 12.3, 7.6 Hz, 4H), 1.59 (d, *J* = 6.5 Hz, 4H), 1.37 – 1.17 (m, 22H), 0.86 (q, *J* = 6.7 Hz, 6H) ppm.
^{13}C-NMR (75 MHz, DMSO-d$_6$, 298 K): δ = 46.60, 31.30, 29.02, 28.72, 28.12, 25.04, 22.10, 21.63, 13.97,

13.73 ppm.
Yield: 59 % (0.59 mmol, 172 mg) as a white solid.

***N*-pentyldodecan-1-aminium chloride (55)**

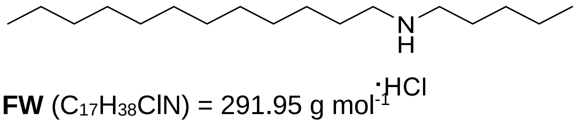

FW ($C_{17}H_{38}ClN$) = 291.95 g mol^{-1}
^{1}H-NMR (300 MHz, DMSO-d$_6$, 298 K): δ = 8.07 (s, 2H), 2.73 (s, 4H), 1.54 (s, 4H), 1.24 (s, 23H), 0.85 (s, 6H).
^{13}C-NMR (75 MHz, DMSO-d$_6$, 298 K): δ = 8.07, 2.73, 2.50, 1.54, 1.24, 0.85.
Yield: 57 % (0.57 mmol, 167 mg) as a white solid.

***N*-benzyl-1,1-diphenylmethanaminium chloride (56)**

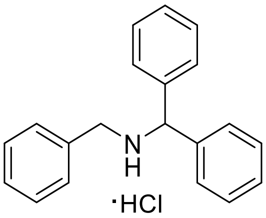

FW ($C_{20}H_{20}ClN$) = 309.84 g mol^{-1}
^{1}H-NMR (400 MHz, DMSO-d$_6$, 298 K): δ = 10.86 (s, 2H), 7.80 (d, *J* = 7.4 Hz, 3H), 7.50 (s, 3H), 7.37 (td, *J* = 14.5, 7.3 Hz, 9H), 5.48 (s, 1H), 4.03 (s, 2H).
^{13}C-NMR (100 MHz, DMSO-d$_6$, 298 K): δ = 136.86, 131.93, 130.26, 128.85, 128.42, 128.07, 64.65, 49.31, 39.52.
Yield: 71 % (0.71 mmol, 220 mg) as a white solid.

***N*-benzyl-1-phenylethan-1-aminium chloride (57)**

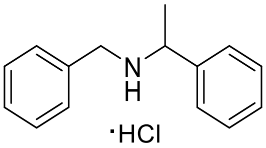

FW ($C_{15}H_{18}ClN$) = 247.77 g mol^{-1}
^{1}H-NMR (400 MHz, DMSO-d$_6$, 298 K): δ = 9.77 (s, 2H), 7.78 – 7.16 (m, 10H), 4.32 (s, 1H), 3.87 (dd, *J* = 97.1, 12.6 Hz, 2H), 1.64 (d, *J* = 6.5 Hz, 3H).
^{13}C-NMR (100 MHz, DMSO-d$_6$, 298 K): δ = 137.30, 131.88, 130.12, 128.93, 128.52, 127.93, 57.20, 48.30, 39.52, 19.72.
Yield: 69 % (0.69 mmol, 171 mg) as a white solid.

***N*-benzyloctan-2-aminium chloride (58)**

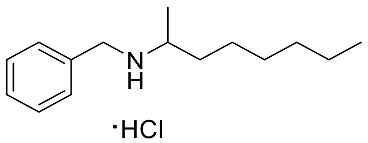

FW ($C_{15}H_{26}ClN$) = 255.83 g mol^{-1}
^{1}H-NMR (300 MHz, DMSO-d$_6$, 298 K): δ = 9.32 (d, *J* = 37.6 Hz, 2H), 7.73 – 7.53 (m, 2H), 7.50 – 7.33 (m, 3H), 4.11 (s, 2H), 3.06 (s, 1H), 1.96 – 1.41 (m, 3H), 1.41 – 1.11 (m, 10H), 0.86 (t, *J* = 6.5 Hz, 3H).
^{13}C-NMR (75 MHz, DMSO-d$_6$, 298 K): δ = 132.28, 130.16, 130.08, 128.75, 128.57, 53.01, 46.93, 39.52, 31.91, 31.04, 28.41, 24.78, 22.01, 15.47, 13.94.
Yield: 65 % (0.65 mmol, 166 mg) as a white solid.

***N*-benzylcyclopentanaminium chloride (59)**

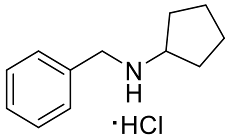

FW ($C_{12}H_{18}ClN$) = 211.73 g mol^{-1}
^{1}H-NMR (300 MHz, DMSO-d$_6$, 298 K): δ = 9.25 (s, 2H), 7.65 – 7.51 (m, 2H), 7.50 – 7.32 (m, 3H), 4.09 (s, 2H), 3.37 (dd, *J* = 15.4, 8.3 Hz, 1H), 1.93 (dd, *J* = 12.1, 4.1 Hz, 2H), 1.80 – 1.60 (m, 4H), 1.50 (s, 2H).
^{13}C-NMR (75 MHz, DMSO-d$_6$, 298 K): δ = 132.20, 130.11, 128.77, 128.55, 57.86, 48.90, 39.52, 28.99, 23.63.
Yield: 85 % (0.85 mmol, 180 mg) as a white solid.

***N*-benzhydrylpentan-1-aminium chloride (60)**

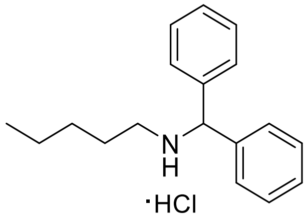

FW ($C_{18}H_{24}ClN$) = 289.85 g mol^{-1}
^{1}H-NMR (400 MHz, DMSO-d$_6$, 298 K): δ = 7.3 Hz, 4H), 7.38 (dt, *J* = 28.8, 7.3 Hz, 6H), 5.57 (s, 1H), 2.73 (s, 2H), 1.86 – 1.66 (m, 2H), 1.19 (dd, *J* = 8.5, 4.9 Hz, 4H), 0.82 (t, *J* = 6.8 Hz, 3H).
^{13}C-NMR (100 MHz, DMSO-d$_6$, 298 K): δ = 137.02, 128.90, 128.47, 127.87, 64.55, 46.05, 39.52, 28.14, 24.62, 21.57, 13.65.
Yield: 62 % (0.62 mmol, 180 mg) as a white solid.

***N*-(1-phenylethyl)pentan-1-aminium chloride (61)**

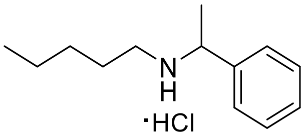

FW ($C_{13}H_{23}ClN$) = 227.78 g mol^{-1}
^{1}H-NMR (400 MHz, DMSO-d$_6$, 298 K): δ = 9.10 (s, 2H), 7.41 (d, *J* = 7.5 Hz, 2H), 7.33 – 7.10 (m, 3H), 4.10 (d, *J* = 5.7 Hz, 1H), 2.29 (d, *J* = 1.6 Hz, 2H), 1.40 (t, *J* = 12.1 Hz, 5H), 0.99 (s, 4H), 0.60 (d, *J* = 6.7 Hz, 3H).
^{13}C-NMR (100 MHz, DMSO-d$_6$, 298 K): δ = 137.46, 128.85, 128.75, 127.80, 56.98, 44.82, 39.52, 28.12, 24.95, 21.57, 19.67, 13.65.
Yield: 61 % (0.61 mmol, 139 mg) as a white solid.

***N*-pentyloctan-2-aminium chloride (62)**

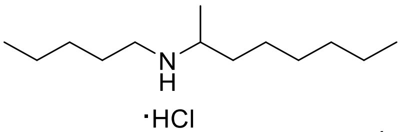

FW ($C_{13}H_{30}ClN$) = 235.84 g mol^{-1}
^{1}H-NMR (300 MHz, DMSO-d$_6$, 298 K): δ = 8.84 (s, 2H), 3.07 (s, 1H), 2.91 – 2.75 (m, 2H), 1.82 – 1.54 (m, 3H), 1.35 – 1.16 (m, 14H), 0.99 – 0.78 (m, 6H).
^{13}C-NMR (75 MHz, DMSO-d$_6$, 298 K): δ = 53.09, 43.62, 39.52, 32.07, 31.07, 28.47, 28.22, 25.27, 24.76, 22.01, 21.66, 15.49, 13.94, 13.75.
Yield: 57 % (0.57 mmol, 135 mg) as a white solid.

***N*-pentylcyclopentanaminium chloride (63)**

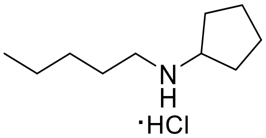

FW ($C_{10}H_{22}ClN$) = 191.74 g mol^{-1}
^{1}H-NMR (300 MHz, DMSO-d$_6$, 298 K): δ = 8.81 (s, 2H), 3.38 (dd, *J* = 13.9, 6.9 Hz, 1H), 2.94 – 2.64 (m, 2H), 1.92 (d, *J* = 8.2 Hz, 2H), 1.80 – 1.39 (m, 8H), 1.39 – 1.13 (m, 4H), 0.87 (t, *J* = 6.5 Hz, 3H).
^{13}C-NMR (75 MHz, DMSO-d$_6$, 298 K): δ = 58.12, 56.95, 45.67, 39.52, 28.96, 28.20, 25.25, 23.57, 21.67, 13.74.
Yield: 70 % (0.70 mmol, 134 mg) as a white solid.

***N*-benzyl-4-(6-methoxynaphthalen-2-yl)butan-2-aminium chloride (64)**

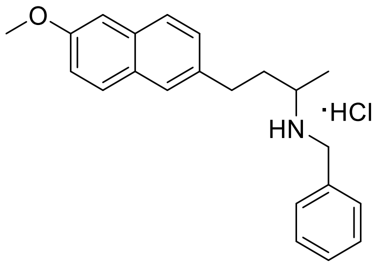

FW ($C_{22}H_{26}ClNO$) = 355.91 g mol^{-1}
^{1}H-NMR (300 MHz, DMSO-d_6, 298 K): δ = 9.61 (s, 2H), 7.71 (dd, *J* = 20.2, 17.7 Hz, 5H), 7.49 – 7.22 (m, 4H), 7.14 (d, *J* = 6.7 Hz, 1H), 4.13 (d, *J* = 10.0 Hz, 2H), 3.85 (s, 3H), 3.16 (d, *J* = 46.5 Hz, 1H), 2.97 – 2.60 (m, 2H), 2.38 – 1.73 (m, 2H), 1.37 (dd, *J* = 16.5, 5.6 Hz, 3H).
^{13}C-NMR (75 MHz, DMSO-d_6, 298 K): δ = 156.83, 135.85, 132.84, 132.20, 130.14, 128.80, 128.68, 128.54, 128.50, 127.59, 126.84, 126.06, 118.56, 105.78, 55.15, 52.47, 46.85, 39.52, 33.59, 30.81, 15.46.
Yield: 78 % (0.78 mmol, 278 mg) as a white solid.
Elemental analysis: calcd.: C 74.24, H 7.36, N 3.94; found: C 74.18, H 7.30, N 3.60

_N_-(4-(6-methoxynaphthalen-2-yl)butan-2-yl)pentan-1-aminium chloride (65)

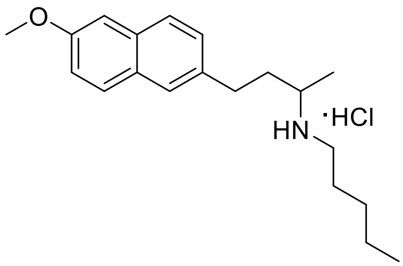

FW ($C_{20}H_{30}ClNO$) = 335.92 g mol^{-1}
^{1}H-NMR (400 MHz, DMSO-d_6, 298 K): δ = 9.08 (s, 2H), 7.83 – 7.54 (m, 3H), 7.41 – 7.22 (m, 2H), 7.13 (dd, *J* = 8.9, 2.6 Hz, 1H), 3.85 (s, 3H), 3.11 (s, 1H), 2.91 – 2.76 (m, 3H), 2.76 – 2.62 (m, 1H), 2.28 – 2.06 (m, 1H), 1.85 (dd, *J* = 9.0, 4.6 Hz, 1H), 1.63 (s, 2H), 1.38 – 1.21 (m, 7H), 0.85 (t, *J* = 7.0 Hz, 3H).
^{13}C-NMR (100 MHz, DMSO-d_6, 298 K): δ = 156.83, 135.95, 132.83, 128.77, 128.55, 127.56, 126.84, 126.01, 118.56, 105.77, 55.12, 52.76, 43.62, 39.52, 33.72, 30.81, 28.22, 25.25, 21.66, 15.48, 13.73.
Yield: 77 % (0.77 mmol, 259 mg) as a white solid.
Elemental analysis: calcd.: C 71.51, H 9.00, N 4.17; found: C 71.74, H 8.72, N 4.32

1-(5-(benzylamino)hexyl)-3,7-dimethyl-3,7-dihydro-1_H_-purine-2,6-dione hydrochloride (66)

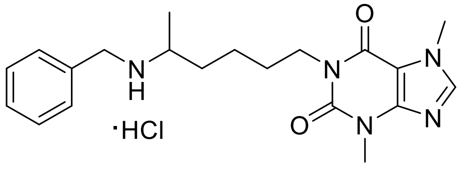

FW ($C_{20}H_{28}ClN_5O_2$) = 405.93 g mol^{-1}
^{1}H-NMR (400 MHz, DMSO-d_6, 298 K): δ = 8.62 (s, 2H), 8.04 (s, 1H), 7.63 (d, *J* = 6.0 Hz, 2H), 7.38 (d, *J* = 6.7 Hz, 3H), 4.10 (d, *J* = 4.4 Hz, 2H), 3.91 – 3.75 (m, 5H), 3.38 (s, 3H), 3.05 (s, 1H), 1.95 – 1.16 (m, 9H).
^{13}C-NMR (100 MHz, DMSO-d_6, 298 K): δ = 154.40, 150.82, 148.12, 142.90, 132.32, 130.14, 128.72, 128.56, 106.67, 52.99, 46.98, 39.52, 33.24, 31.65, 29.46, 27.29, 22.35, 15.48.

Yield: 76 % (0.76 mmol, 309 mg) as a white solid.
Elemental analysis: calcd.: C 59.18, H 6.95, N 17.25; found: C 58.91, H 6.86, N 16.97

3,7-dimethyl-1-(5-(pentylamino)hexyl)-3,7-dihydro-1*H*-purine-2,6-dione hydrochloride (67)

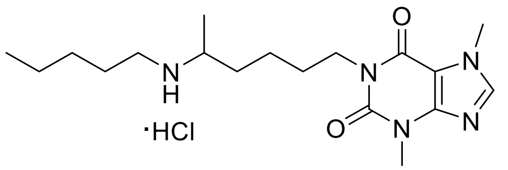

FW ($C_{18}H_{32}ClN_5O_2$) = 385.94 g mol^{-1}
^{1}H-NMR (400 MHz, DMSO-d$_6$, 298 K): δ = 9.02 (s, 2H), 8.03 (s, 1H), 3.94 – 3.78 (m, 5H), 3.39 (s, 3H), 3.07 (s, 1H), 2.81 (s, 2H), 1.87 – 1.13 (m, 15H), 0.86 (s, 3H).
^{13}C-NMR (100 MHz, DMSO-d$_6$, 298 K): δ = 154.38, 150.81, 148.15, 142.92, 106.64, 53.00, 43.68, 39.52, 33.18, 31.67, 29.41, 28.27, 27.24, 25.24, 22.26, 21.69, 15.52, 13.77.
Yield: 72 % (0.72 mmol, 278 mg) as a white solid.
Elemental analysis: calcd.: C 56.02, H 8.36, N 18.15; found: C 55.76, H 8.72, N 17.94

Tecalcet hydrochloride (68)

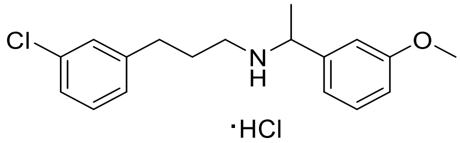

FW ($C_{18}H_{23}Cl_2NO$) = 340.29 g mol^{-1}
^{1}H-NMR (400 MHz, DMSO-d$_6$, 298 K): δ = 9.42 (s, 2H), 7.42 – 7.19 (m, 6H), 7.13 (d, *J* = 7.8 Hz, 1H), 6.94 (ddd, *J* = 8.3, 2.5, 0.8 Hz, 1H), 4.30 (d, *J* = 6.4 Hz, 1H), 3.77 (s, 3H), 2.84 – 2.53 (m, 4H), 2.06 – 1.85 (m, 2H), 1.58 (d, *J* = 6.8 Hz, 3H).
^{13}C-NMR (100 MHz, DMSO-d$_6$, 298 K): δ = 159.54, 138.83, 138.06, 132.81, 130.56, 129.96, 129.27, 128.11, 127.29, 119.90, 114.33, 113.33, 57.01, 55.24, 44.30, 39.52, 29.80, 25.40, 19.67.
Yield: 66 % (0.66 mmol, 226 mg) as a white solid.

(8*R*,9*S*,13*S*,14*S*)-17-(benzylamino)-13-methyl-7,8,9,11,12,13,14,15,16,17-decahydro-6*H*-cyclopenta[a]phenanthren-3-ol hydrochloride (69)

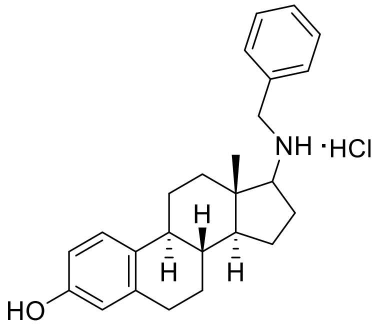

FW ($C_{25}H_{32}ClNO$) = 394.99 g mol^{-1}
^{1}H-NMR (400 MHz, DMSO-d$_6$, 298 K): δ = 9.48 (s, 2H), 9.17 (s, 1H), 7.41 (ddd, *J* = 22.9, 14.9, 7.4 Hz, 5H), 7.05 (d, *J* = 8.2 Hz, 1H), 6.63 – 6.35 (m, 2H), 4.78 (q, *J* = 14.8 Hz, 2H), 3.14 – 2.60 (m, 4H), 2.42 – 0.72 (m, 16H).
^{13}C-NMR (100 MHz, DMSO-d$_6$, 298 K): δ = 155.19, 136.93, 129.45, 128.75, 128.25, 126.05, 114.96, 112.85, 51.82, 50.84, 49.58, 48.32, 47.34, 46.30, 42.94, 39.52, 37.56, 32.48, 28.96, 26.67, 25.49, 22.46, 15.74, 1.58.
Yield: 80 % (0.80 mmol, 316 mg) as a white solid.

(8*R*,9*S*,13*S*,14*S*)-13-methyl-17-(pentylamino)-7,8,9,11,12,13,14,15,16,17-decahydro-6*H*-cyclopenta[a]phenanthren-3-ol hydrochloride (70)

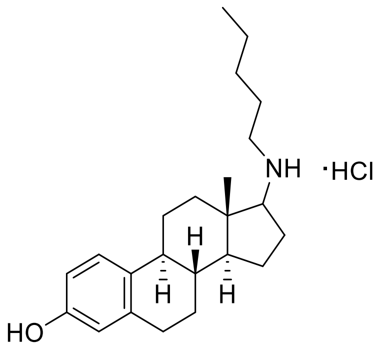

FW ($C_{24}H_{42}ClNO$) = 378.00 g mol^{-1}
^{1}H-NMR (500 MHz, DMSO-d$_6$, 298 K): δ = 9.04 (s, 1H), 8.52 (s, 3H), 7.04 (d, *J* = 8.7 Hz, 1H), 6.56 – 6.40

(m, 2H), 3.06 (d, *J* = 5.8 Hz, 1H), 2.95 – 2.84 (m, 2H), 2.71 (d, *J* = 5.2 Hz, 2H), 2.31 – 0.74 (m, 24H).
^{13}C-NMR (125 MHz, DMSO-d$_6$, 298 K): δ = 155.04, 137.01, 129.90, 126.07, 114.94, 112.79, 66.43, 51.01, 46.84, 43.10, 42.29, 39.52, 38.00, 36.07, 29.05, 28.21, 26.94, 25.64, 25.11, 24.94, 24.77, 22.82, 21.71, 13.79, 11.82.
Yield: 74 % (0.74 mmol, 280 mg) as a white solid.
Elemental analysis: calcd.: C 73.08, H 9.60, N 3.71; found: C 72.01, H 9.11, N 3.25

***N*-benzyl-1,1-diphenylmethanaminium chloride(8*R*,9*S*,10*S*,13*S*,14*S*,17*S*)-3-(benzylamino)-10,13-dimethylhexadecahydro-1H-cyclopenta[a]phenanthren-17-ol hydrochloride (71)**

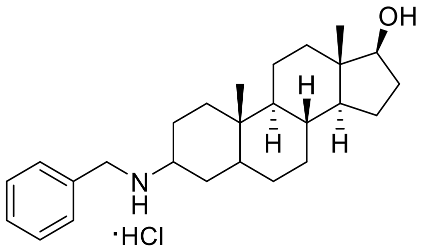

FW ($C_{26}H_{40}ClNO$) = 418.06 g mol^{-1}
^{1}H-NMR (400 MHz, DMSO-d$_6$, 298 K): δ = 9.30 (s, 2H), 7.61 (dd, *J* = 14.5, 7.0 Hz, 2H), 7.41 (d, *J* = 6.4 Hz, 3H), 4.43 (d, *J* = 3.9 Hz, 1H), 4.12 (d, *J* = 13.8 Hz, 2H), 2.97 (s, 1H), 2.01 – 0.70 (m, 26H), 0.62 (s, 3H).
^{13}C-NMR (100 MHz, DMSO-d$_6$, 298 K): δ = 132.43, 130.31, 130.00, 128.75, 128.59, 80.01, 55.94, 53.54, 50.78, 50.53, 46.92, 44.24, 42.57, 42.54, 39.52, 36.56, 36.08, 35.47, 35.30, 35.29, 35.06, 31.14, 30.21, 29.84, 28.07, 27.57, 24.90, 23.92, 23.05, 20.34, 11.81, 11.34.
Yield: 73 % (0.73 mmol, 305 mg) as a white solid.

(8*R*,9*S*,10*S*,13*S*,14*S*,17*S*)-10,13-dimethyl-3-(pentylamino)hexadecahydro-1H-cyclopenta[a]phenanthren-17-ol hydrochloride (72)

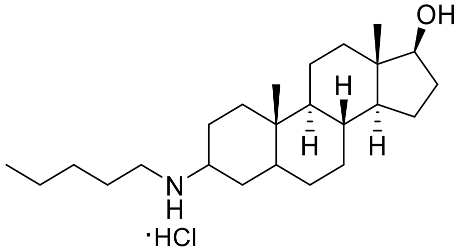

FW ($C_{24}H_{44}ClNO$) = 398.07 g mol^{-1}
^{1}H-NMR (400 MHz, DMSO-d$_6$, 298 K): δ = 8.92 (s, 2H), 4.43 (d, *J* = 3.9 Hz, 1H), 3.41 (d, *J* = 4.7 Hz, 1H), 2.87 (d, *J* = 50.3 Hz, 2H), 1.91 – 0.51 (m, 38H).
^{13}C-NMR (100 MHz, DMSO-d$_6$, 298 K): δ = 80.00, 55.71, 53.62, 50.54, 44.16, 43.47, 42.54, 39.52, 36.57, 36.05, 35.28, 35.06, 31.16, 30.27, 29.84, 28.22, 28.05, 25.30, 24.01, 23.05, 21.66, 20.34, 13.75, 11.81, 11.33.
Yield: 69 % (0.69 mmol, 275 mg) as a white solid.
Elemental analysis: calcd.: C 72.42, H 11.14, N 3.52; found: C 72.21, H 11.08, N 3.39

(8*R*,9*S*,10*R*,13*S*,14*S*,17*S*)-3-(benzylamino)-10,13-dimethyl-2,3,6,7,8,9,10,11,12,13,14,15,16,17-tetradecahydro-1H-cyclopenta[a]phenanthren-17-ol hydrochloride (73)

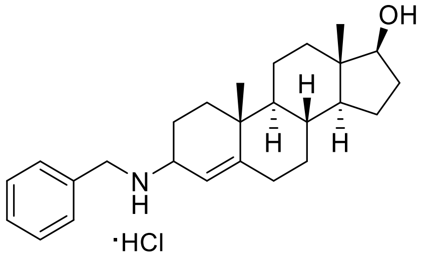

FW ($C_{26}H_{38}ClNO$) = 416.05 g mol^{-1}
^{1}H-NMR (400 MHz, DMSO-d_6, 298 K): δ = 9.17 (s, 2H), 7.56 (d, *J* = 8.0 Hz, 2H), 7.42 (t, *J* = 6.7 Hz, 3H), 5.45 (s, 1H), 4.31 – 4.04 (m, 2H), 3.42 (t, *J* = 8.4 Hz, 1H), 2.26 – 0.59 (m, 27H).
^{13}C-NMR (100 MHz, DMSO-d_6, 298 K): δ = 150.68, 132.32, 129.96, 128.65, 114.83, 109.59, 79.91, 53.90, 50.15, 42.43, 39.52, 36.97, 36.39, 35.39, 35.09, 34.74, 32.10, 31.78, 29.80, 23.06, 21.79, 20.31, 18.40, 11.24.
Yield: 76 % (0.76 mmol, 316 mg) as a white solid.
Elemental analysis: calcd.: C 75.06, H 9.21 N 3.37; found: C 74.05, H 9.54, N 3.25

(8*R*,9*S*,10*R*,13*S*,14*S*,17*S*)-10,13-dimethyl-3-(pentylamino)-2,3,6,7,8,9,10,11,12,13,14,15,16,17-tetradecahydro-1*H*-cyclopenta[a]phenanthren-17-ol hydrochloride (74)

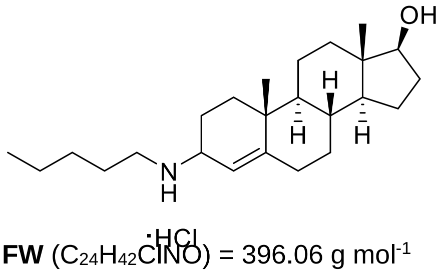

FW ($C_{24}H_{42}ClNO$) = 396.06 g mol^{-1}
^{1}H-NMR (500 MHz, DMSO-d_6, 298 K): δ = 8.83 (s, 2H), 5.38 (s, 1H), 4.47 (d, *J* = 4.8 Hz, 1H), 2.85 (dd, *J* = 15.0, 7.5 Hz, 2H), 2.24 – 0.52 (m, 36H).
^{13}C-NMR (125 MHz, DMSO-d_6, 298 K): δ = 150.33, 115.19, 79.92, 53.96, 53.69, 50.17, 43.35, 42.44, 39.52, 36.95, 36.40, 35.39, 34.73, 32.08, 31.71, 29.80, 28.21, 25.43, 23.07, 21.67, 20.31, 18.42, 13.77, 11.25.
Yield: 73 % (0.73 mmol, 289 mg) as a white solid.
Elemental analysis: calcd.: C 72.78, H 10.69, N 3.54; found: C 73.76, H 10.62, N 3.22

6. NMR spectra

N-benzyl-1-(*p*-tolyl)methanaminium chloride (1)

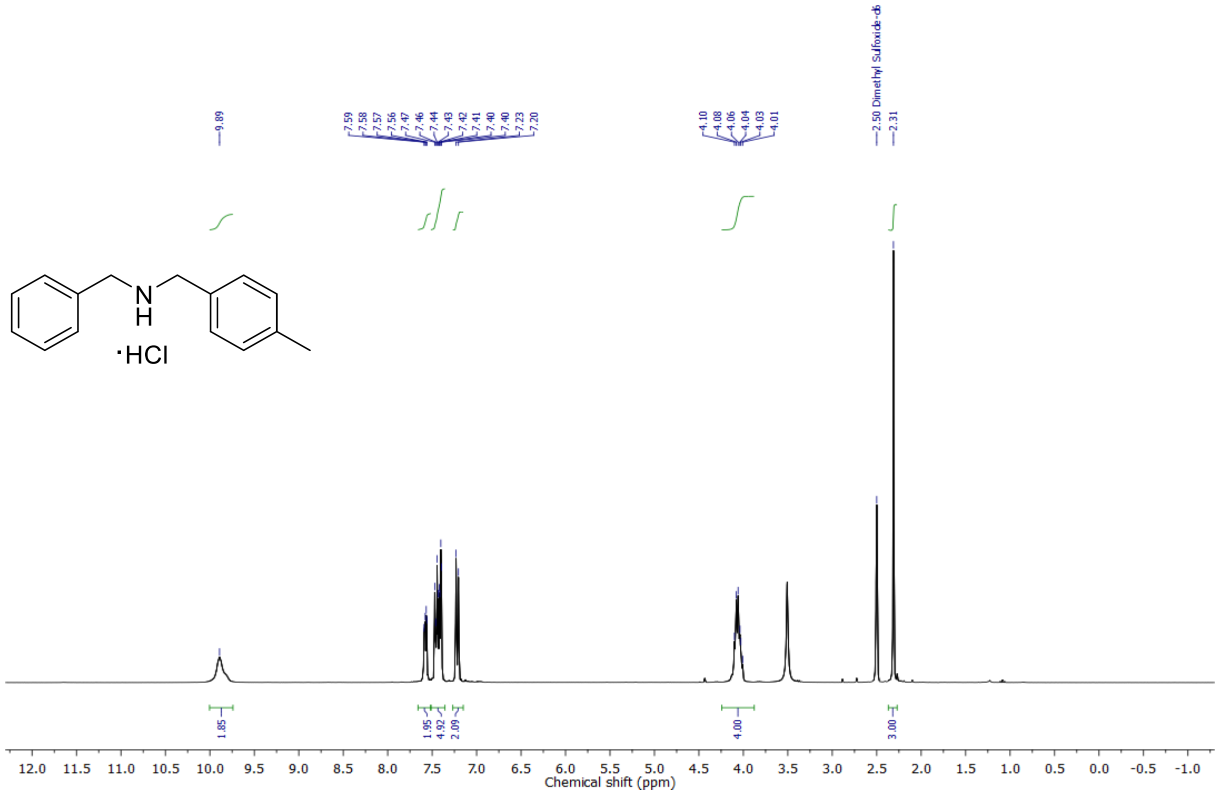

Figure S10: ^{1}H-NMR spectrum of **1**.

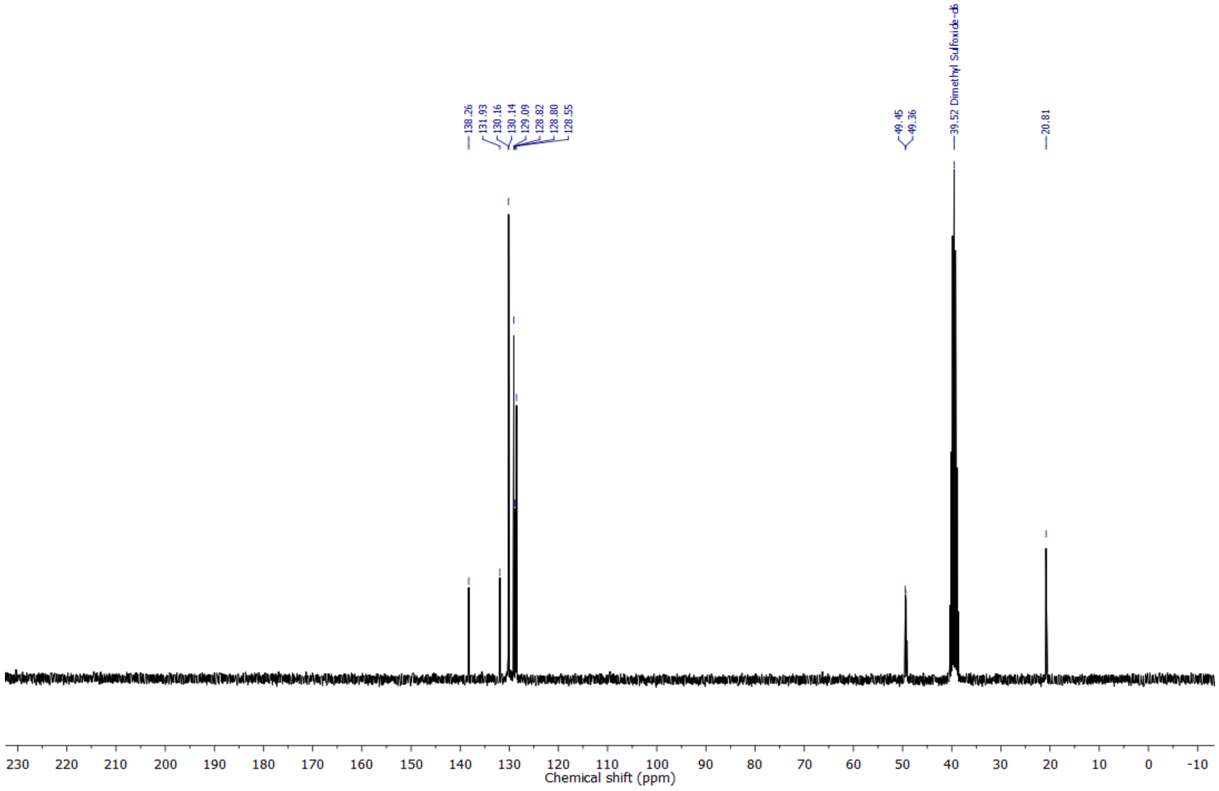

Figure S11: ^{13}C-NMR spectrum of **1**.

N-benzyl-1-(m-tolyl)methanaminium chloride (2)

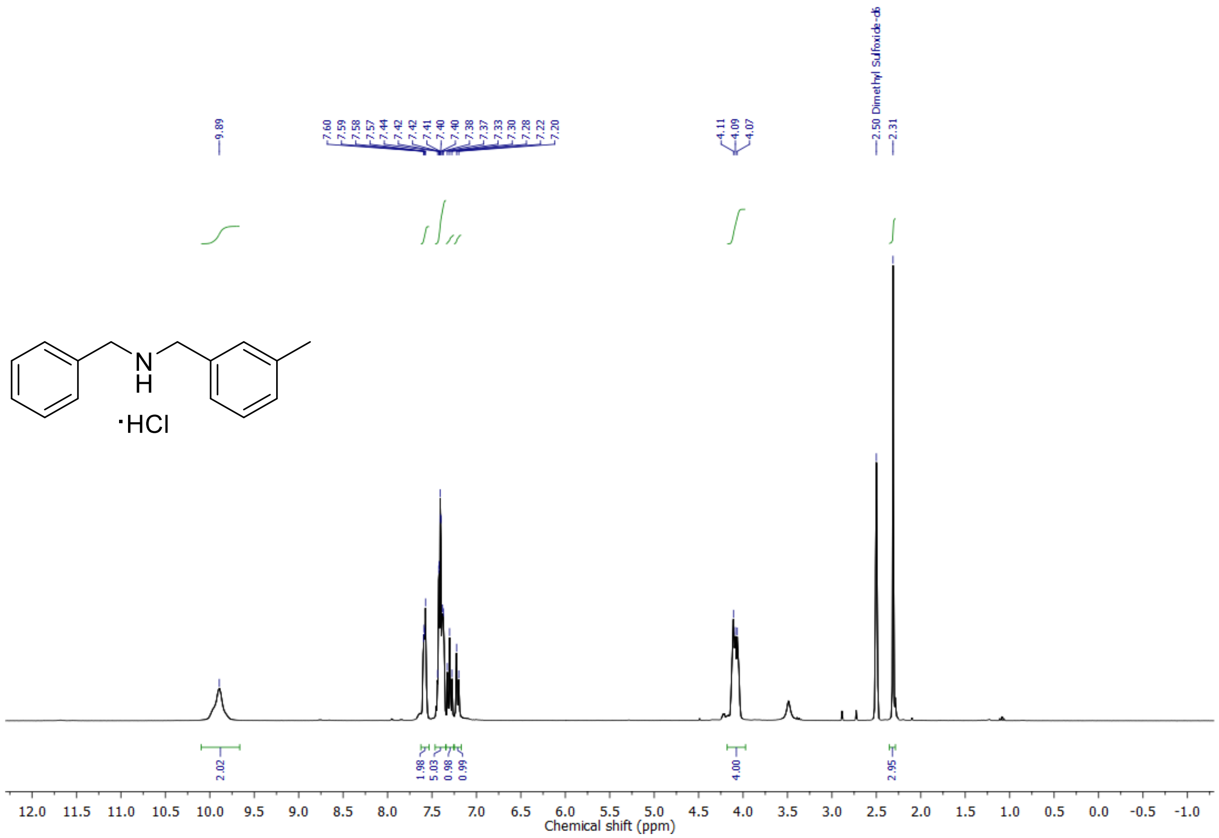

Figure S12: ^{1}H-NMR spectrum of **2**.

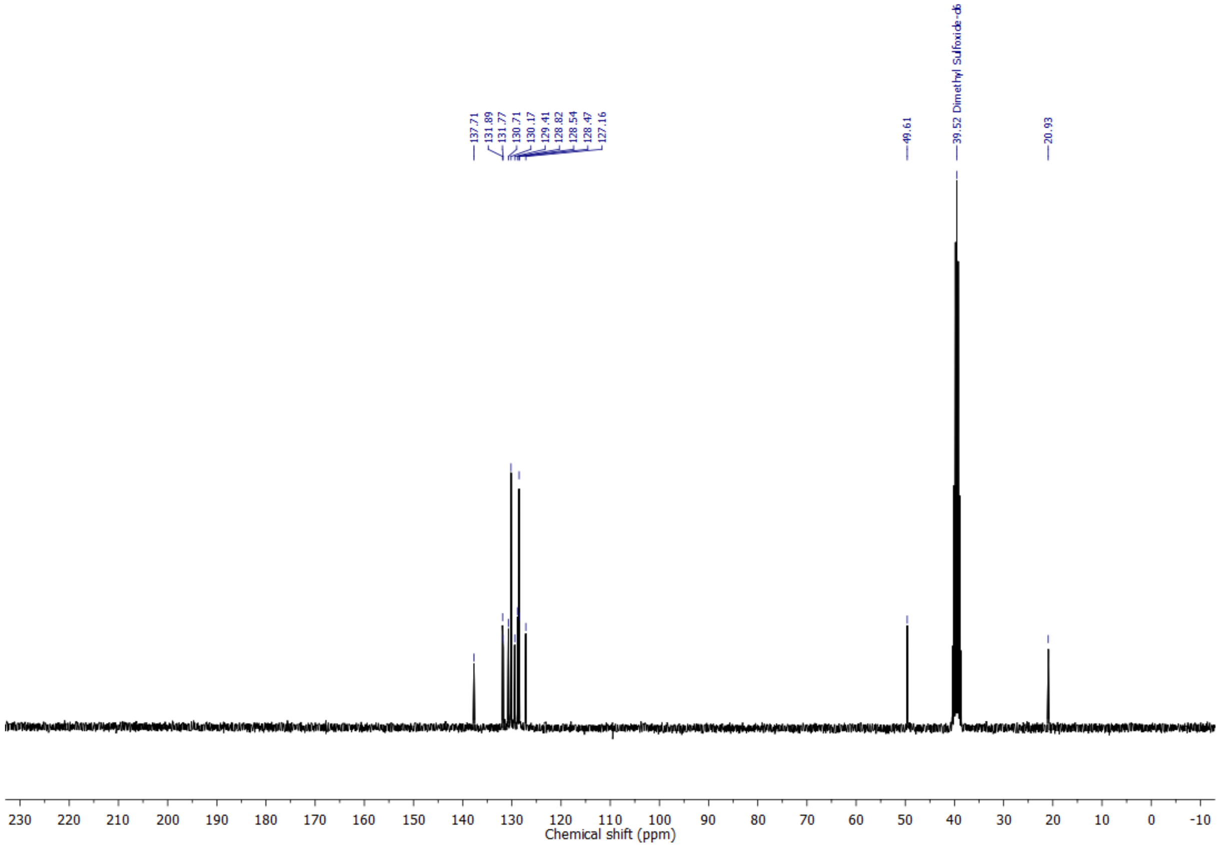

Figure S13: ^{13}C-NMR spectrum of **2**.

N-benzyl-1-(*o*-tolyl)methanaminium chloride (3)

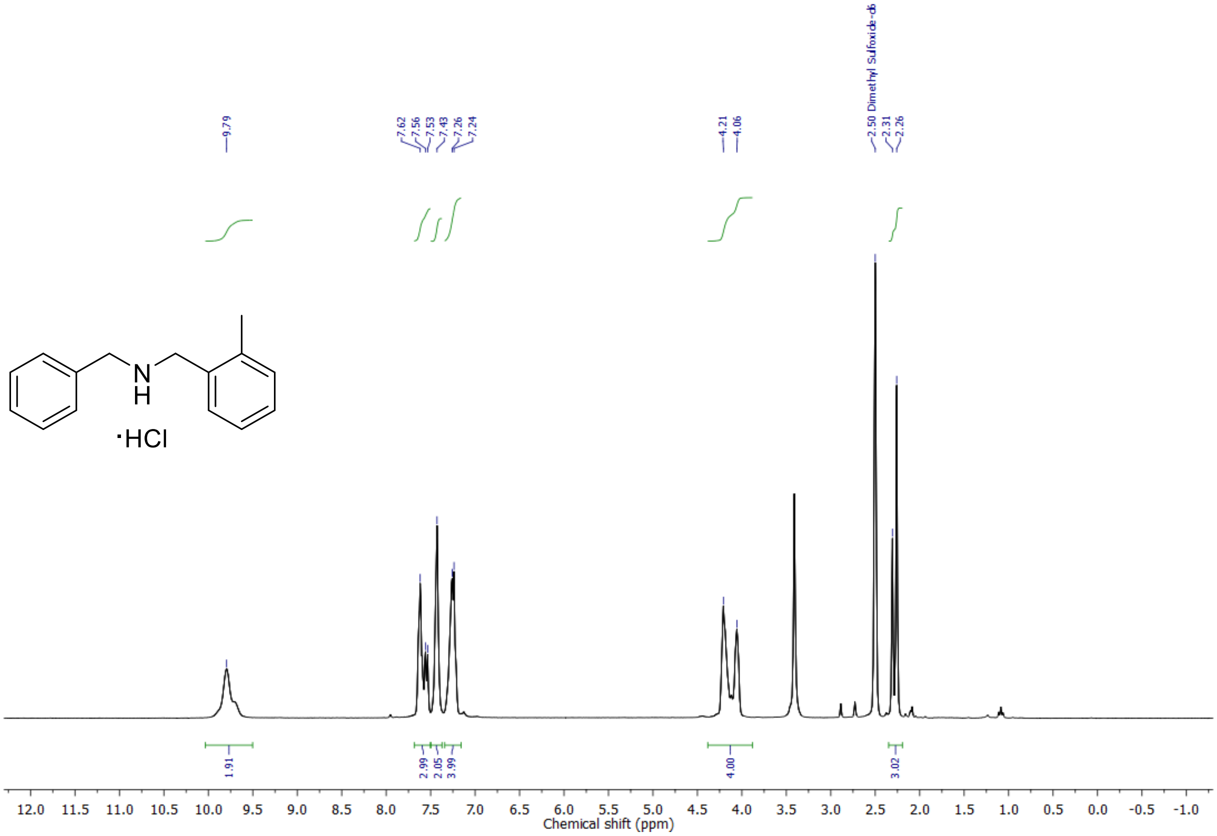

Figure S14: ^{1}H-NMR spectrum of **3**.

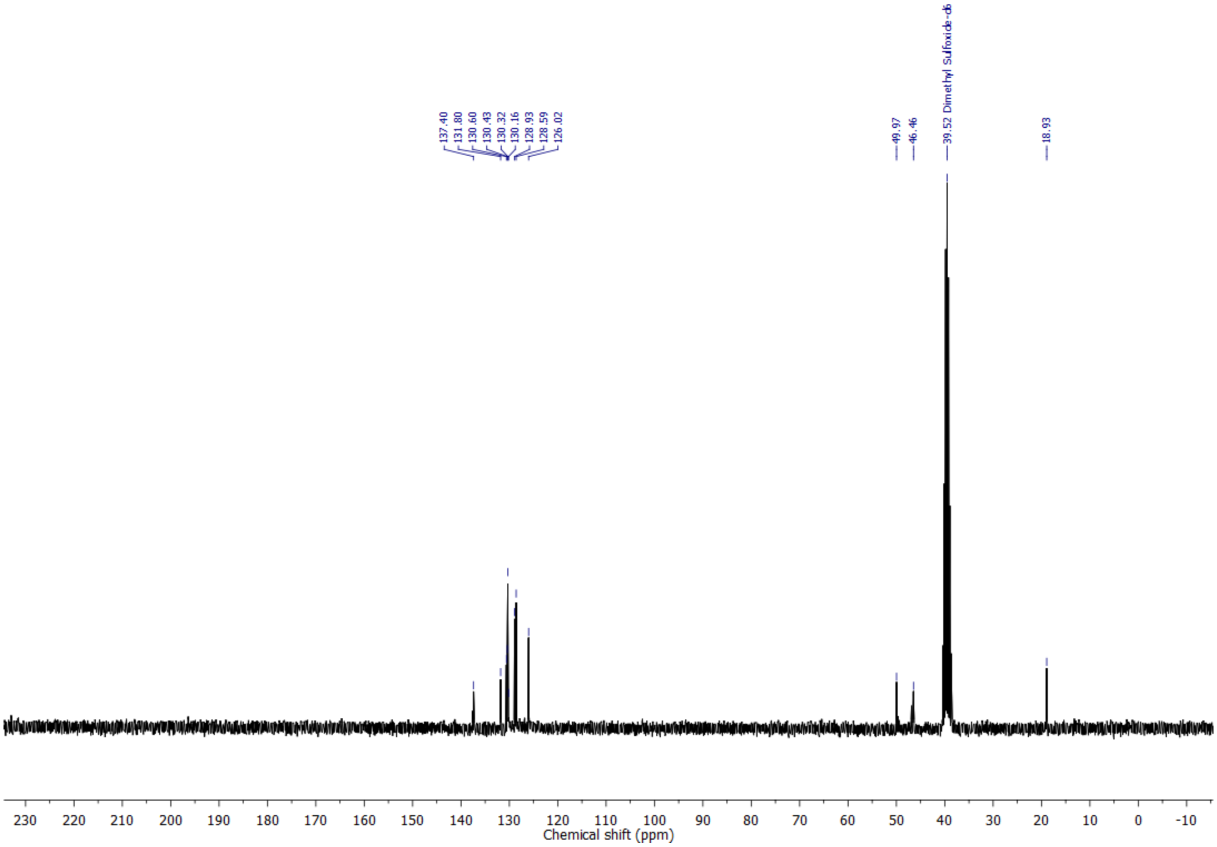

Figure S15: ^{13}C-NMR spectrum of **3**.

N-benzyl-1-(4-fluorophenyl)methanaminium chloride (4)

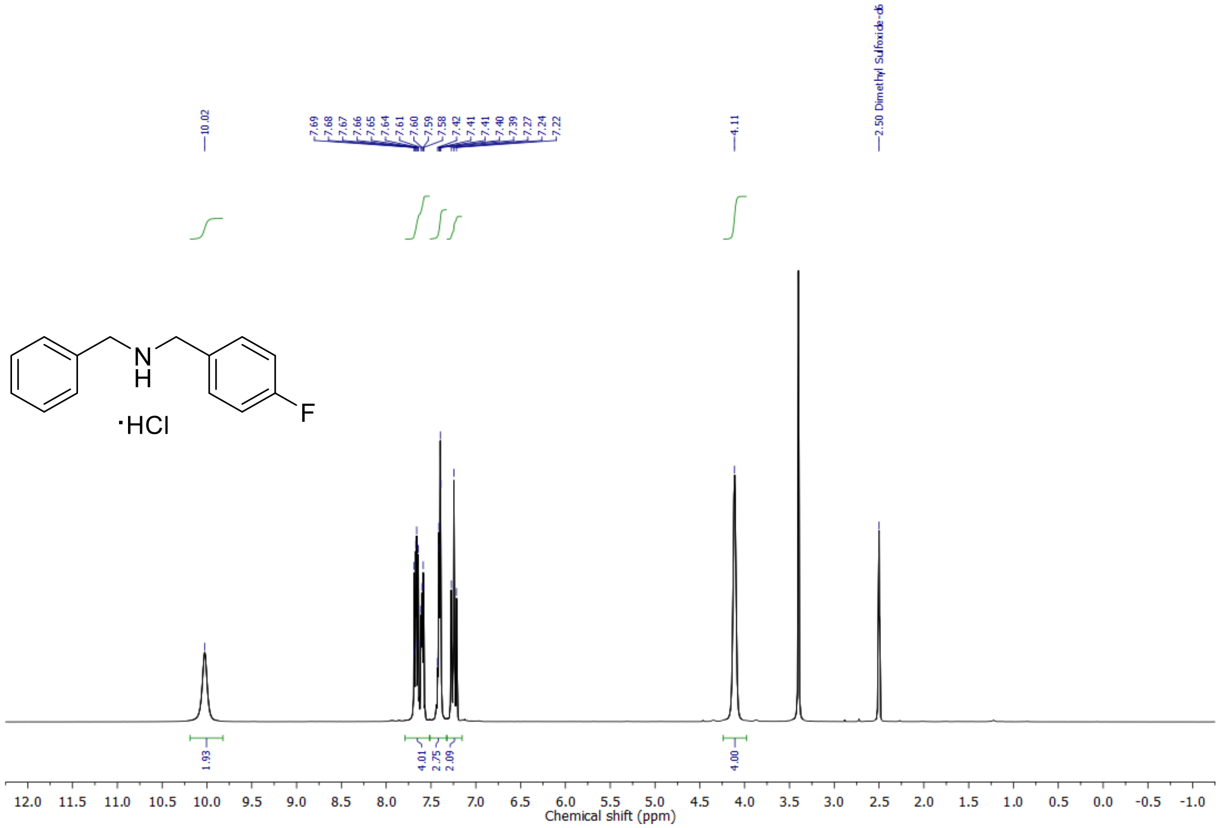

Figure S16: ^{1}H-NMR spectrum of **4**.

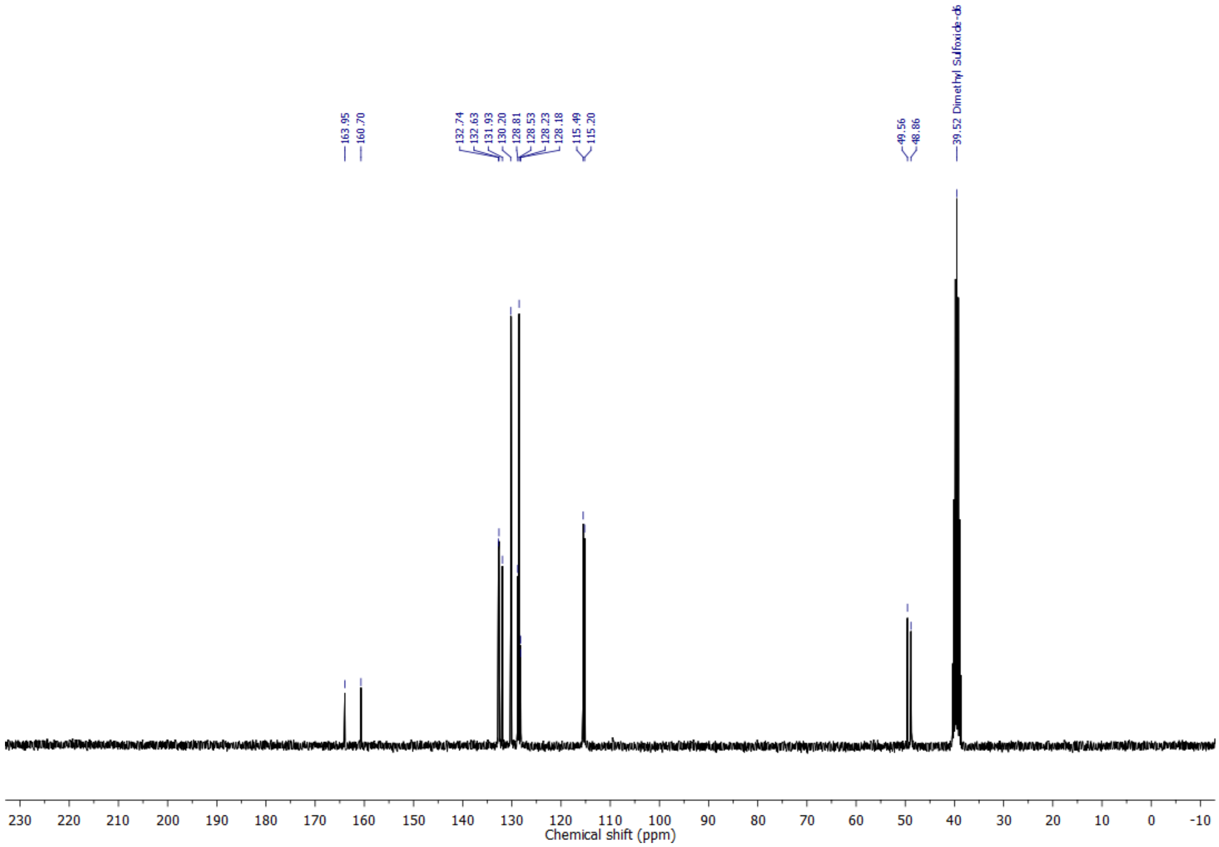

Figure S17: ^{13}C-NMR spectrum of **4**.

N-benzyl-1-(4-chlorophenyl)methanaminium chloride (5)

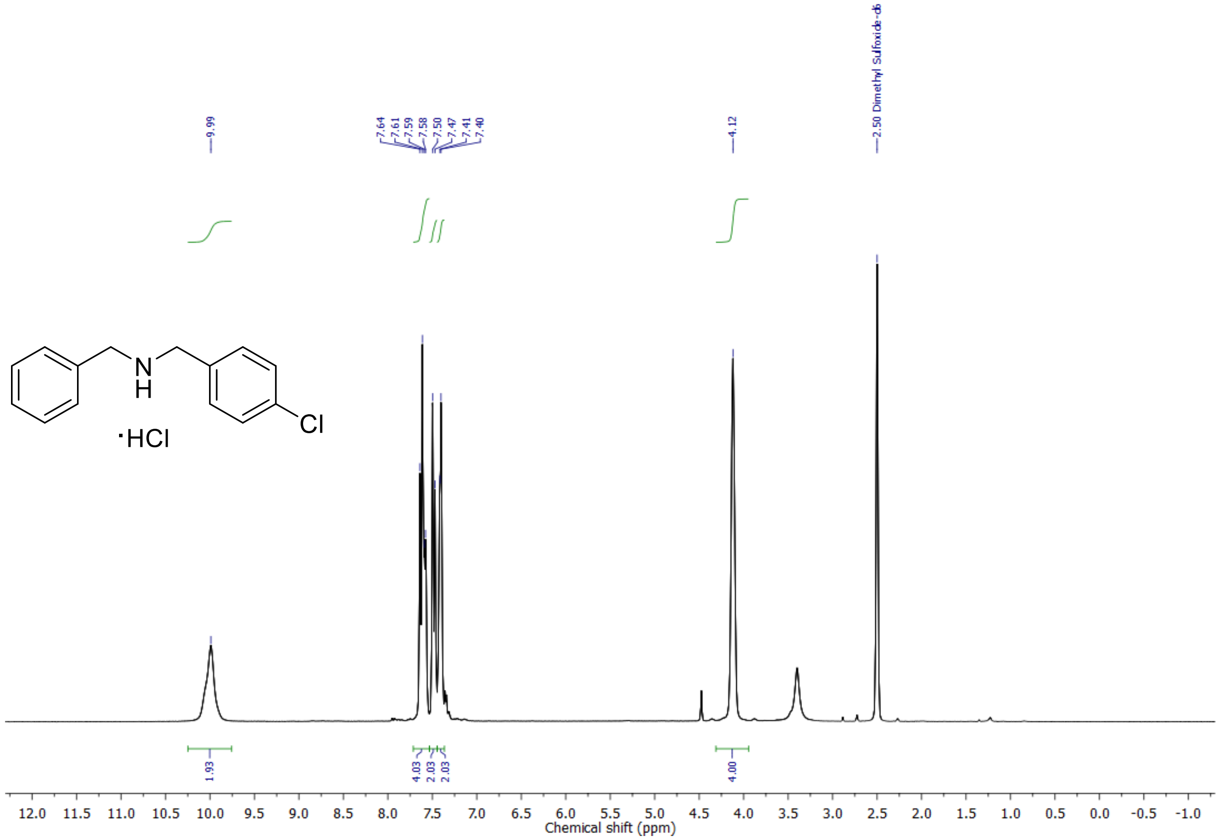

Figure S18: ^{1}H-NMR spectrum of **5**.

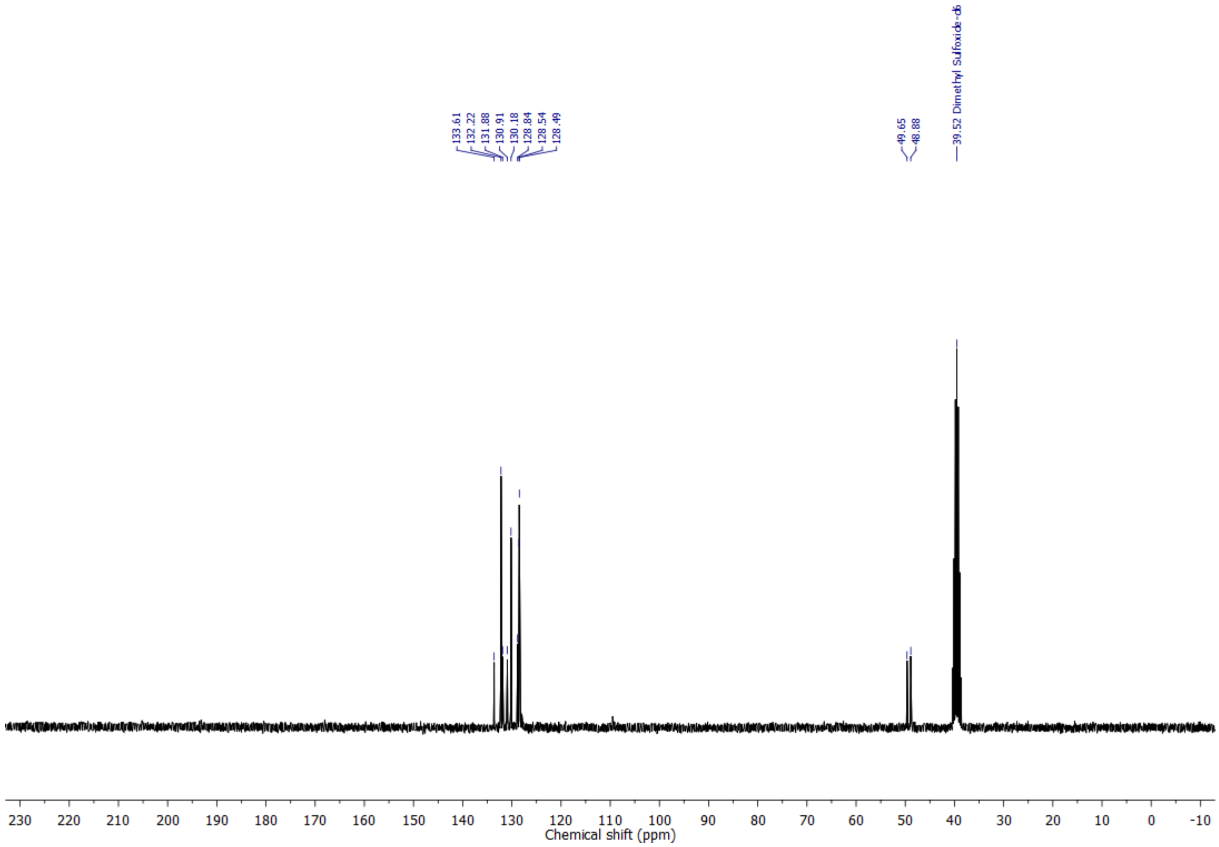

Figure S19: ^{13}C-NMR spectrum of **5**.

***N*-benz yl-1-(3-chlorophenyl)methanaminium chloride (6)**

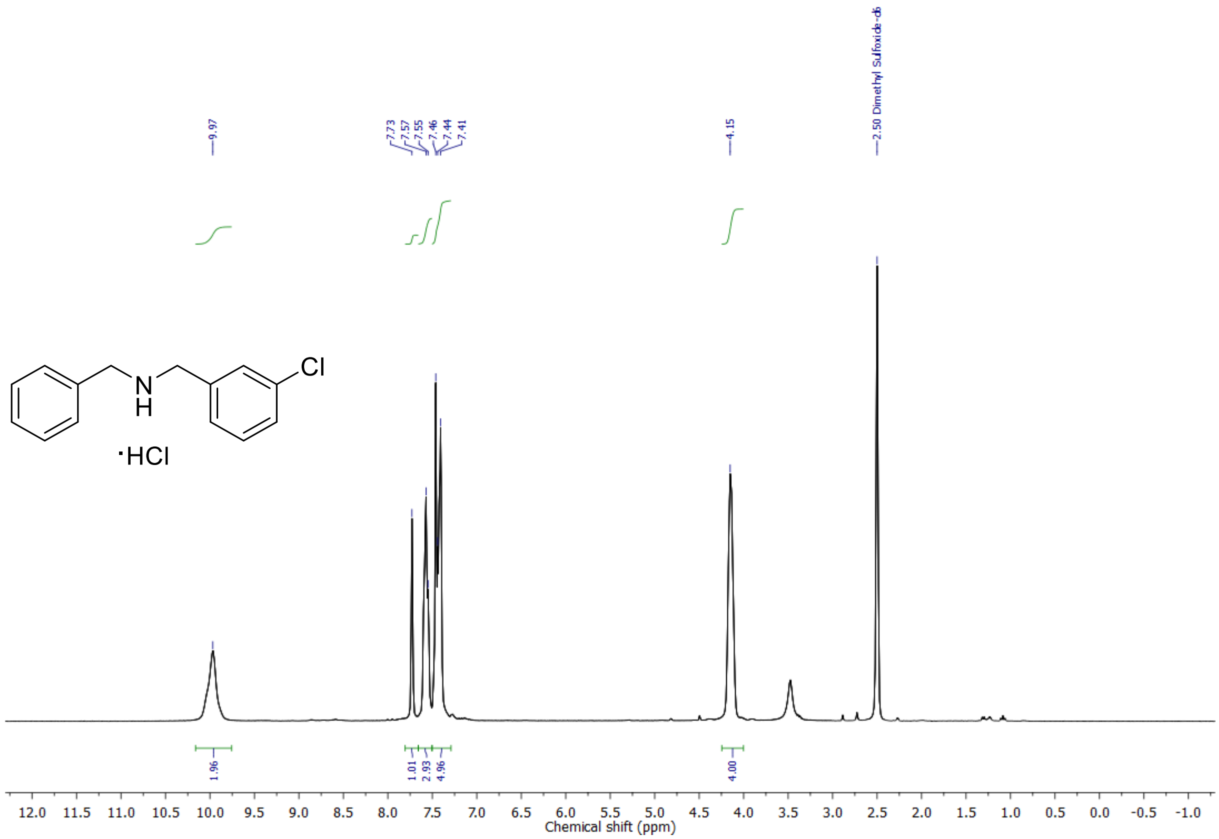

Figure S20: ^{1}H-NMR spectrum of **6**.

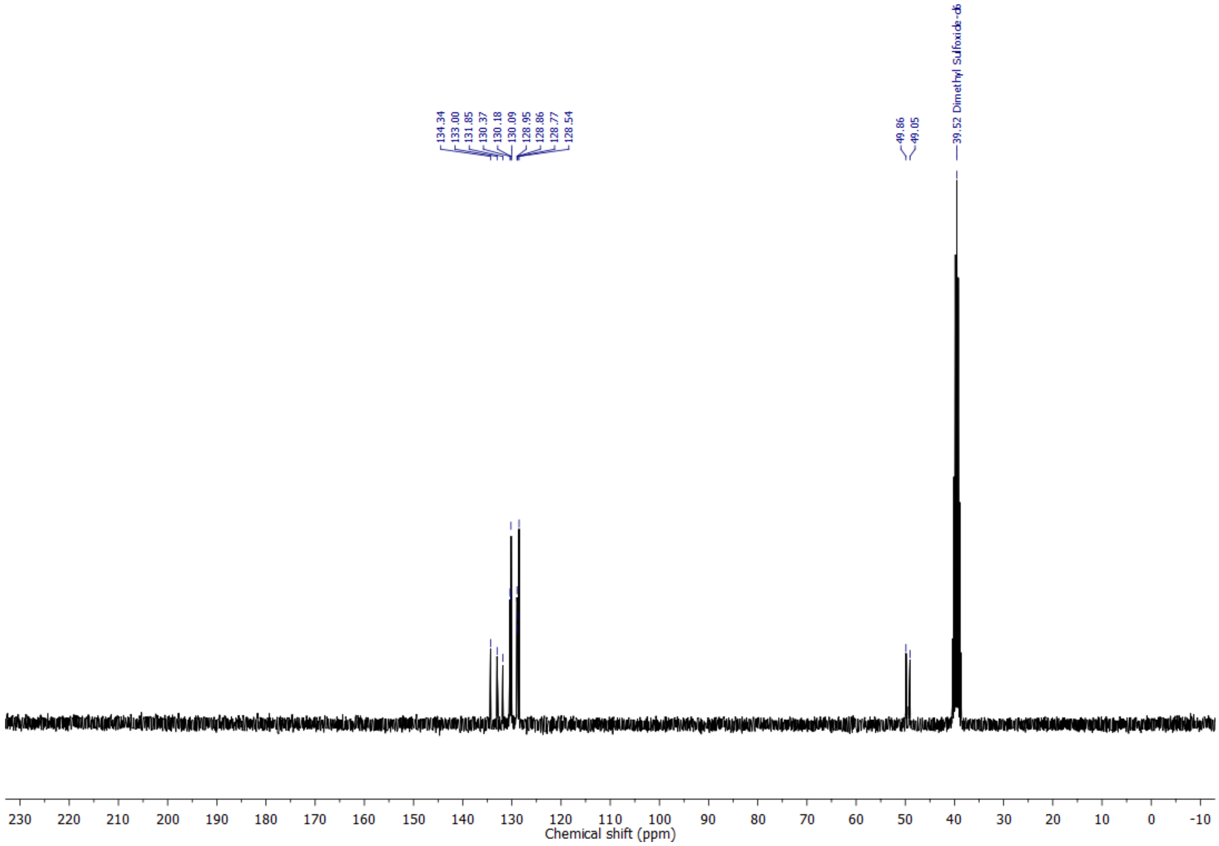

Figure S21: ^{13}C-NMR spectrum of **6**.

N-benzyl-1-(2-chlorophenyl)methanaminium chloride (7)

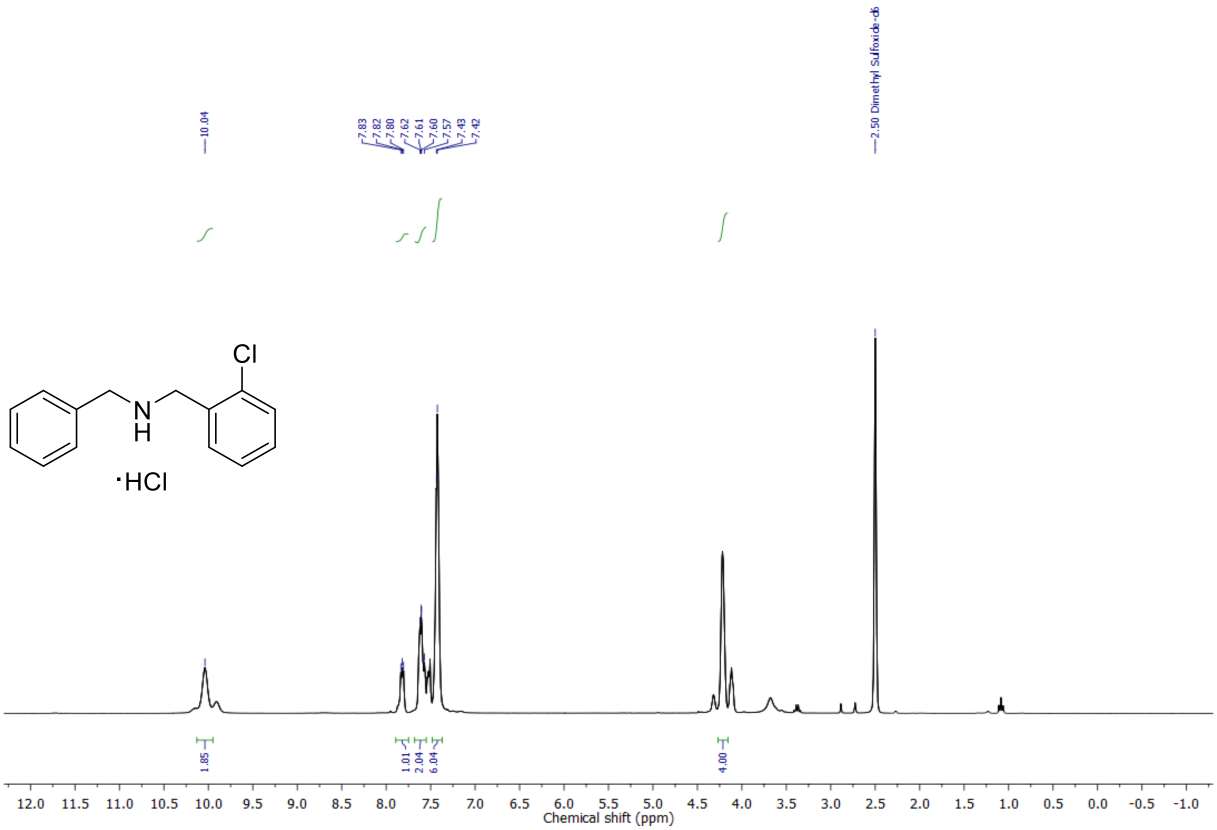

Figure S22: ^{1}H-NMR spectrum of **7**.

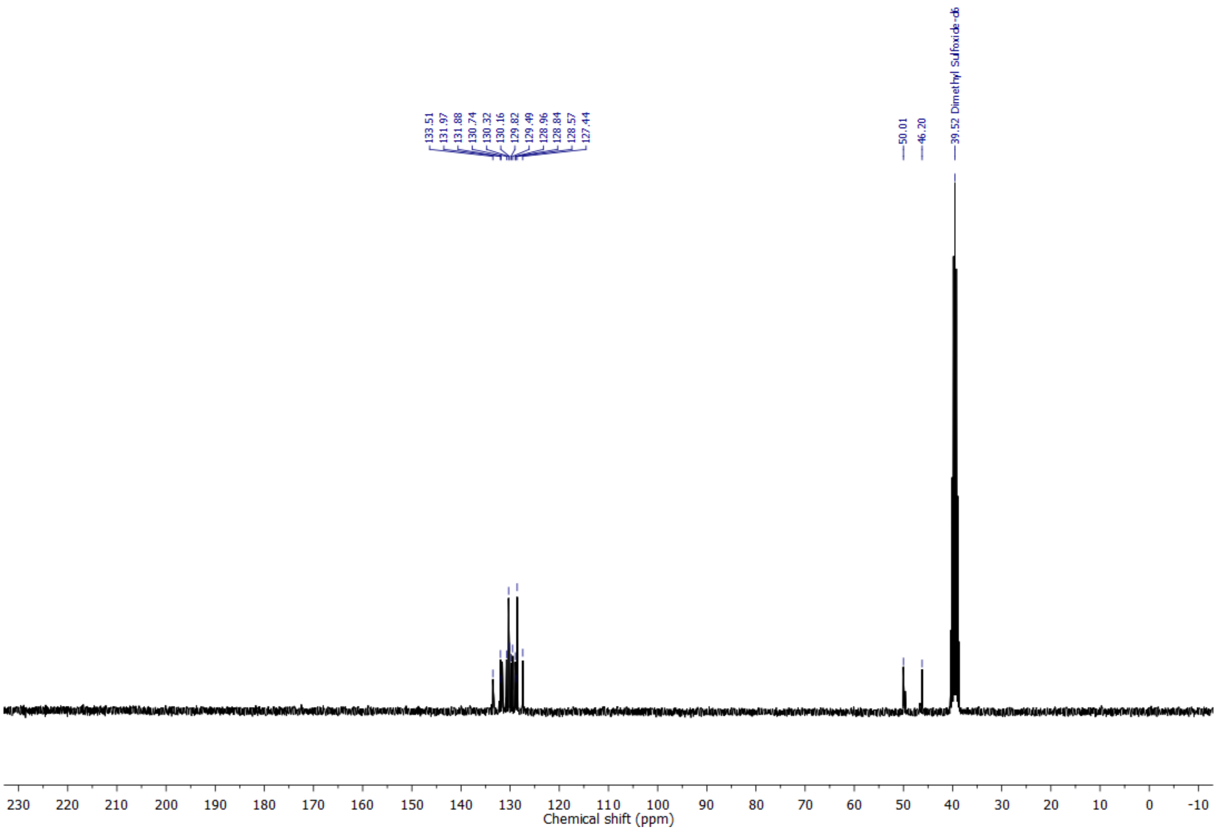

Figure S23: ^{13}C-NMR spectrum of **7**.

N-benzyl-1-(4-bromophenyl)methanaminium chloride (8)

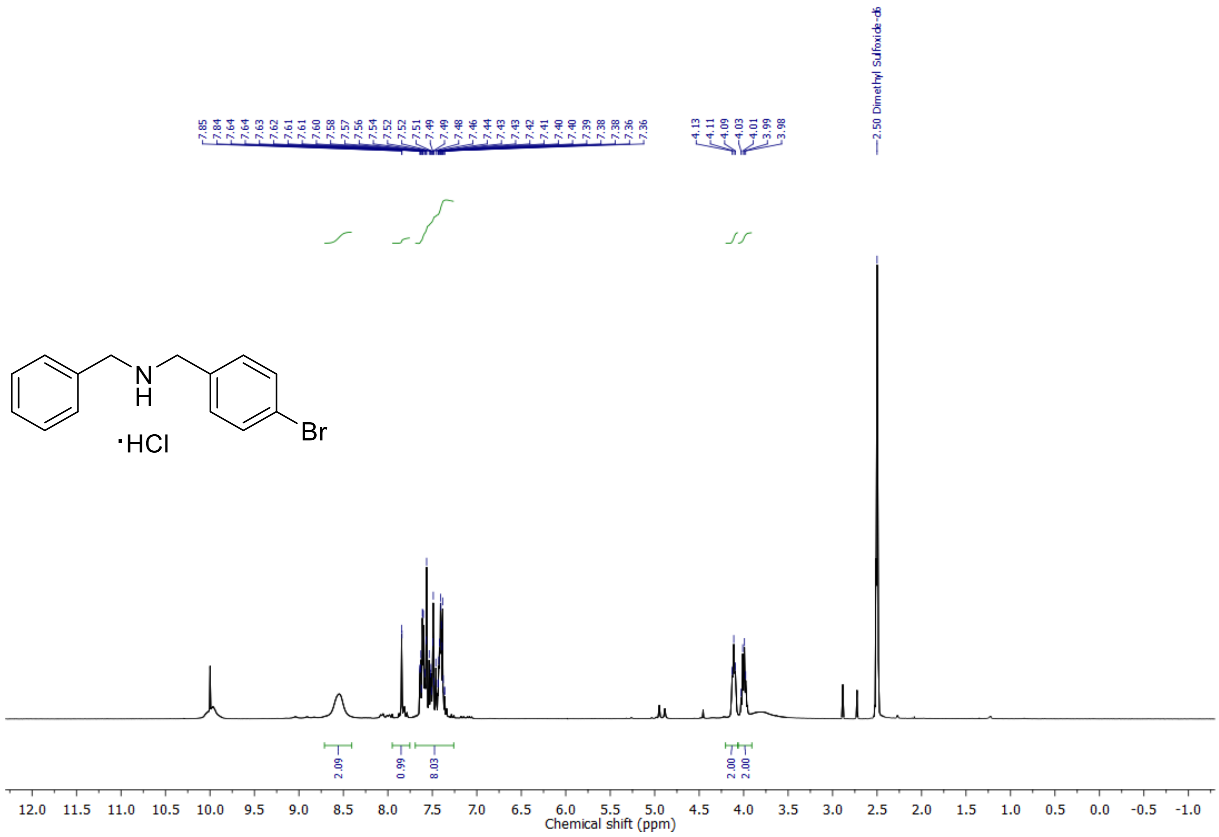

Figure S24: ^{1}H-NMR spectrum of **8**.

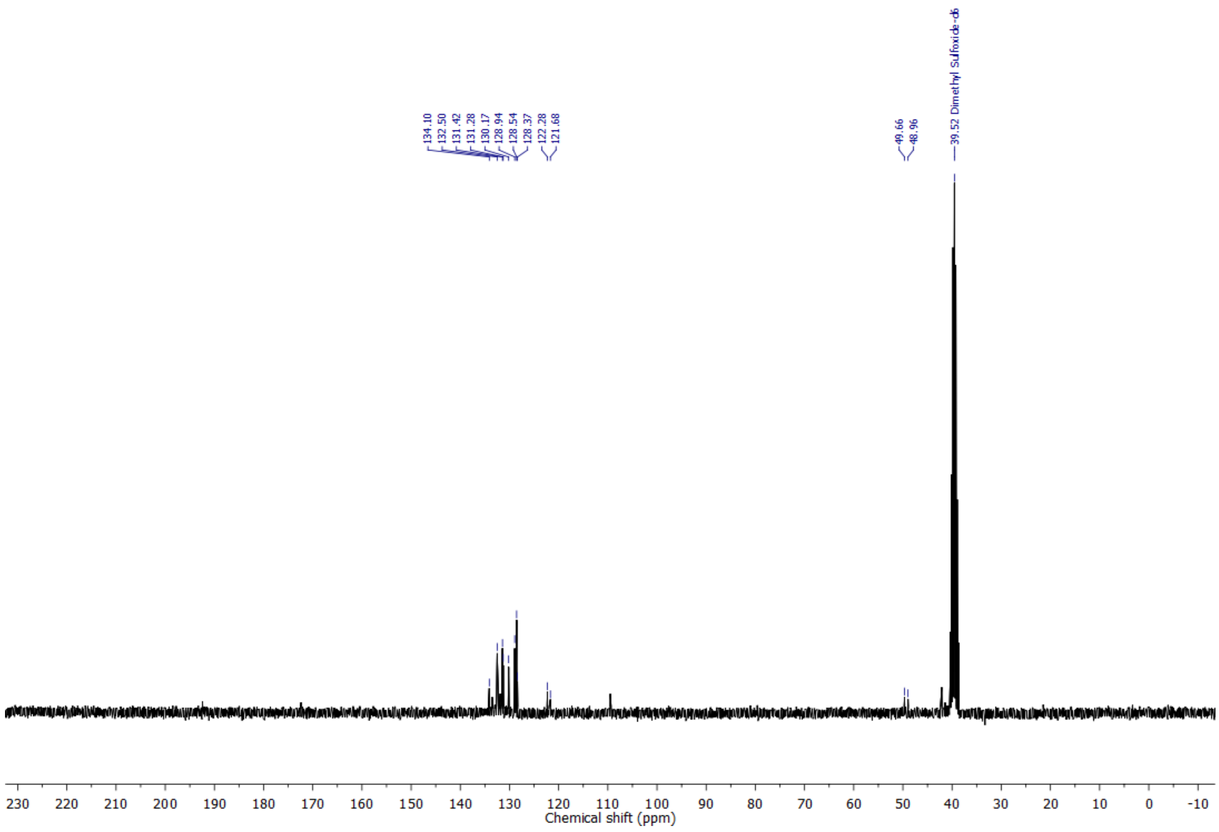

Figure S25: ^{13}C-NMR spectrum of **8**.

***N*-benzyl-1-(4-(tert-butyl)phenyl)methanaminium chloride (9)**

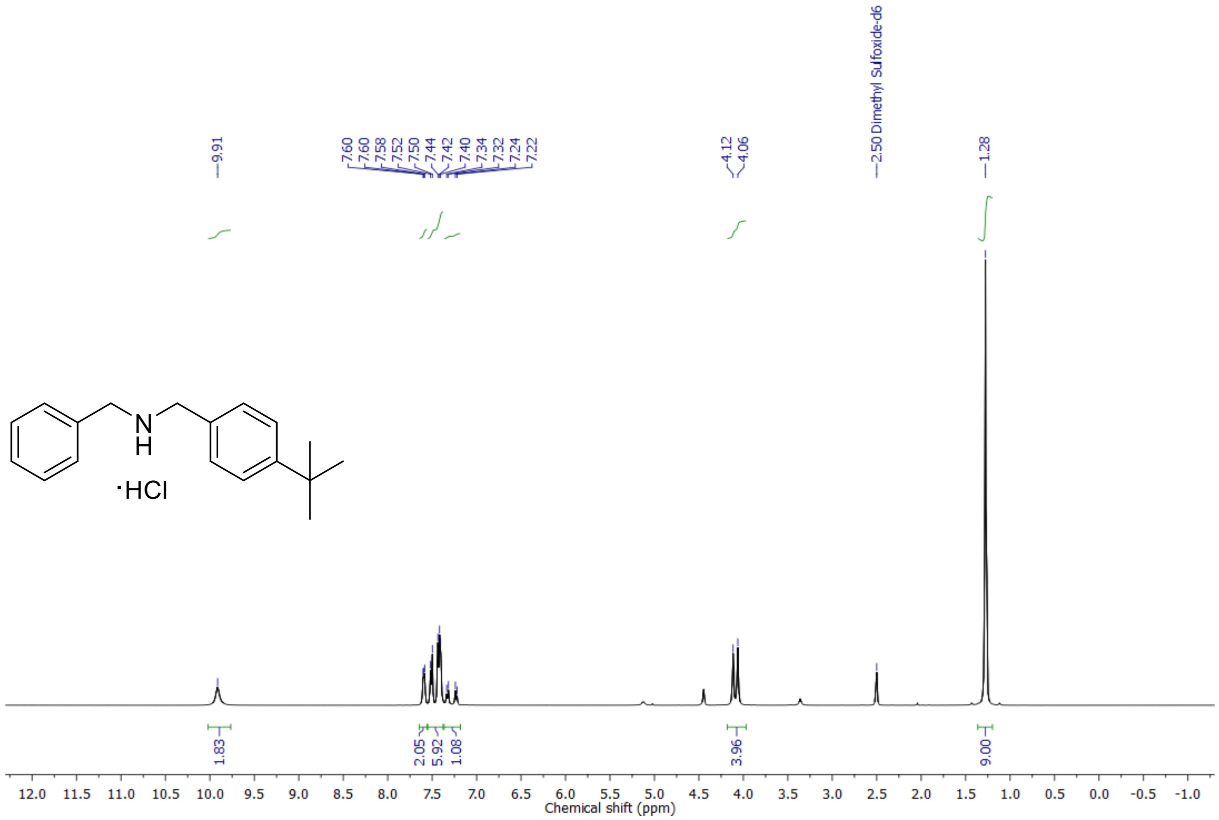

Figure S26: ^{1}H-NMR spectrum of **9**.

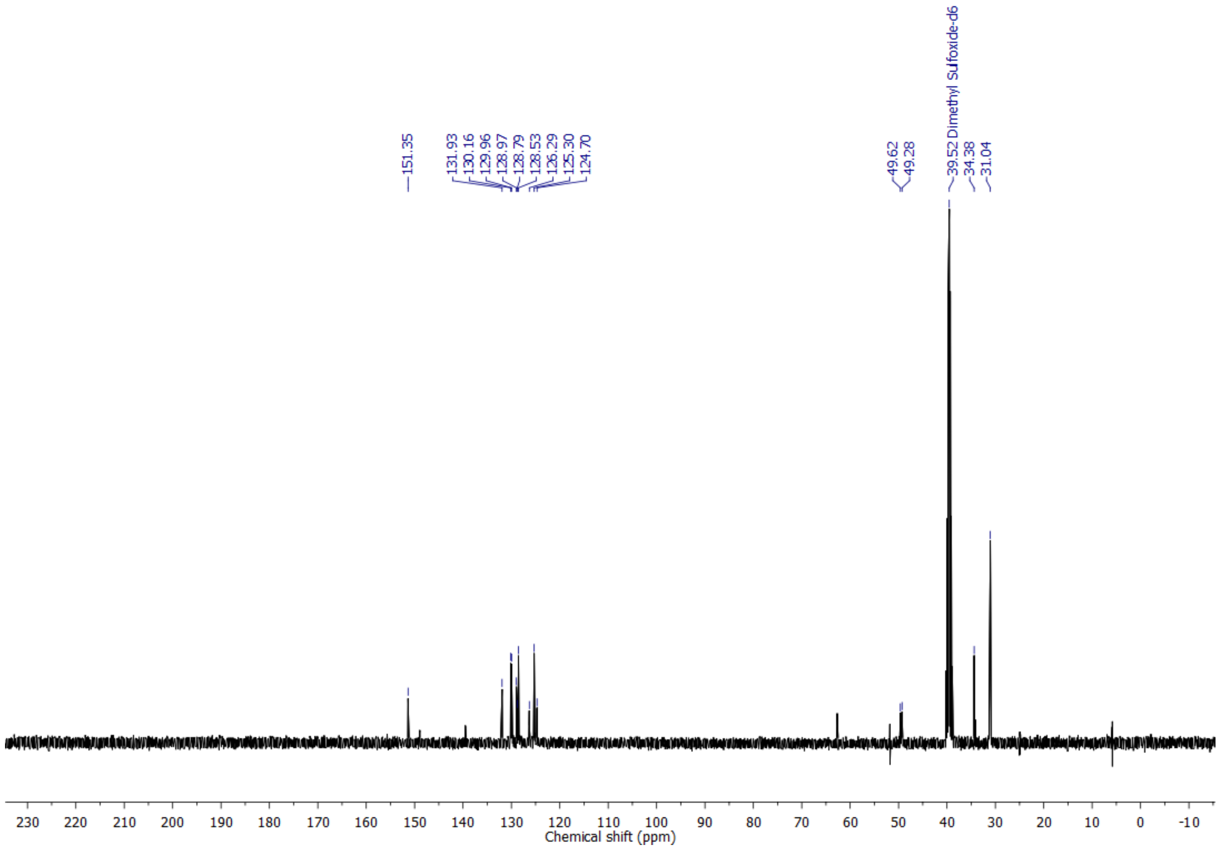

Figure S27: ^{13}C-NMR spectrum of **9**.

N-benzyl-1-(4-(4,4,5,5-tetramethyl-1,3,2-dioxaborolan-2-yl)phenyl)methanaminium chloride (10)

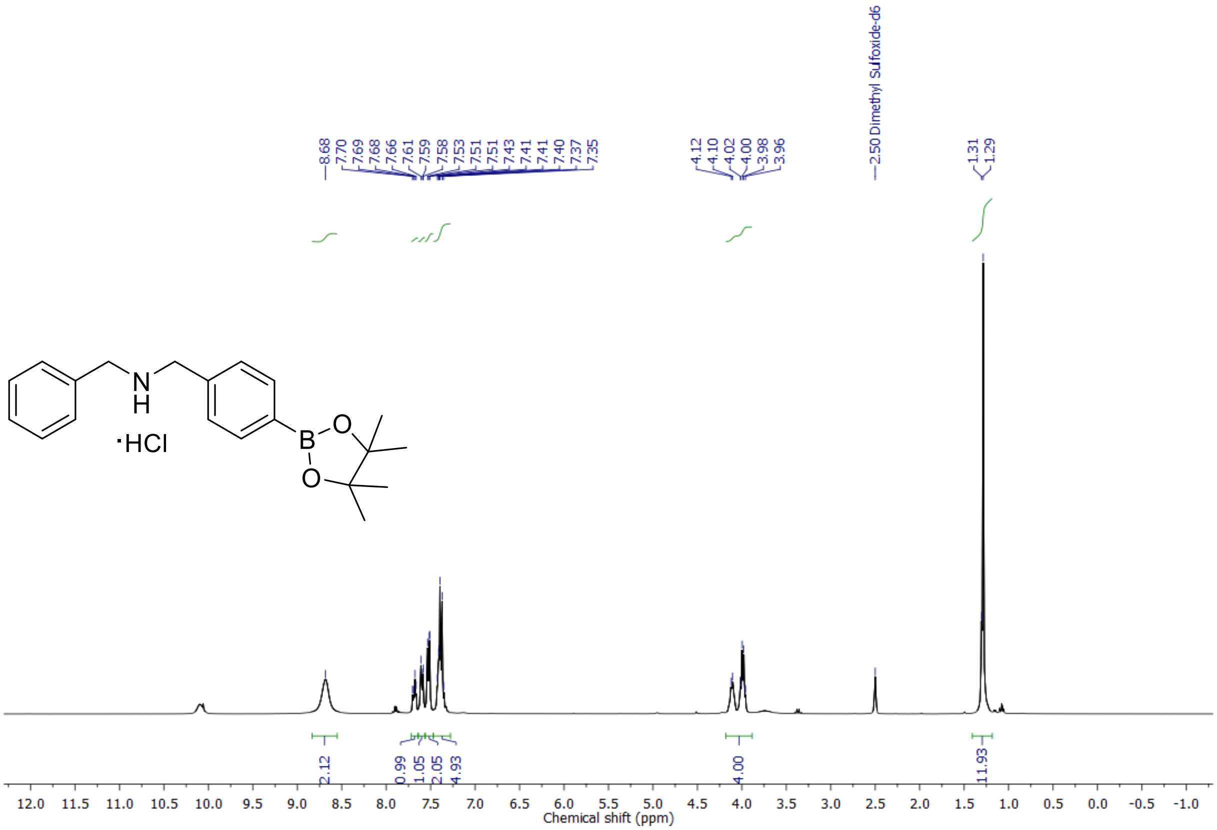

Figure S28: ^{1}H-NMR spectrum of **10**.

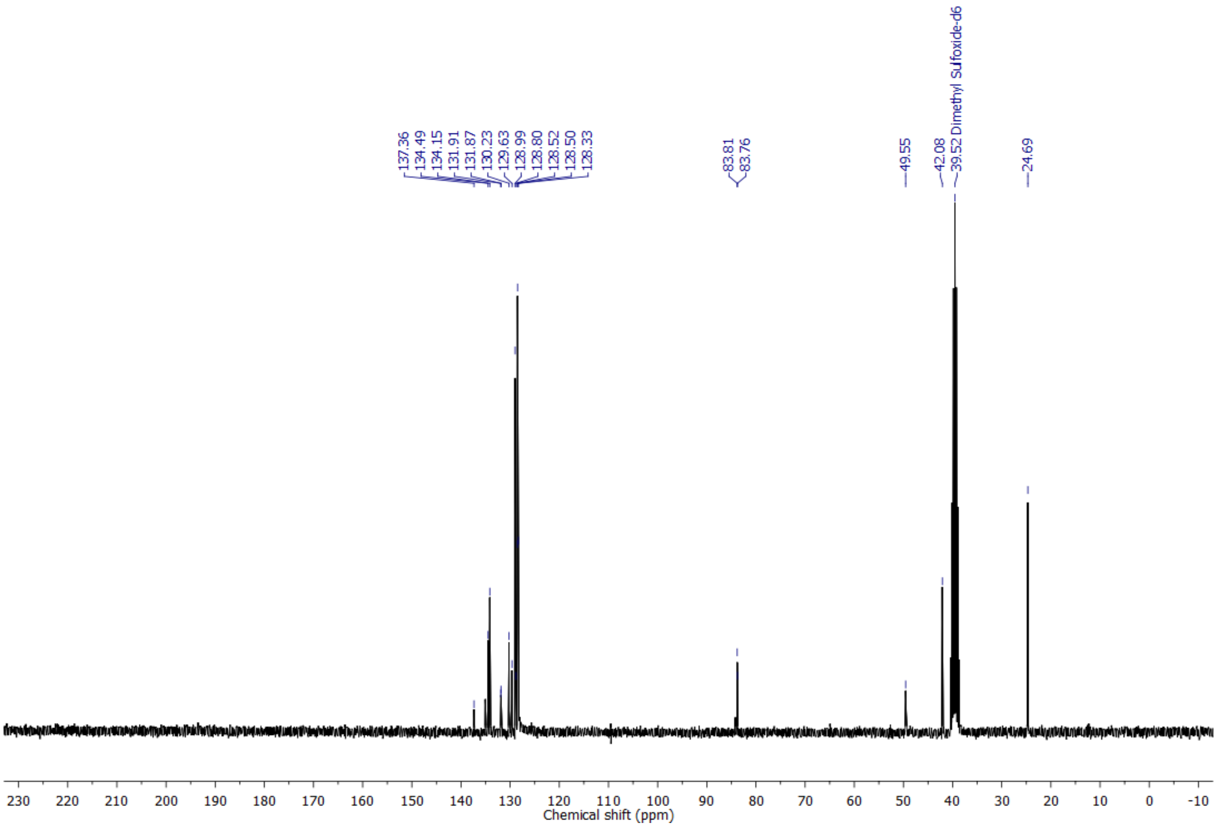

Figure S29: ^{13}C-NMR spectrum of **10**.

N-benzyl-1-(4-methoxyphenyl)methanaminium chloride (11)

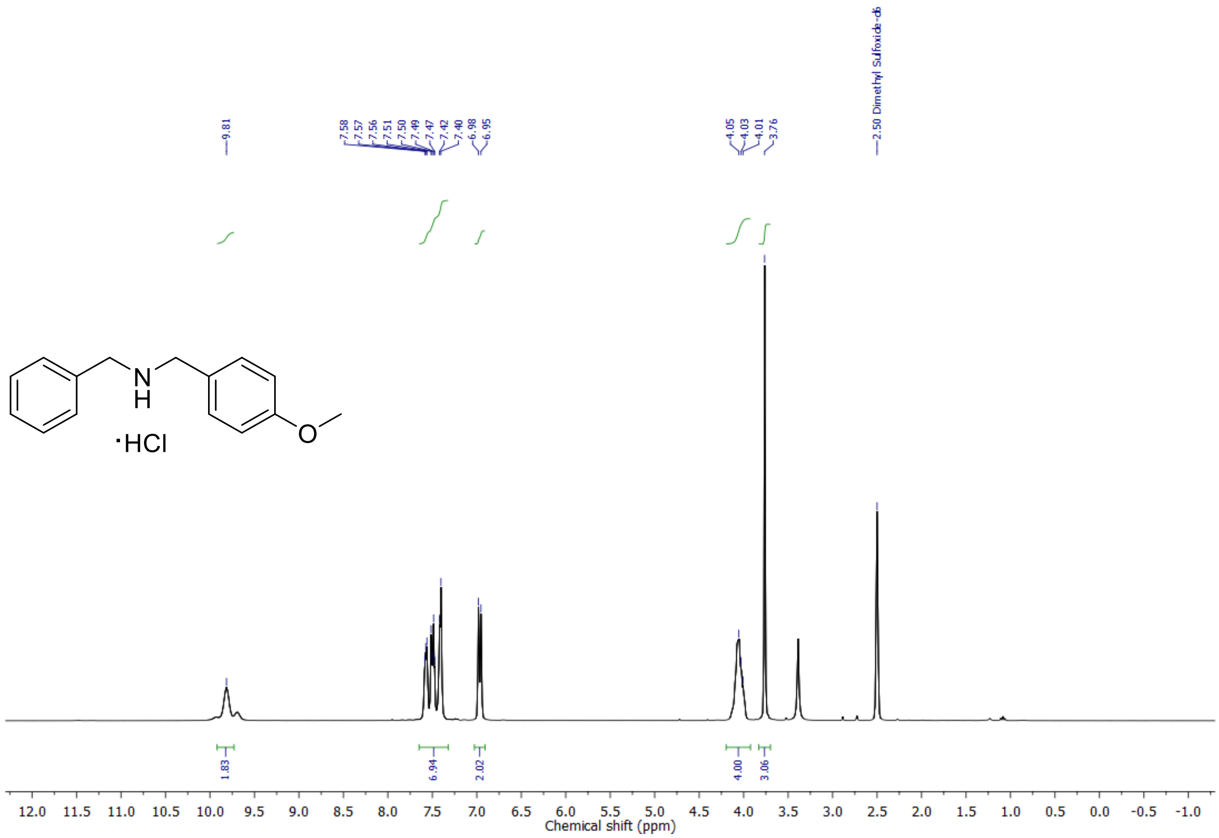

Figure S30: ^{1}H-NMR spectrum of **11**.

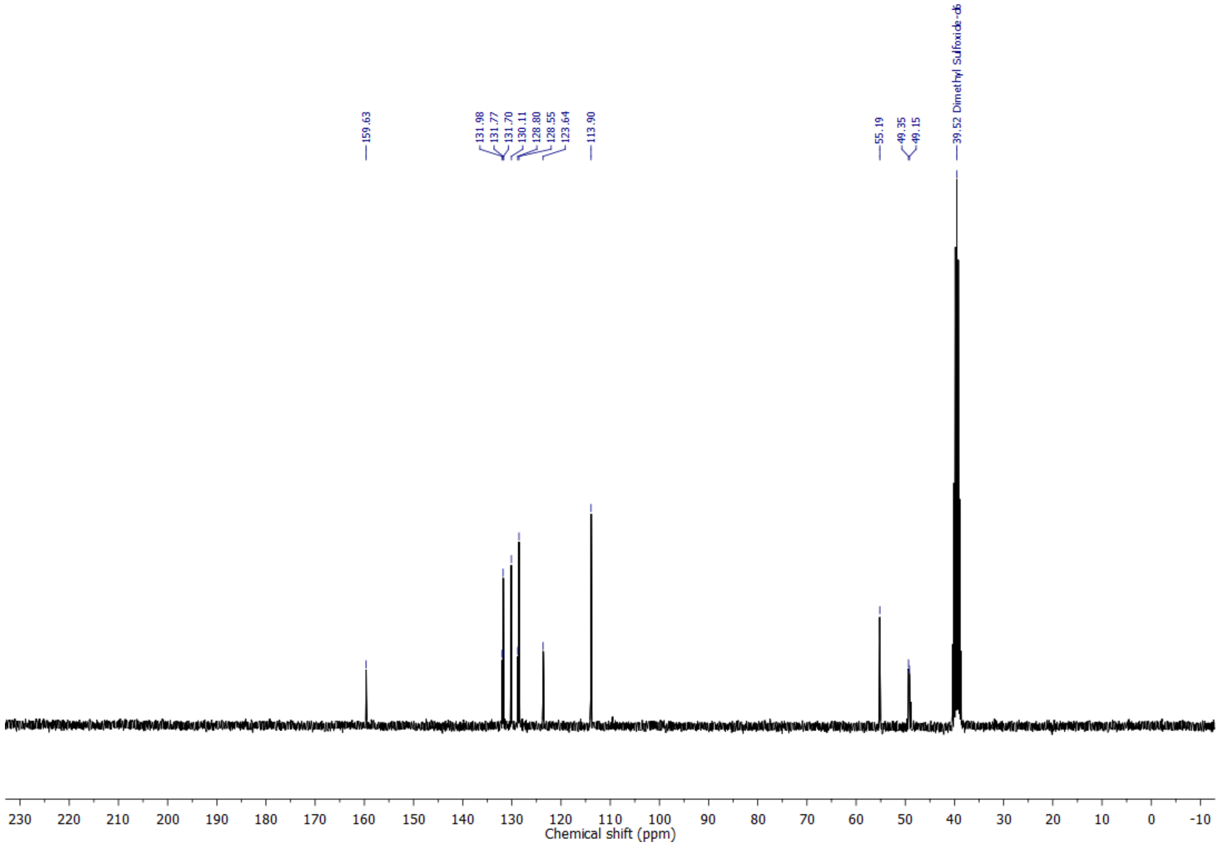

Figure S31: ^{13}C-NMR spectrum of **11**.

N-benzyl-1-(4-(benzyloxy)phenyl)methanaminium chloride (12)

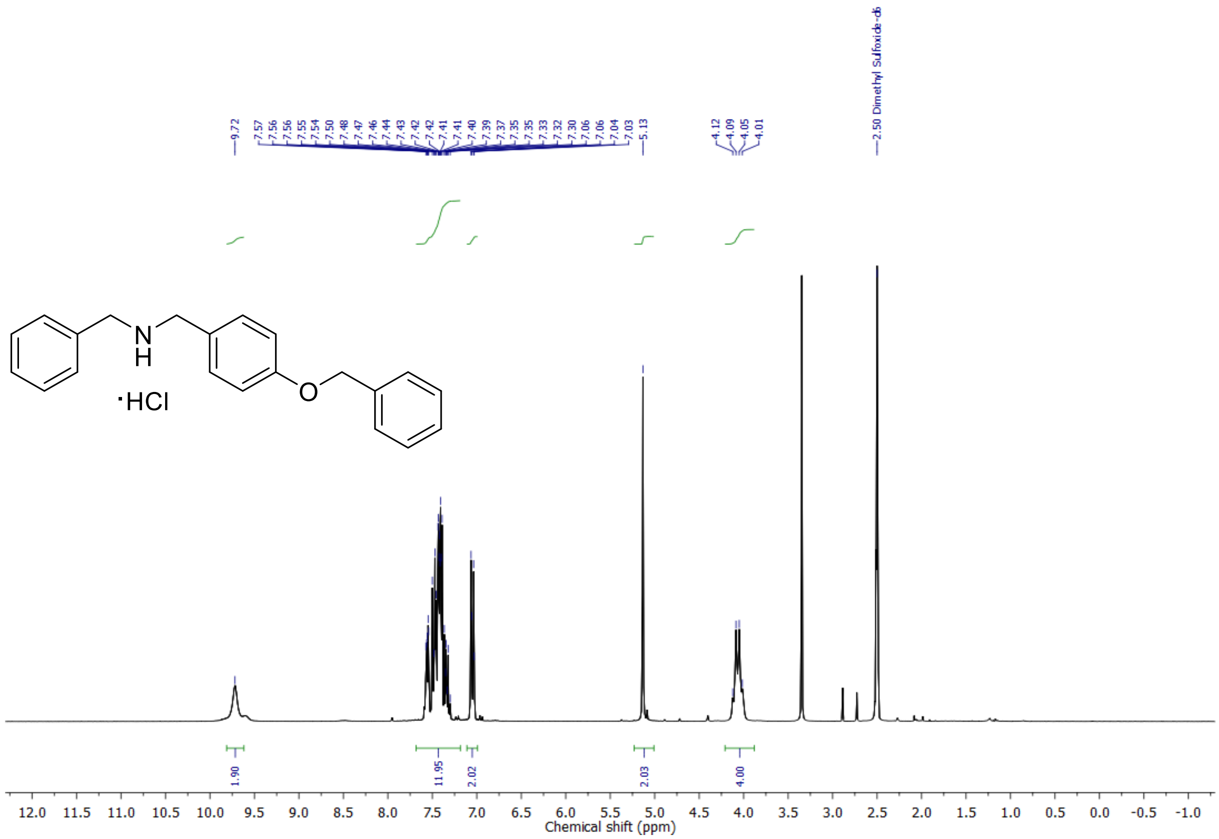

Figure S32: ^{1}H-NMR spectrum of **12**.

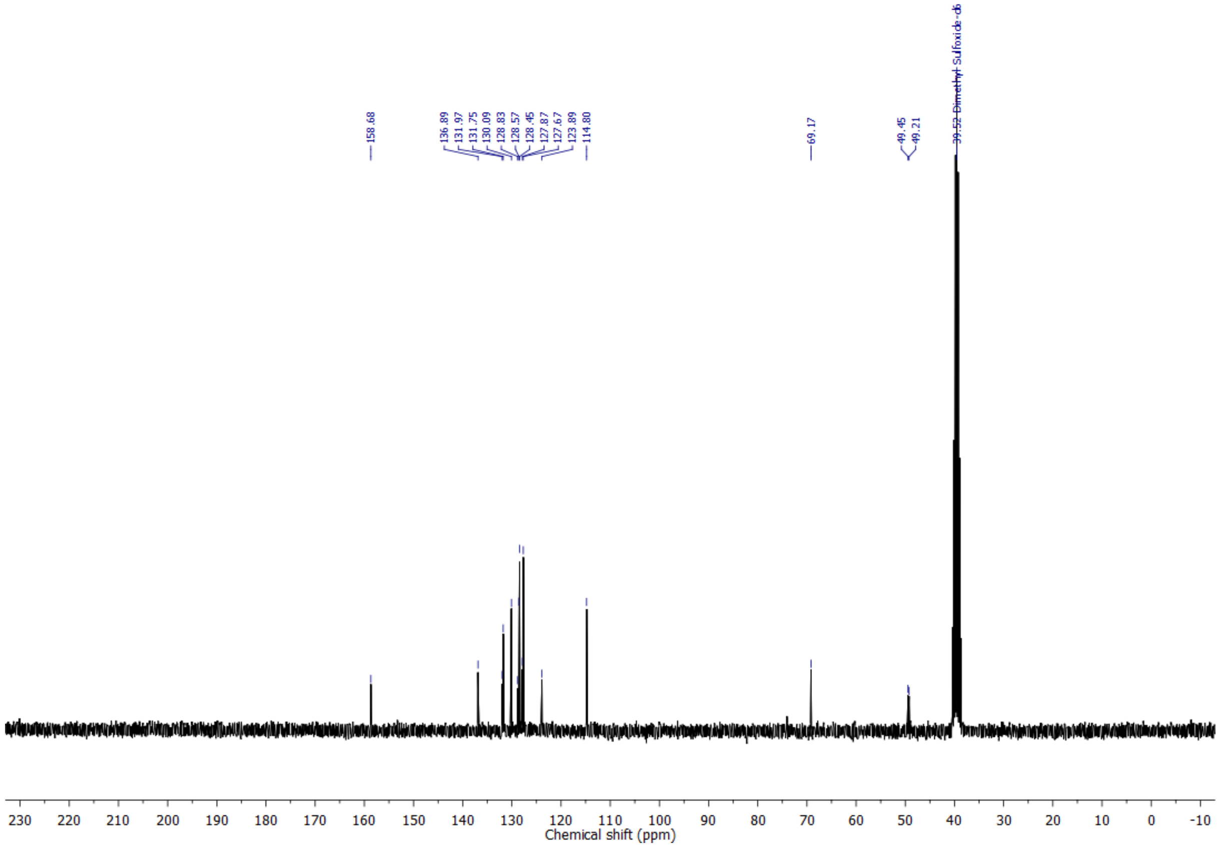

Figure S33: ^{13}C-NMR spectrum of **12**.

1-(benzo[d][1,3]dioxol-5-yl)-*N*-benzylmethanaminium chloride (13)

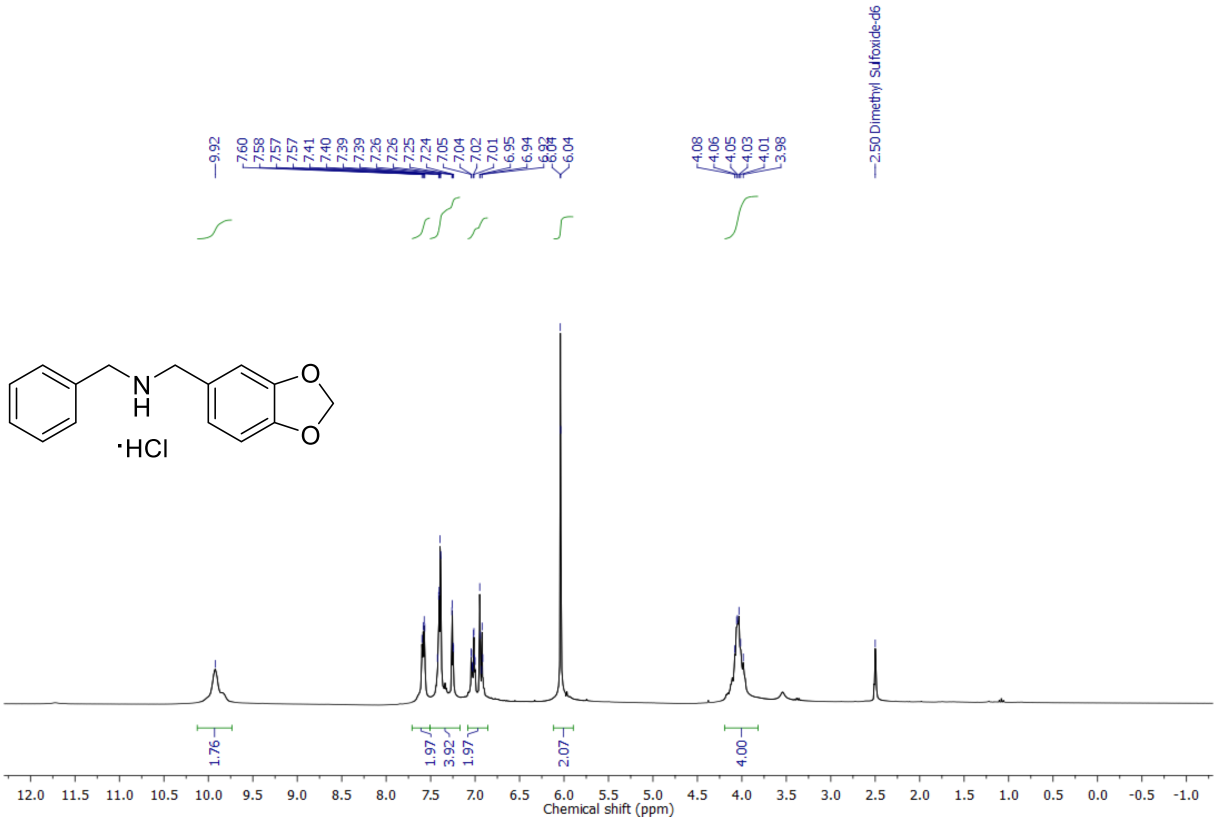

Figure S34: ^{1}H-NMR spectrum of **13**.

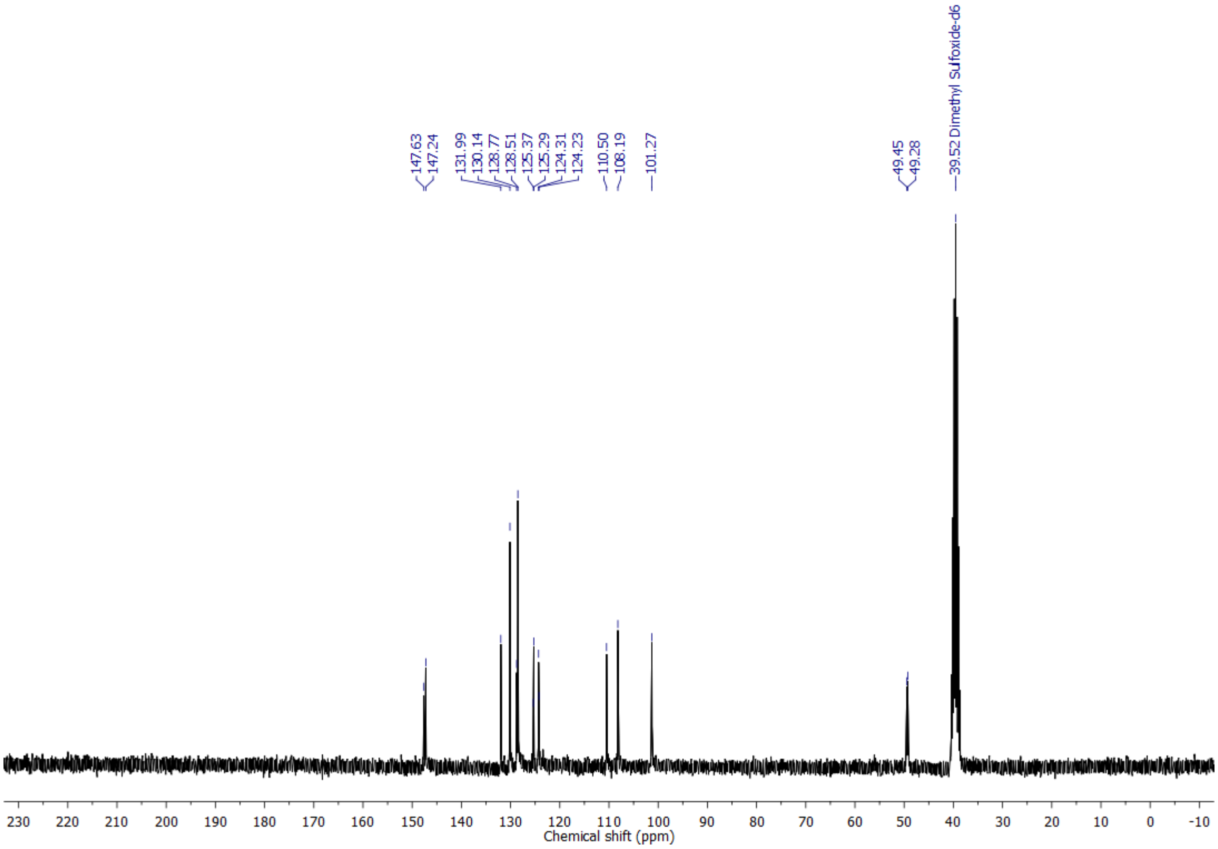

Figure S35: ^{13}C-NMR spectrum of **13**.

***N*-benzylpentan-1-aminium chloride (14)**

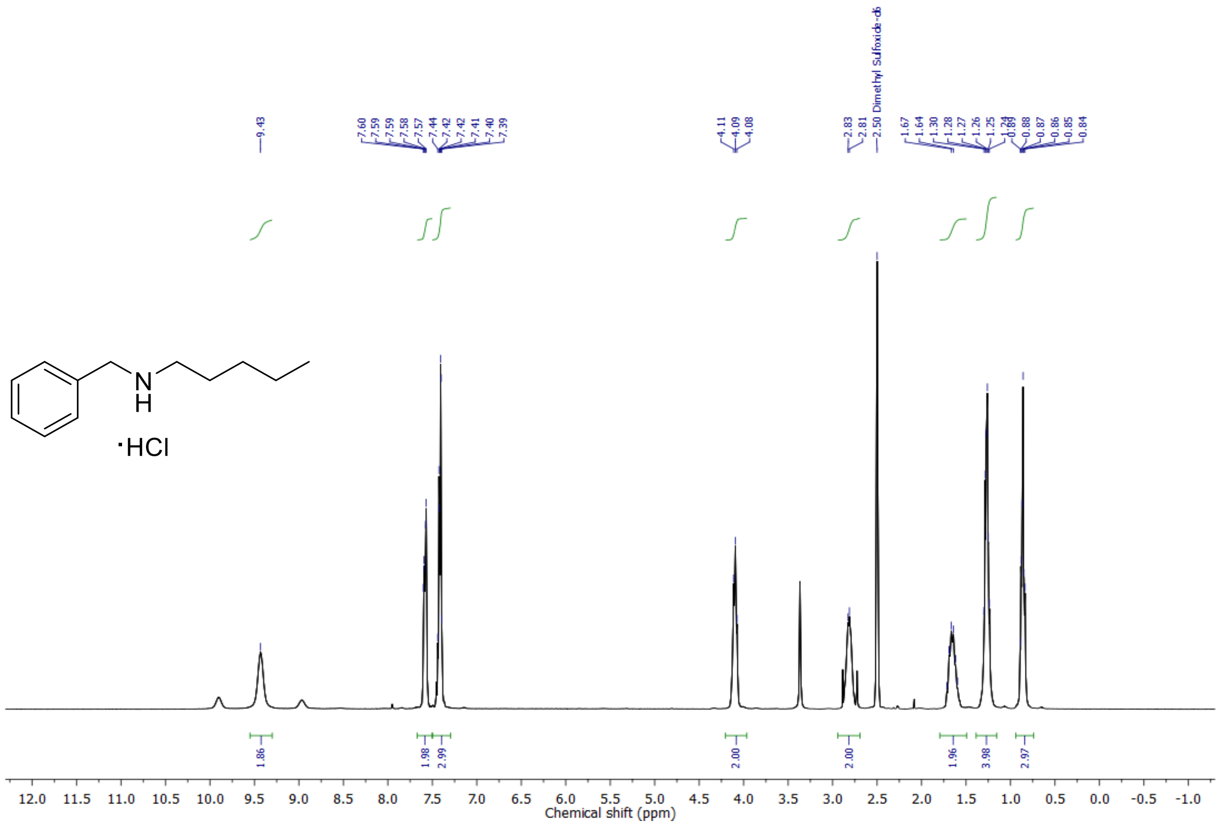

Figure S36: ^{1}H-NMR spectrum of **14**.

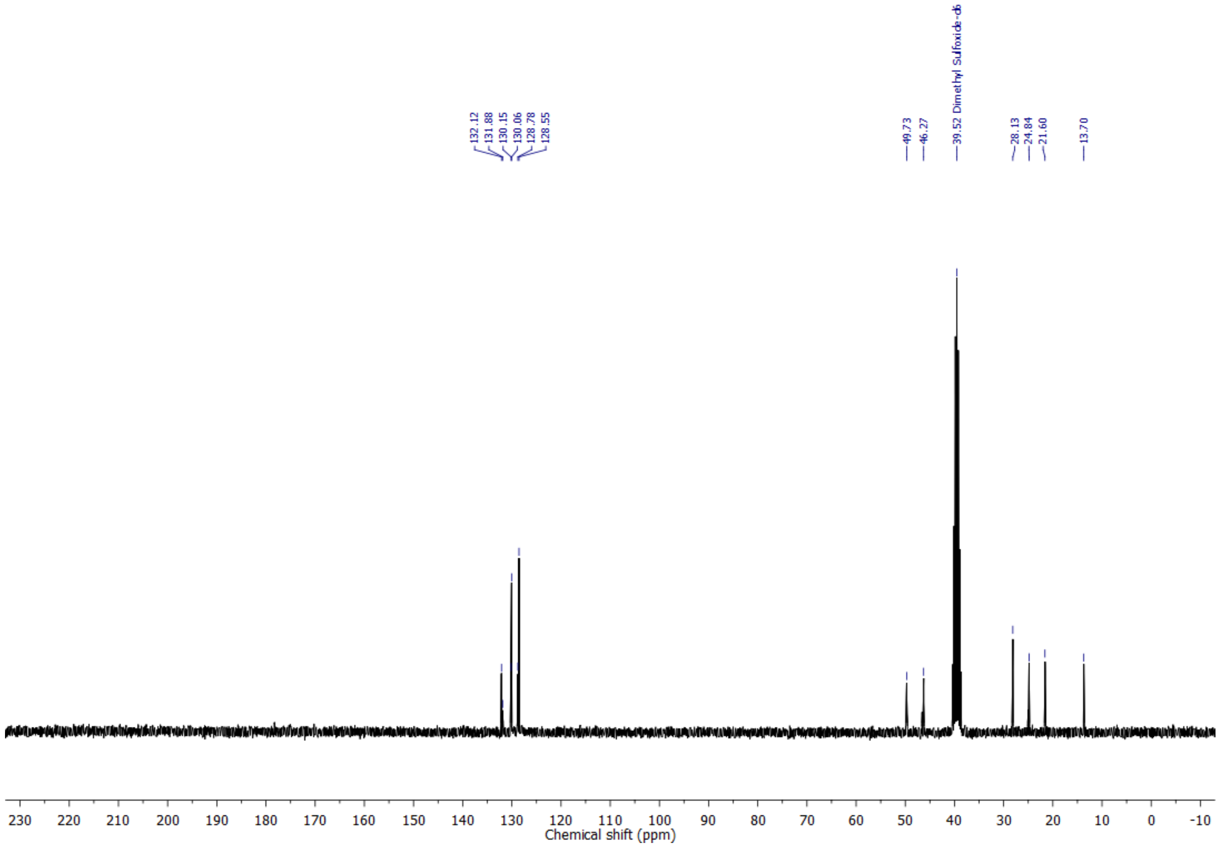

Figure S37: ^{13}C-NMR spectrum of **14**.

N-benzylheptan-1-aminium chloride (15)

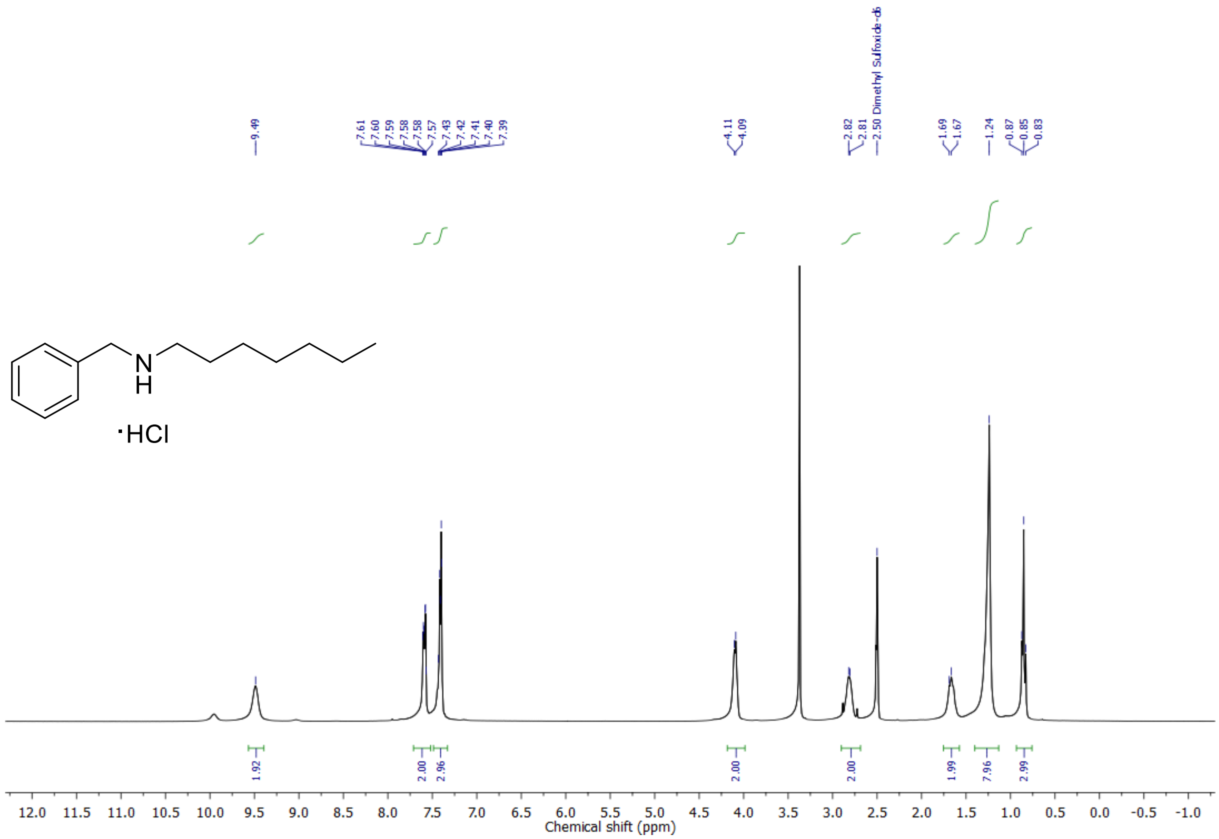

Figure S38: ^{1}H-NMR spectrum of **15**.

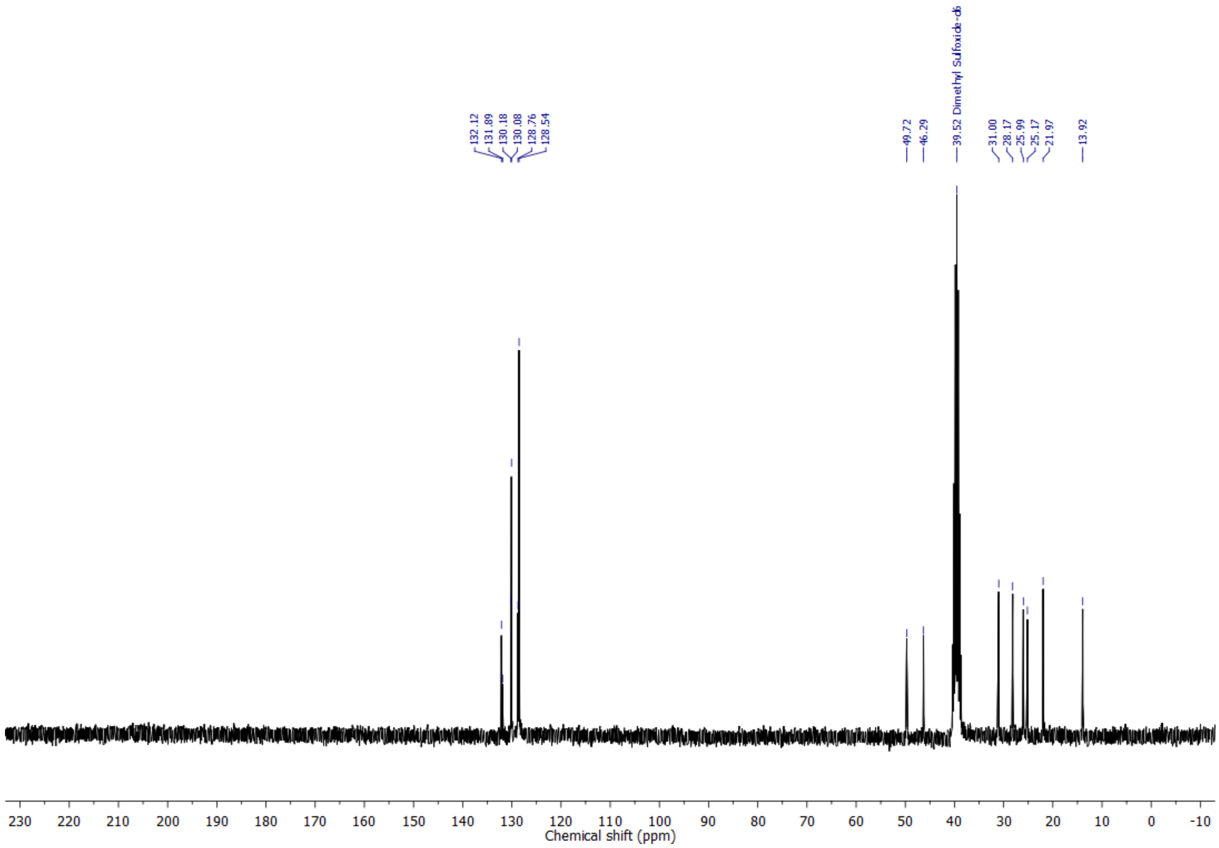

Figure S39: ^{13}C-NMR spectrum of **15**.

***N*-benzyldecan-1-aminium chloride (16)**

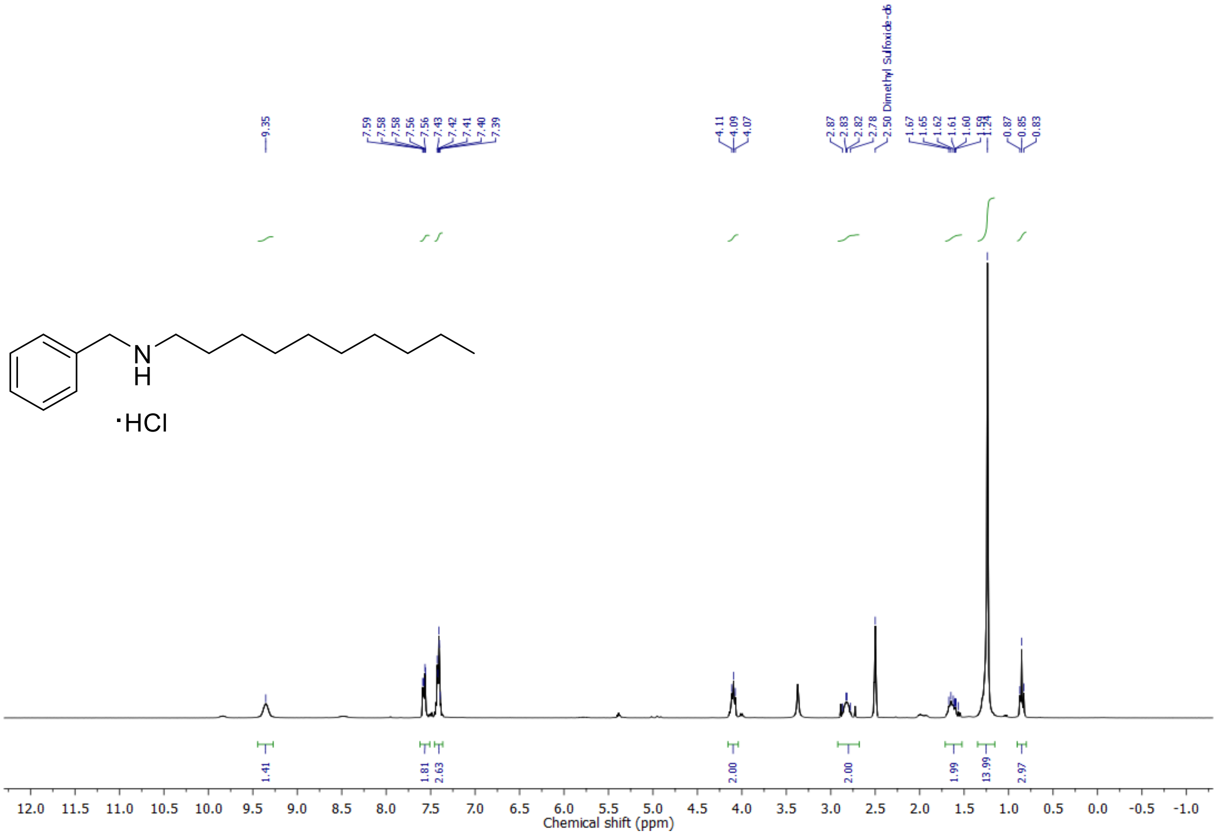

Figure S40: ^{1}H-NMR spectrum of **16**.

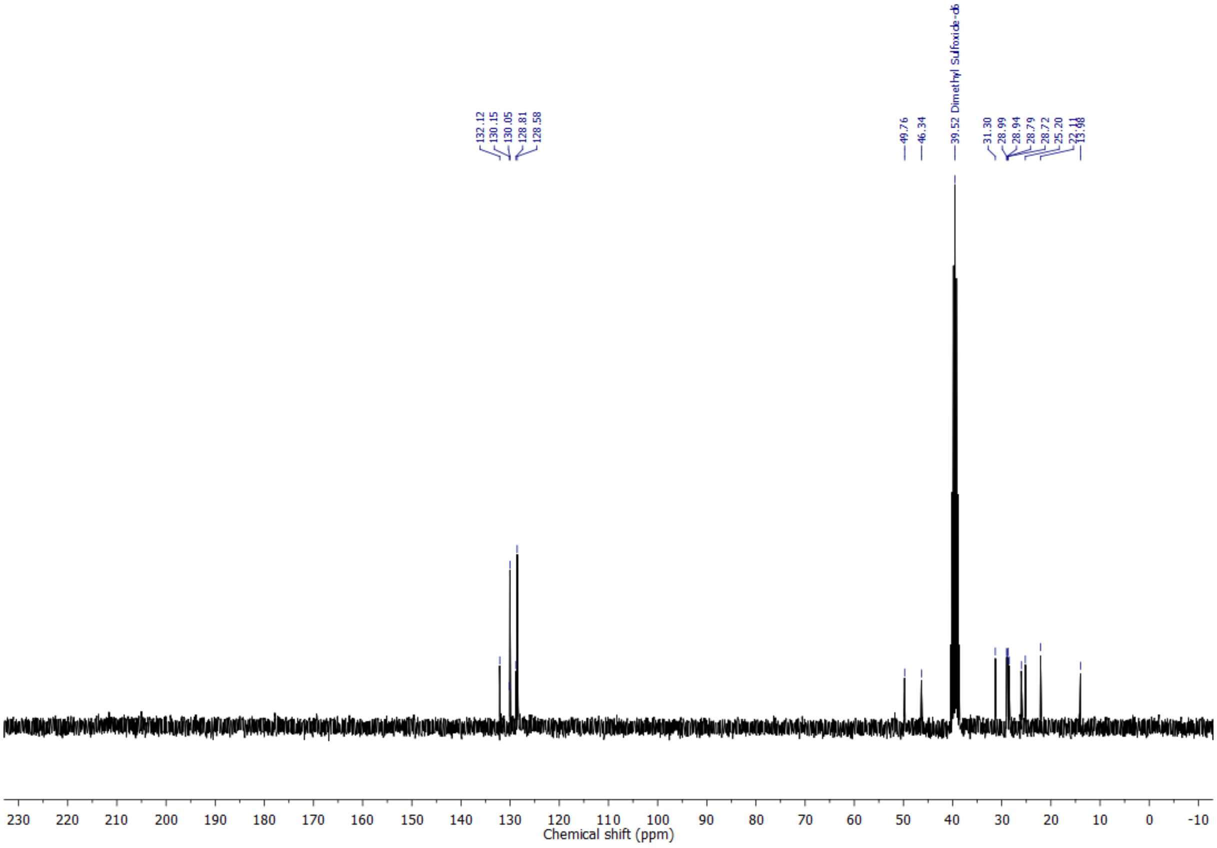

Figure S41: ^{13}C-NMR spectrum of **16**.

N-benzyldodecan-1-aminium chloride (17)

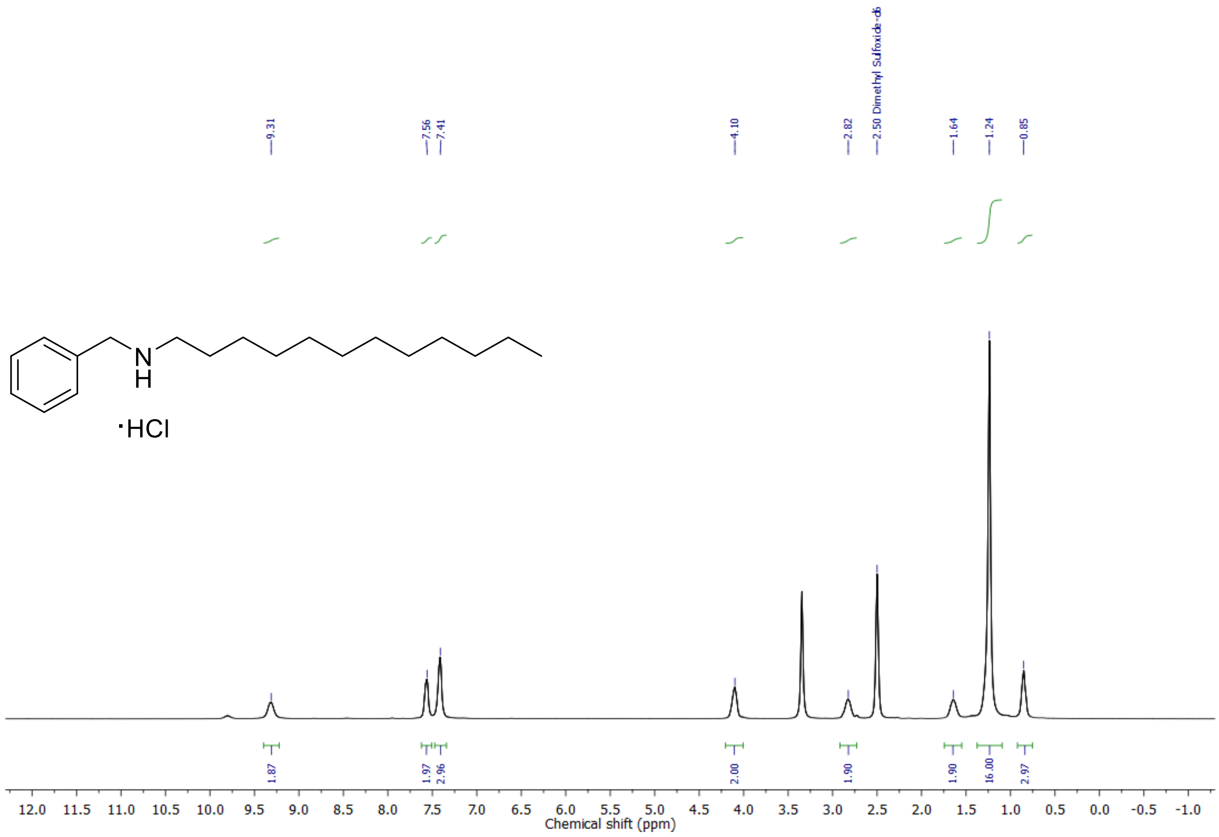

Figure S42: ^{1}H-NMR spectrum of **17**.

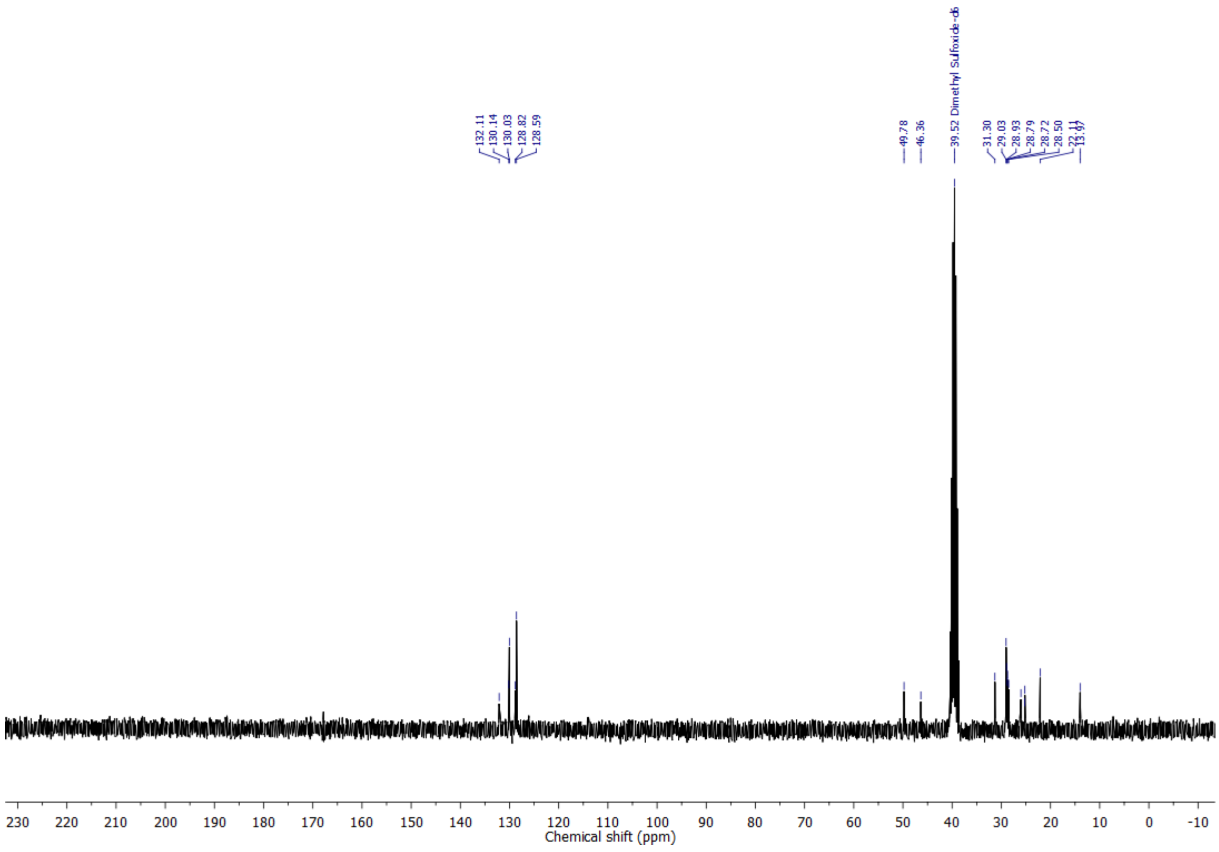

Figure S43: ^{13}C-NMR spectrum of **17**.

N-benzyl-3,7-dimethyloct-6-en-1-aminium chloride (18)

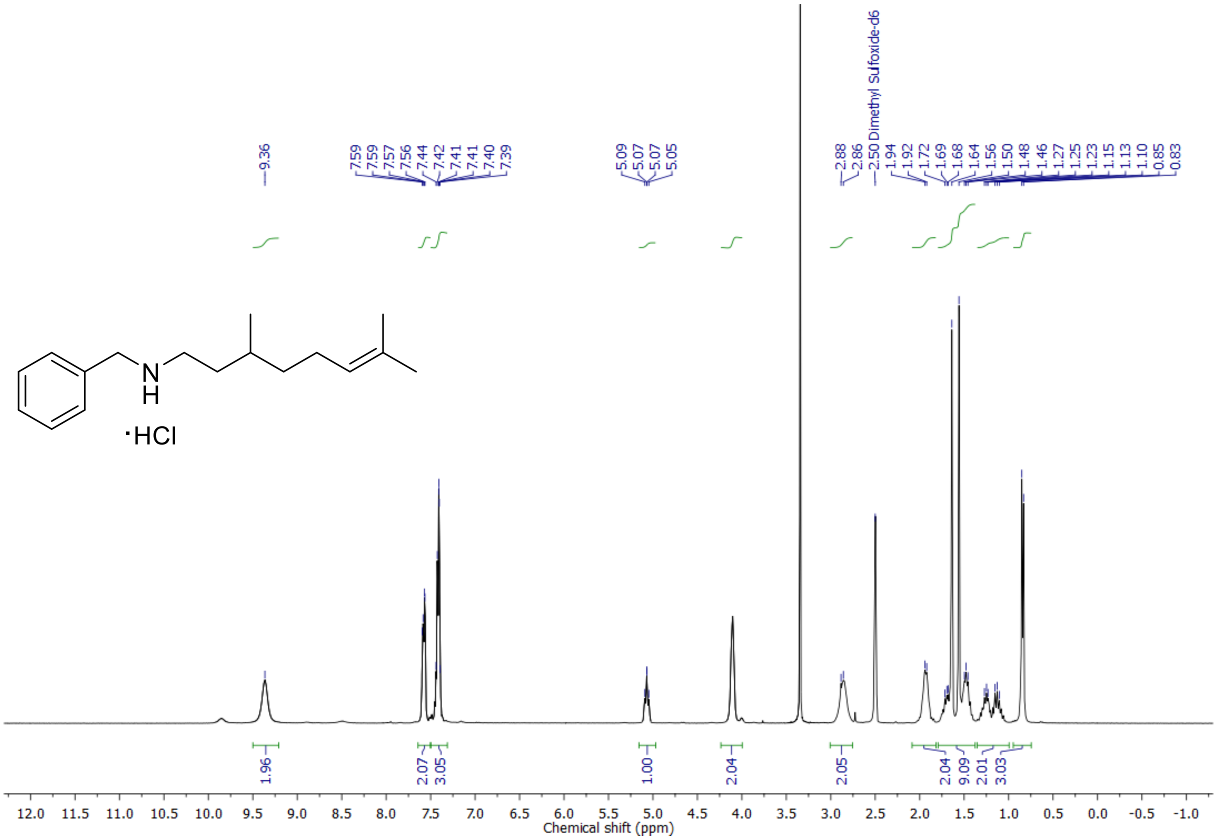

Figure S44: ^{1}H-NMR spectrum of **18**.

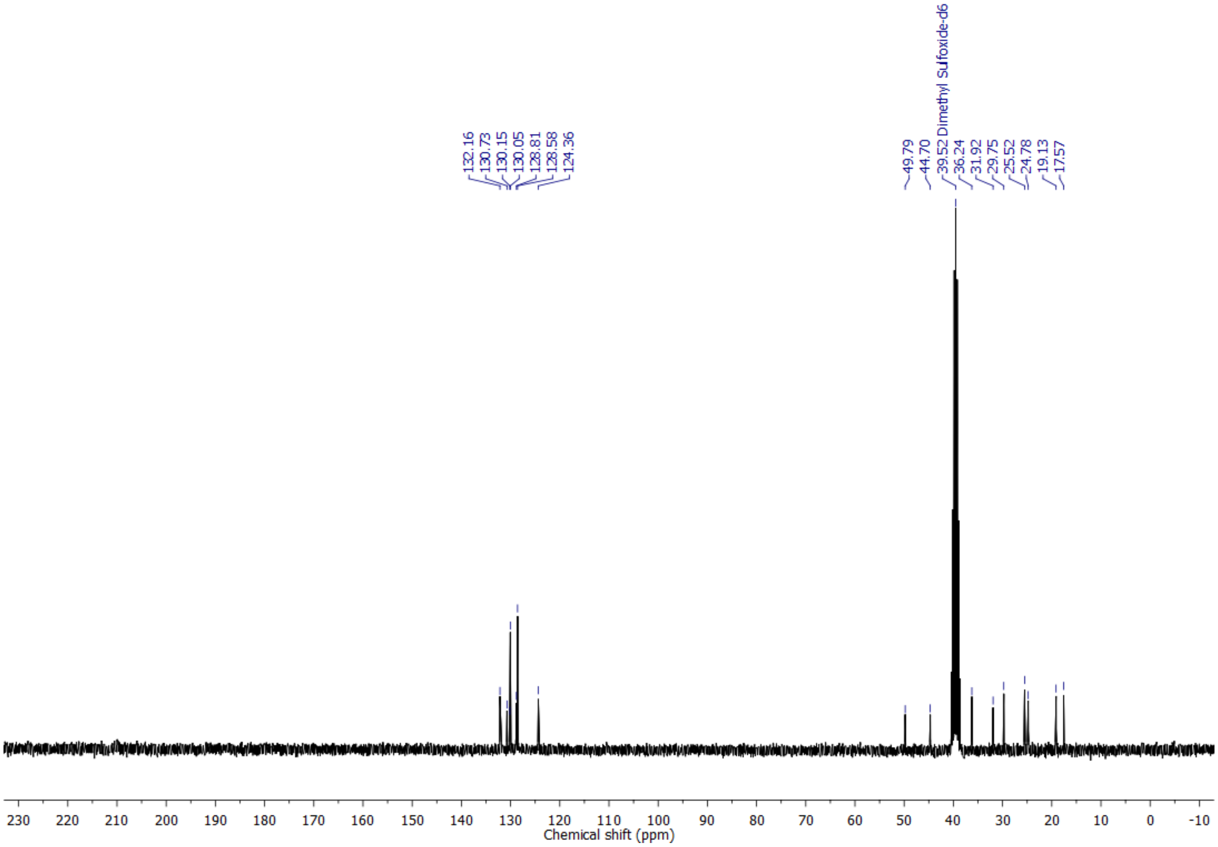

Figure S45: ^{13}C-NMR spectrum of **18**.

N-benzyl-1-cyclopropylmethanaminium chloride (19)

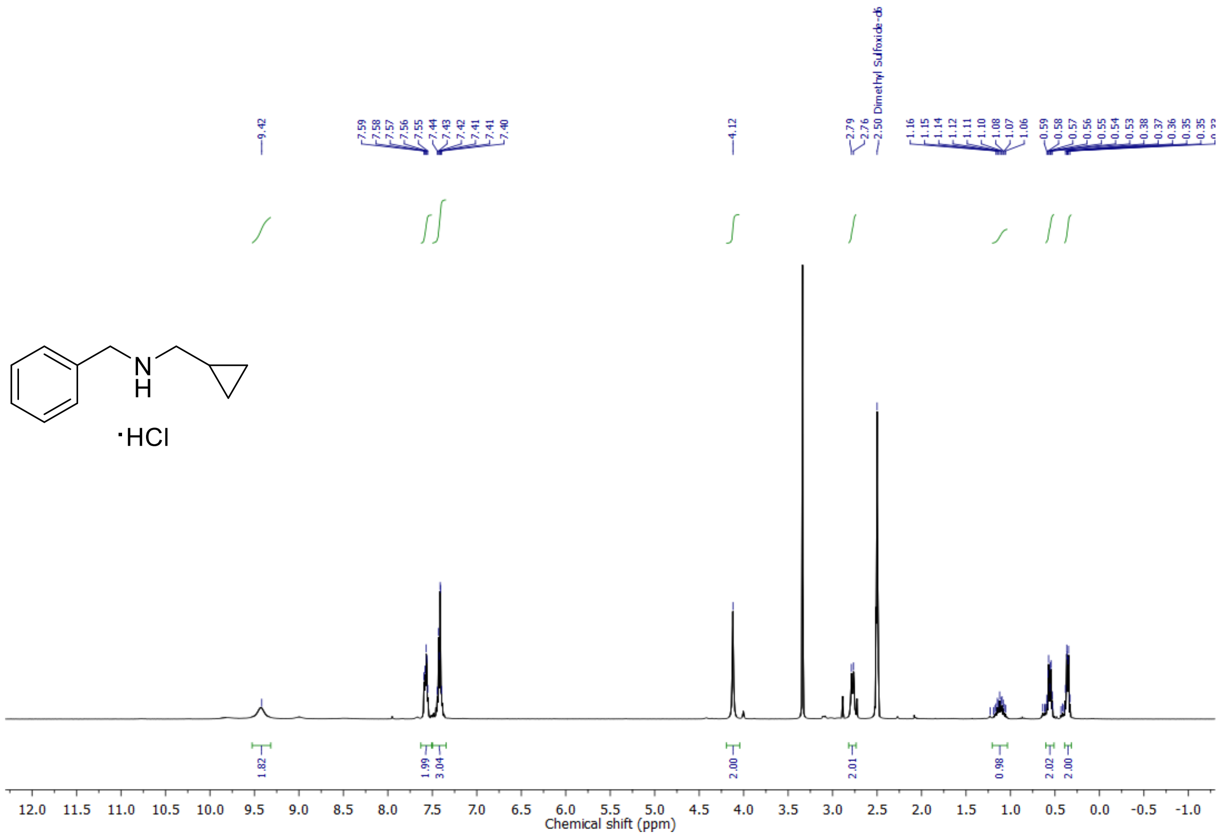

Figure S46: ^{1}H-NMR spectrum of **19**.

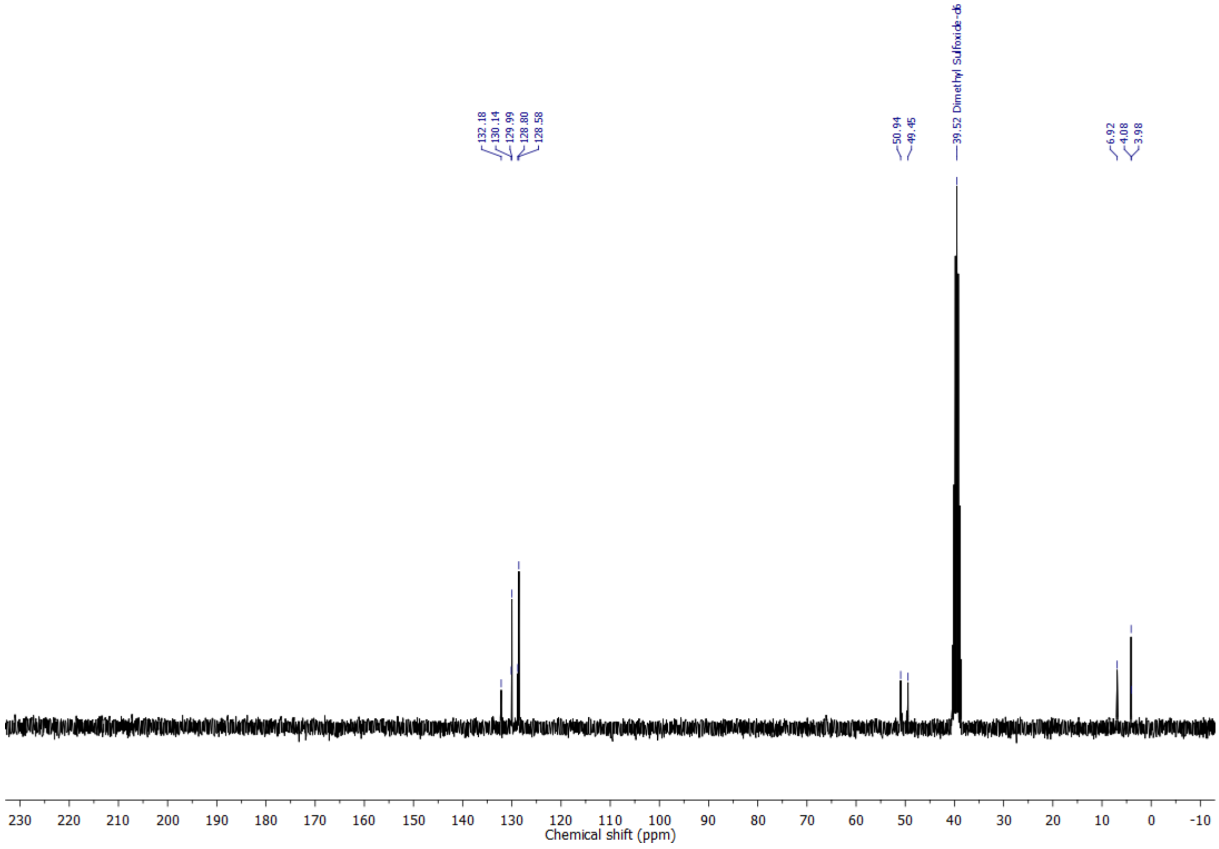

Figure S47: ^{13}C-NMR spectrum of **19**.

N-benzyl-1-(*p*-tolyl)methanaminium chloride (20)

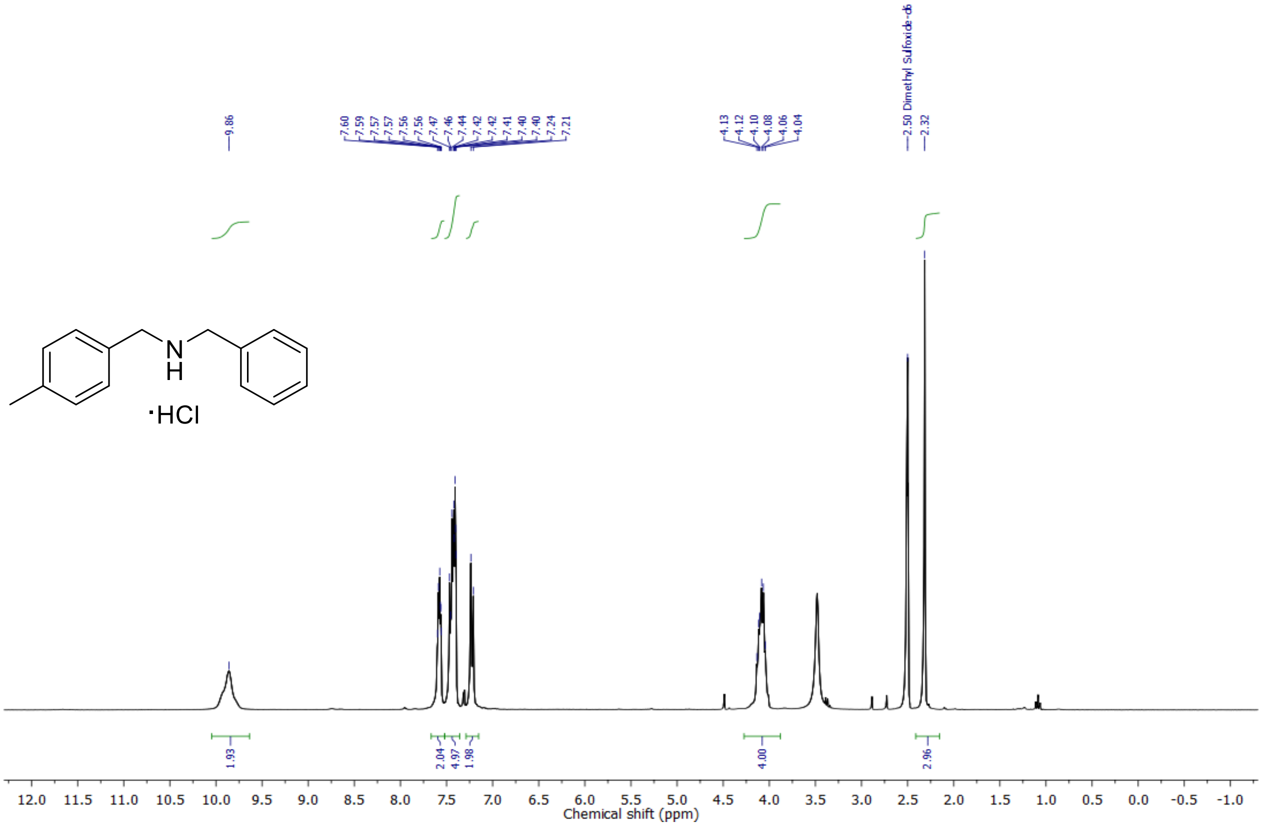

Figure S48: ^{1}H-NMR spectrum of **20**.

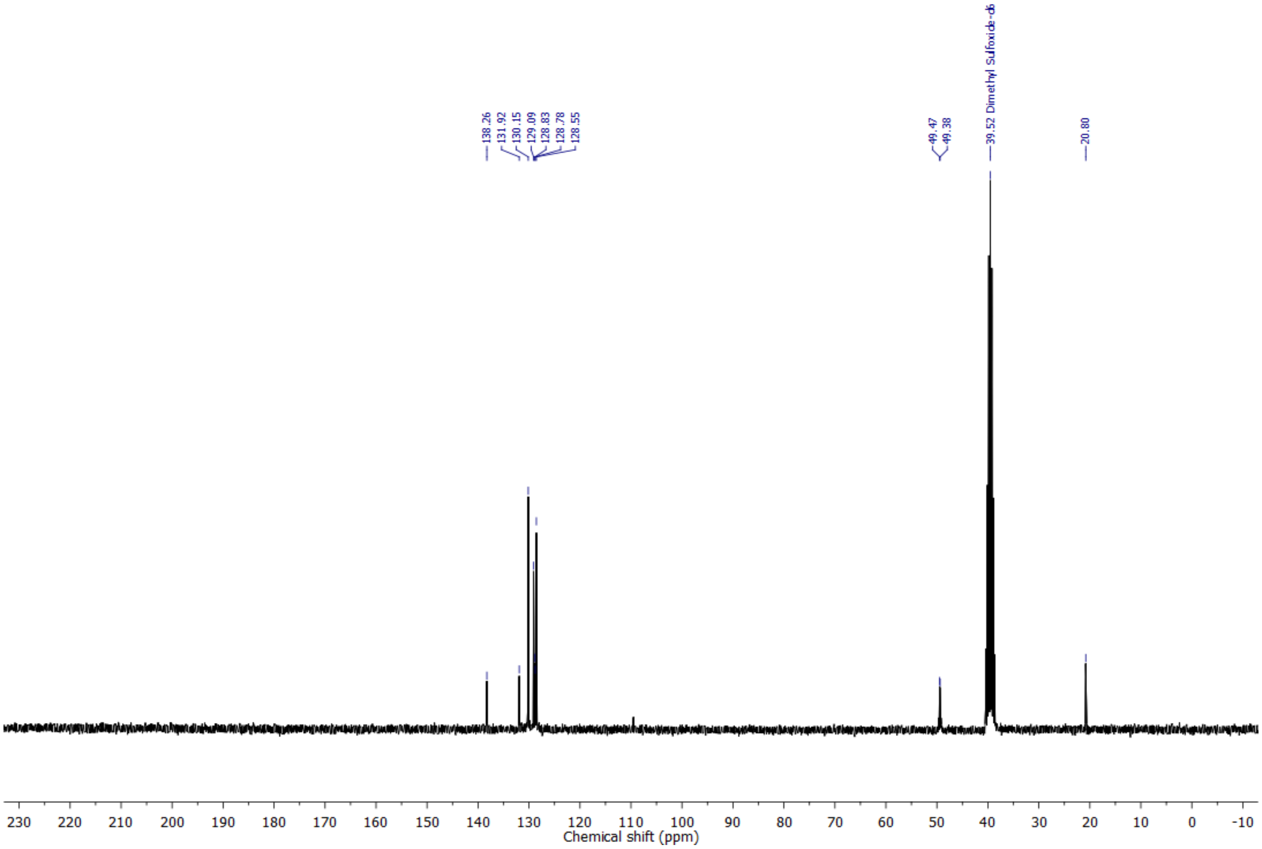

Figure S49: ^{13}C-NMR spectrum of **20**.

***N*-benzyl-1-(*m*-tolyl)methanaminium chloride (21)**

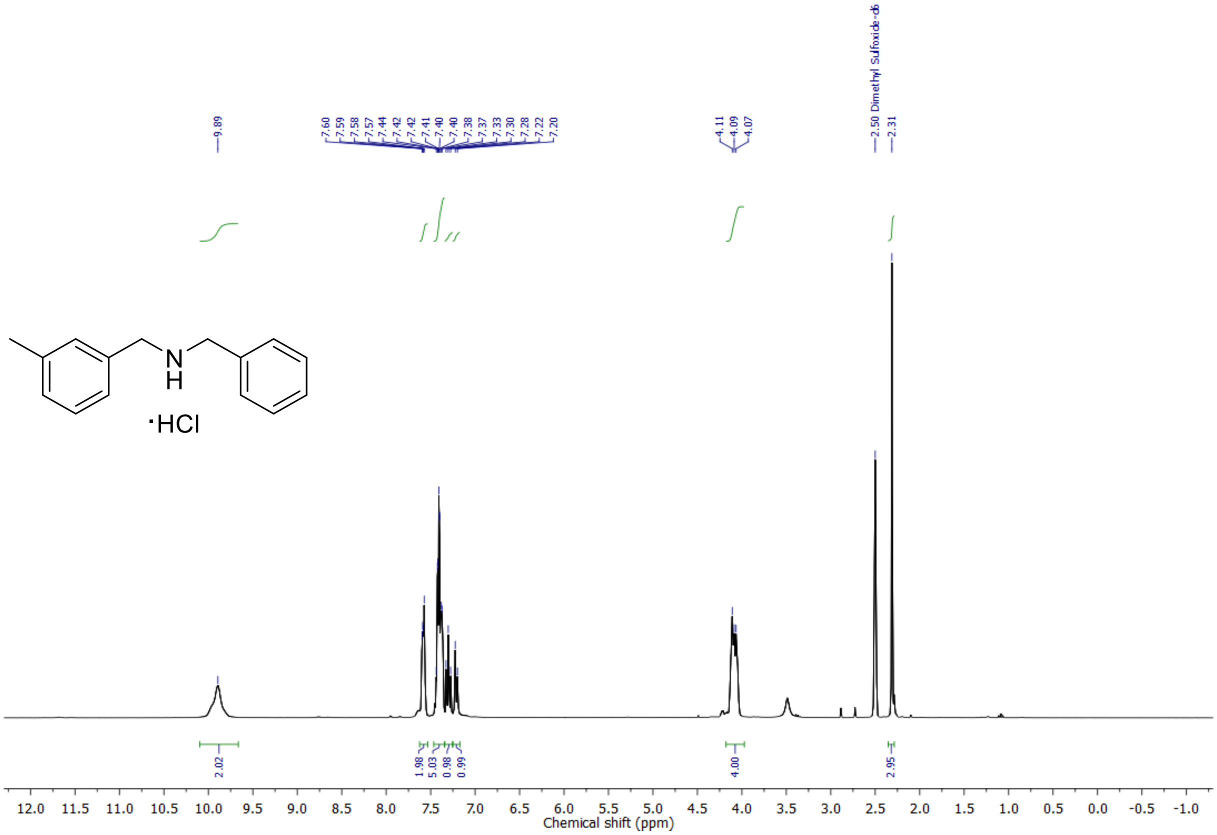

Figure S50: ^{1}H-NMR spectrum of **21**.

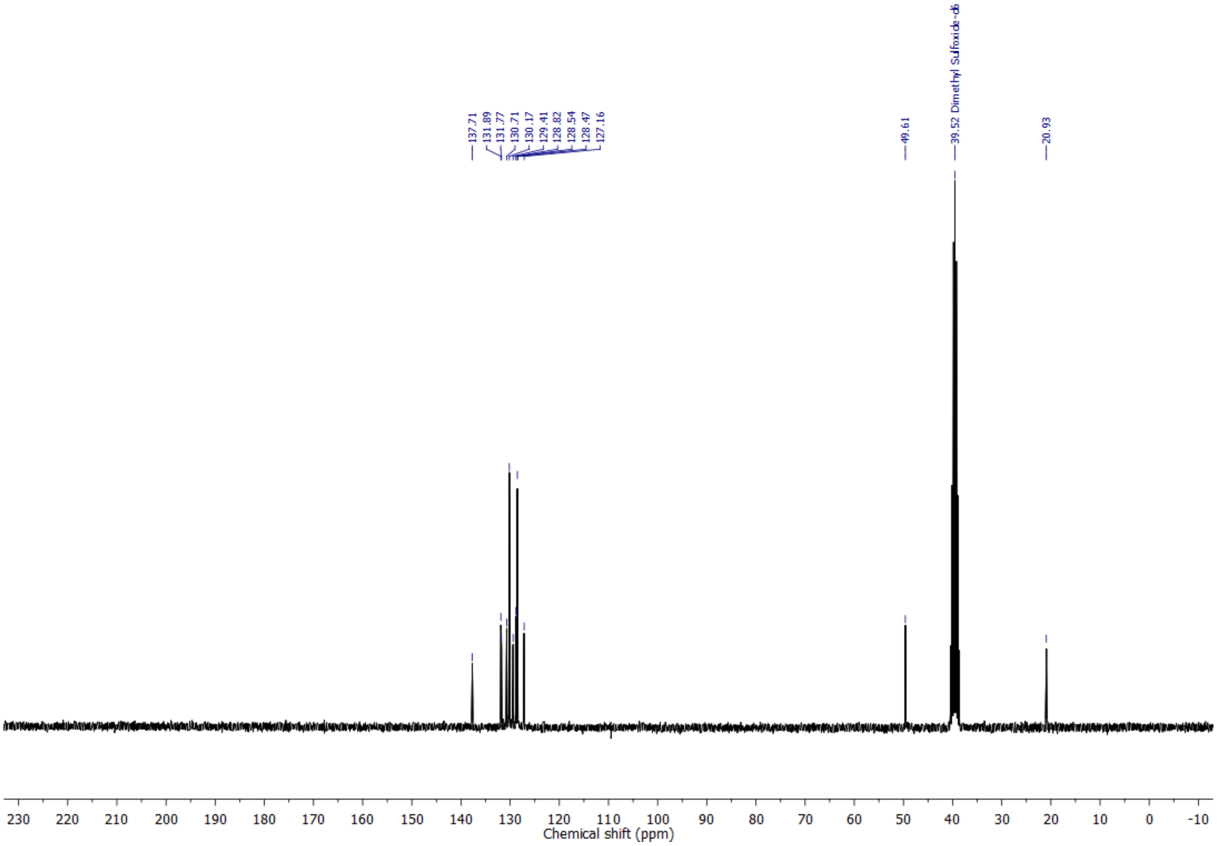

Figure S51: ^{13}C-NMR spectrum of **21**.

N-benzyl-1-(*o*-tolyl)methanaminium chloride (22)

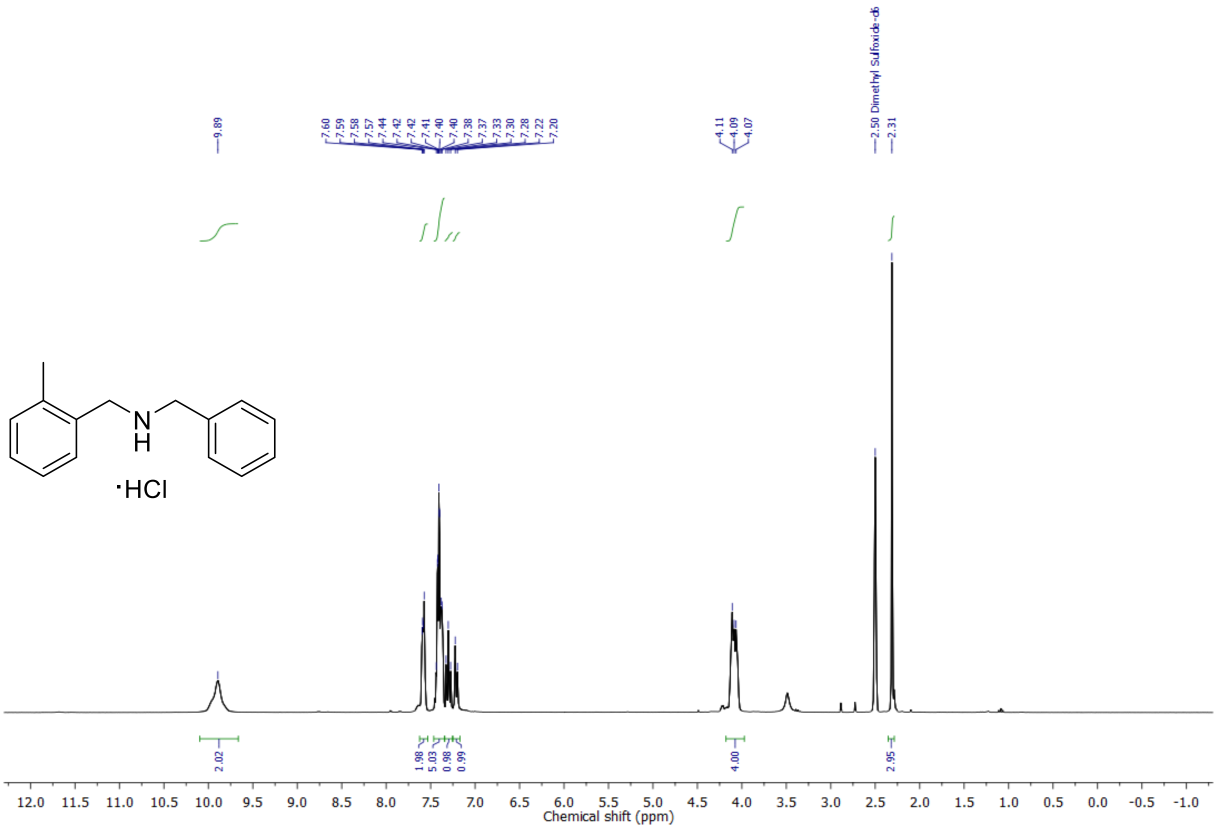

Figure S52: ^{1}H-NMR spectrum of **22**.

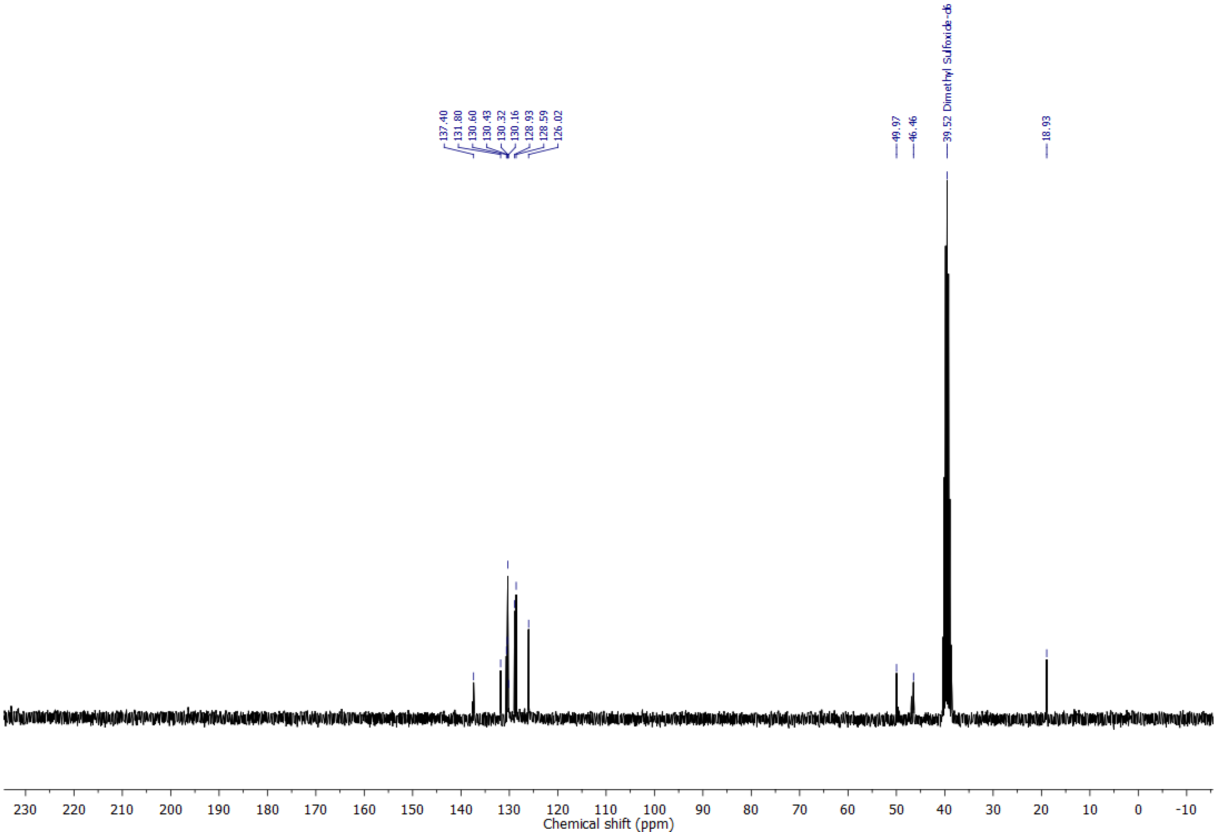

Figure S53: ^{13}C-NMR spectrum of **22**.

***N*-benzyl-1-(4-fluorophenyl)methanaminium chloride (23)**

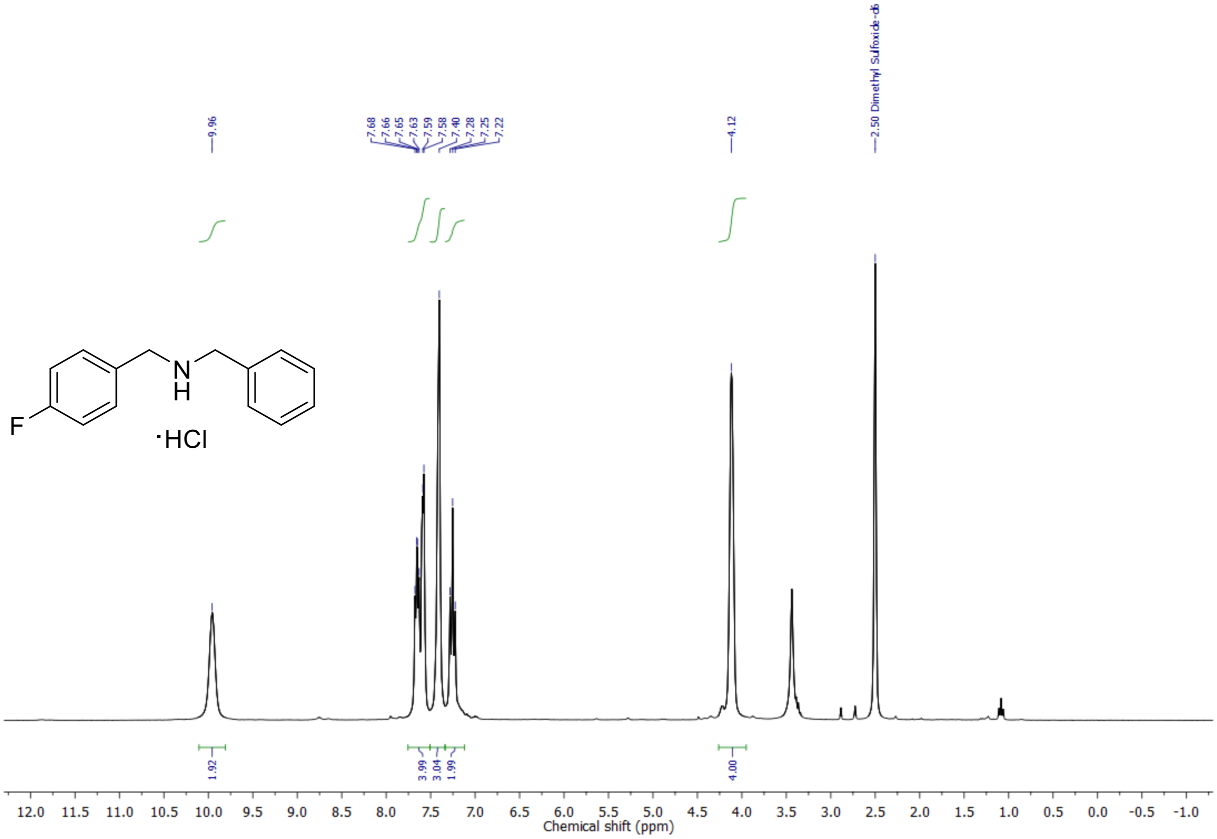

Figure S54: ^{1}H-NMR spectrum of **23**.

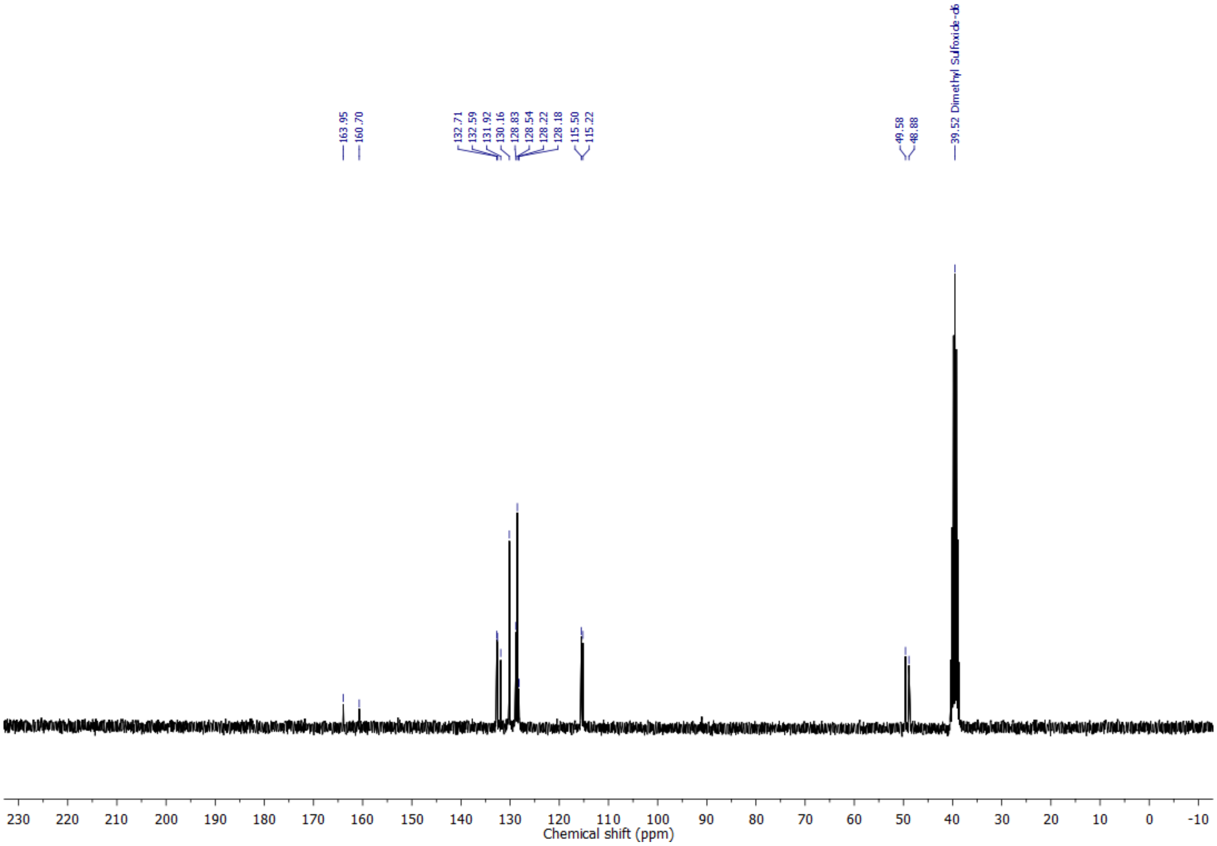

Figure S55: ^{13}C-NMR spectrum of **23**.

_N_-benzyl-1-(4-chlorophenyl)methanaminium chloride (24)

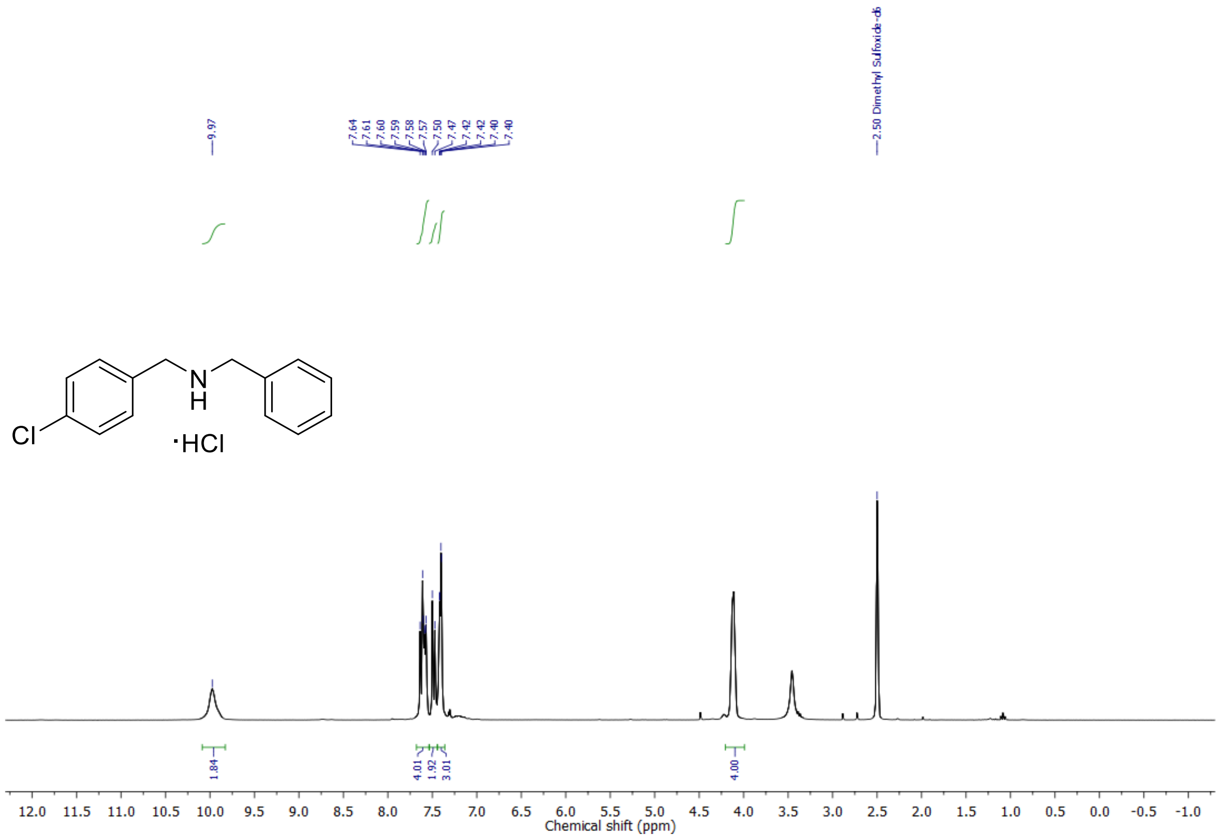

Figure S56: ^{1}H-NMR spectrum of **24**.

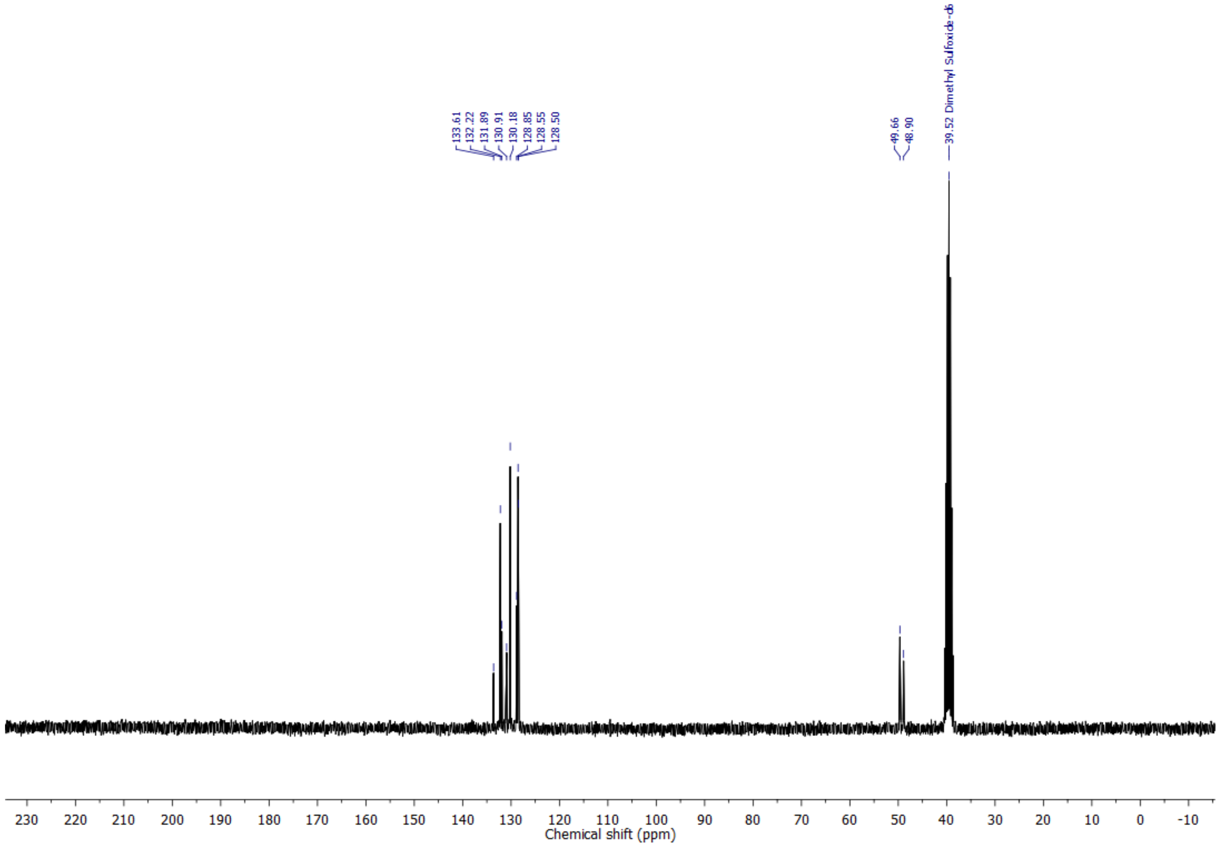

Figure S57: ^{13}C-NMR spectrum of **24**.

***N*-benzyl-1-(3-chlorophenyl)methanaminium chloride (25)**

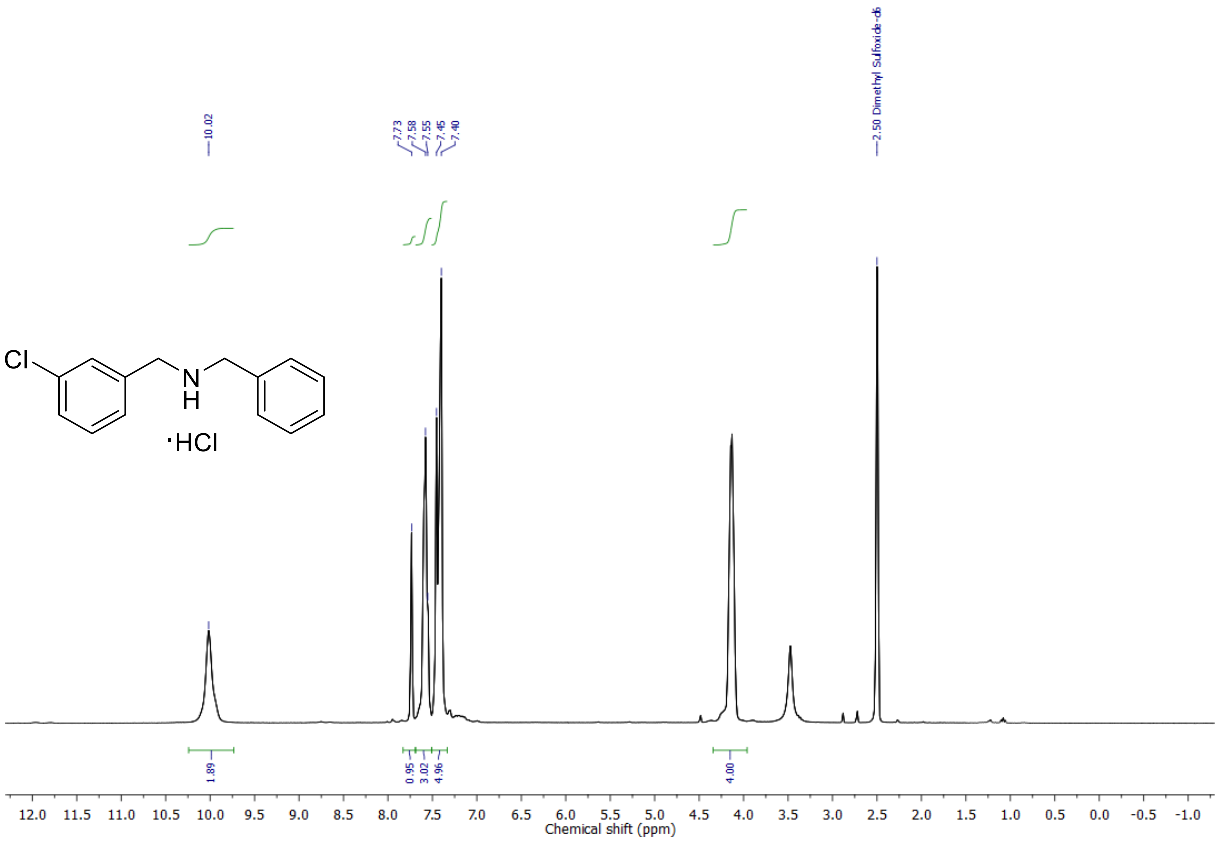

Figure S58: ^{1}H-NMR spectrum of **25**.

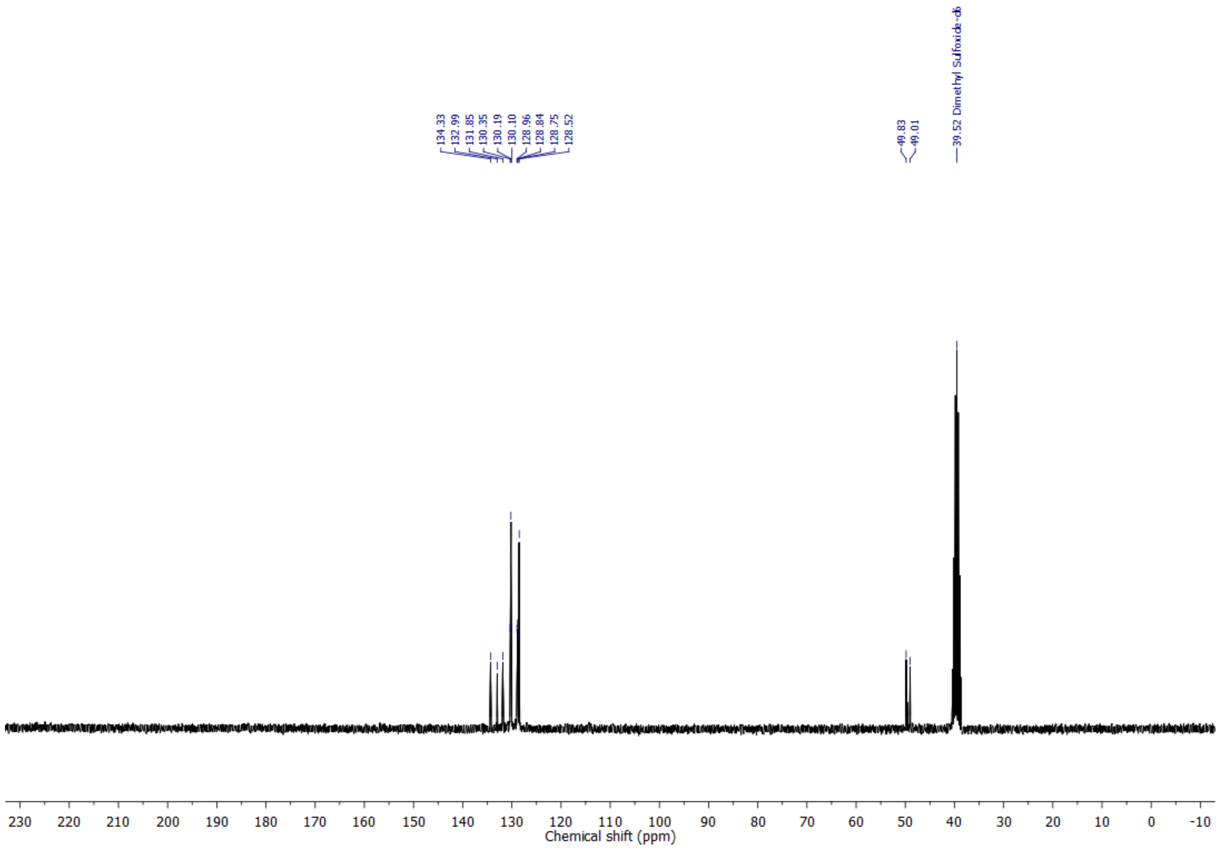

Figure S59: ^{13}C-NMR spectrum of **25**.

N-benzyl-1-(2-chlorophenyl)methanaminium chloride (26)

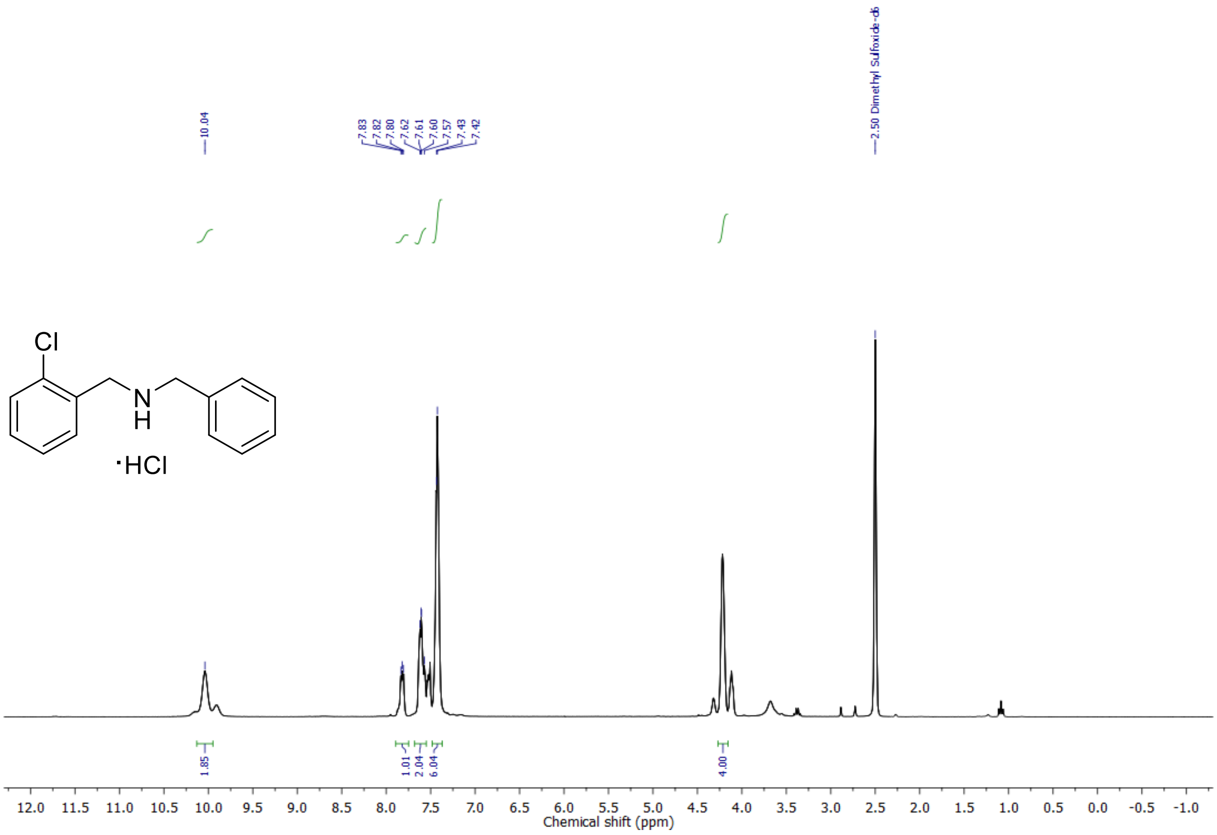

Figure S60: ^{1}H-NMR spectrum of **26**.

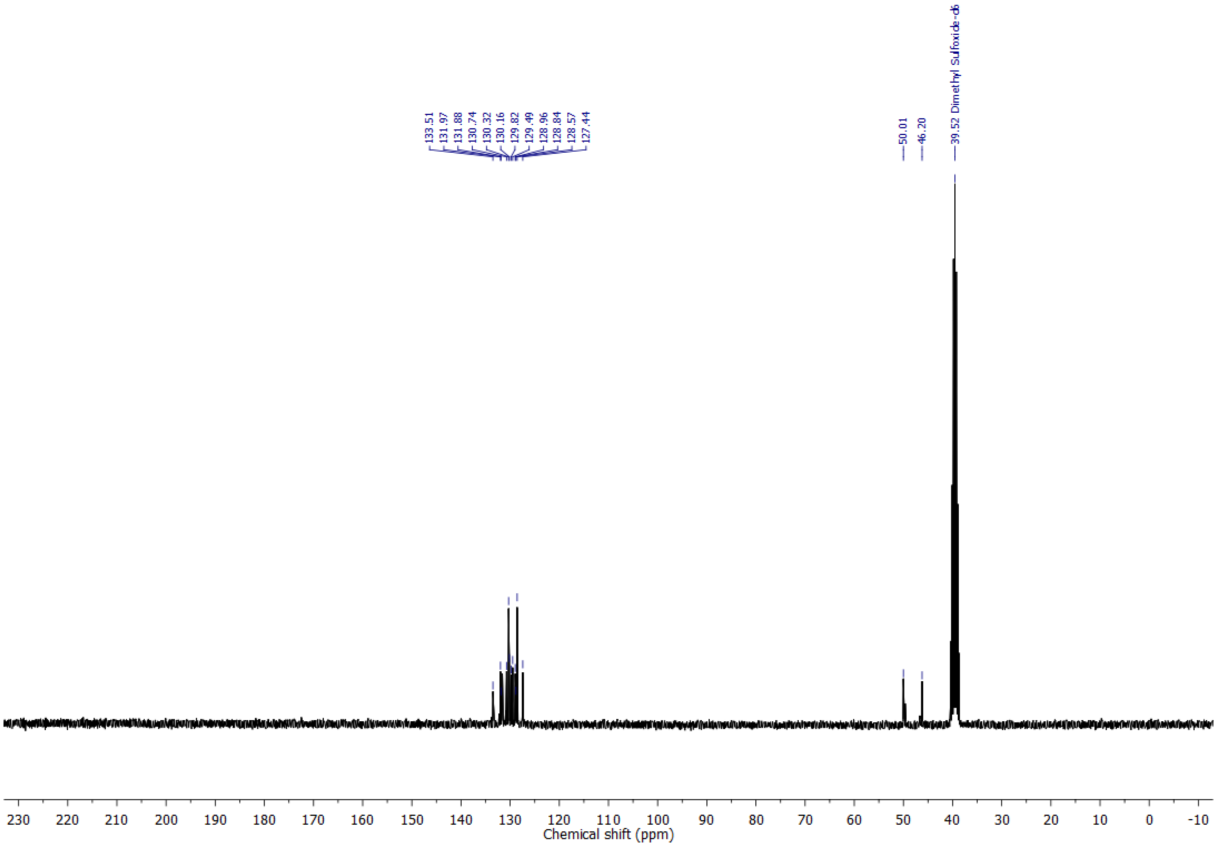

Figure S61: ^{13}C-NMR spectrum of **26**.

N-benzyl-1-(4-bromophenyl)methanaminium chloride (27)

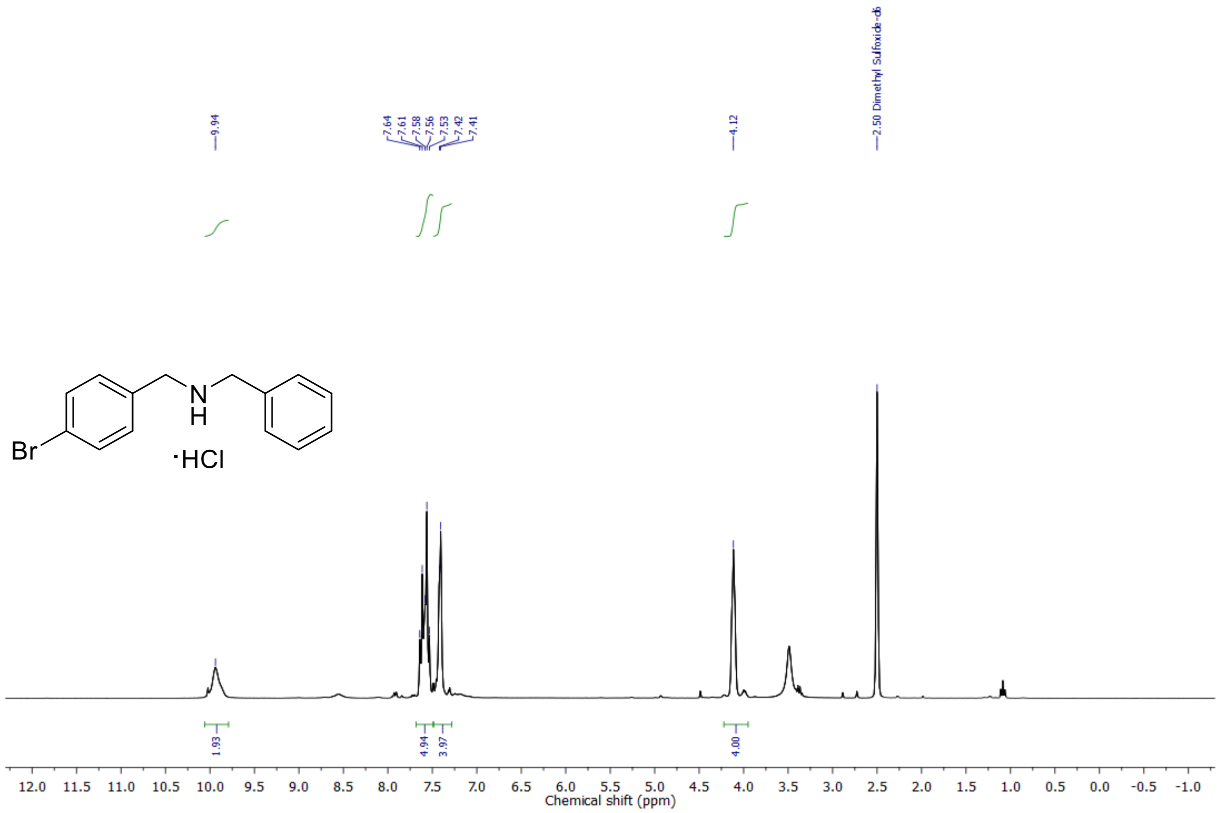

Figure S62: ^{1}H-NMR spectrum of **27**.

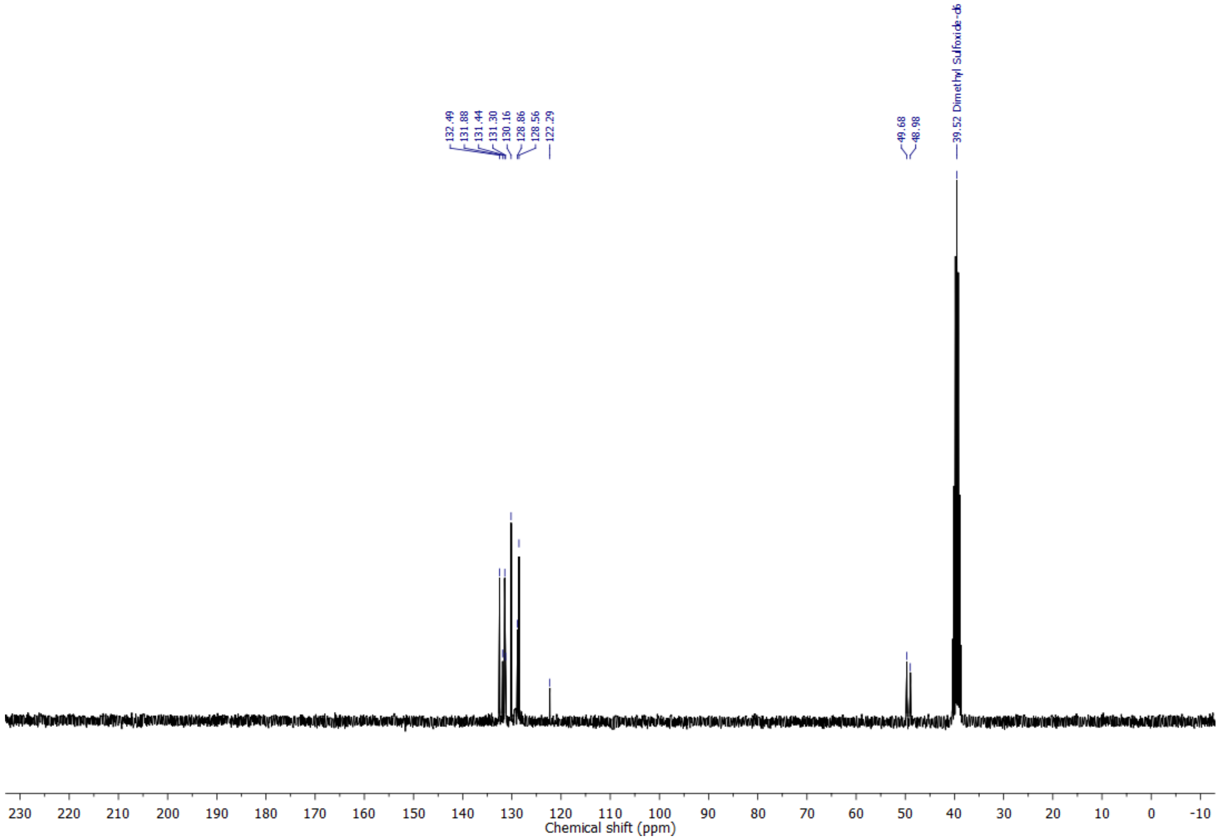

Figure S63: ^{13}C-NMR spectrum of **27**.

N-benzyl-1-(4-methoxyphenyl)methanaminium chloride (28)

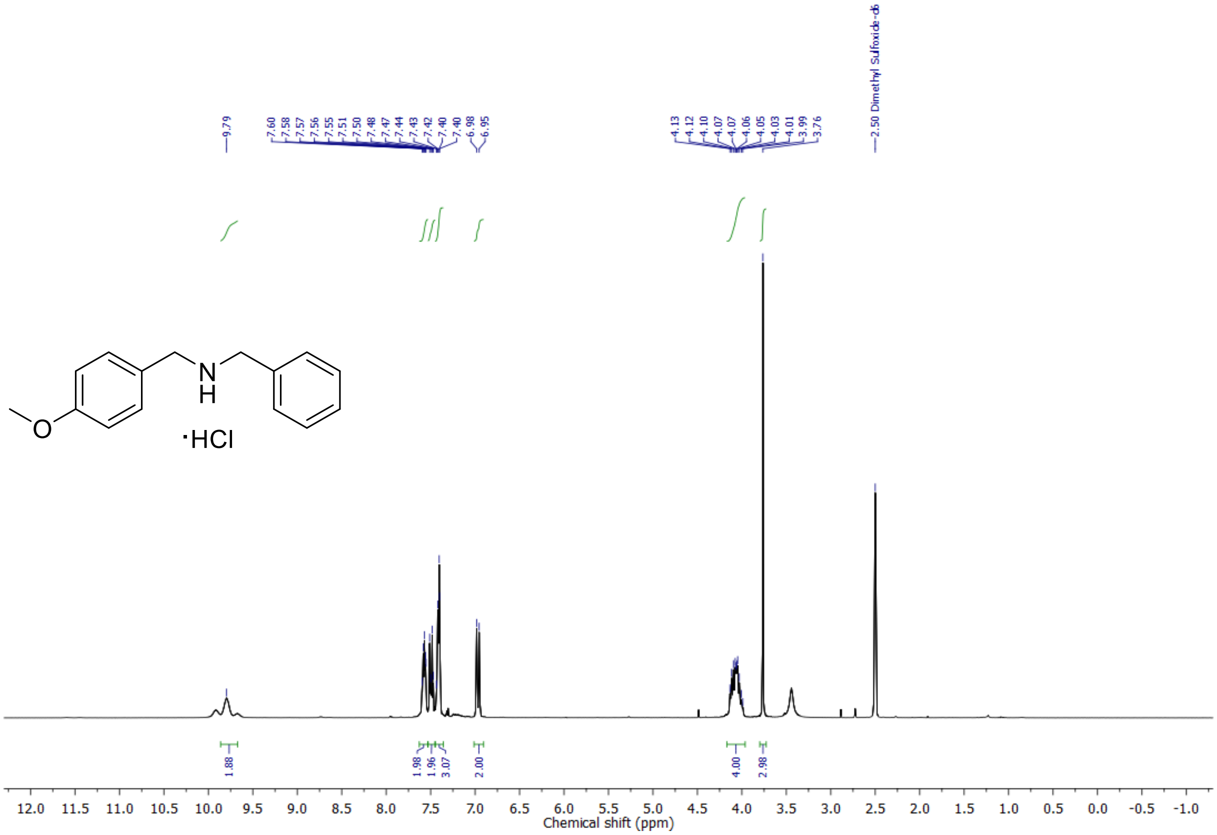

Figure S64: ^{1}H-NMR spectrum of **28**.

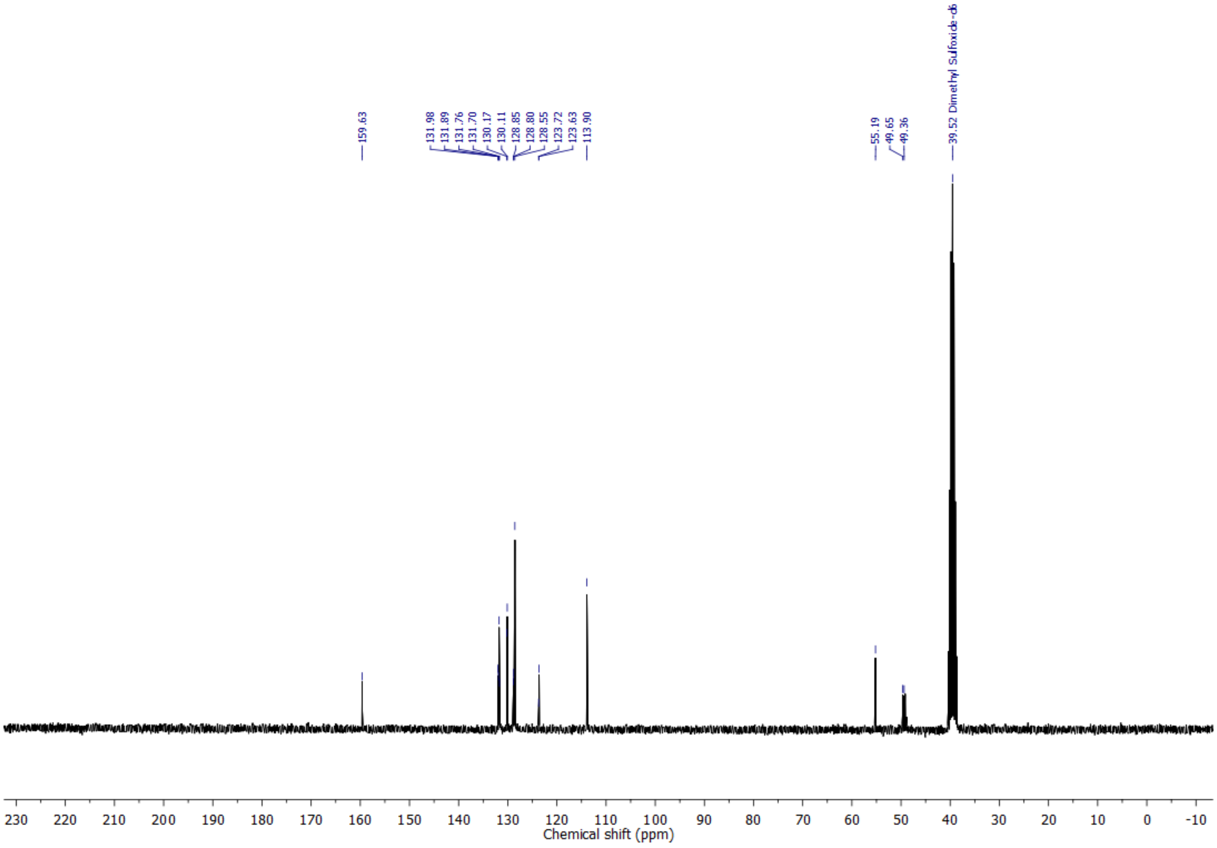

Figure S65: ^{13}C-NMR spectrum of **28**.

***N*-benzyl-1-(3-methoxyphenyl)methanaminium chloride (29)**

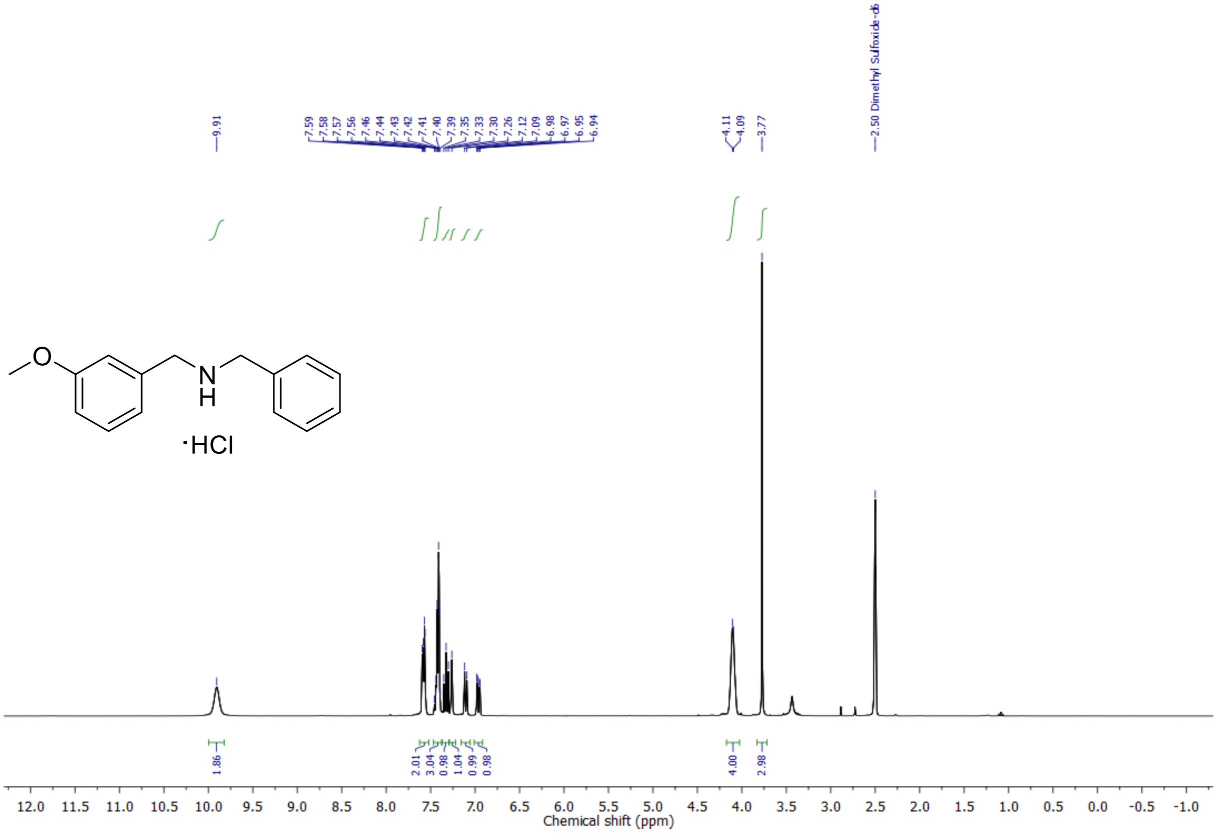

Figure S66: ^{1}H-NMR spectrum of **29**.

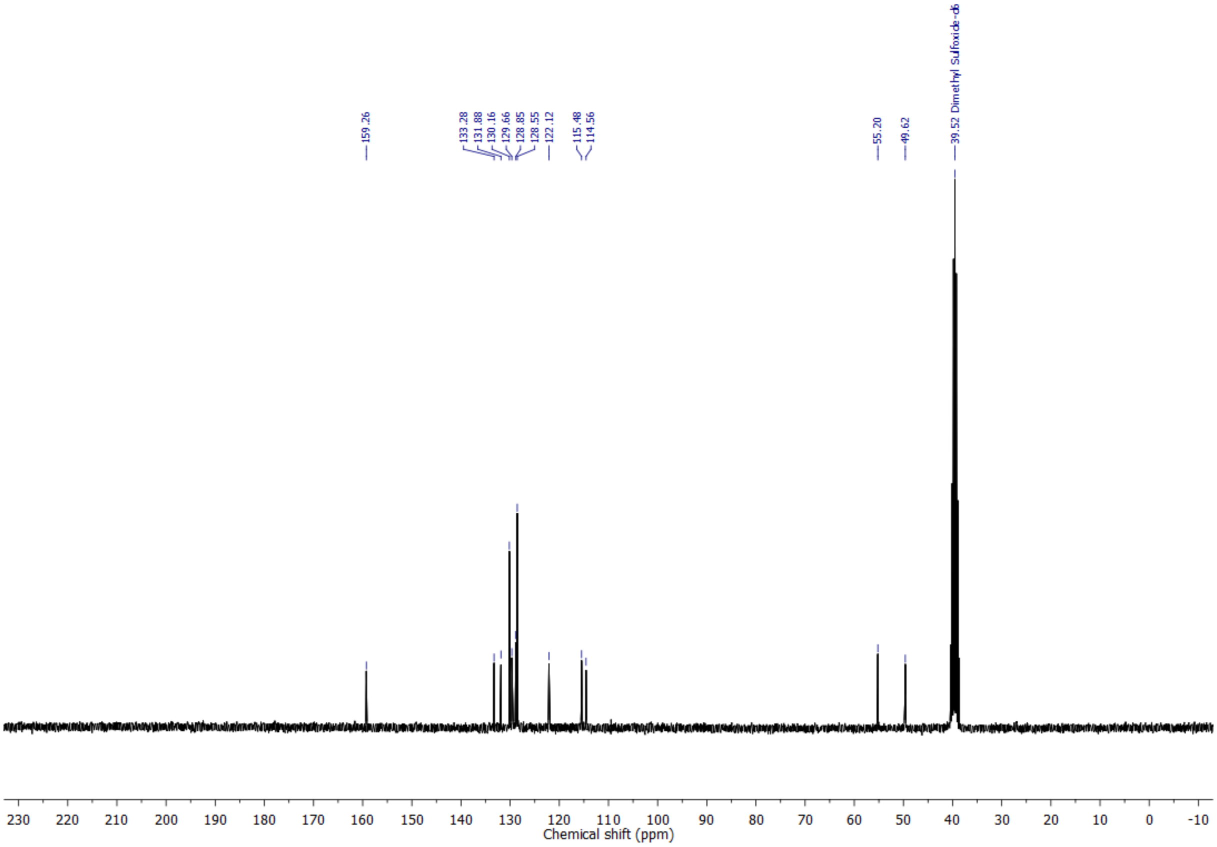

Figure S67: ^{13}C-NMR spectrum of **29**.

N-benzyl-1-(2,6-difluorophenyl)methanaminium chloride (30)

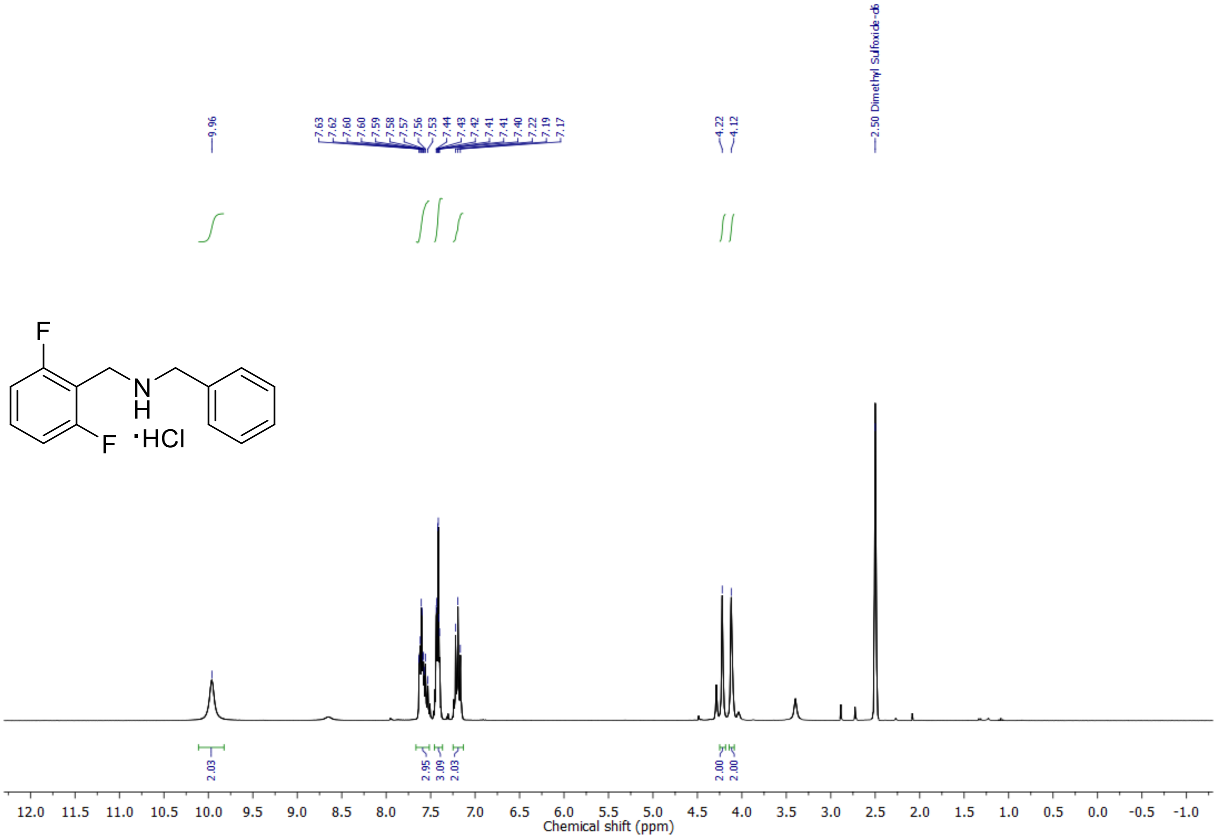

Figure S68: ^{1}H-NMR spectrum of **30**.

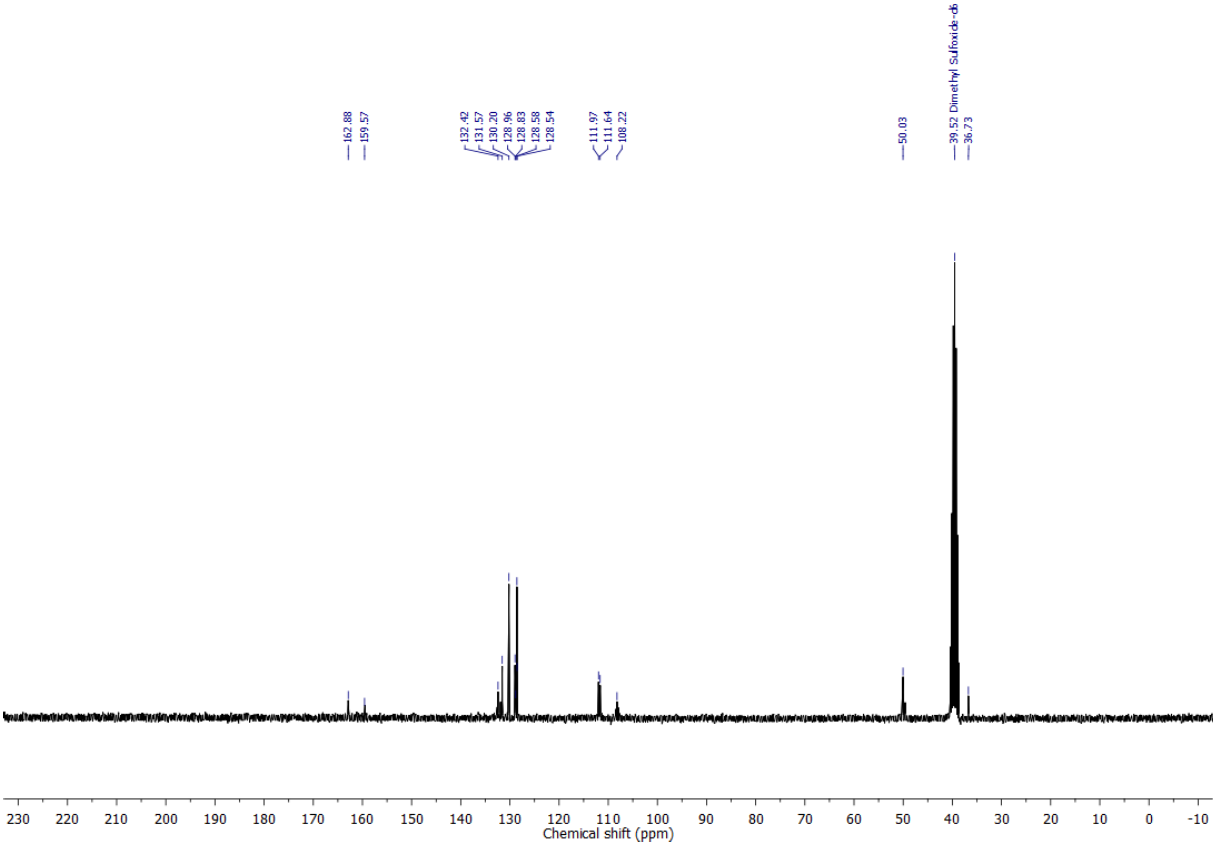

Figure S69: ^{13}C-NMR spectrum of **30**.

N-benzyl-1-(maphthalen-2-yl)methanaminium chloride (31)

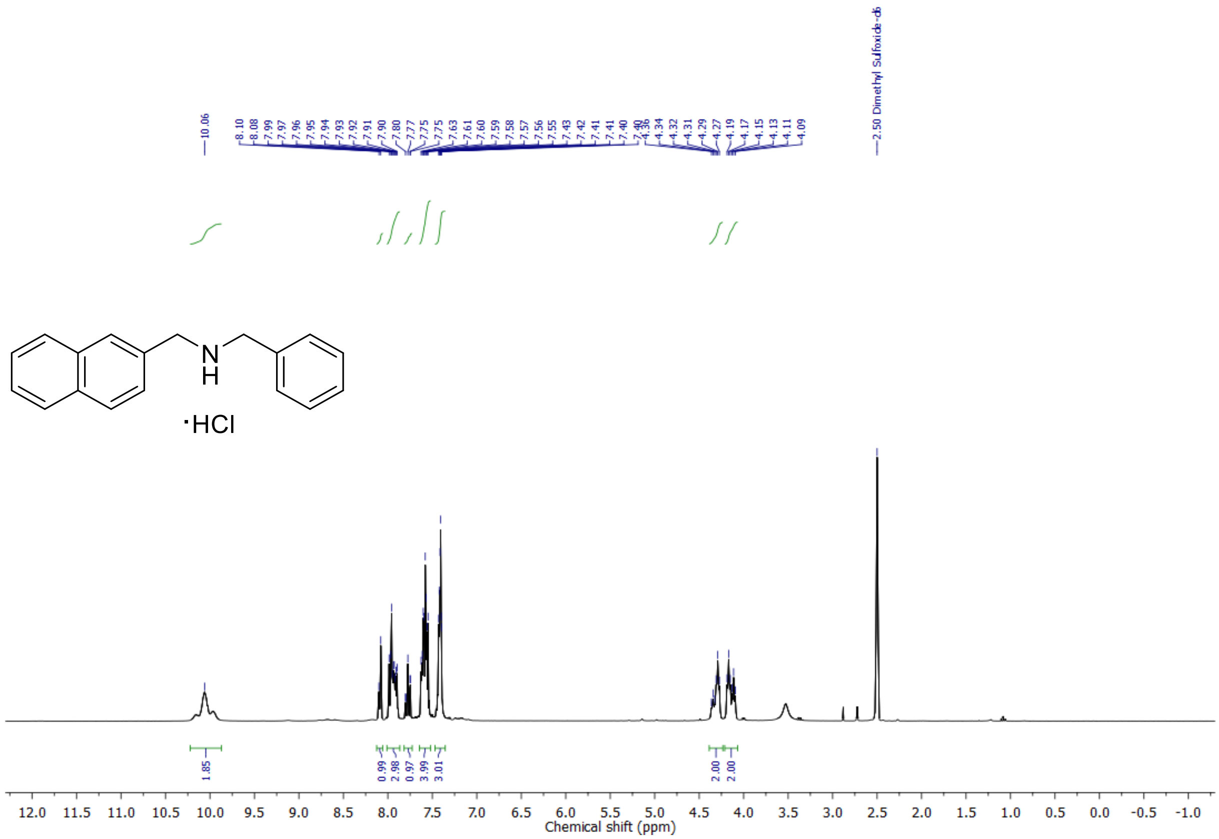

Figure S70: ^{1}H-NMR spectrum of **31**.

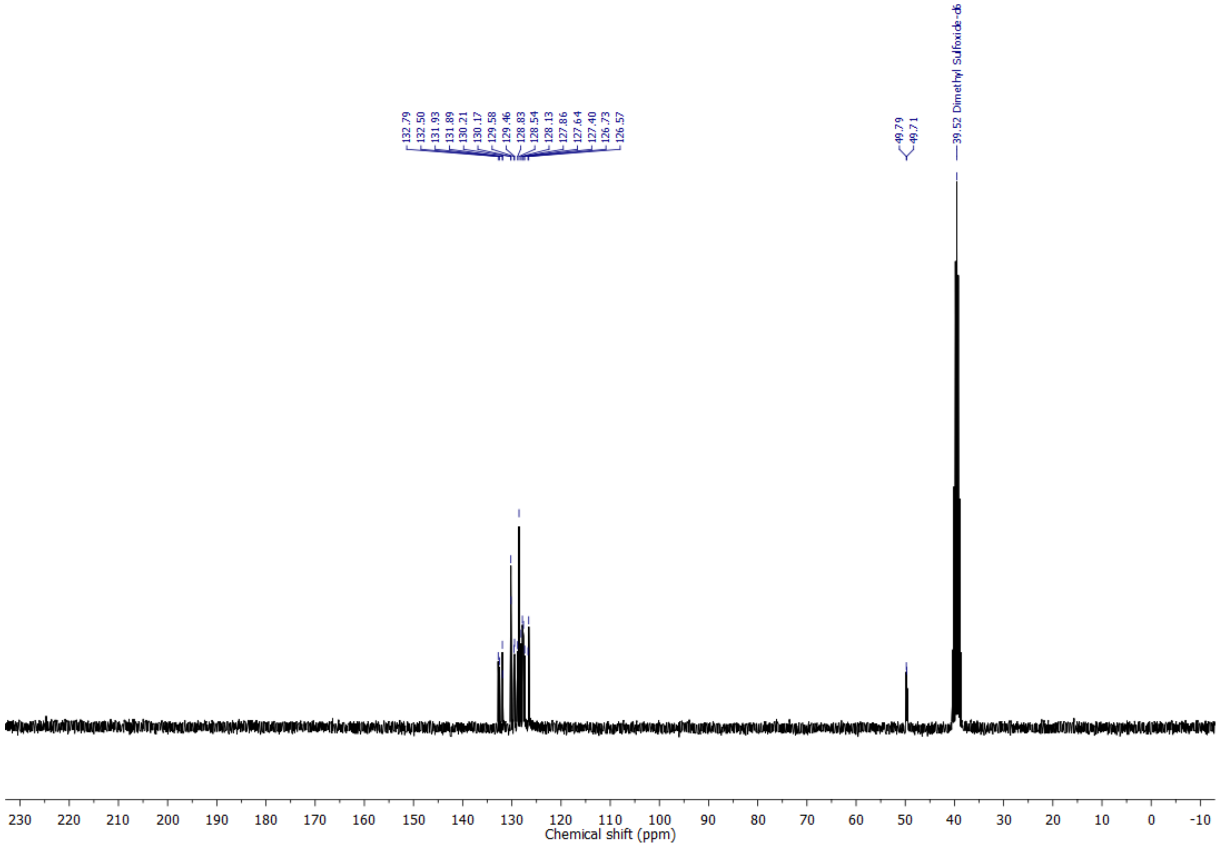

Figure S71: ^{13}C-NMR spectrum of **31**.

***N*-benzyl-1-(furan-2-yl)methanaminium chloride (32)**

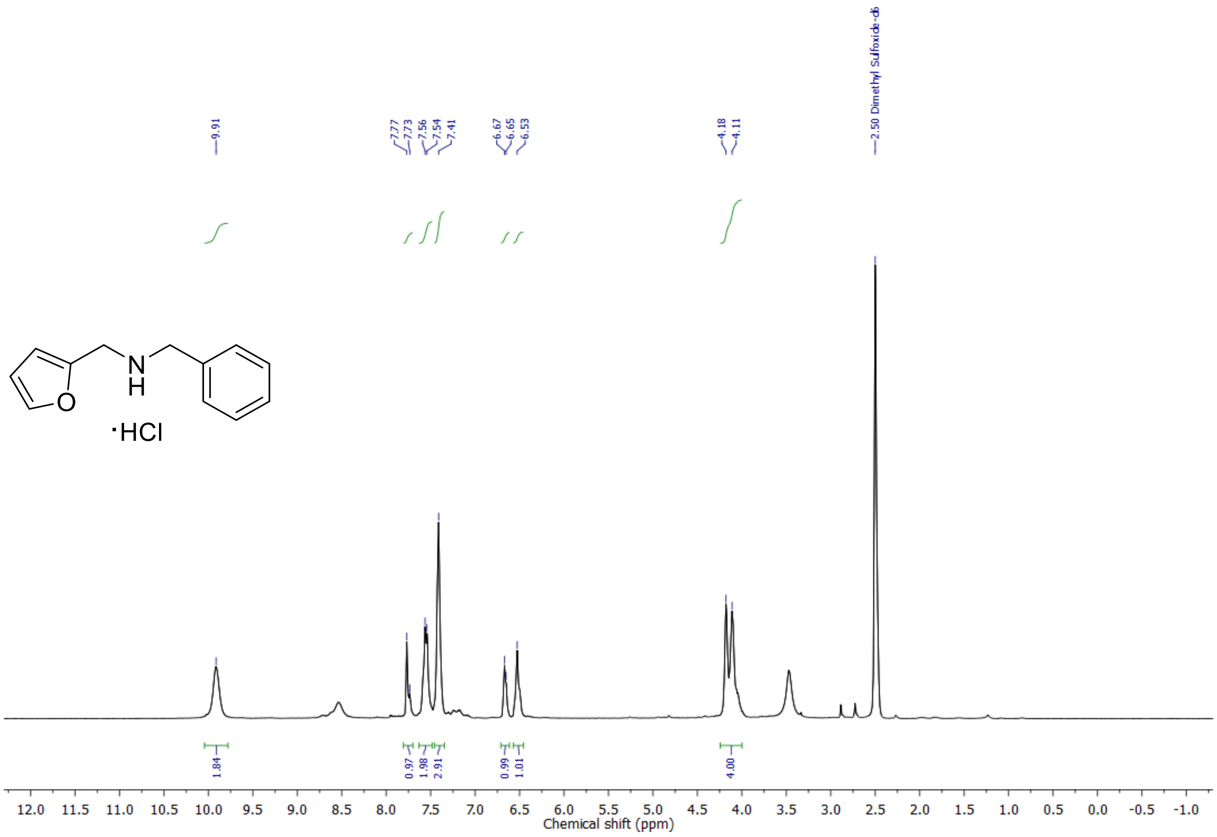

Figure S72: ^{1}H-NMR spectrum of **32**.

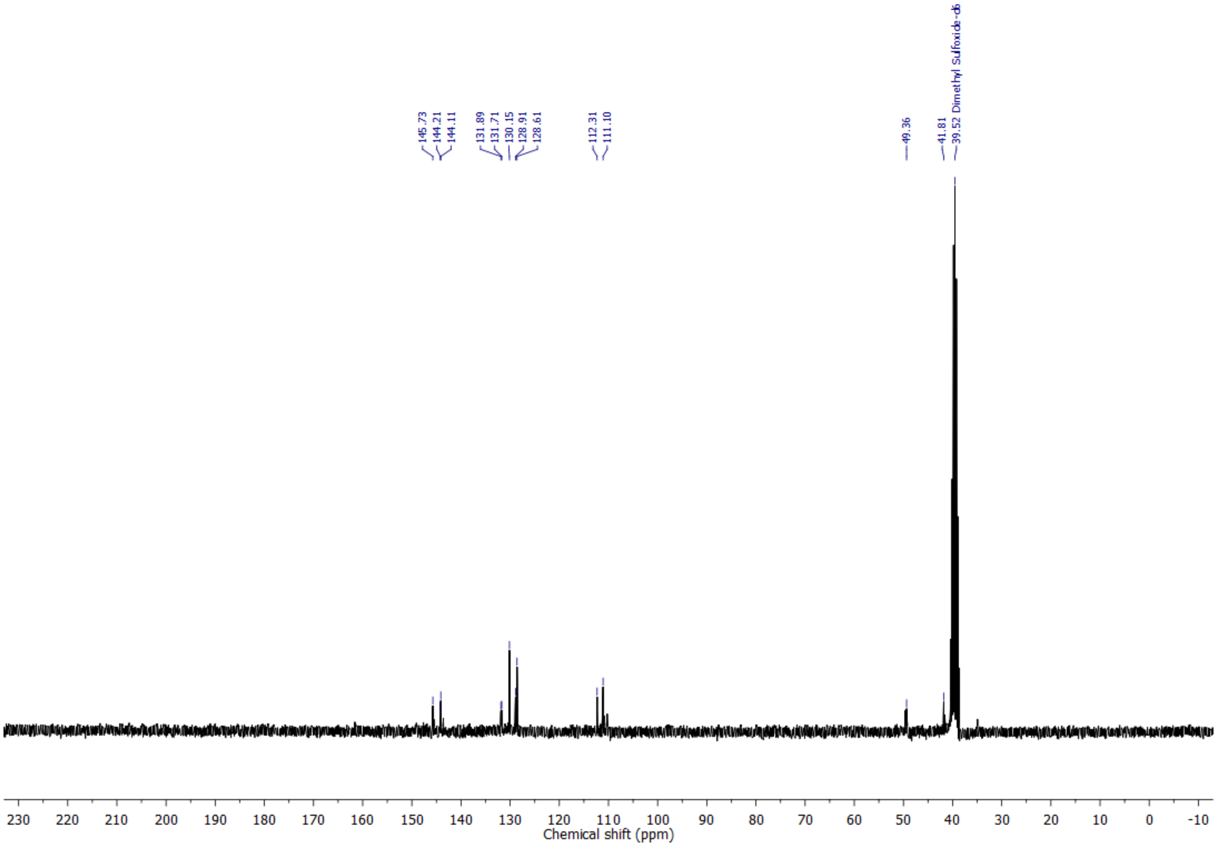

Figure S73: ^{13}C-NMR spectrum of **32**.

N-(4-methylbenzyl)pentan-1-aminium chloride (33)

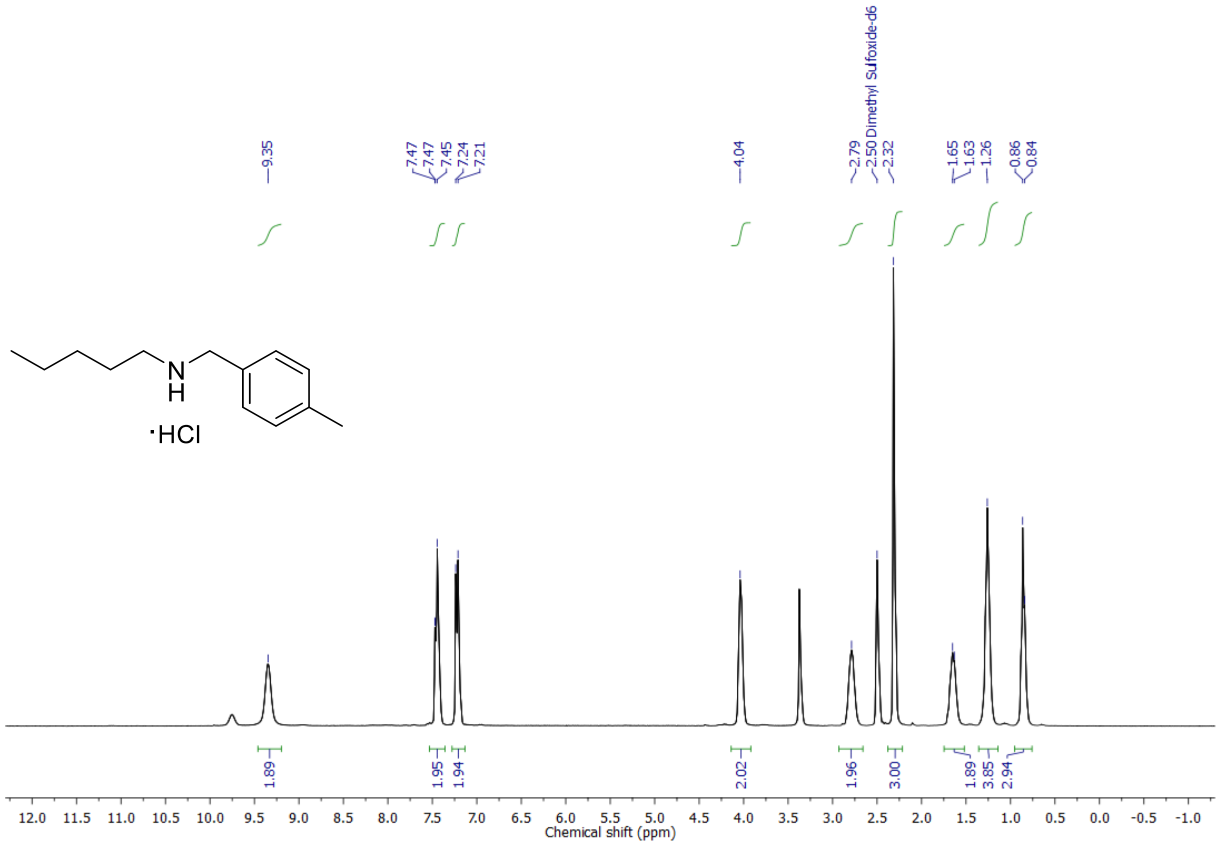

Figure S74: ^{1}H-NMR spectrum of **33**.

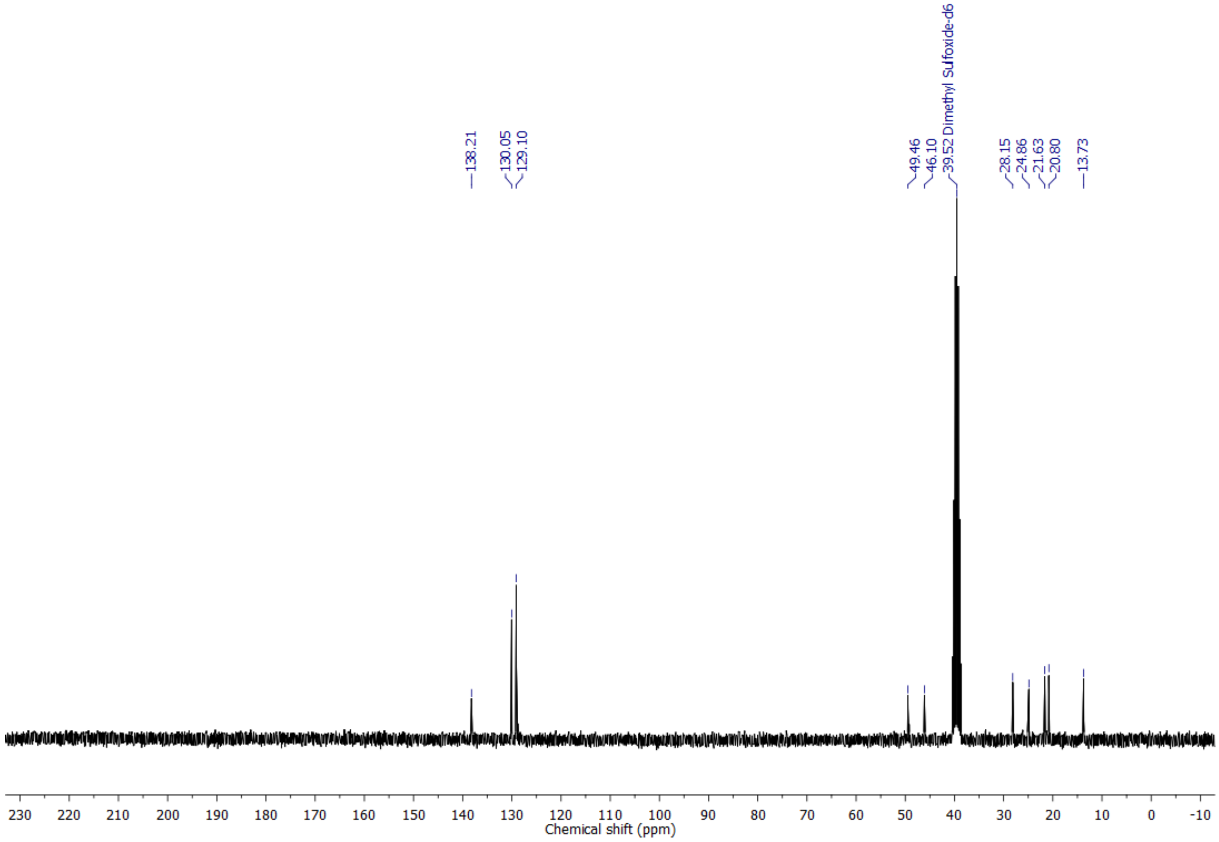

Figure S75: ^{13}C-NMR spectrum of **33**.

_N_-(3-methylbenzyl)pentan-1-aminium chloride (34)

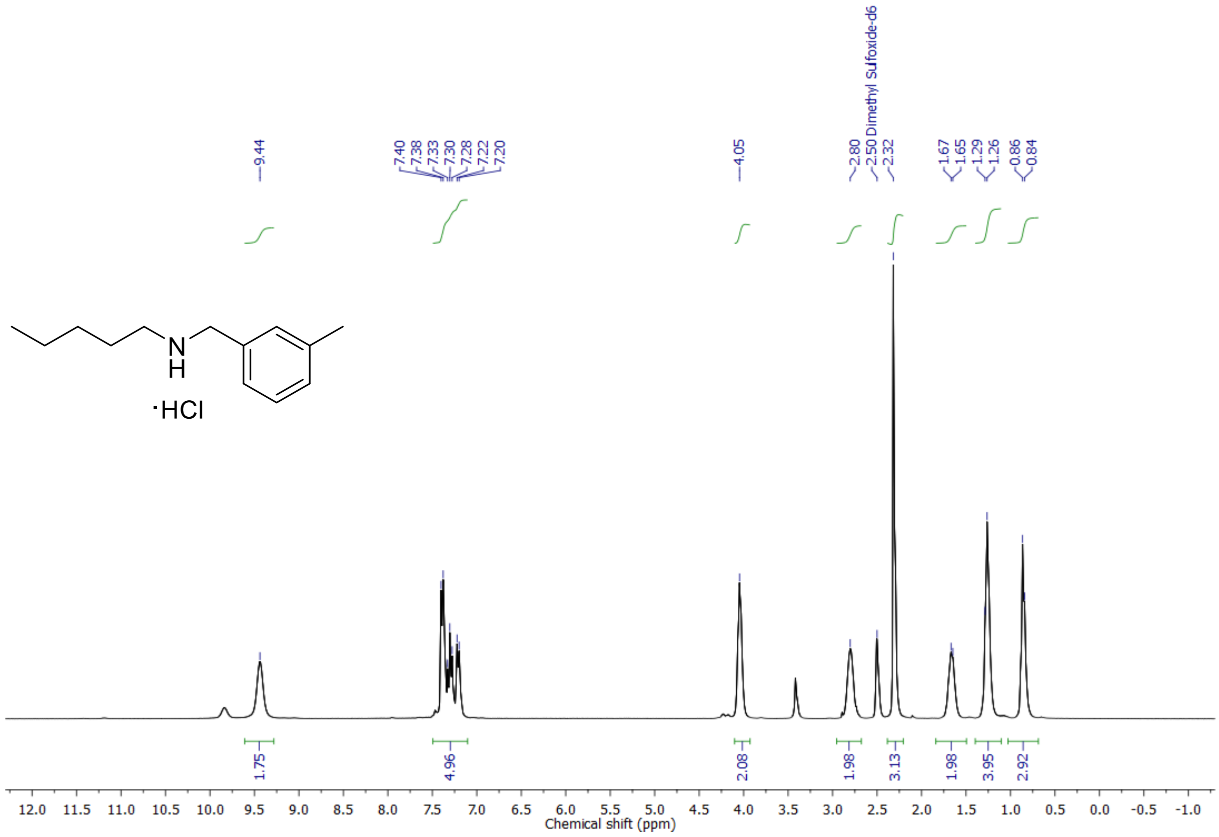

Figure S76: ^{1}H-NMR spectrum of **34**.

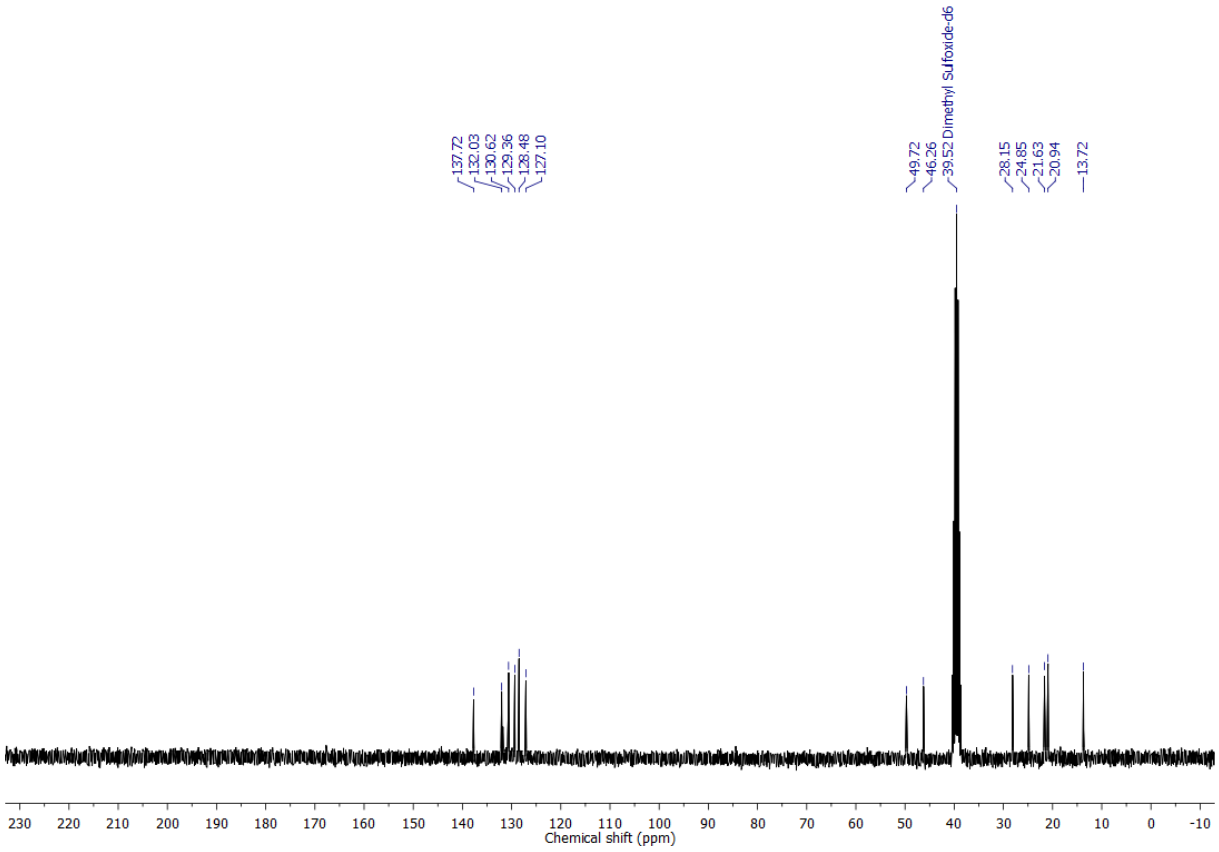

Figure S77: ^{13}C-NMR spectrum of **34**.

***N*-(2-methylbenzyl)pentan-1-aminium chloride (35)**

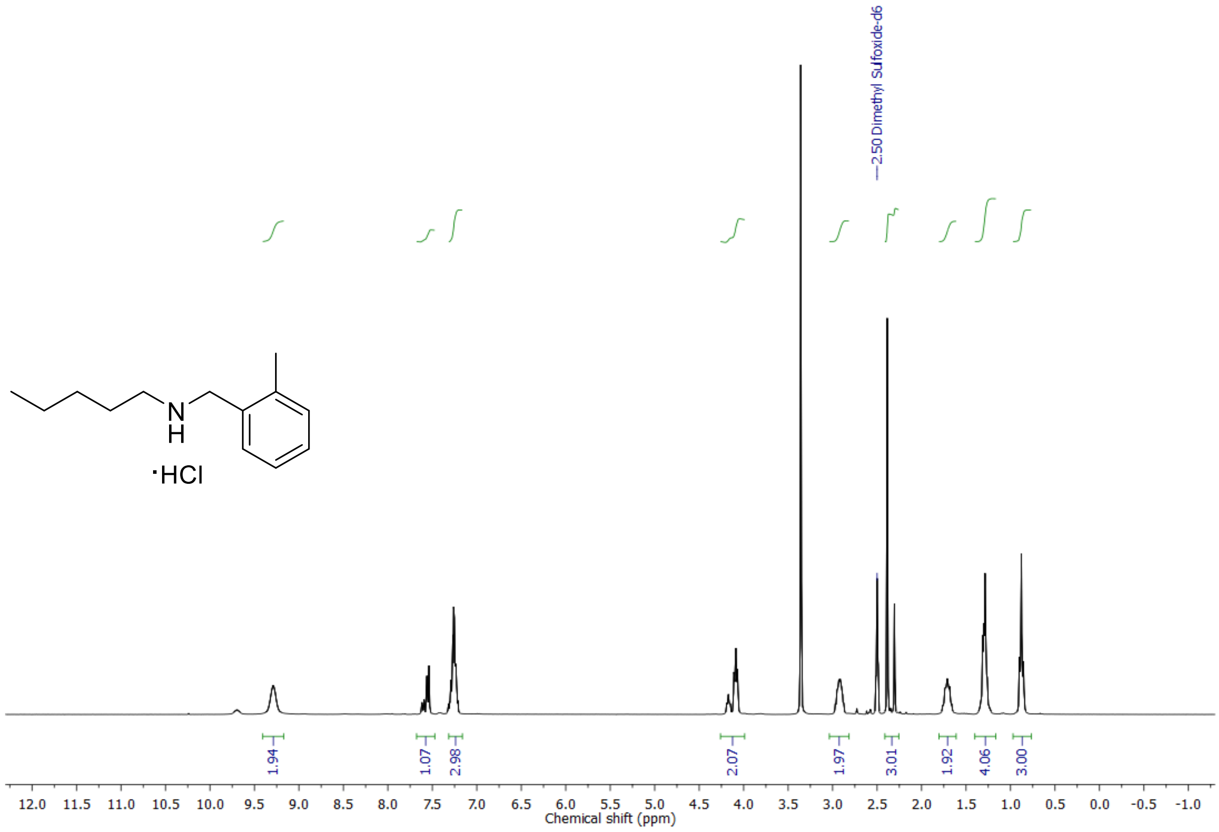

Figure S78: ^{1}H-NMR spectrum of **35**.

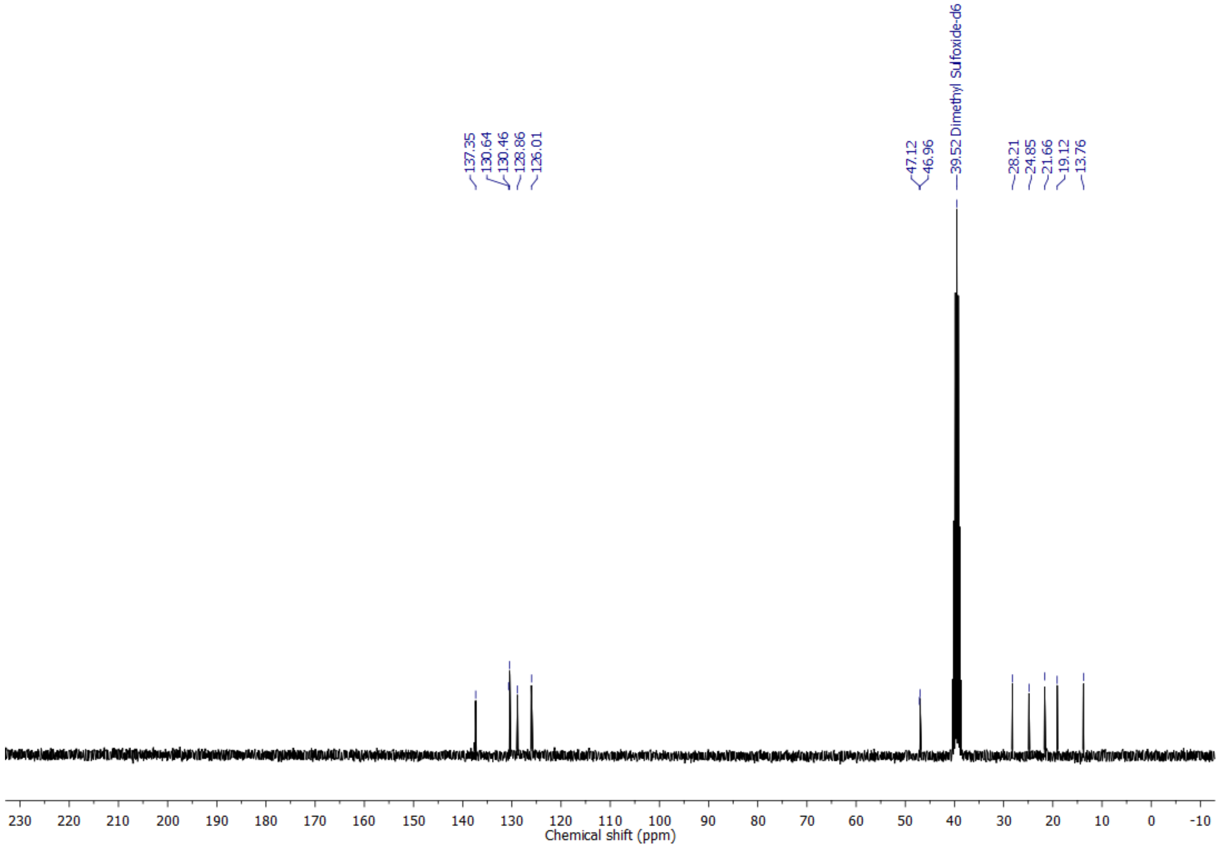

Figure S79: ^{13}C-NMR spectrum of **35**.

N-(4-fluorobenzyl)pentan-1-aminium chloride (36)

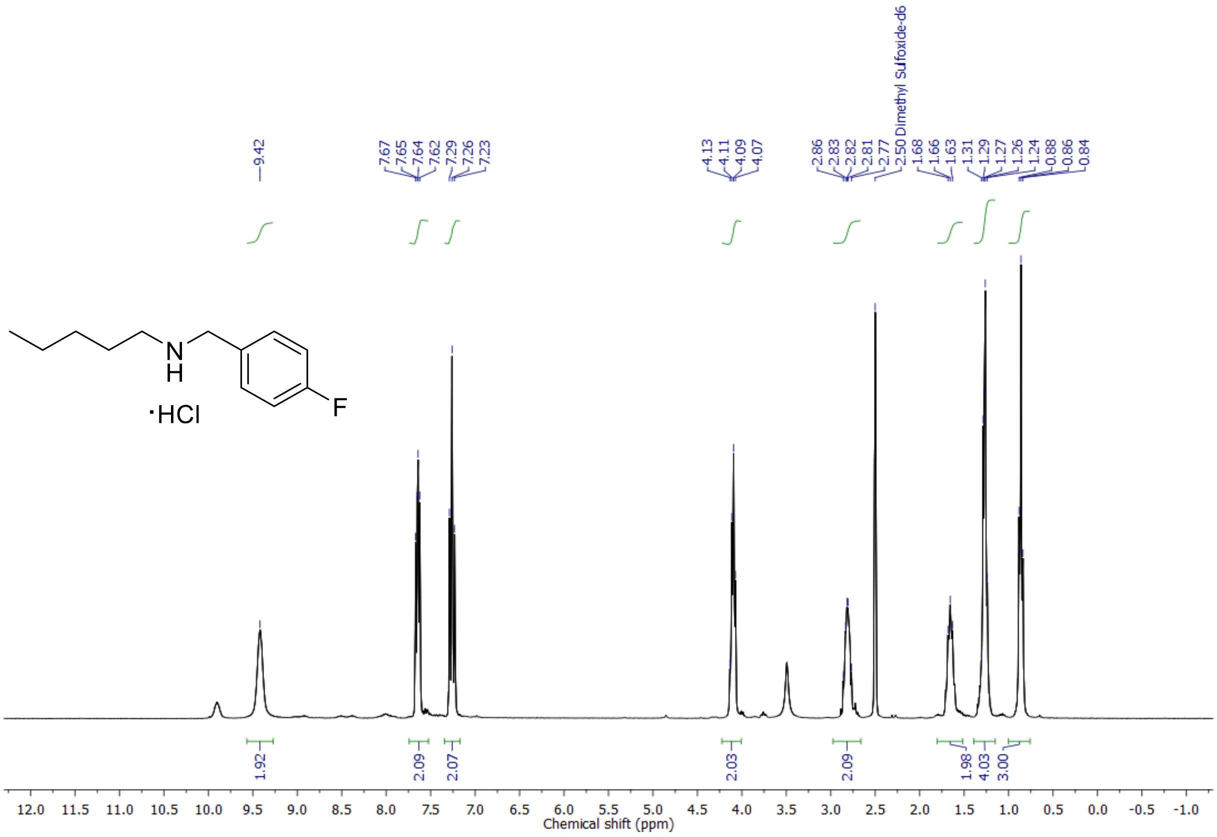

Figure S80: ^{1}H-NMR spectrum of **36**.

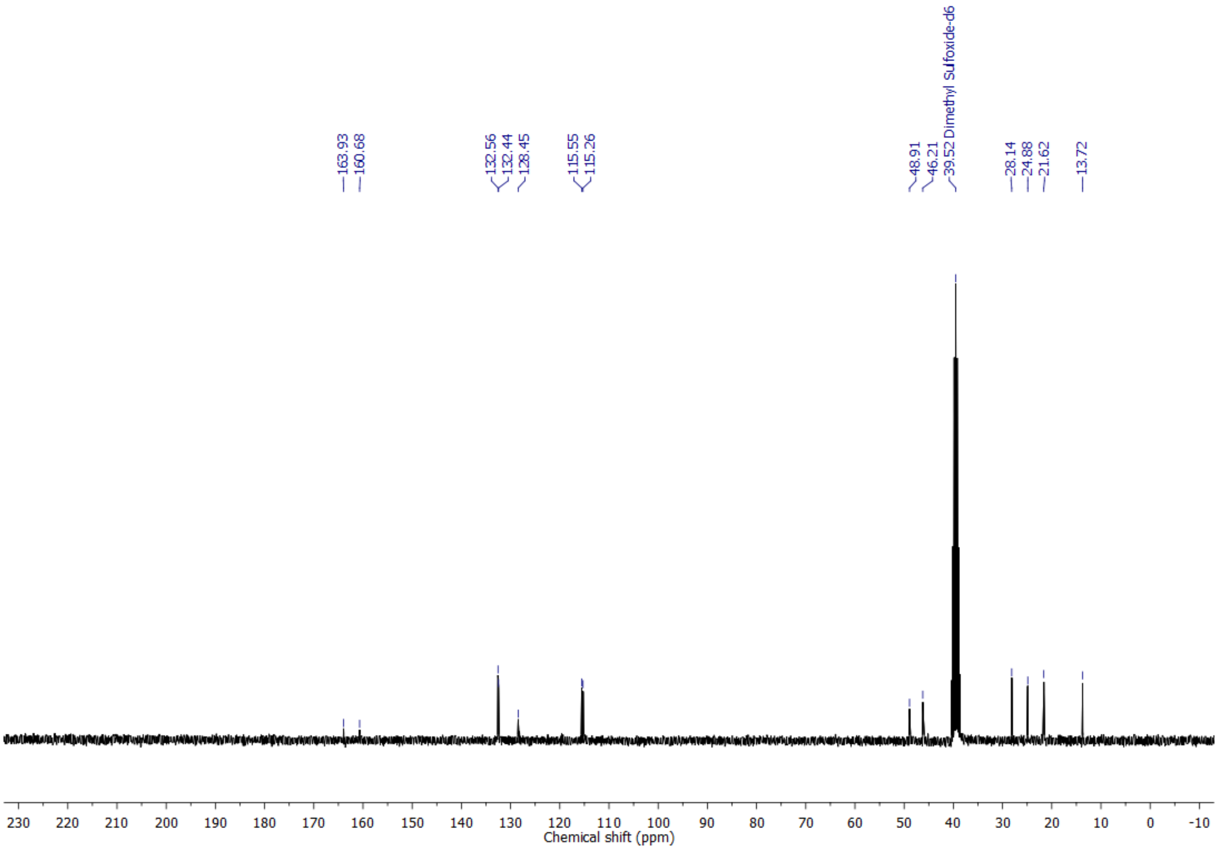

Figure S81: ^{13}C-NMR spectrum of **36**.

***N*-(4-chlorobenzyl)pentan-1-aminium chloride (37)**

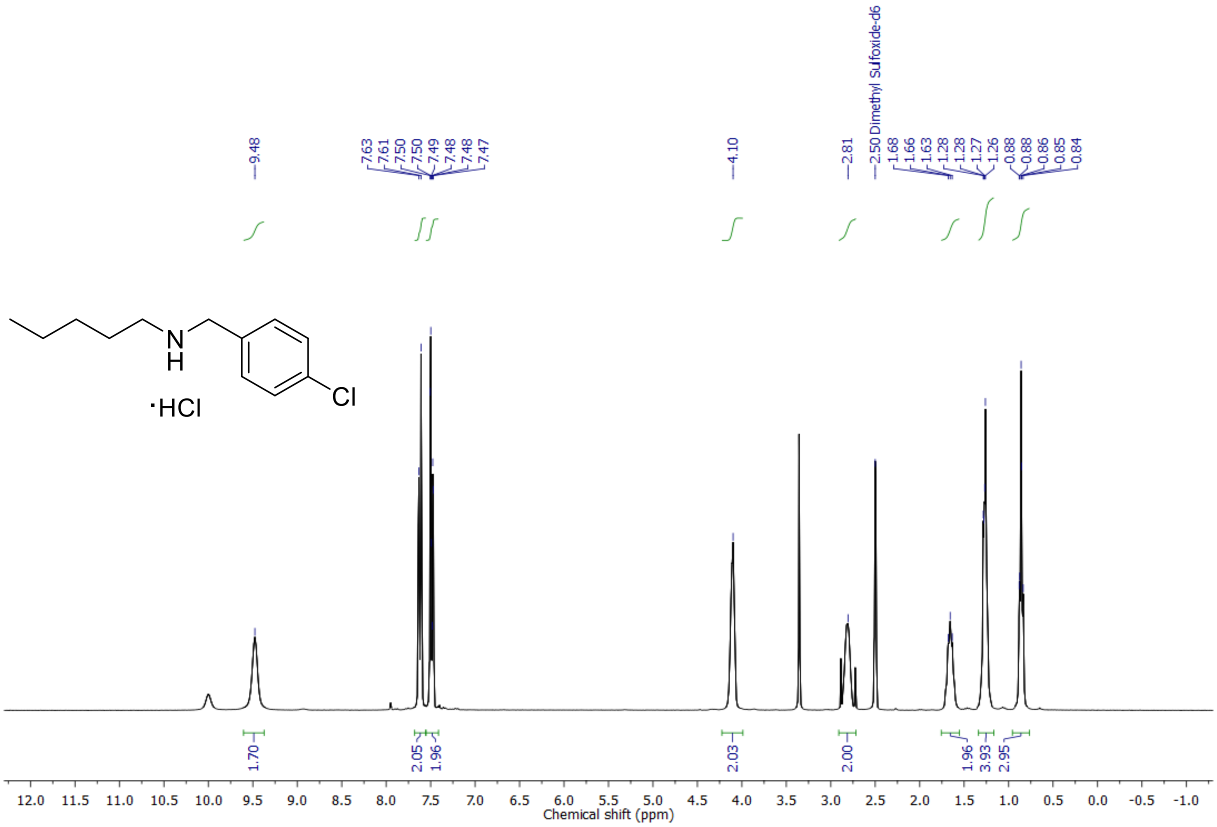

Figure S82: ^{1}H-NMR spectrum of **37**.

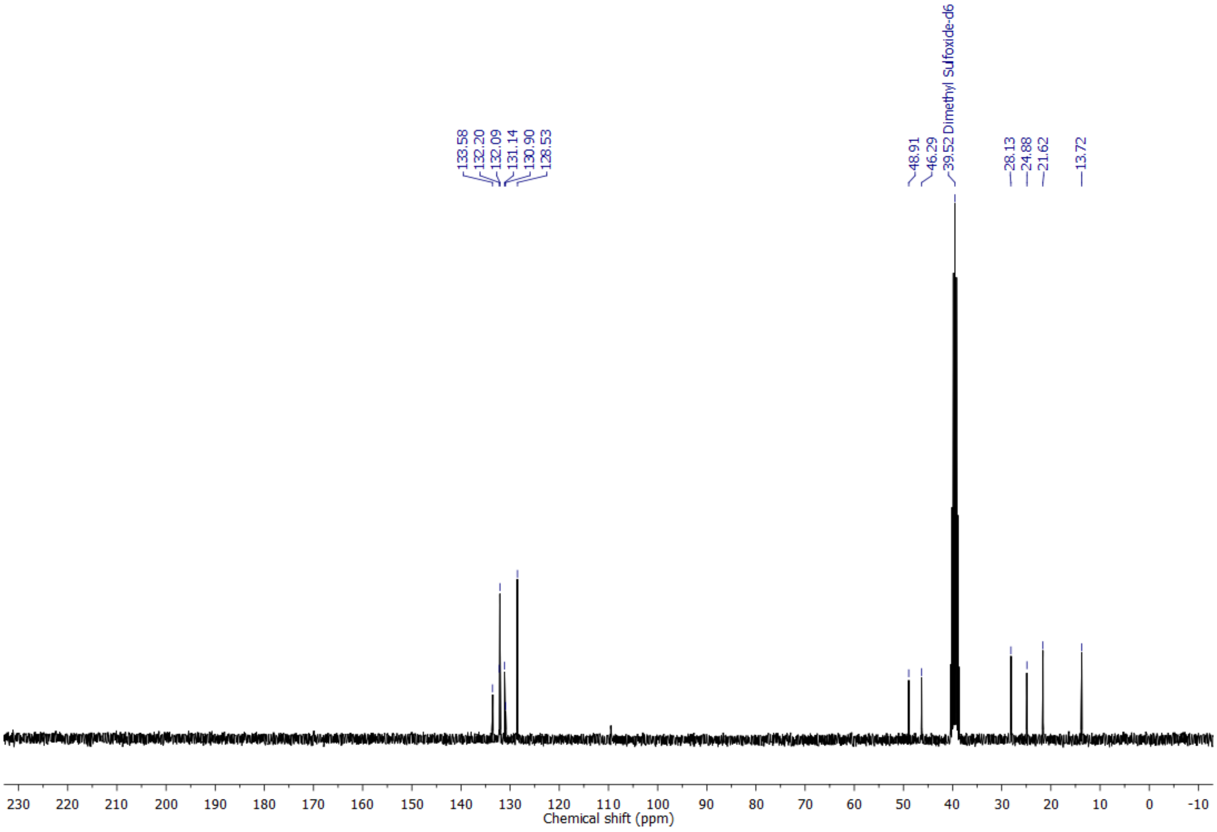

Figure S83: ^{13}C-NMR spectrum of **37**.

N-(3-chlorobenzyl)pentan-1-aminium chloride (38)

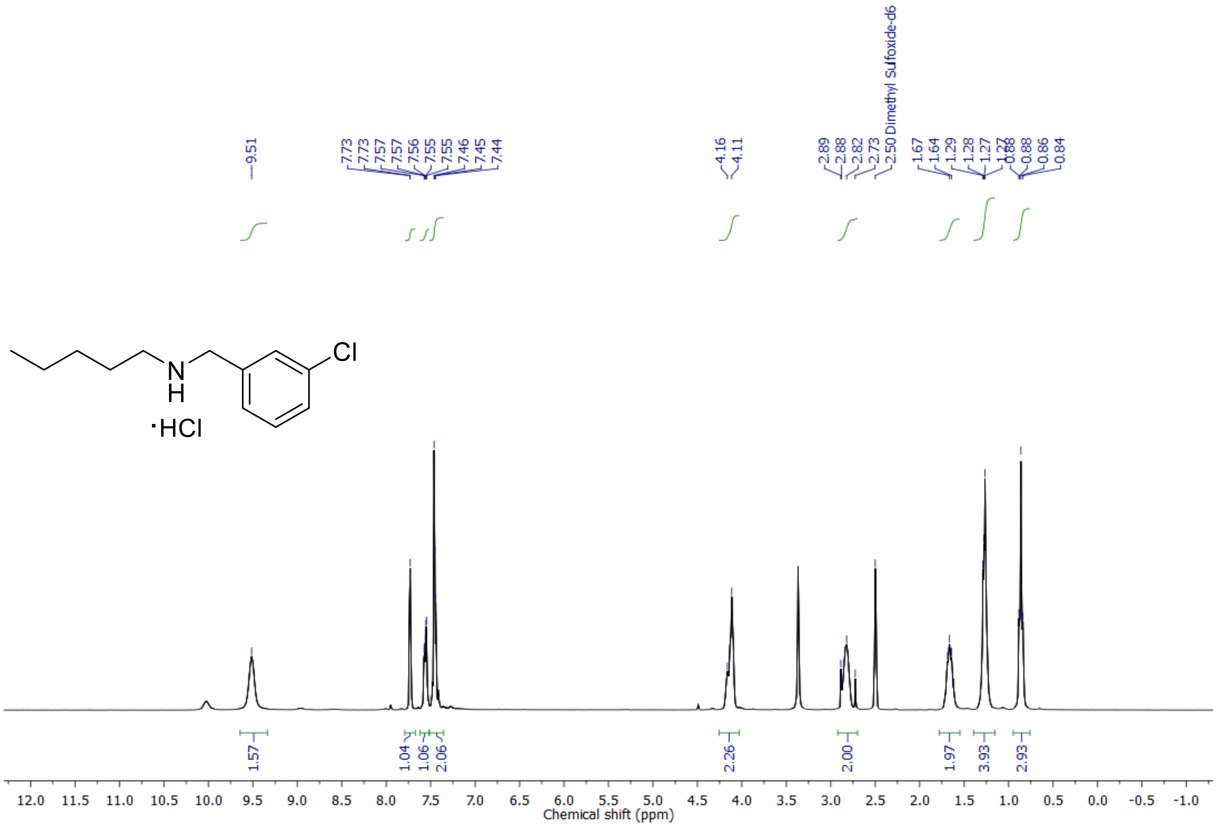

Figure S84: ^{1}H-NMR spectrum of **38**.

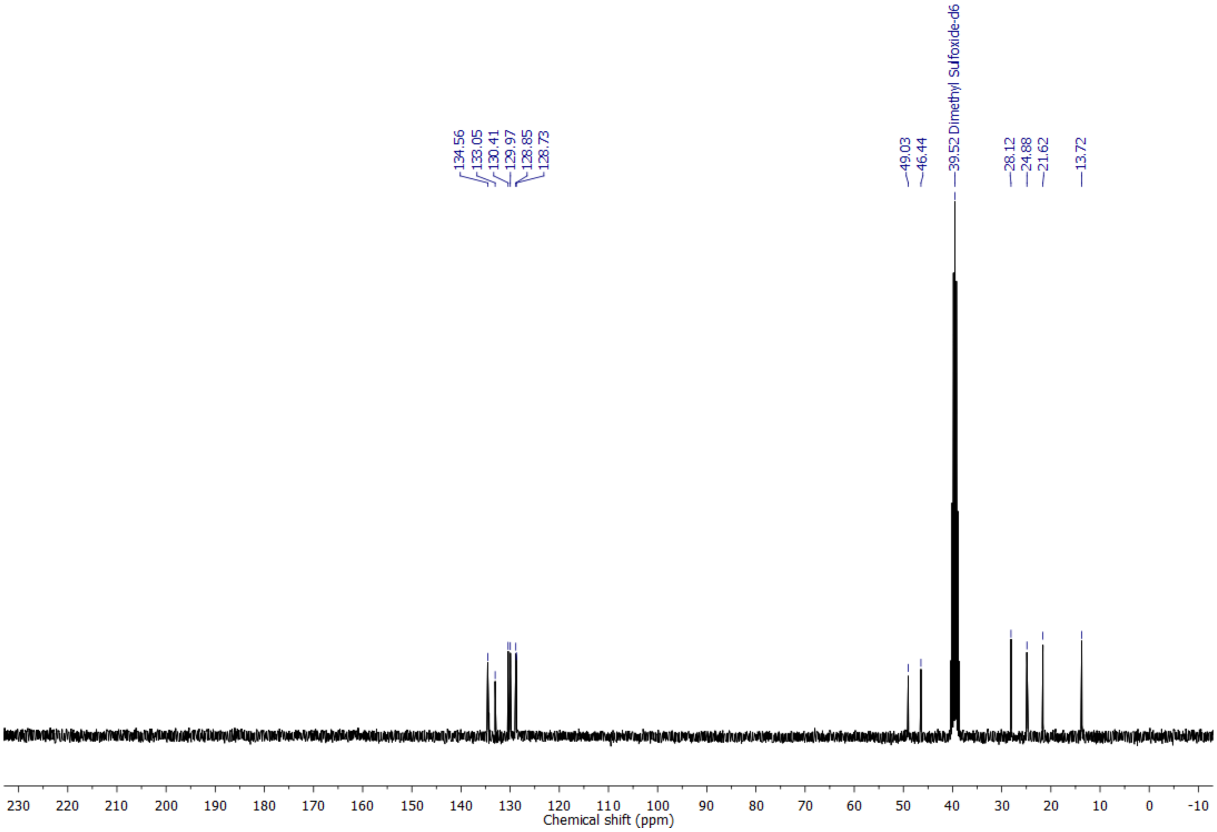

Figure S85: ^{13}C-NMR spectrum of **38**.

N-(2-chlorobenzyl)pentan-1-aminium chloride (39)

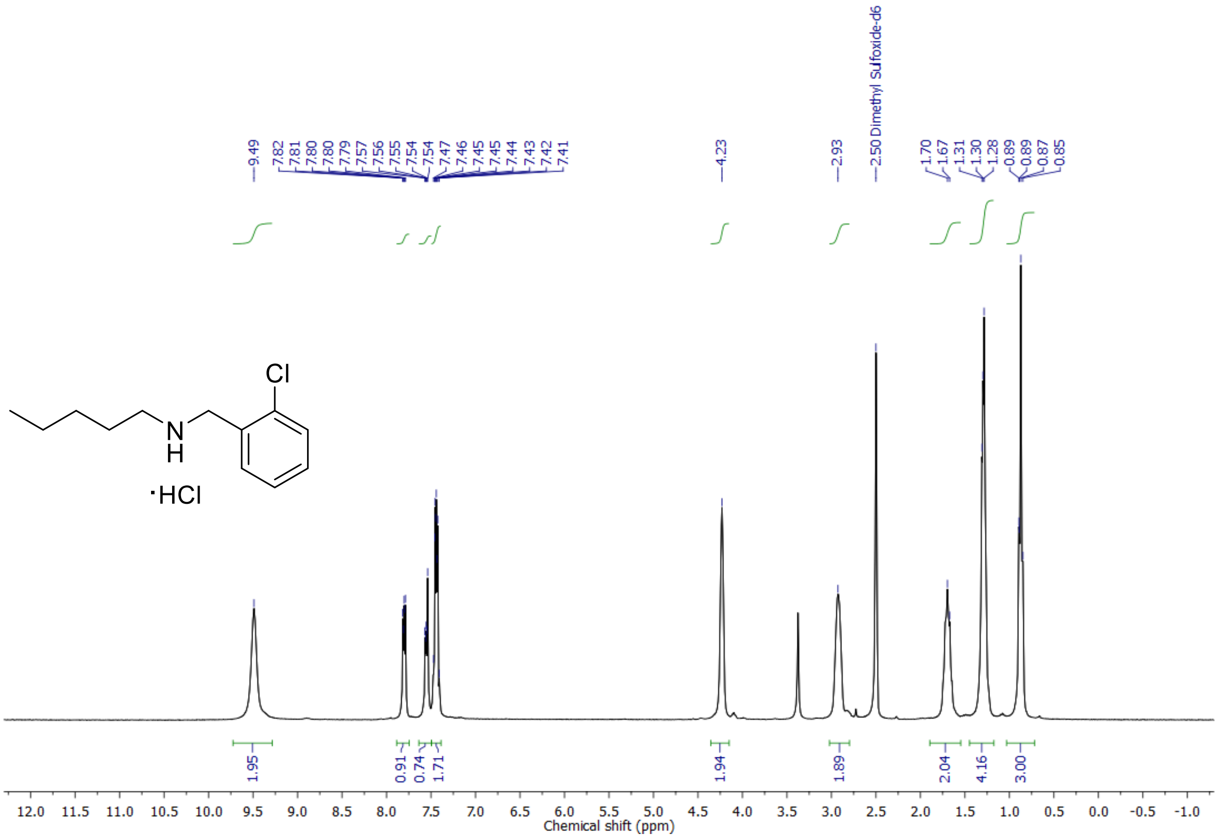

Figure S86: ^{1}H-NMR spectrum of **39**.

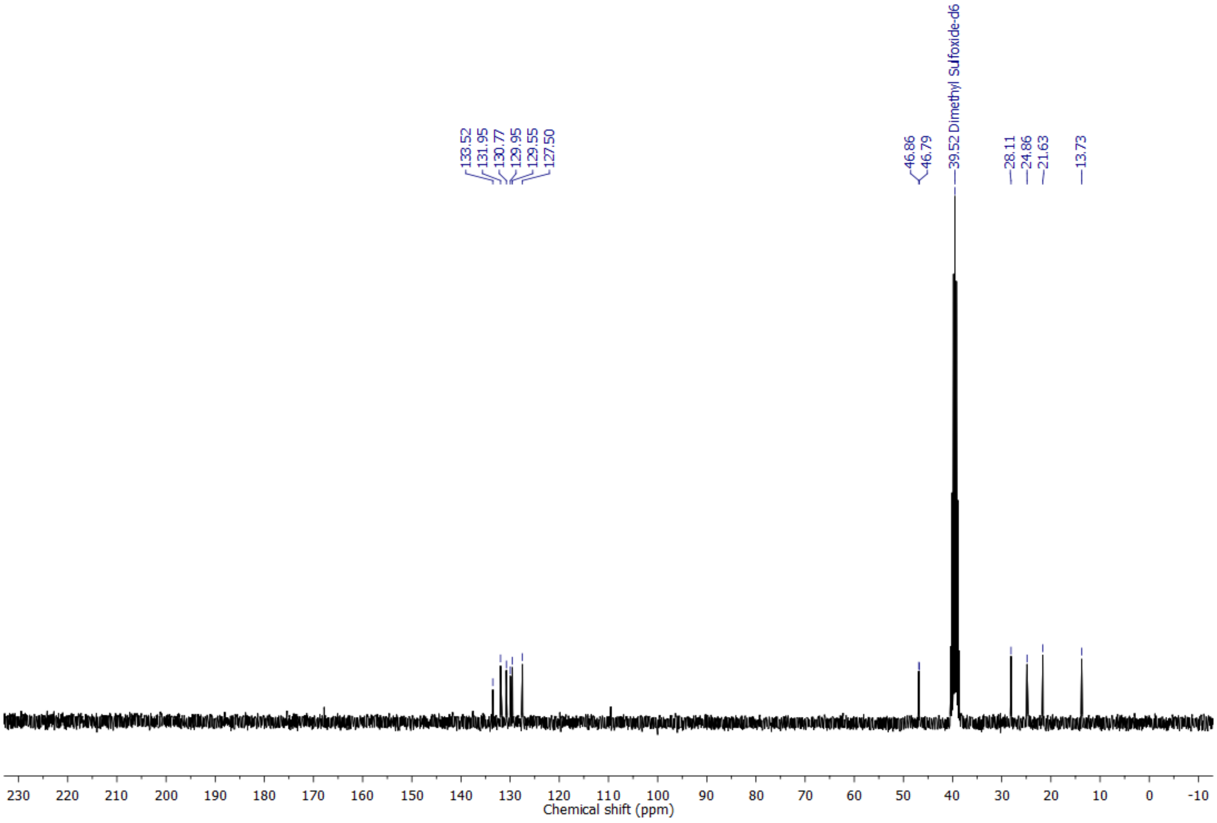

Figure S87: ^{13}C-NMR spectrum of **39**.

***N*-(4-bromobenzyl)pentan-1-aminium chloride (40)**

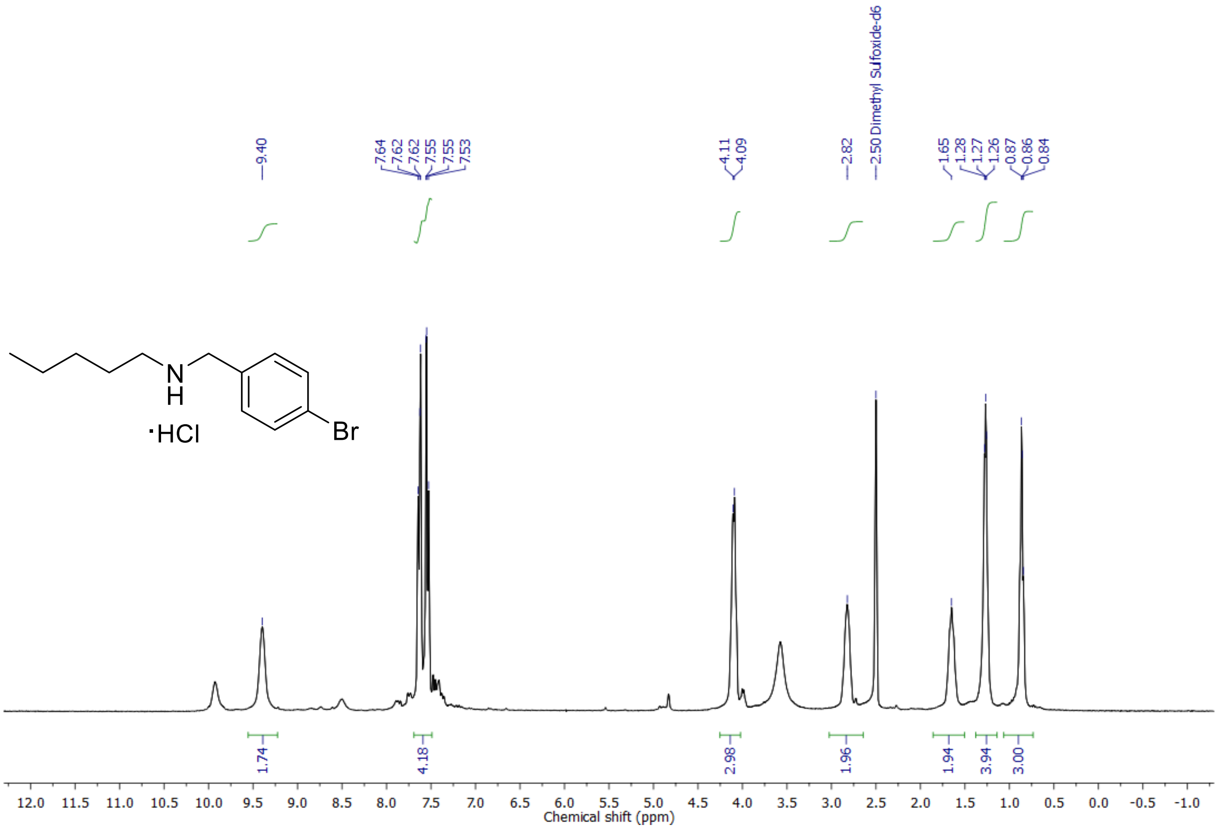

Figure S88: ^{1}H-NMR spectrum of **40**.

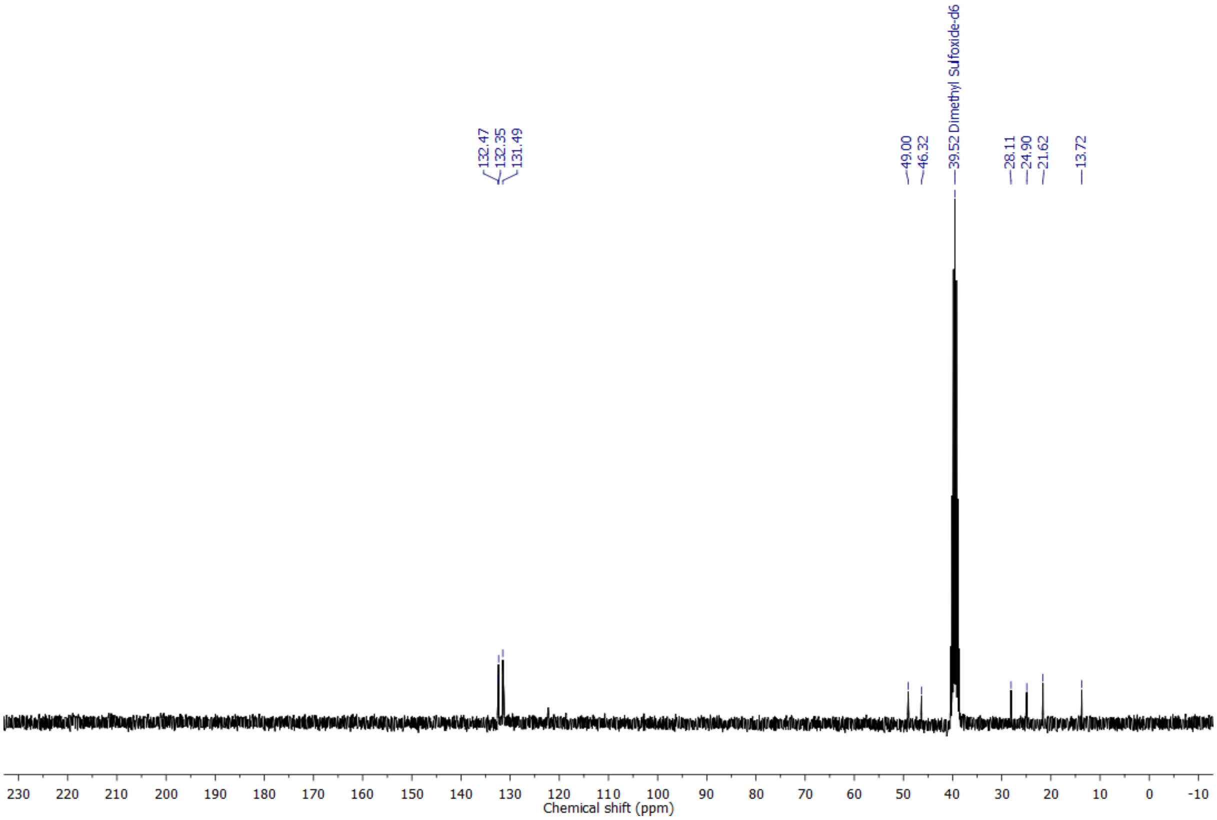

Figure S89: ^{13}C-NMR spectrum of **40**.

N-(4-methoxybenzyl)pentan-1-aminium chloride (41)

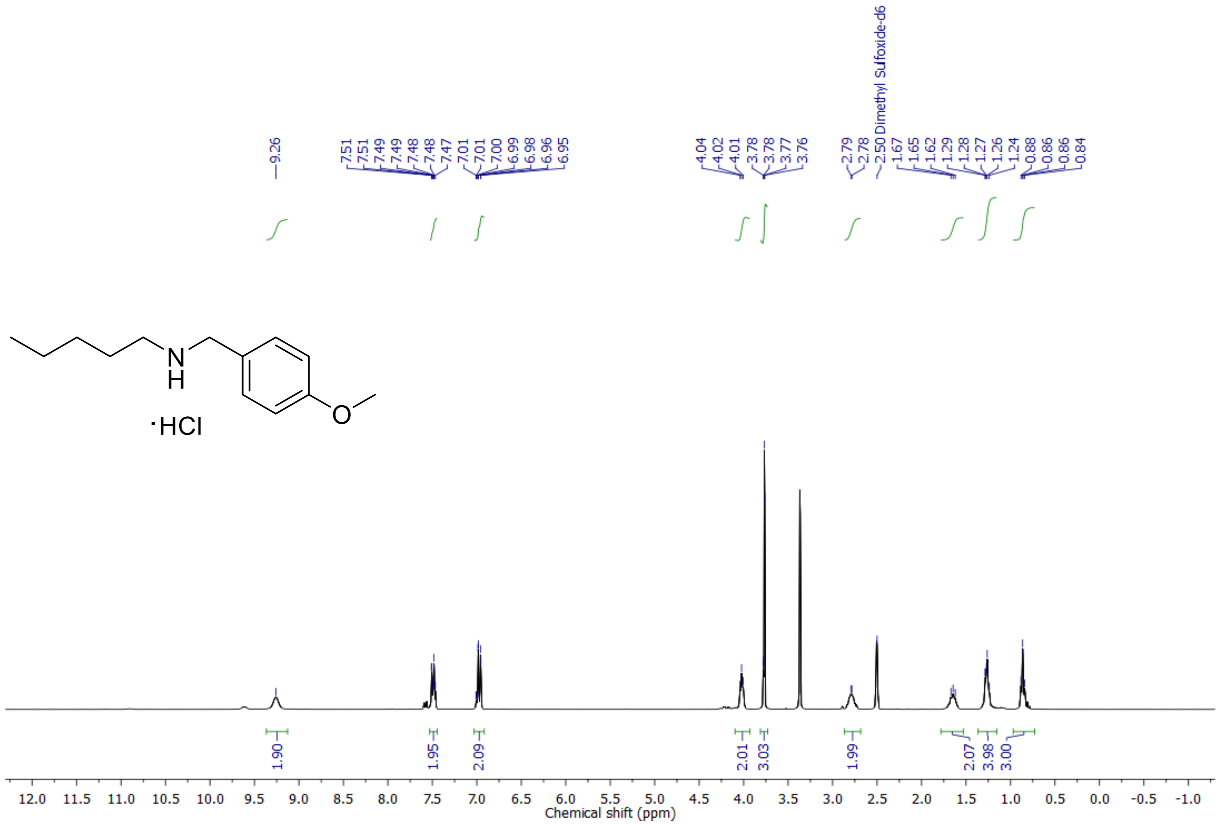

Figure S90: ^{1}H-NMR spectrum of **41**.

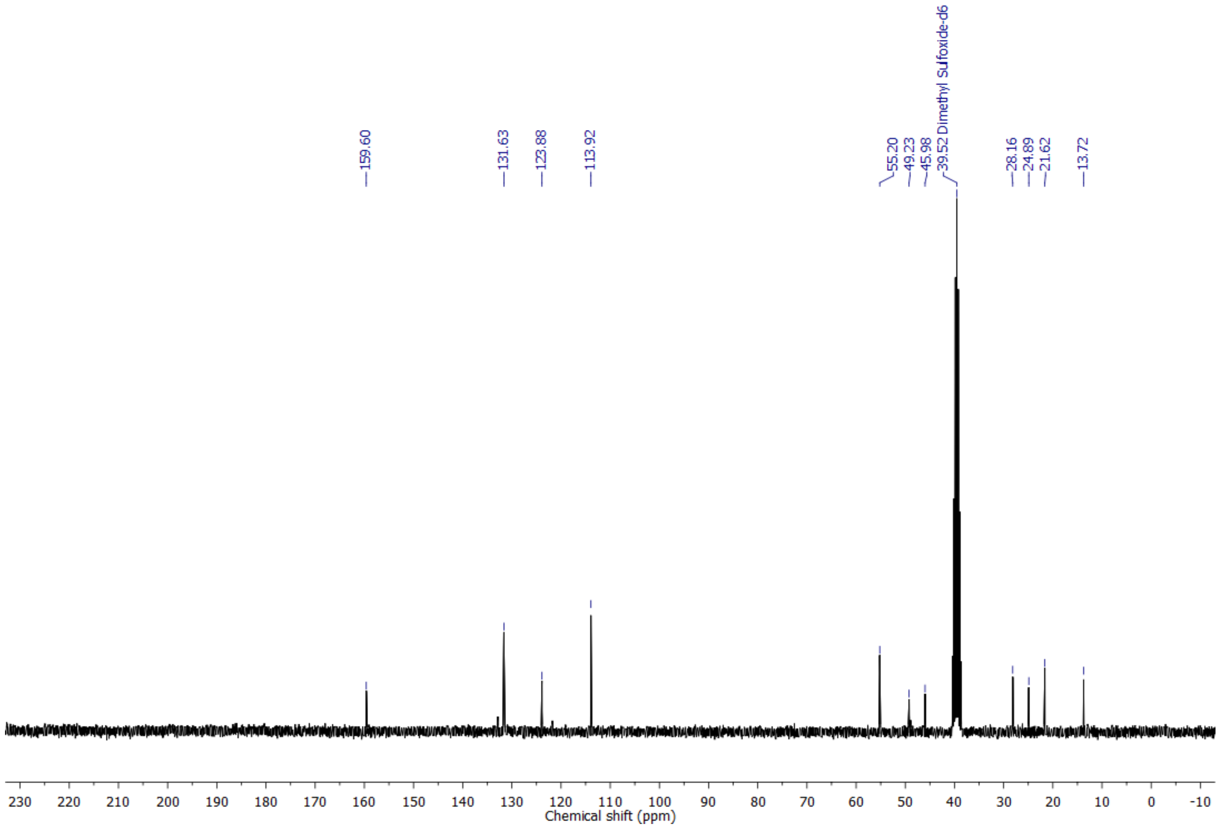

Figure S91: ^{13}C-NMR spectrum of **41**.

N-(4-(benzyloxy)benzyl)pentan-1-aminium chloride (42)

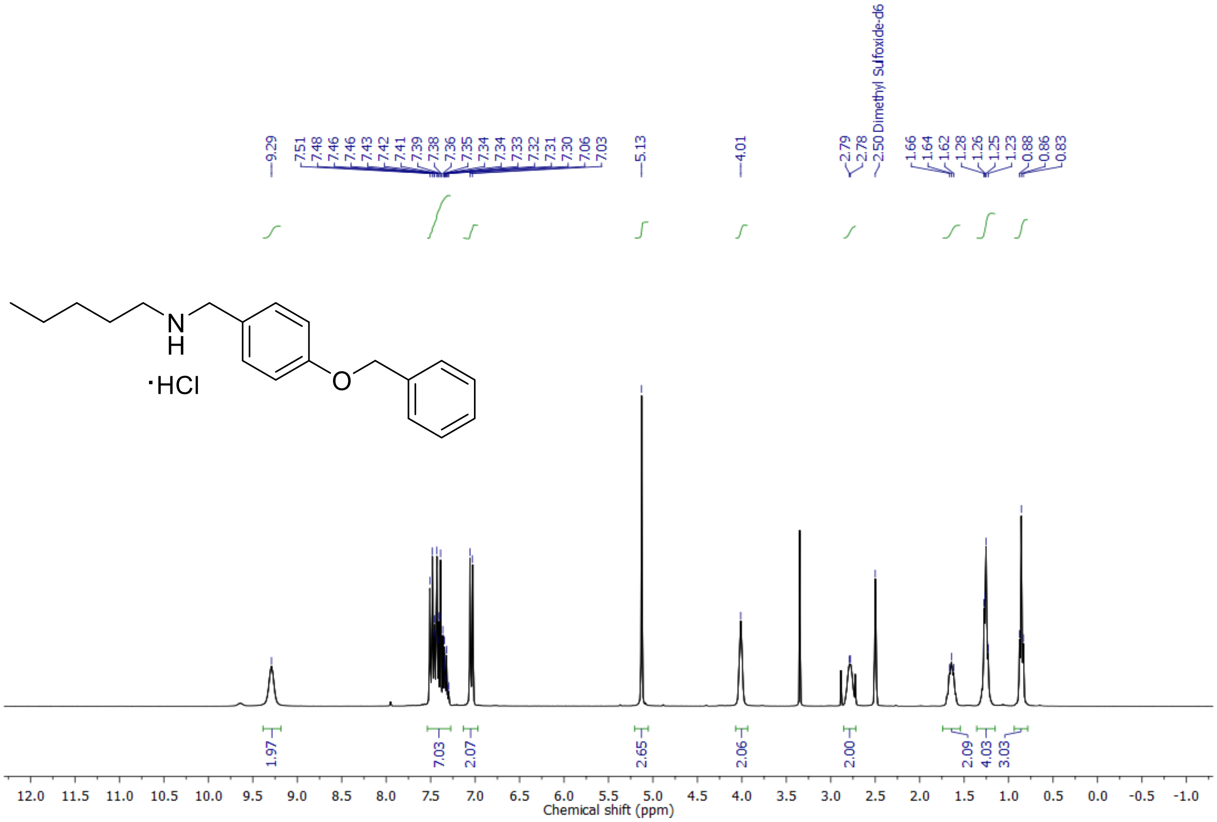

Figure S92: ^{1}H-NMR spectrum of **42**.

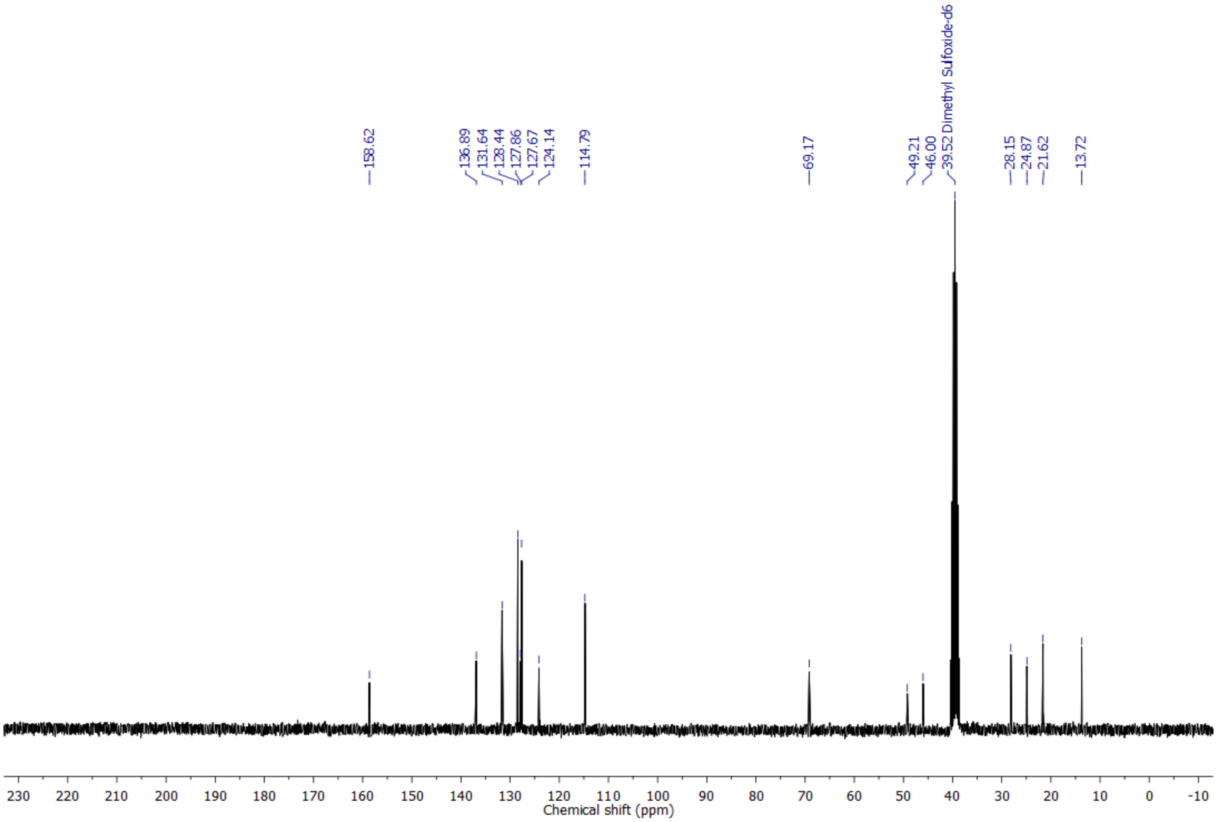

Figure S93: ^{13}C-NMR spectrum of **42**.

***N*-(benzo[d][1,3]dioxol-5-ylmethyl)pentan-1-aminium chloride (43)**

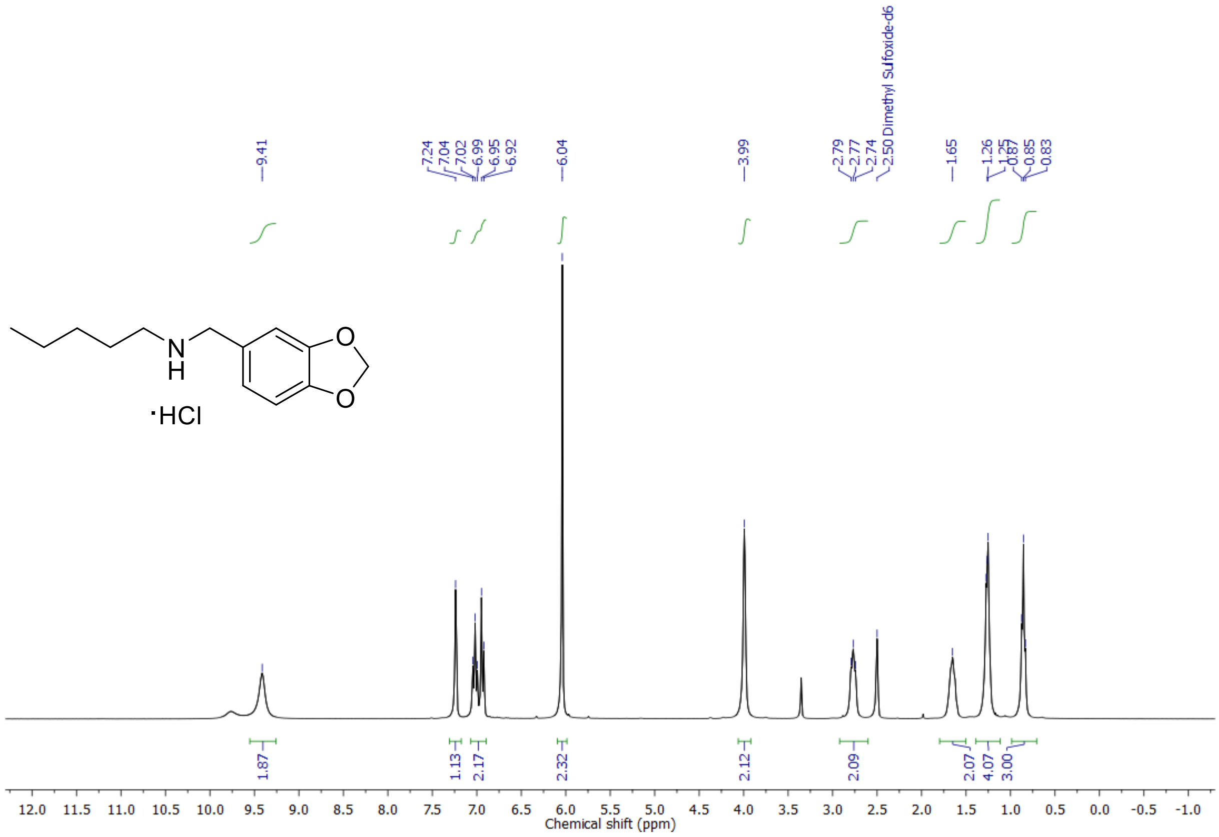

Figure S94: ^{1}H-NMR spectrum of **43**.

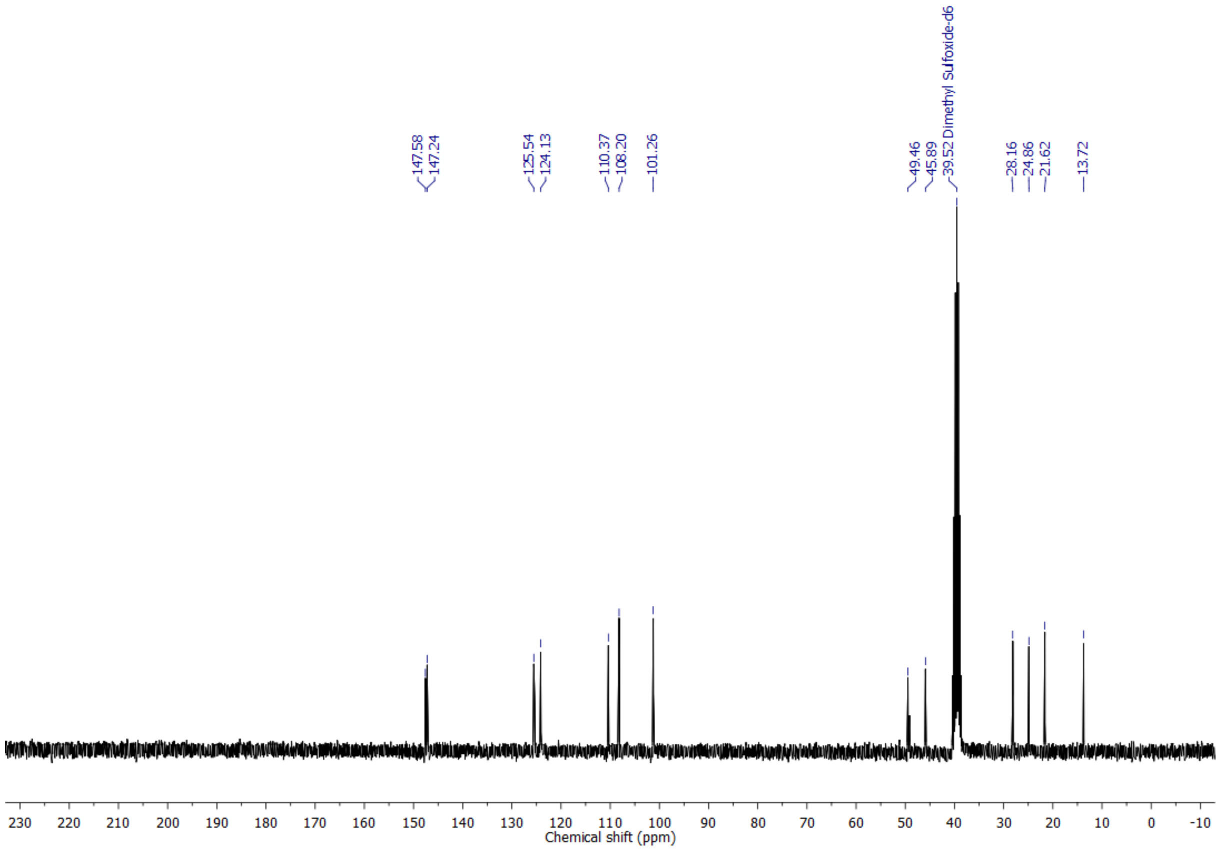

Figure S95: ^{13}C-NMR spectrum of **43**.

***N*-(4-(tert-butyl)benzyl)pentan-1-aminium chloride (44)**

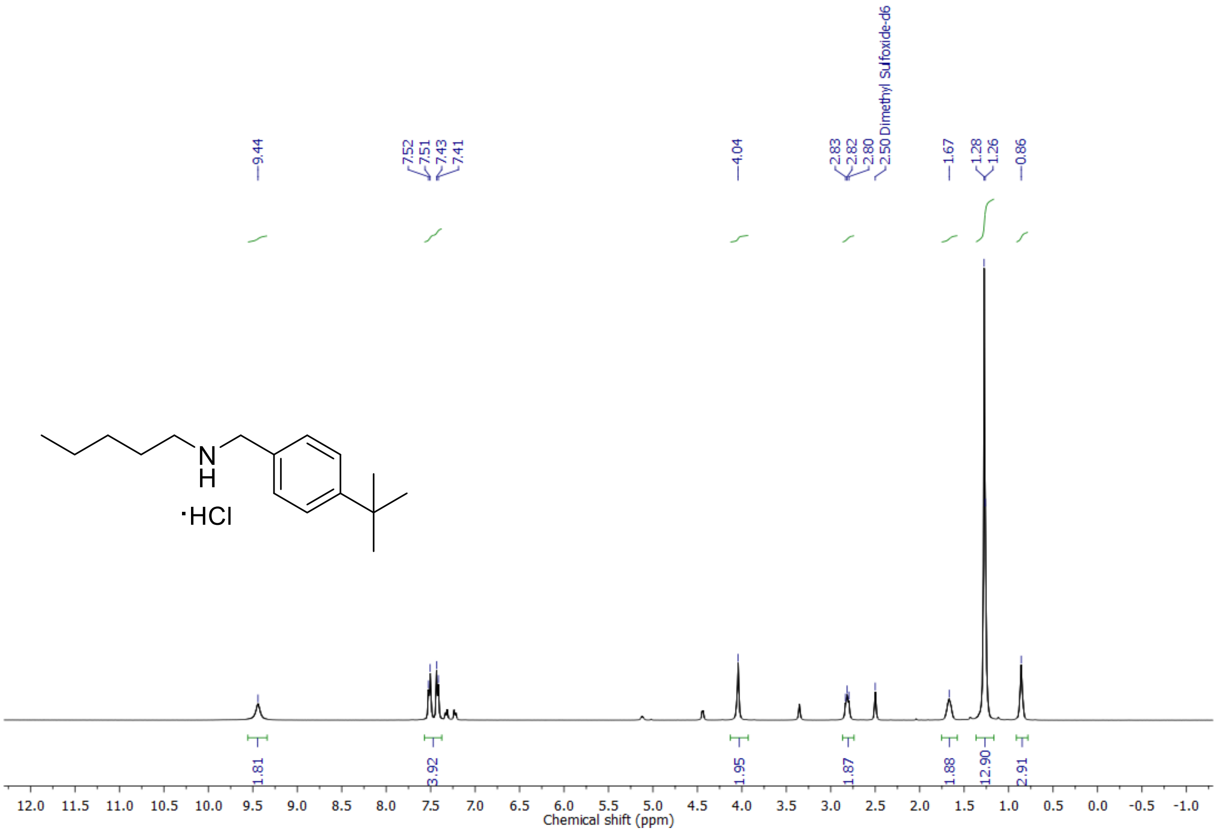

Figure S96: ^{1}H-NMR spectrum of **44**.

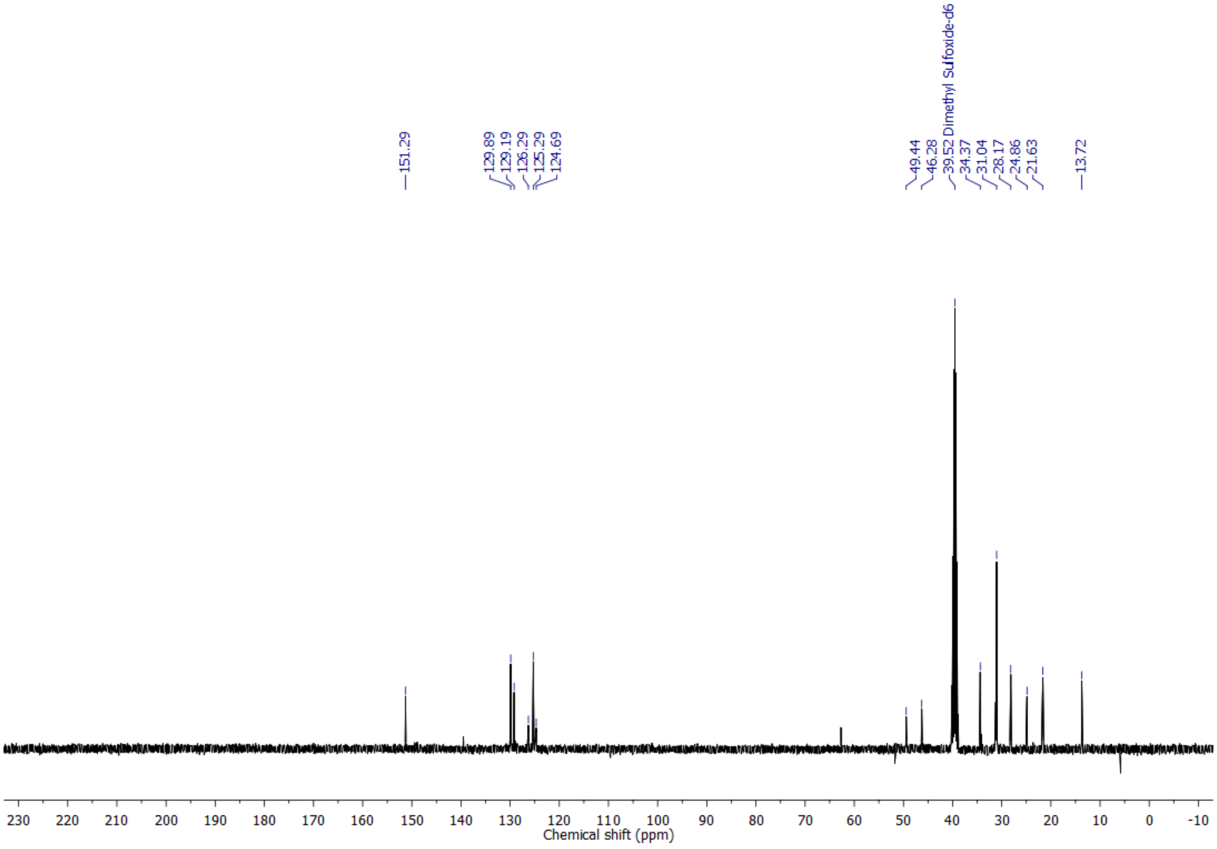

Figure S97: ^{13}C-NMR spectrum of **44**.

N-benzylbutan-1-aminium chloride (45)

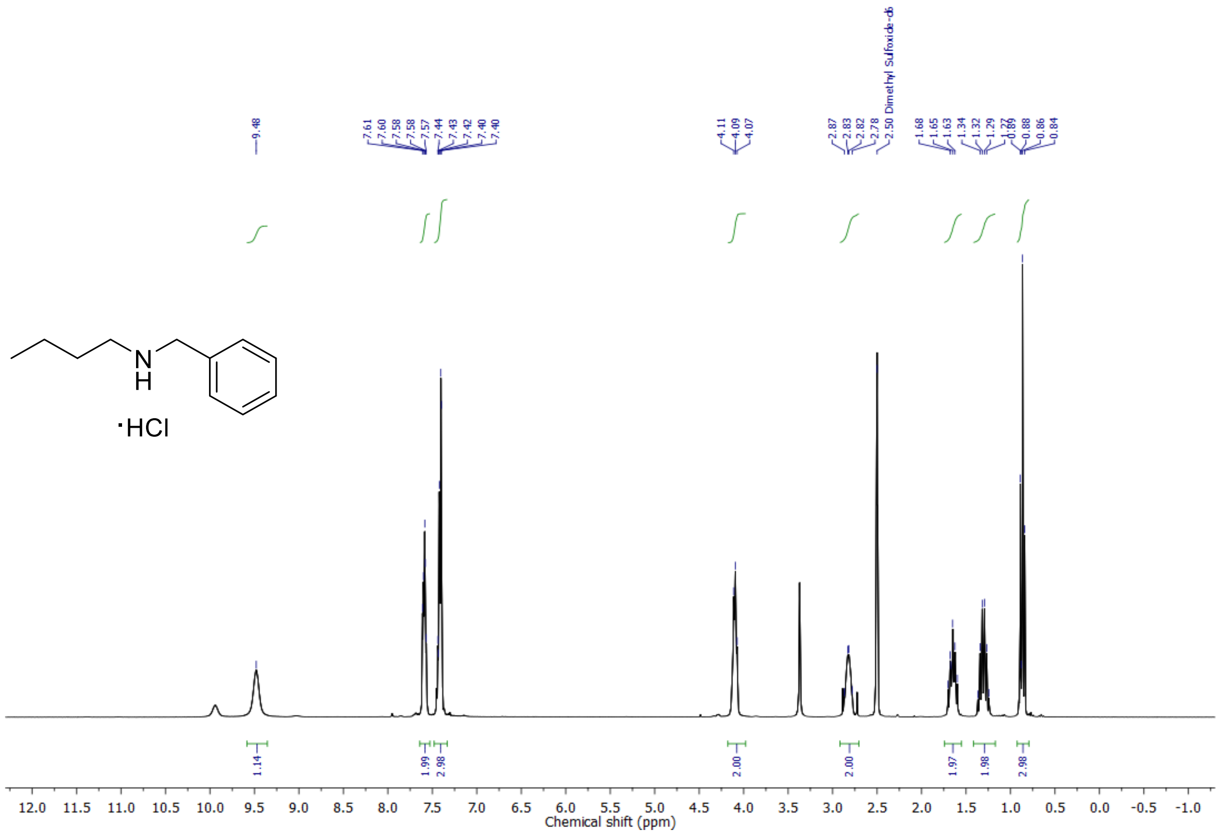

Figure S98: ^{1}H-NMR spectrum of **45**.

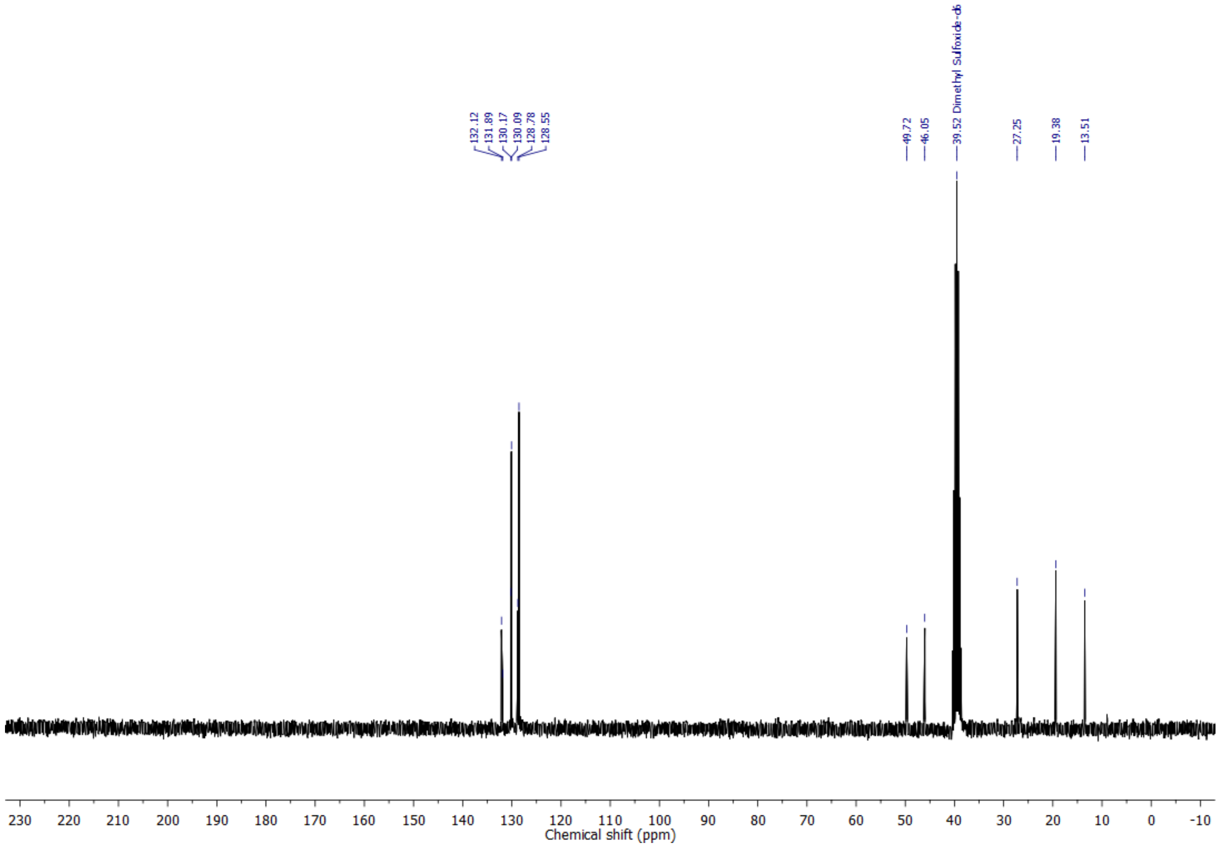

Figure S99: ^{13}C-NMR spectrum of **45**.

N-benzylpentanaminium chloride (46)

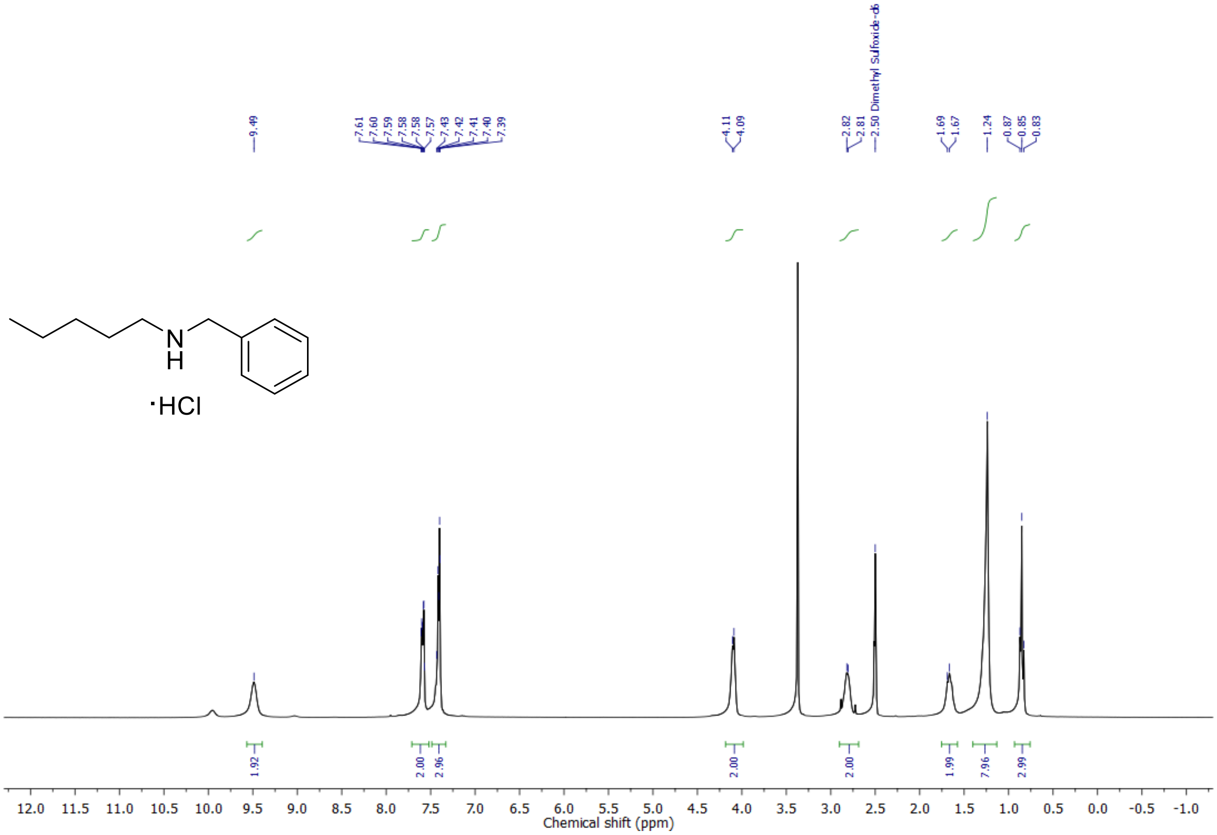

Figure S100: ^{1}H-NMR spectrum of **46**.

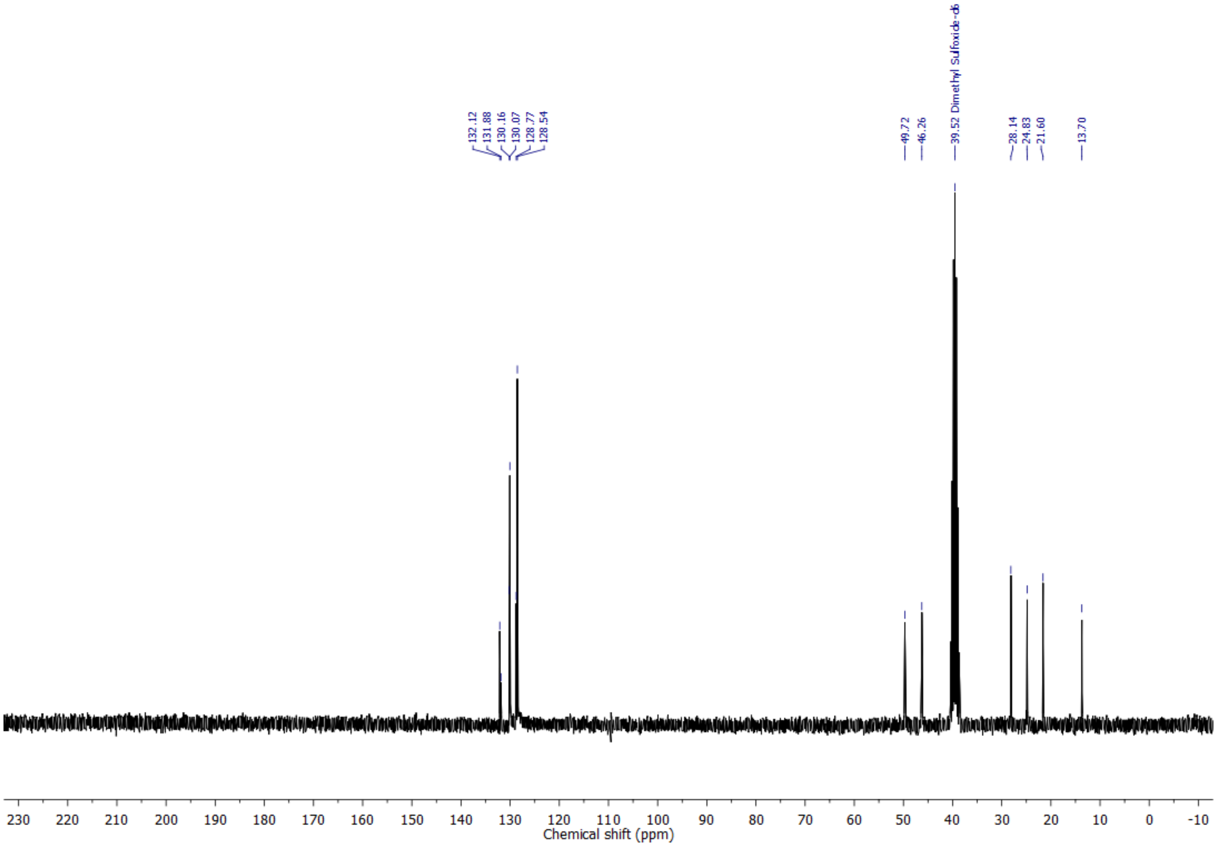

Figure S101: ^{13}C-NMR spectrum of **46**.

N-benzyl-1-cyclohexylmethanaminium chloride (47)

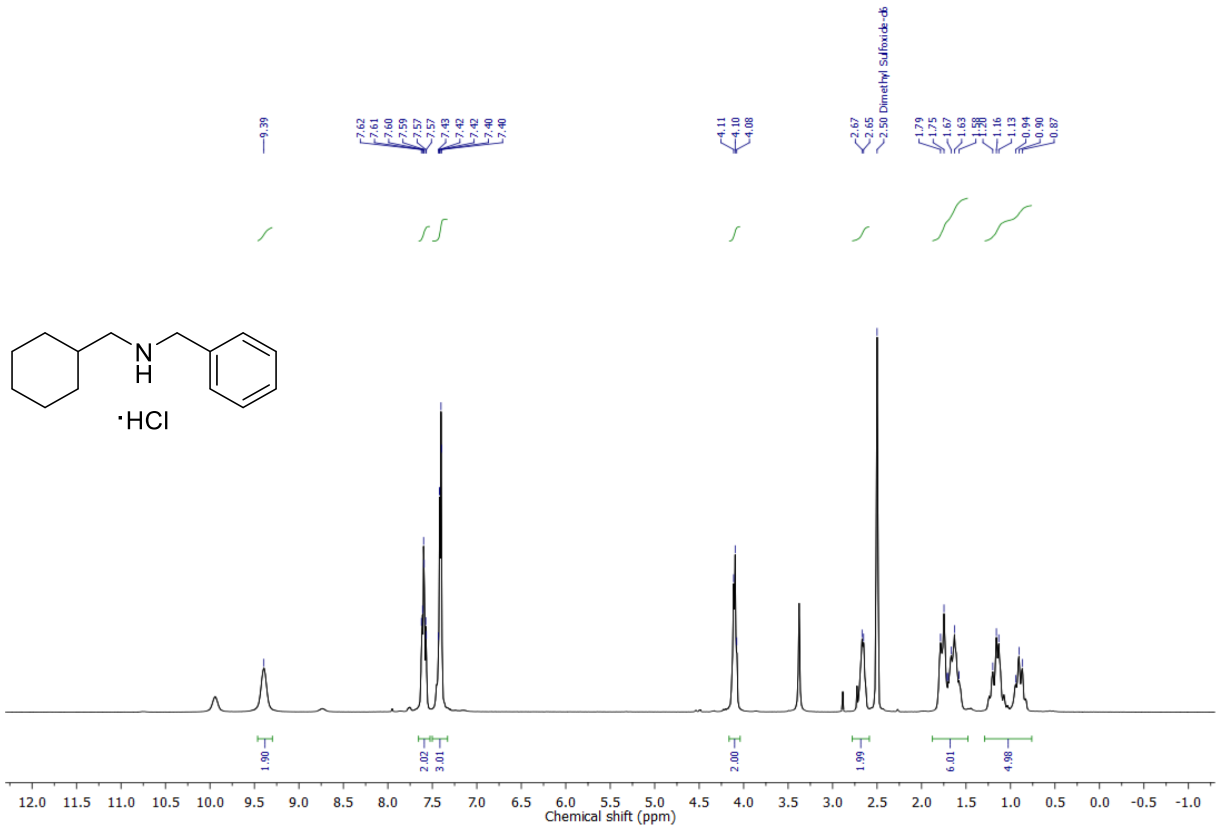

Figure S102: ^{1}H-NMR spectrum of **47**.

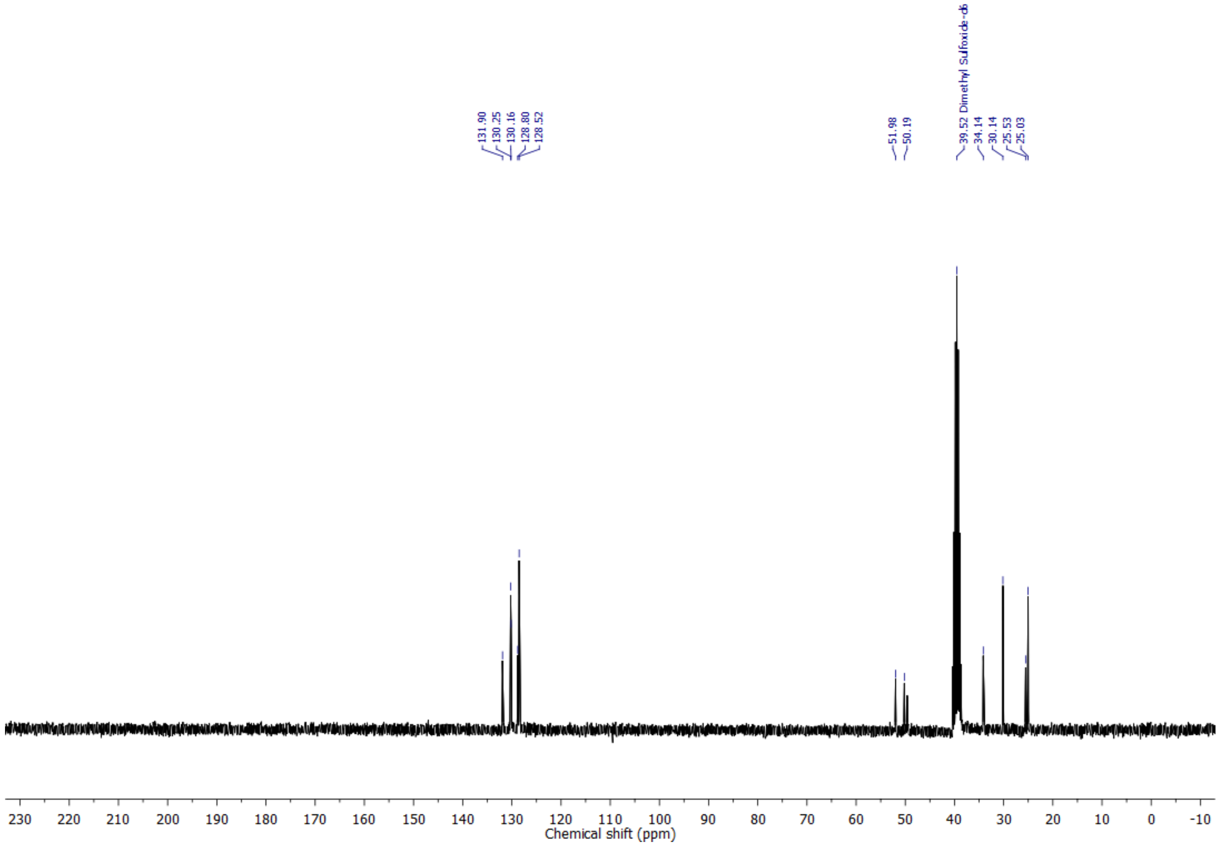

Figure S103: ^{13}C-NMR spectrum of **47**.

N-benzyl-2,2-dimethylpropan-1-aminium chloride (48)

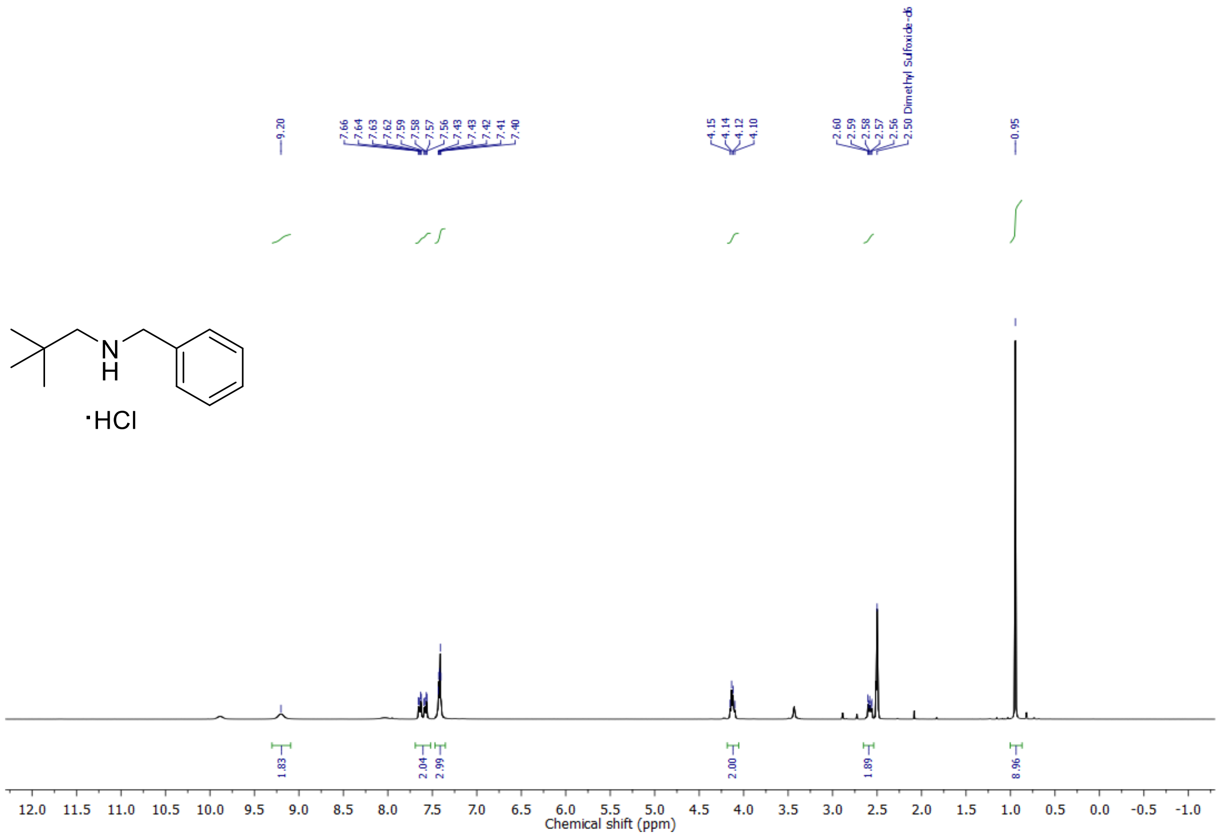

Figure S104: ^{1}H-NMR spectrum of **48**.

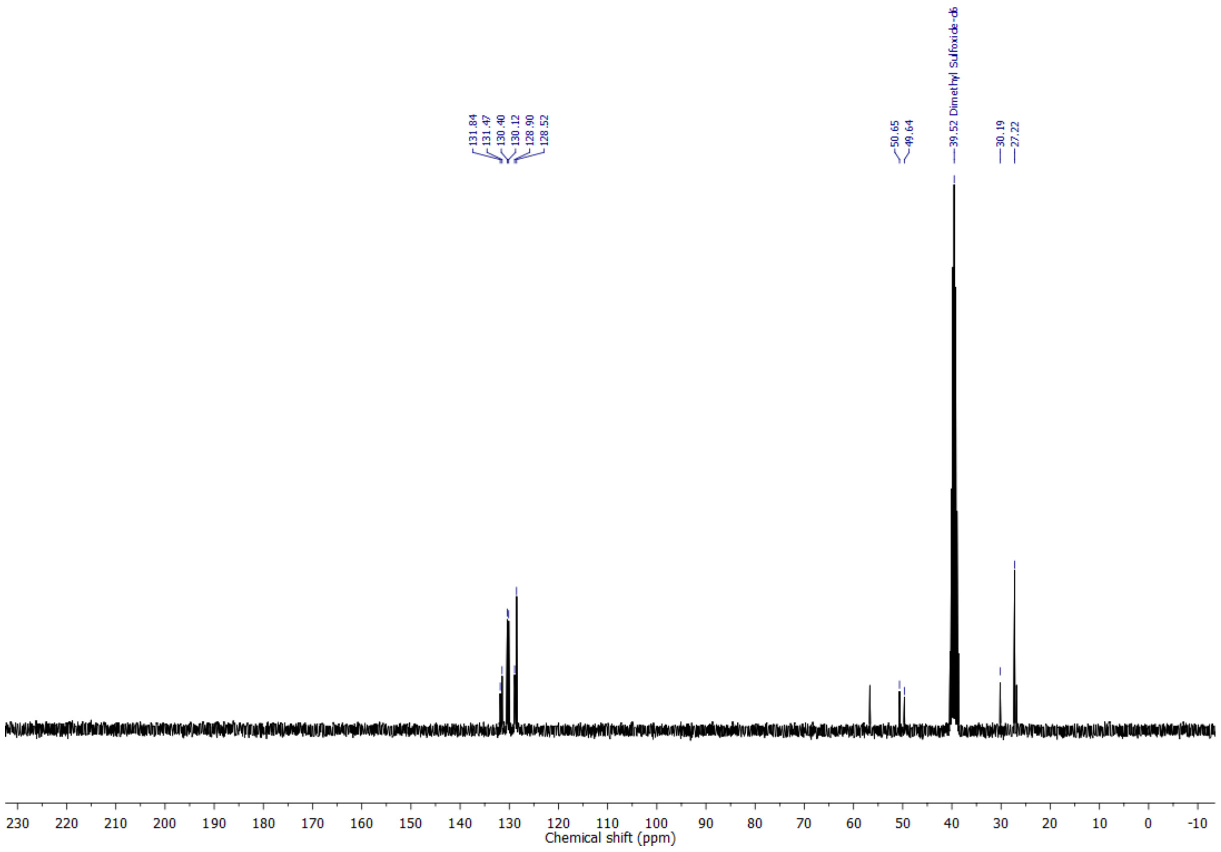

Figure S105: ^{13}C-NMR spectrum of **48**.

3,7-dimethyl-N-pentyloct-6-en-1-aminium chloride (49)

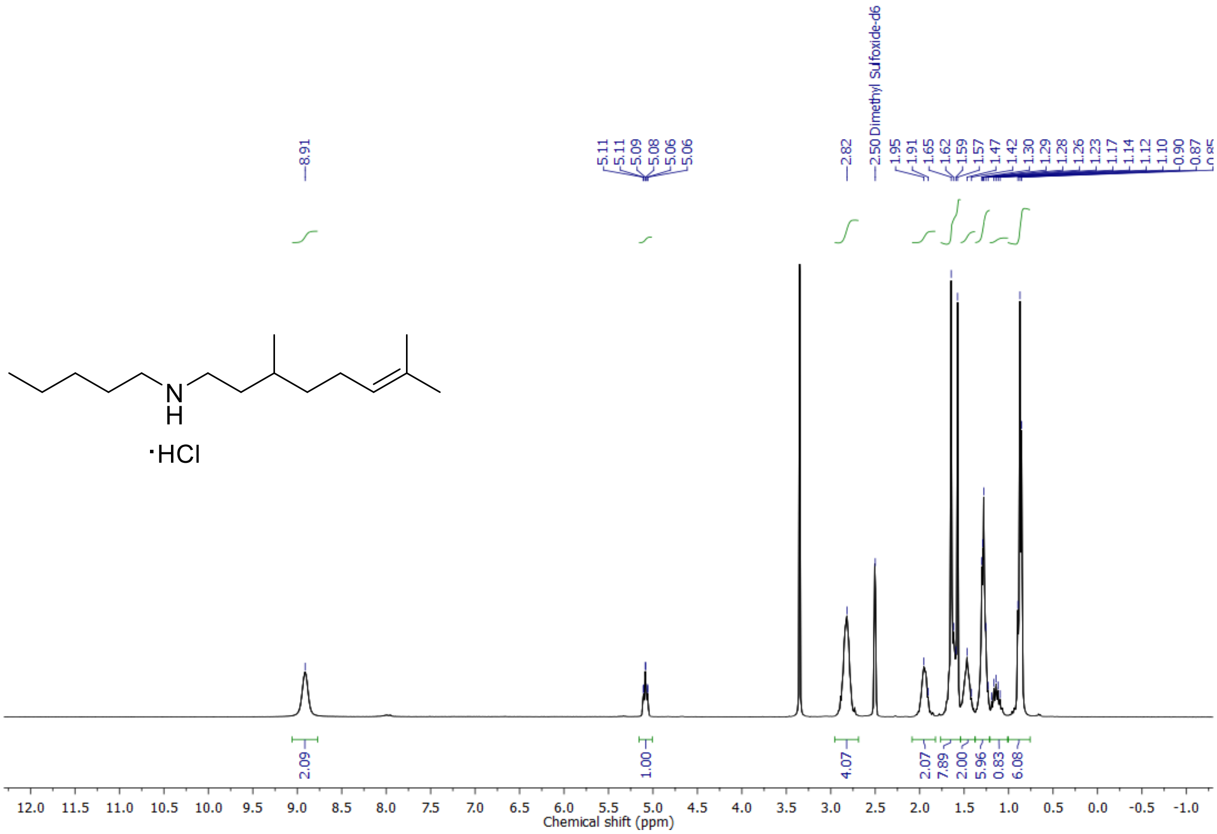

Figure S106: ^{1}H-NMR spectrum of **49**.

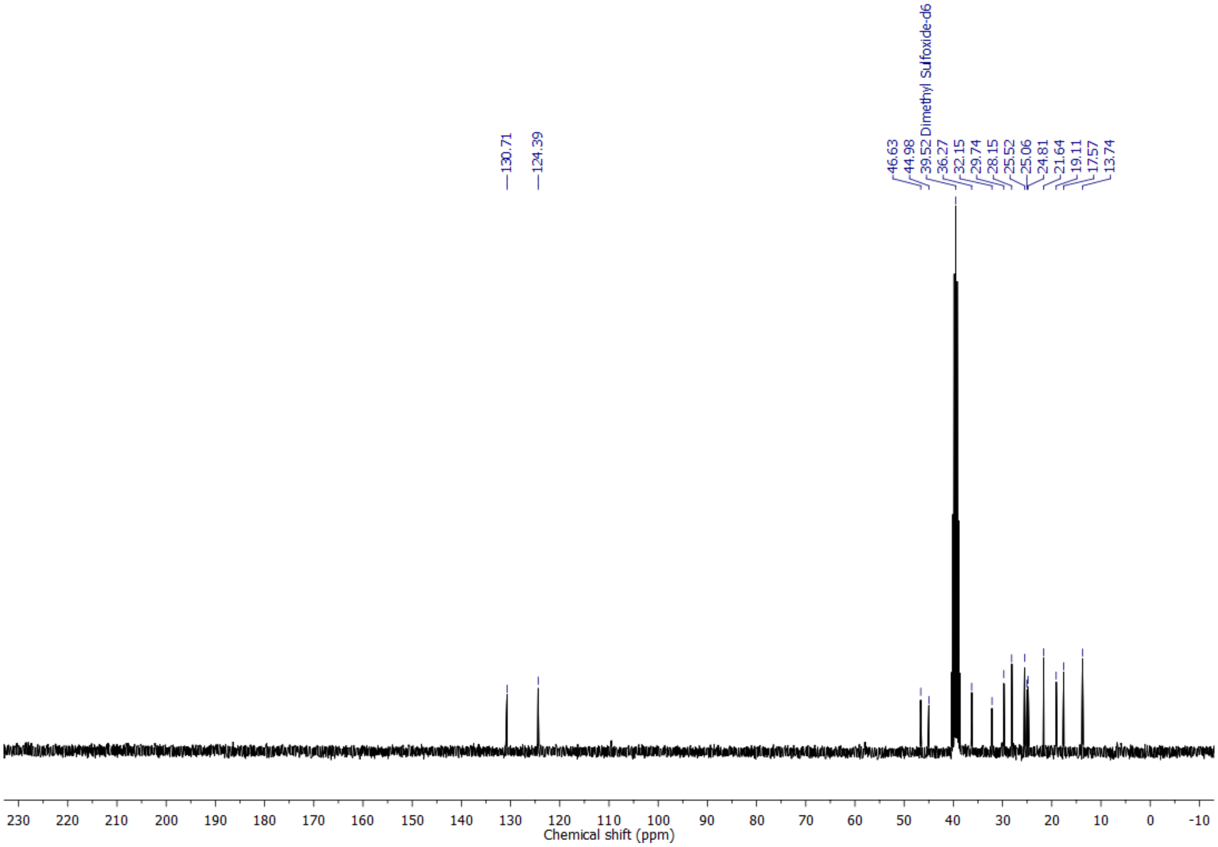

Figure S107: ^{13}C-NMR spectrum of **49**.

N-butyldodecan-1-aminium chloride (50)

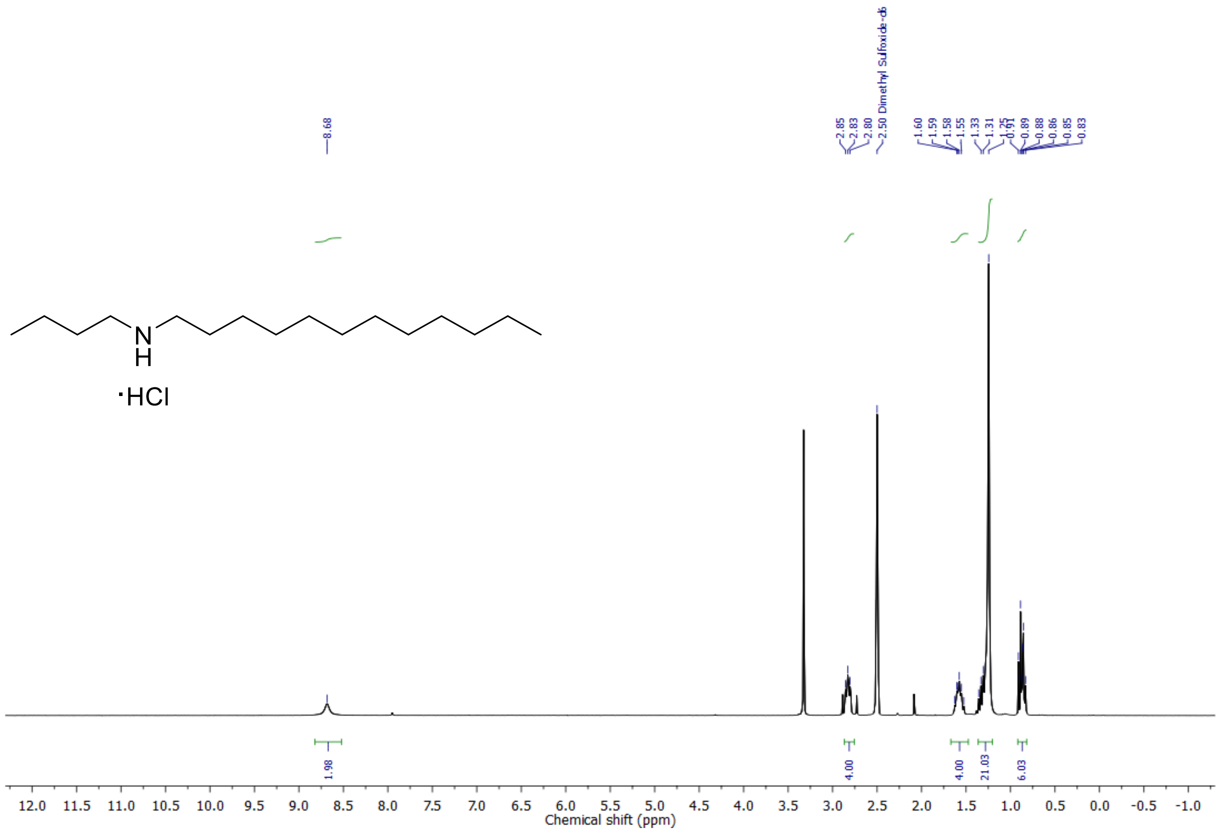

Figure S108: ^{1}H-NMR spectrum of **50**.

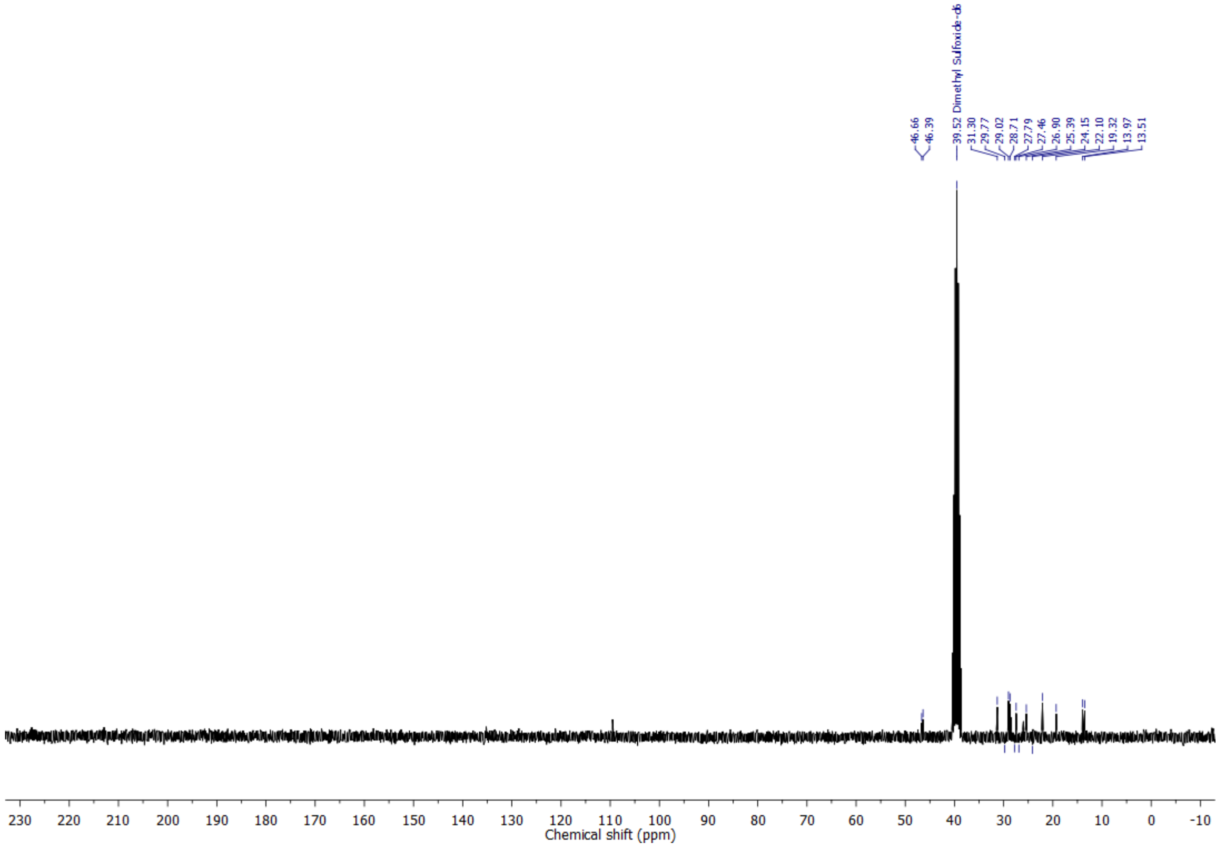

Figure S109: ^{13}C-NMR spectrum of **50**.

Dipentylaminium chloride (51)

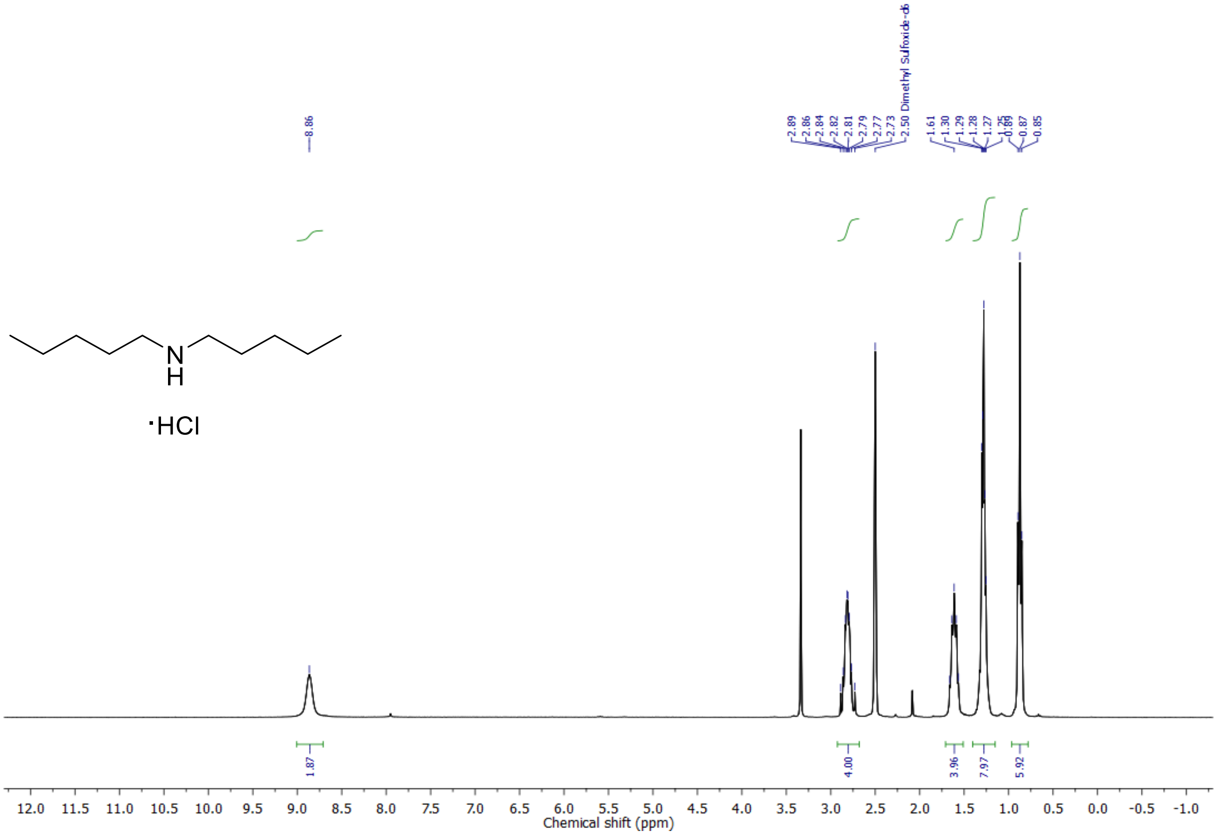

Figure S110: ^{1}H-NMR spectrum of **51**.

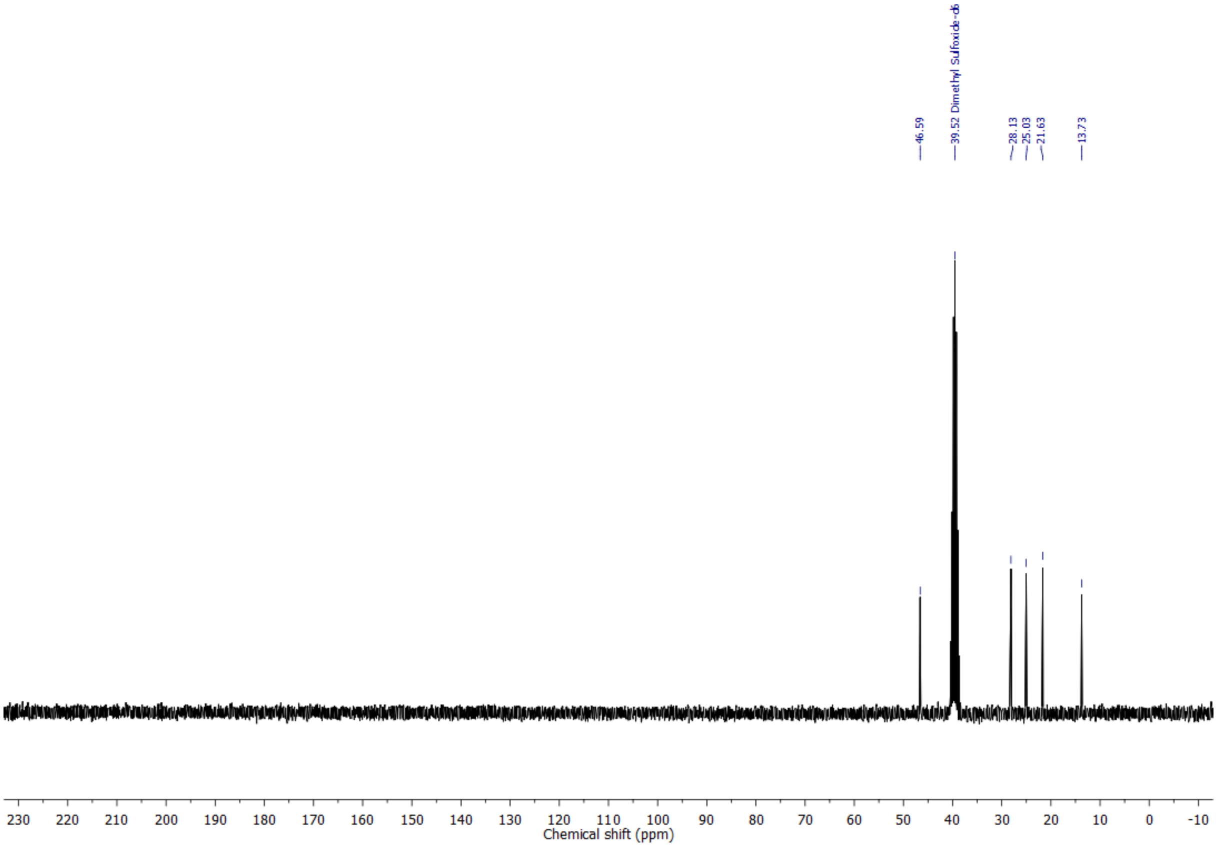

Figure S111: ^{13}C-NMR spectrum of **51**.

_N_-pentylheptan-1-aminium chloride (52)

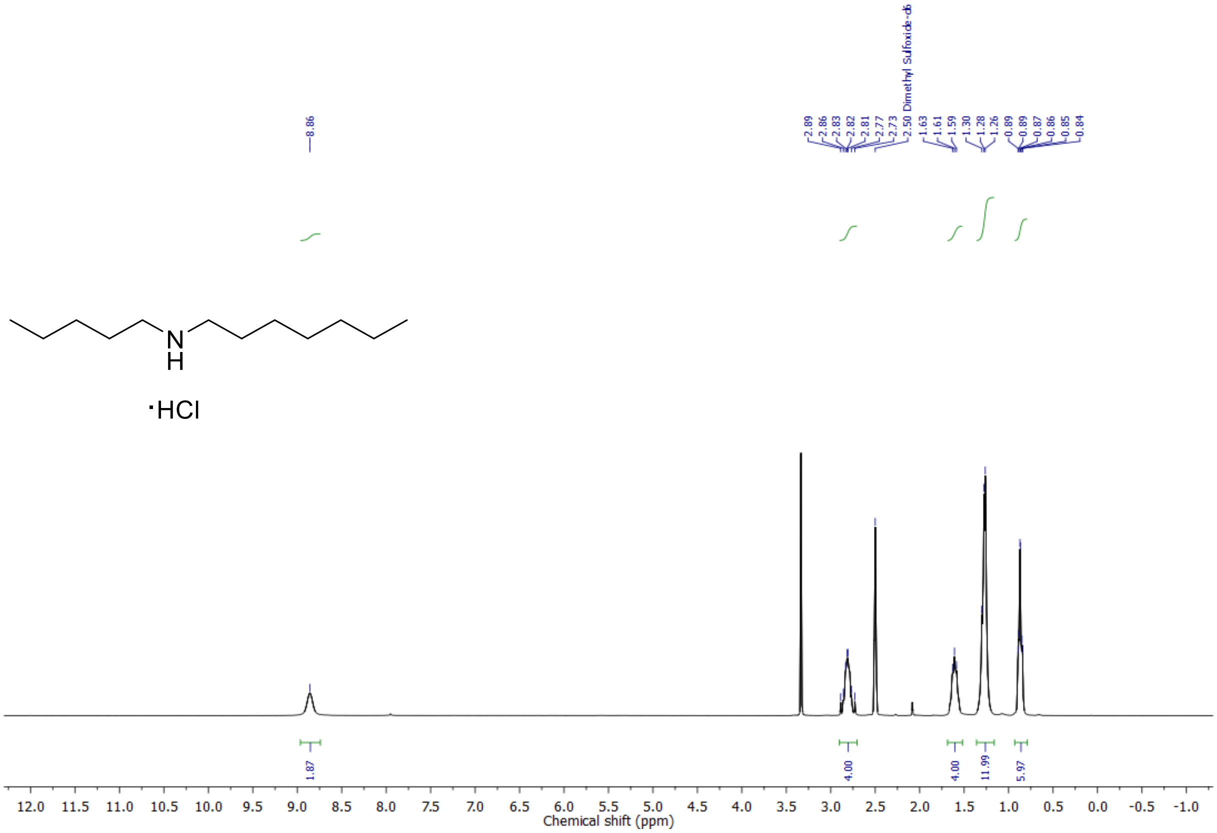

Figure S112: ^{1}H-NMR spectrum of **52**.

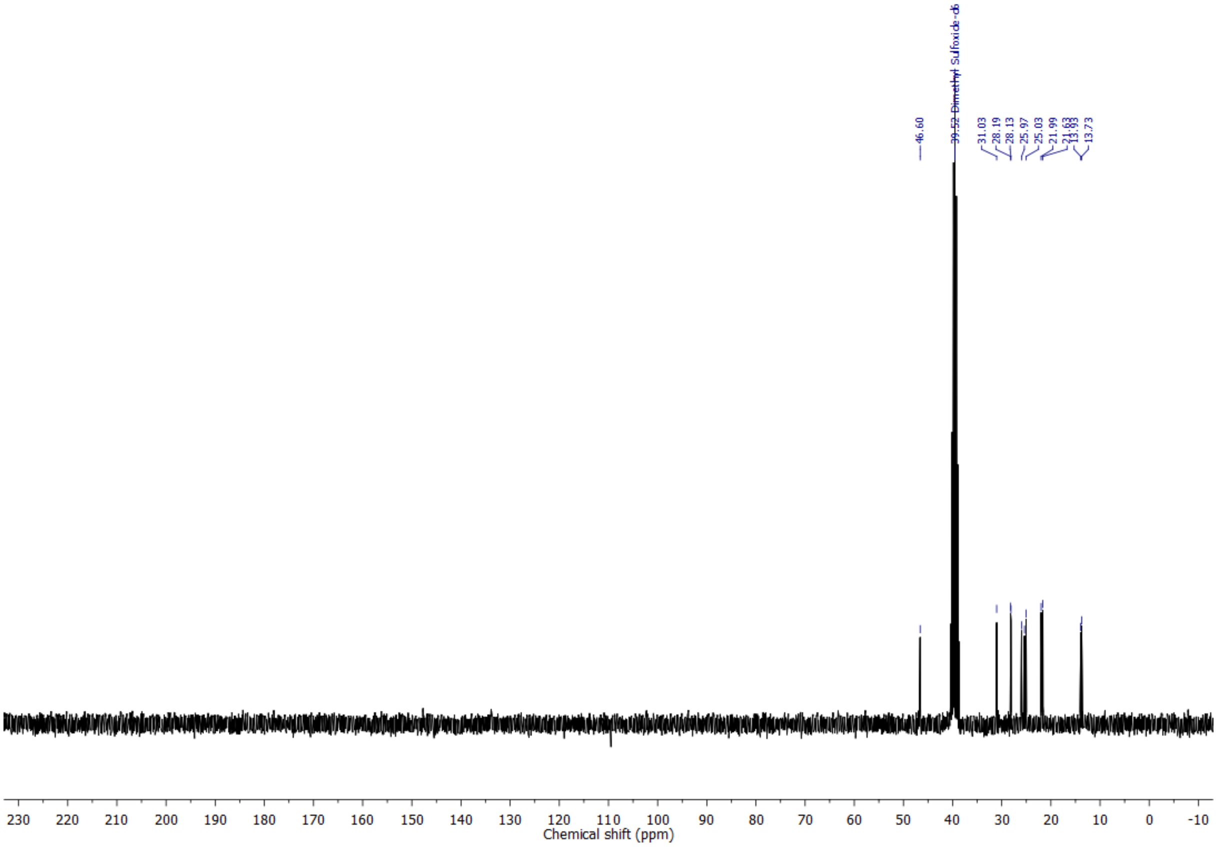

Figure S113: ^{13}C-NMR spectrum of **52**.

N-pentyldecan-1-aminium chloride (53)

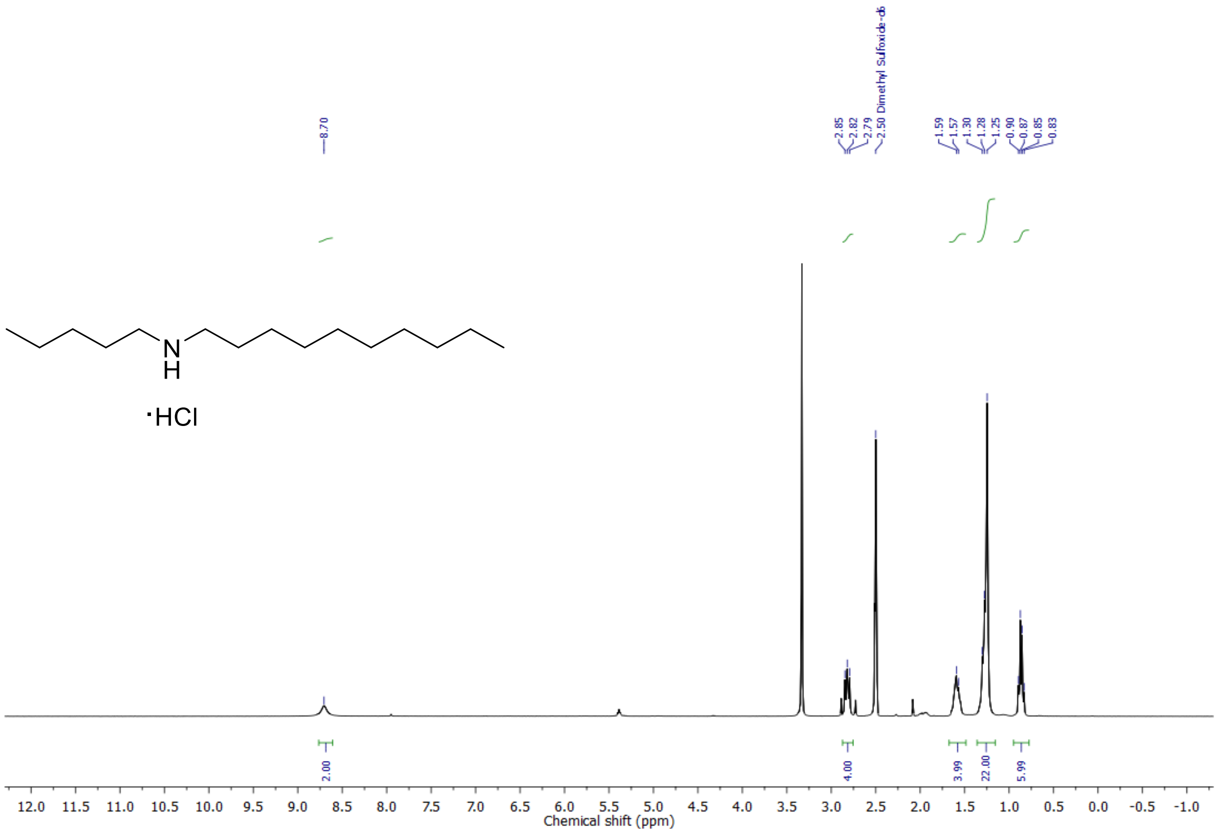

Figure S114: ^{1}H-NMR spectrum of **53**.

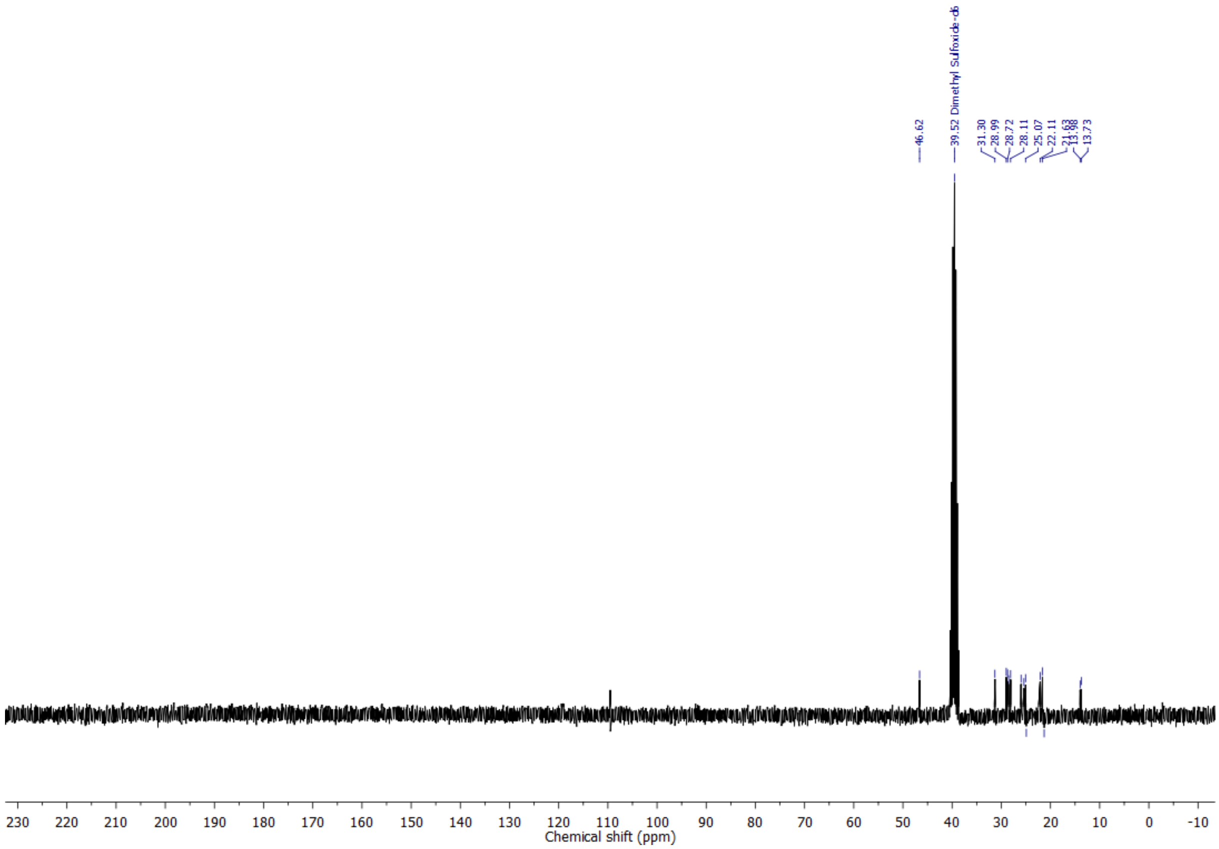

Figure S115: ^{13}C-NMR spectrum of **53**.

***N*-pentyldodecan-1-aminium chloride (54)**

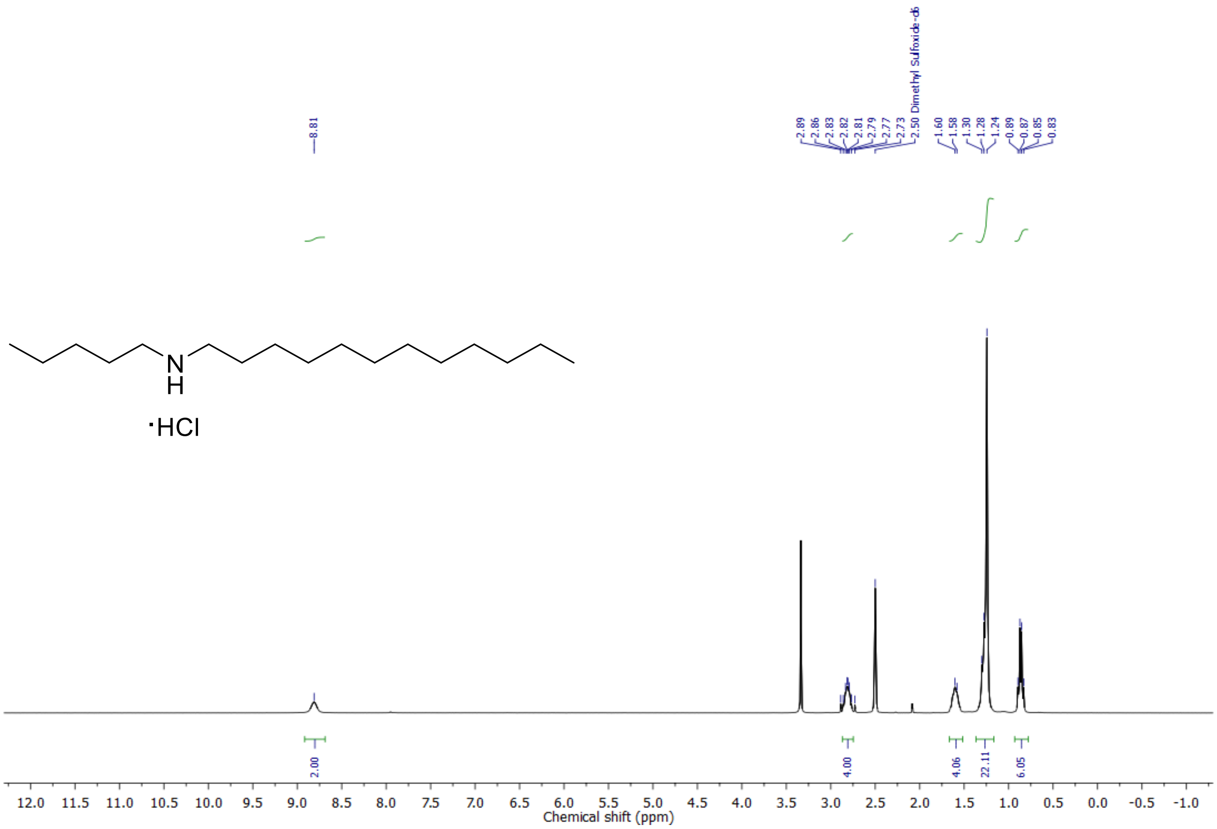

Figure S116: ^{1}H-NMR spectrum of **54**.

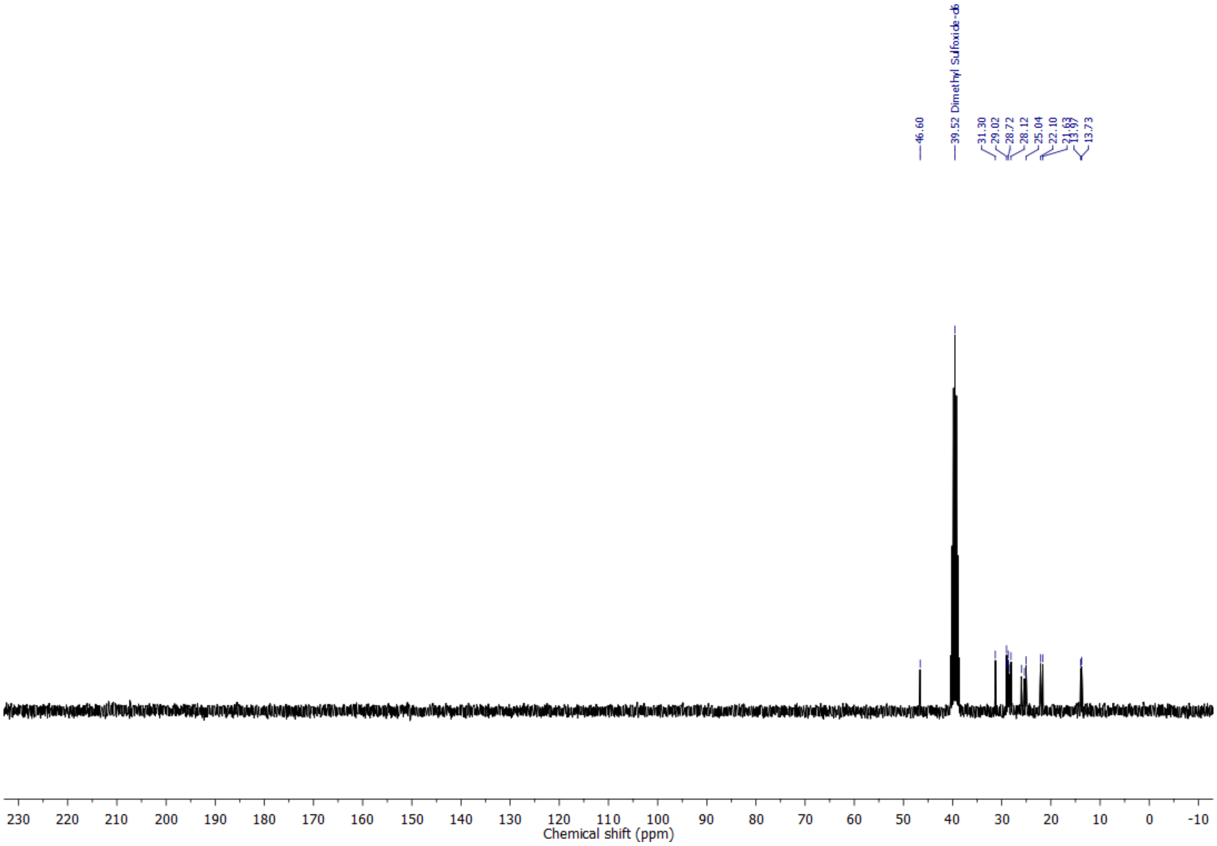

Figure S117: ^{13}C-NMR spectrum of **54**.

***N*-pentyldodecan-1-ammoniumchlorid (55)**

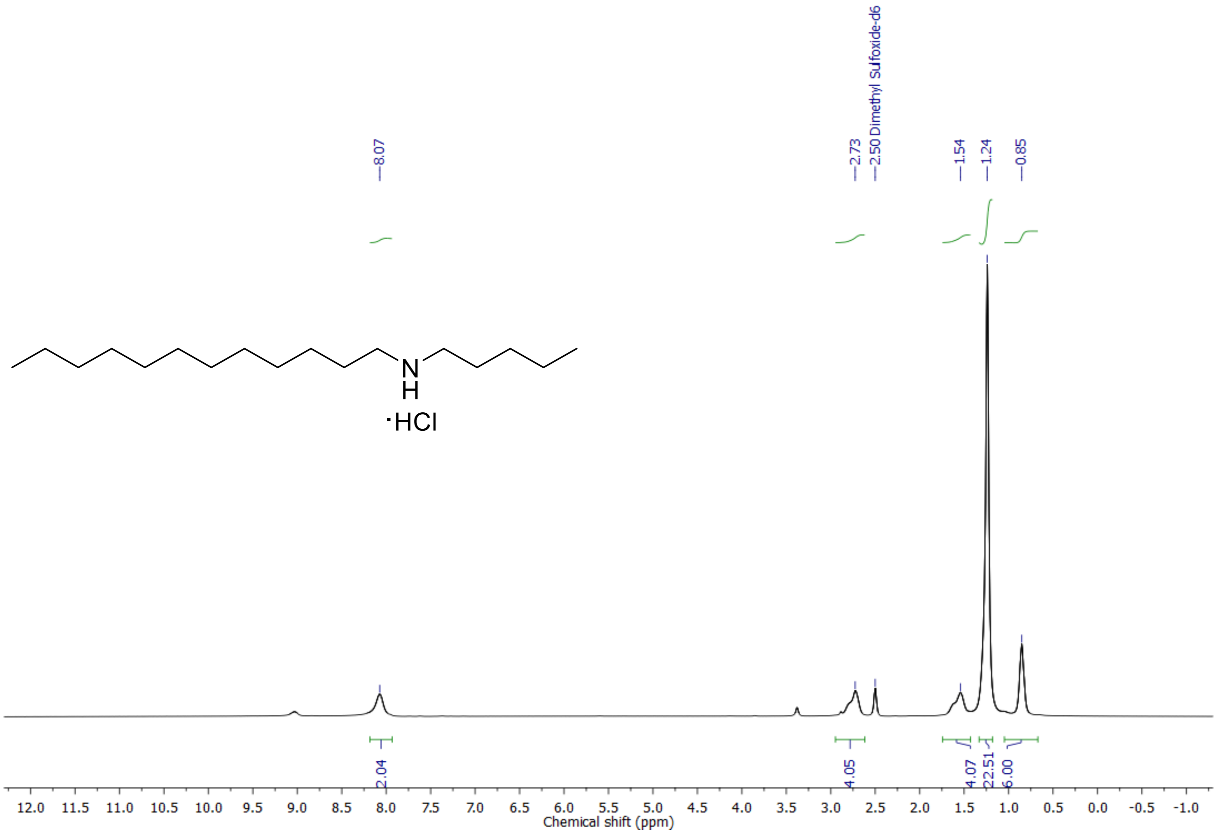

Figure S118: ^{1}H-NMR spectrum of **55**.

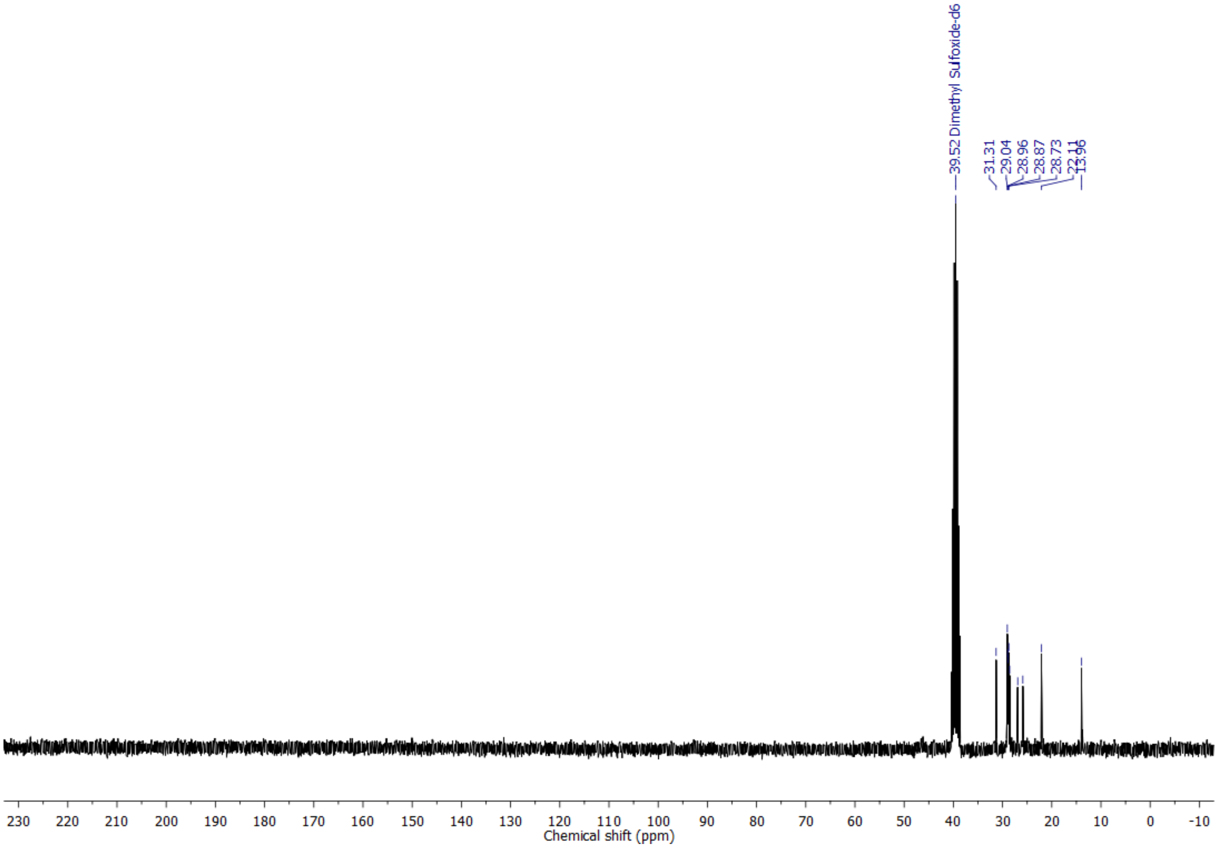

Figure S119: ^{13}C-NMR spectrum of **55**.

N-benzyl-1,1-diphenylmethanaminium chloride (56)

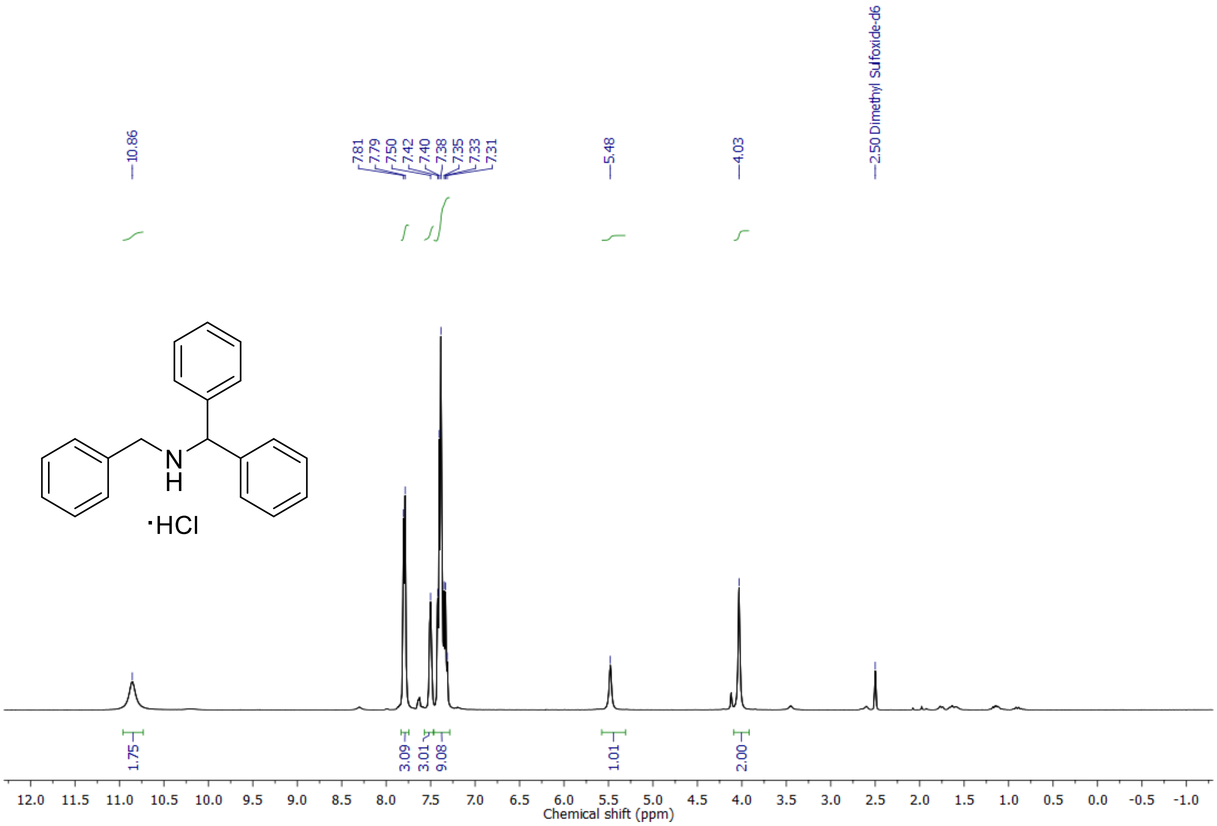

Figure S120: ^{1}H-NMR spectrum of **56**.

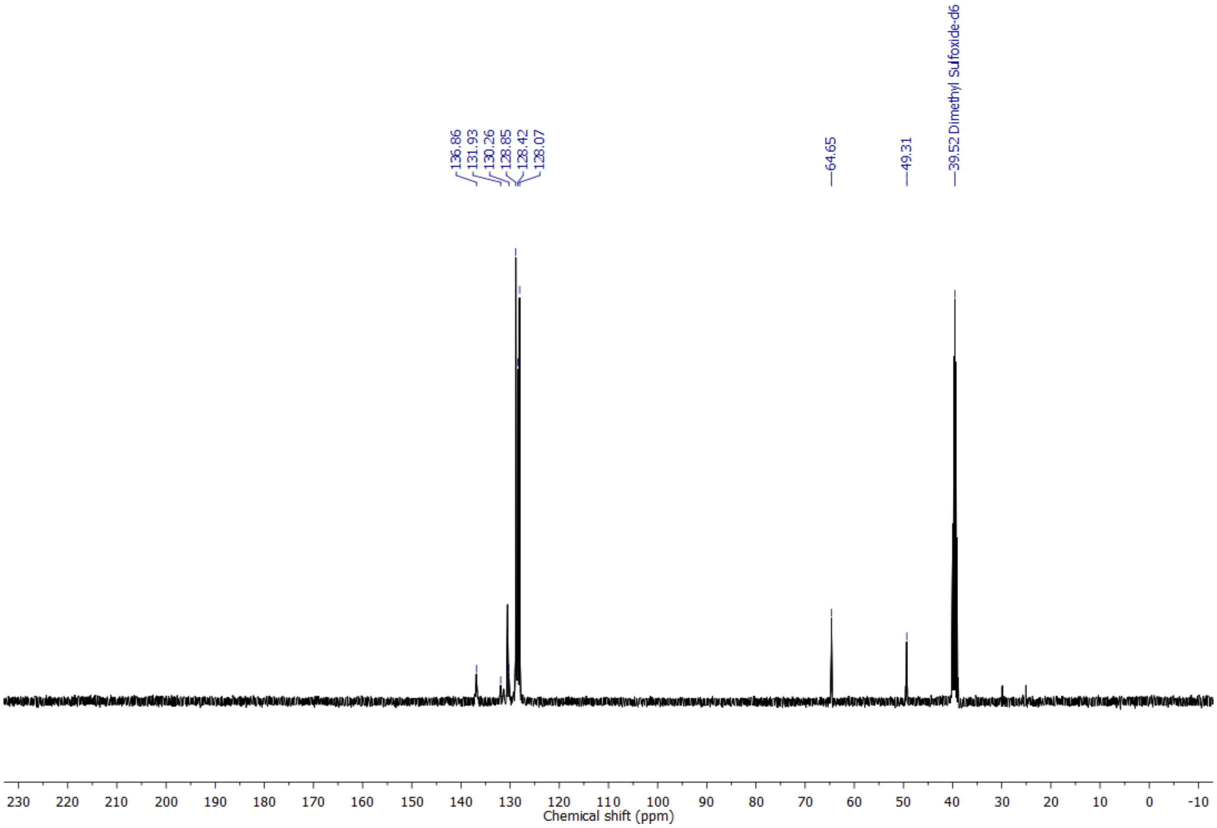

Figure S121: ^{13}C-NMR spectrum of **56**.

***N*-benzyl-1-phenylethan-1-aminium chloride (57)**

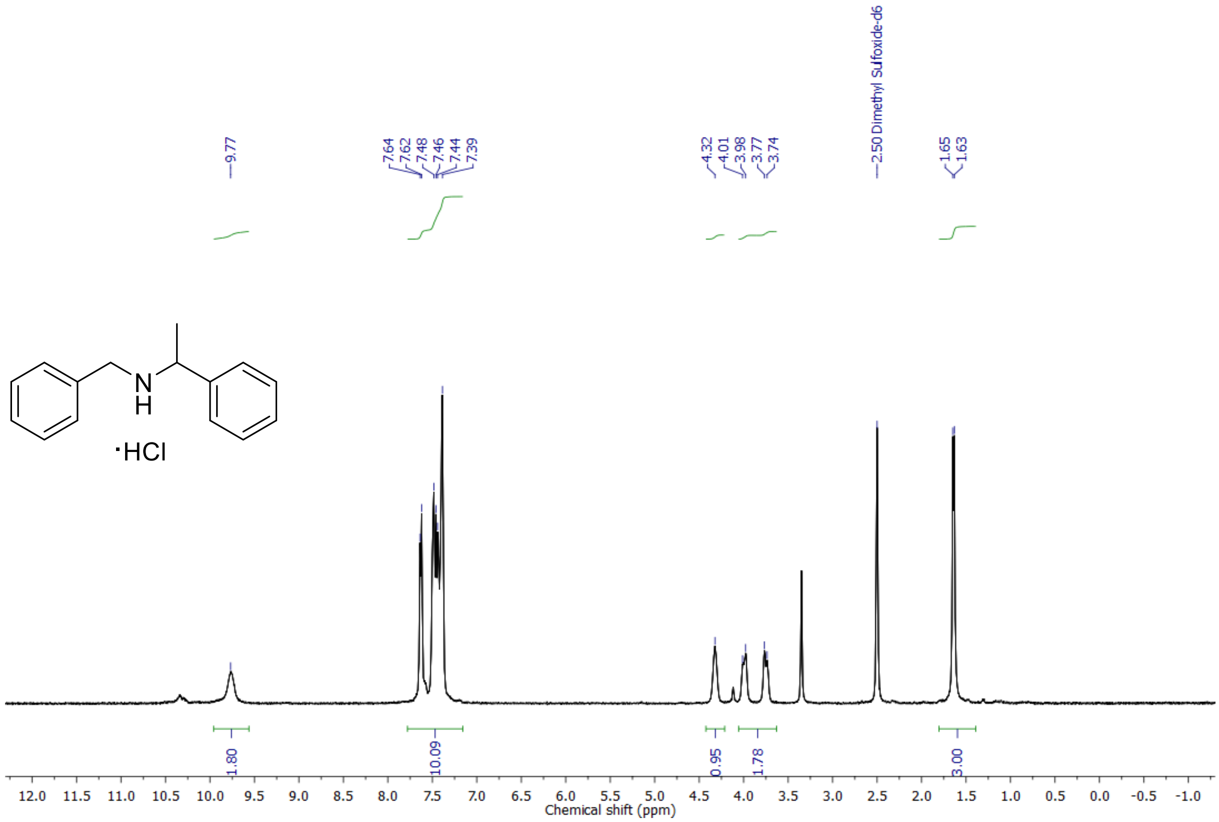

Figure S122: ^{1}H-NMR spectrum of **57**.

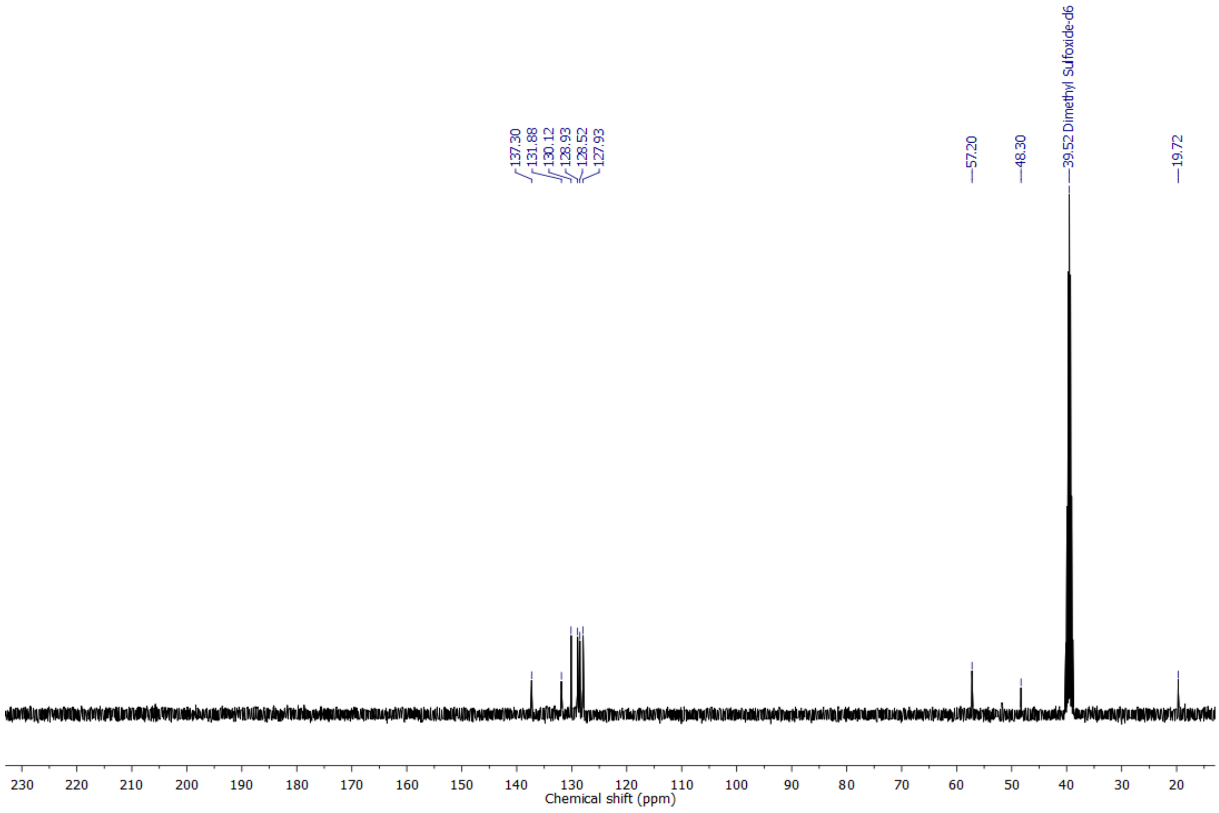

Figure S123: ^{13}C-NMR spectrum of **57**.

N-benzyloctan-2-aminium chloride (58)

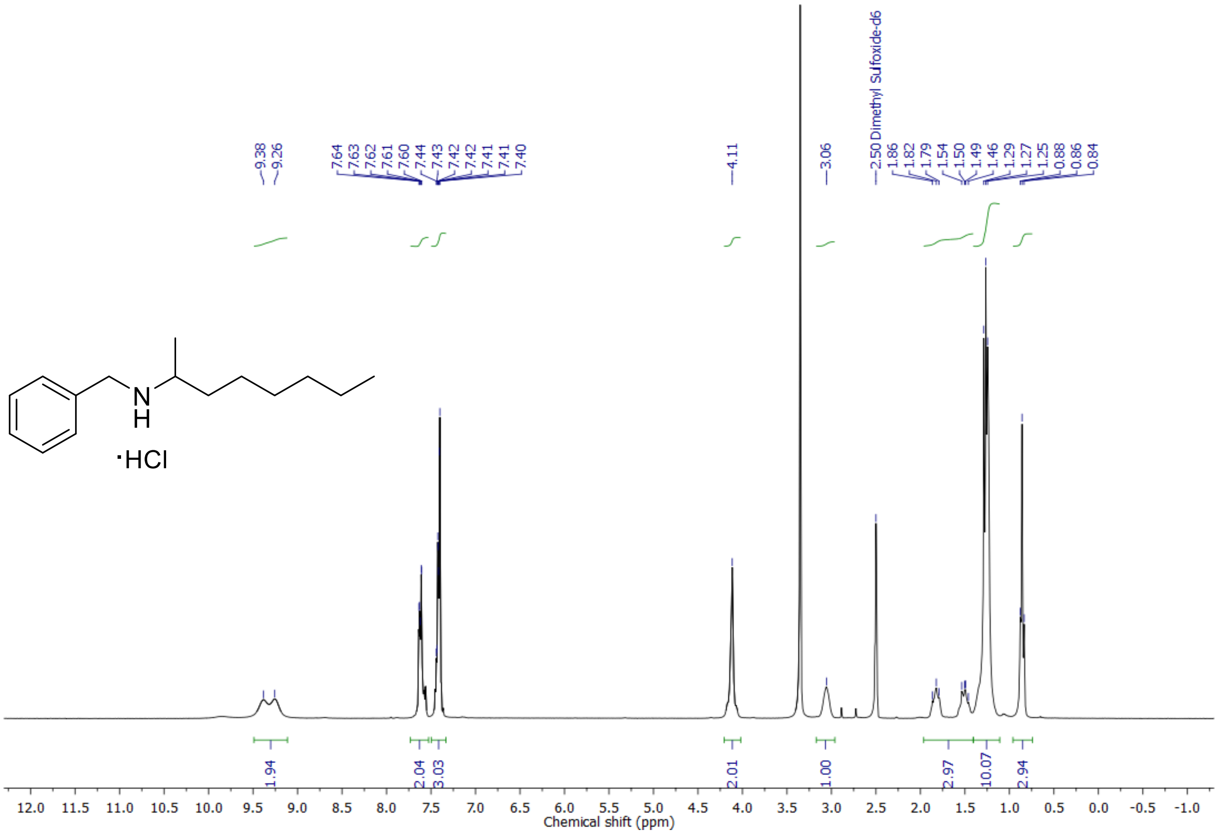

Figure S124: ^{1}H-NMR spectrum of **58**.

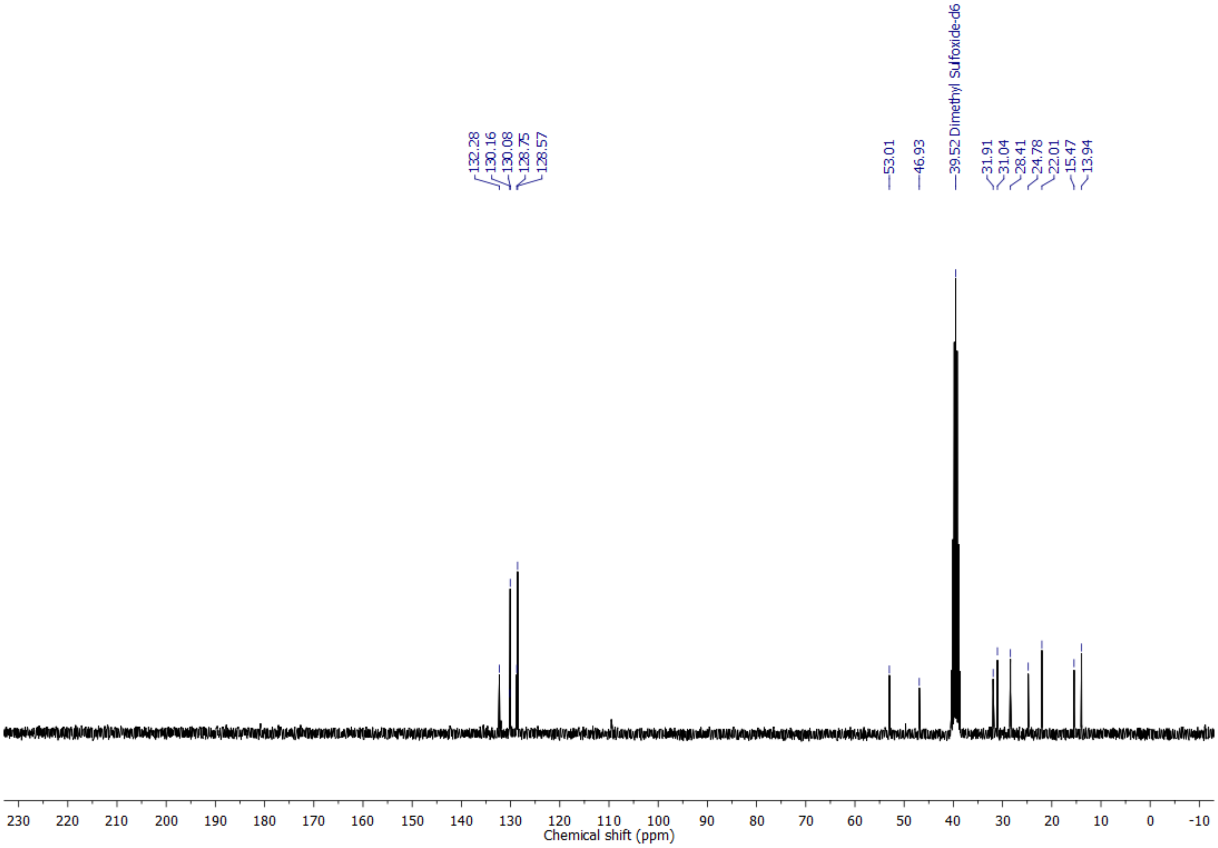

Figure S125: ^{13}C-NMR spectrum of **58**.

***N*-benzylcyclopentanaminium chloride (59)**

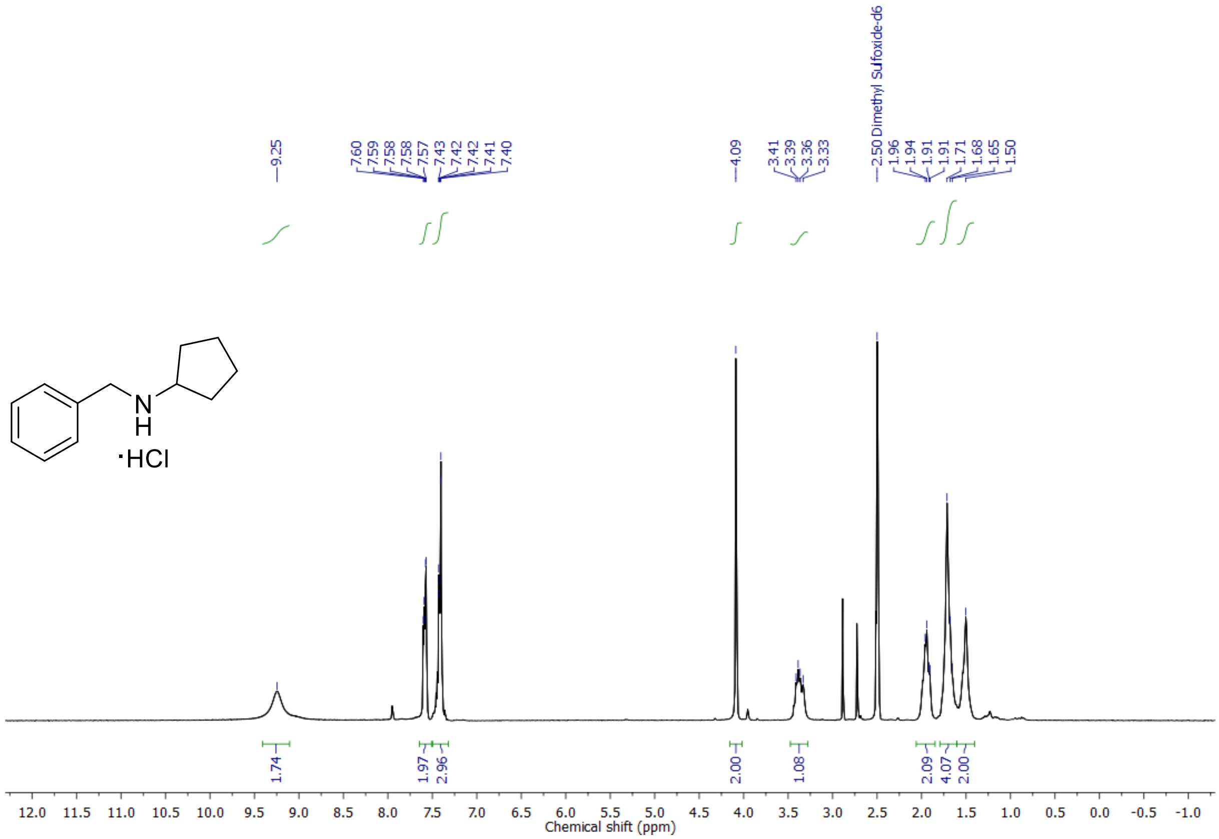

Figure S126: ^{1}H-NMR spectrum of **59**.

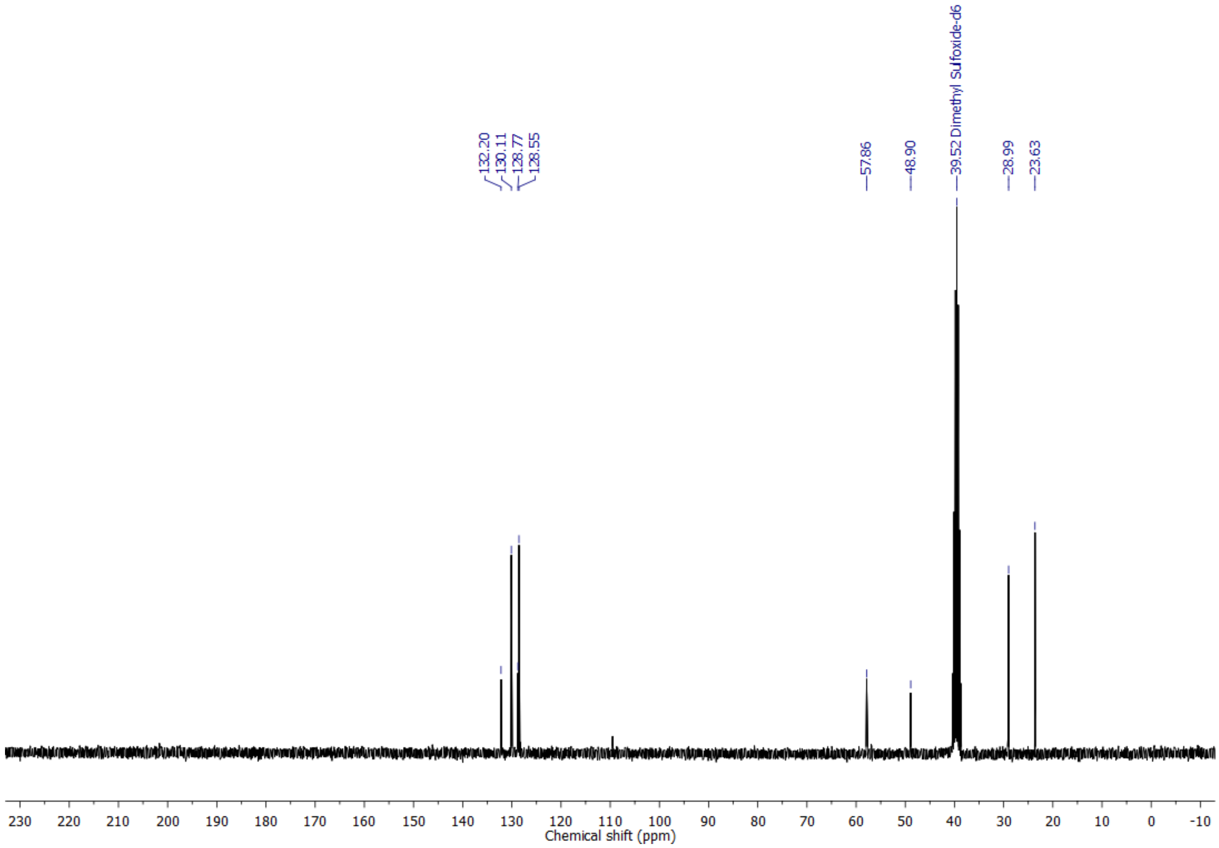

Figure S127: ^{13}C-NMR spectrum of **59**.

***N*-benzhydrylpentan-1-aminium chloride (60)**

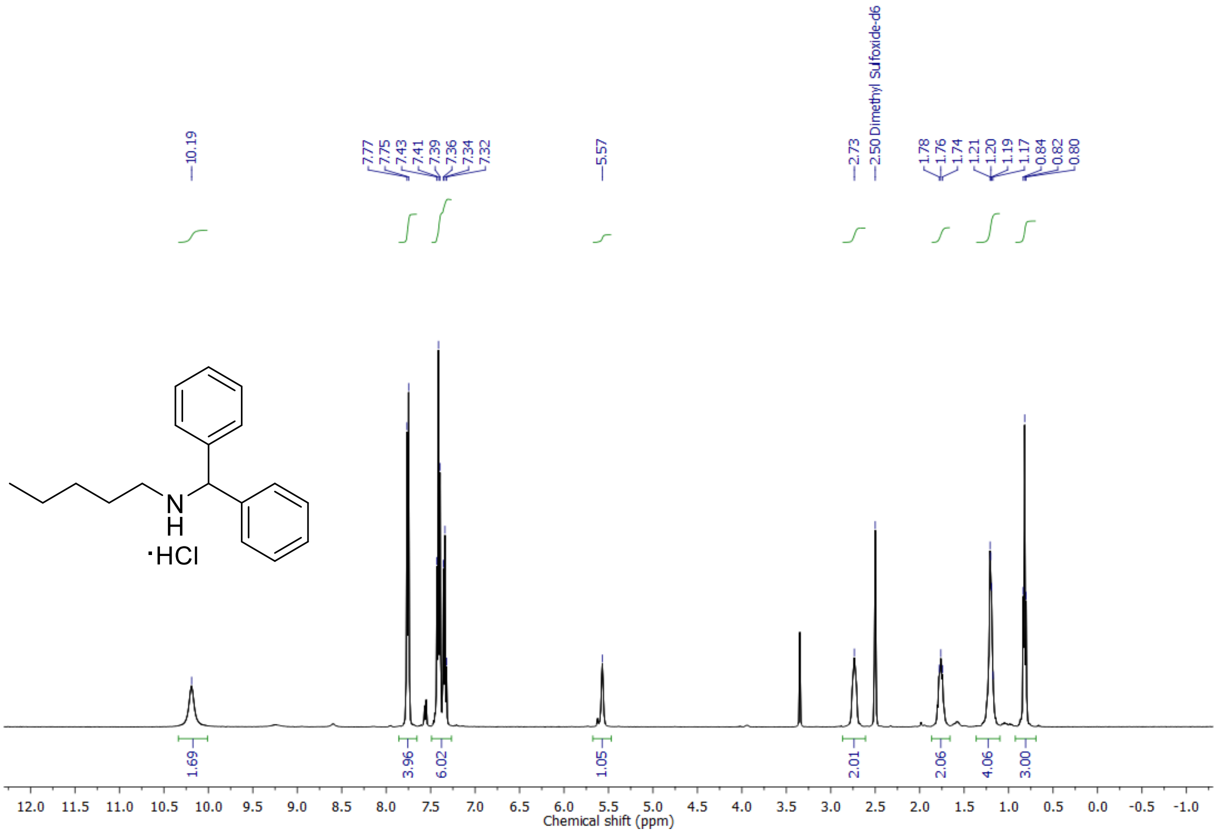

Figure S128: ^{1}H-NMR spectrum of **60**.

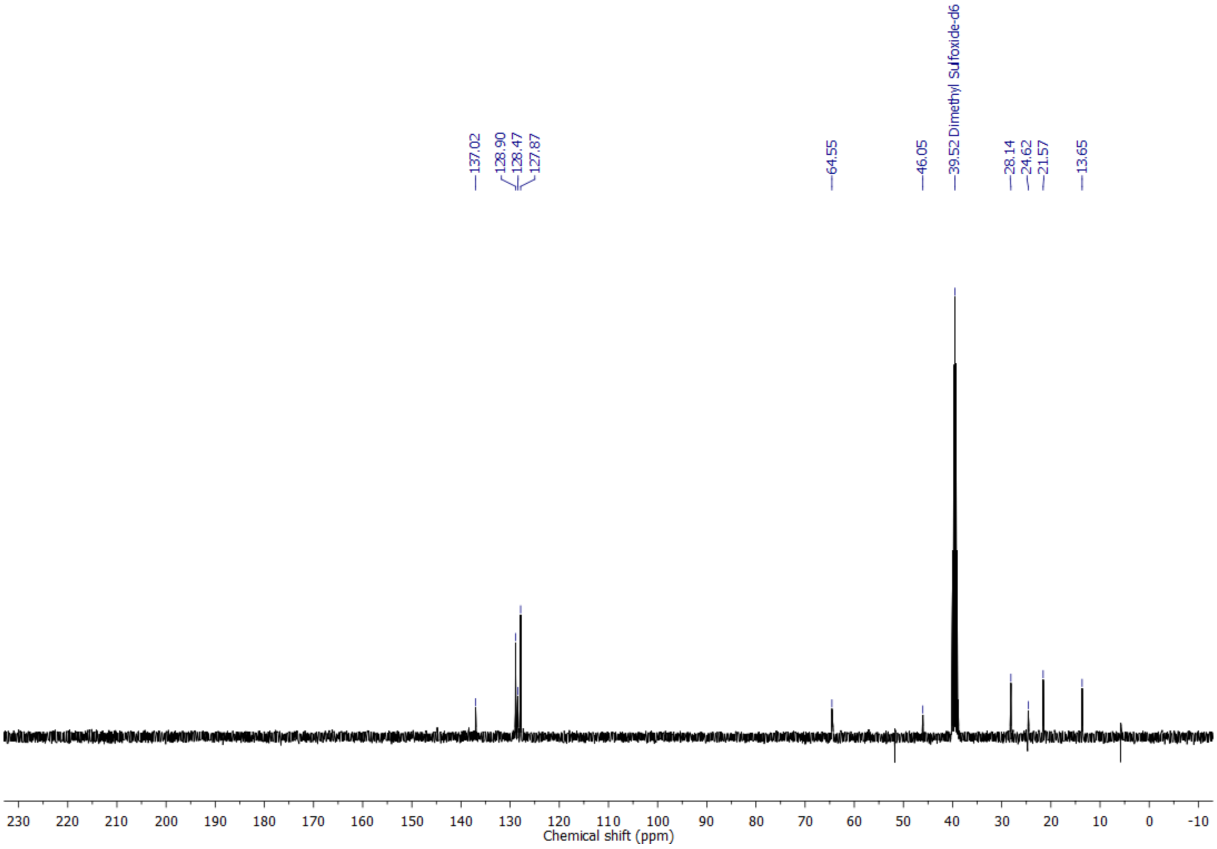

Figure S129: ^{13}C-NMR spectrum of **60**.

***N*-(1-phenylethyl)pentan-1-aminium chloride (61)**

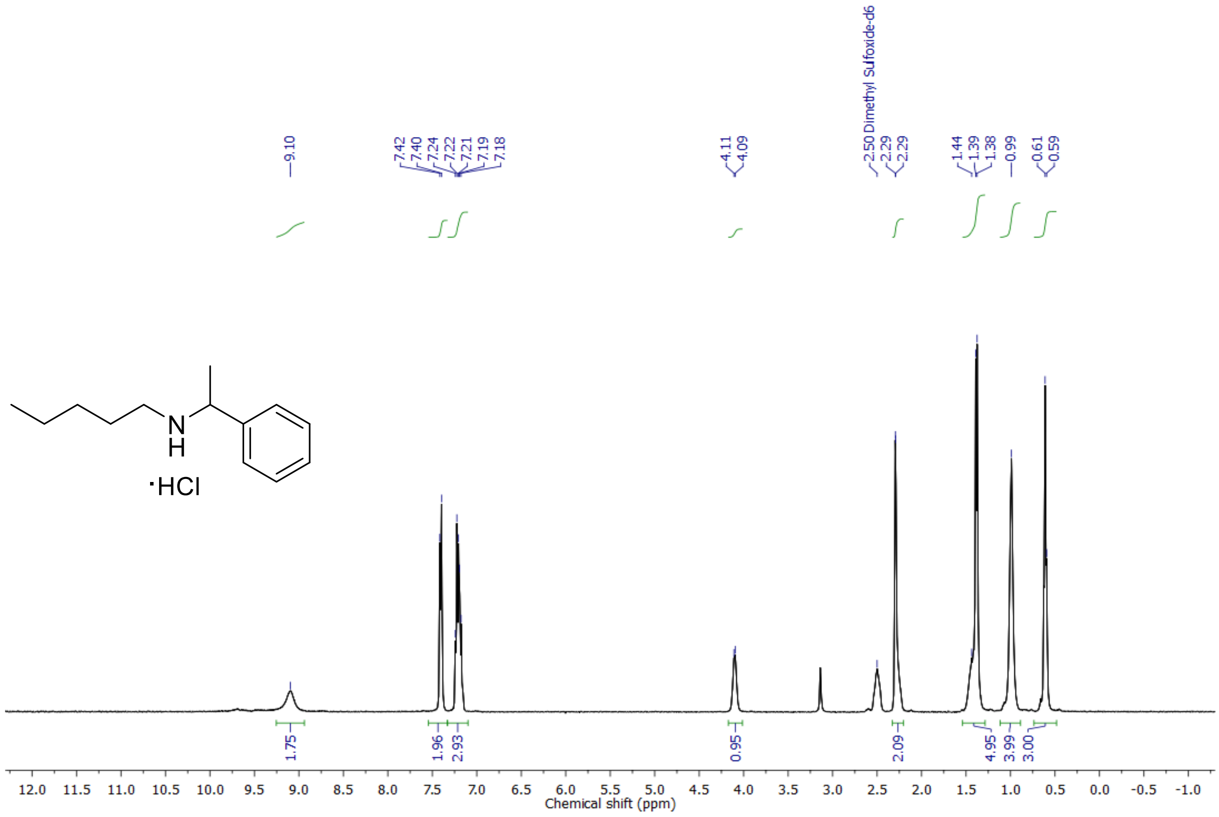

Figure S130: ^{1}H-NMR spectrum of **61**.

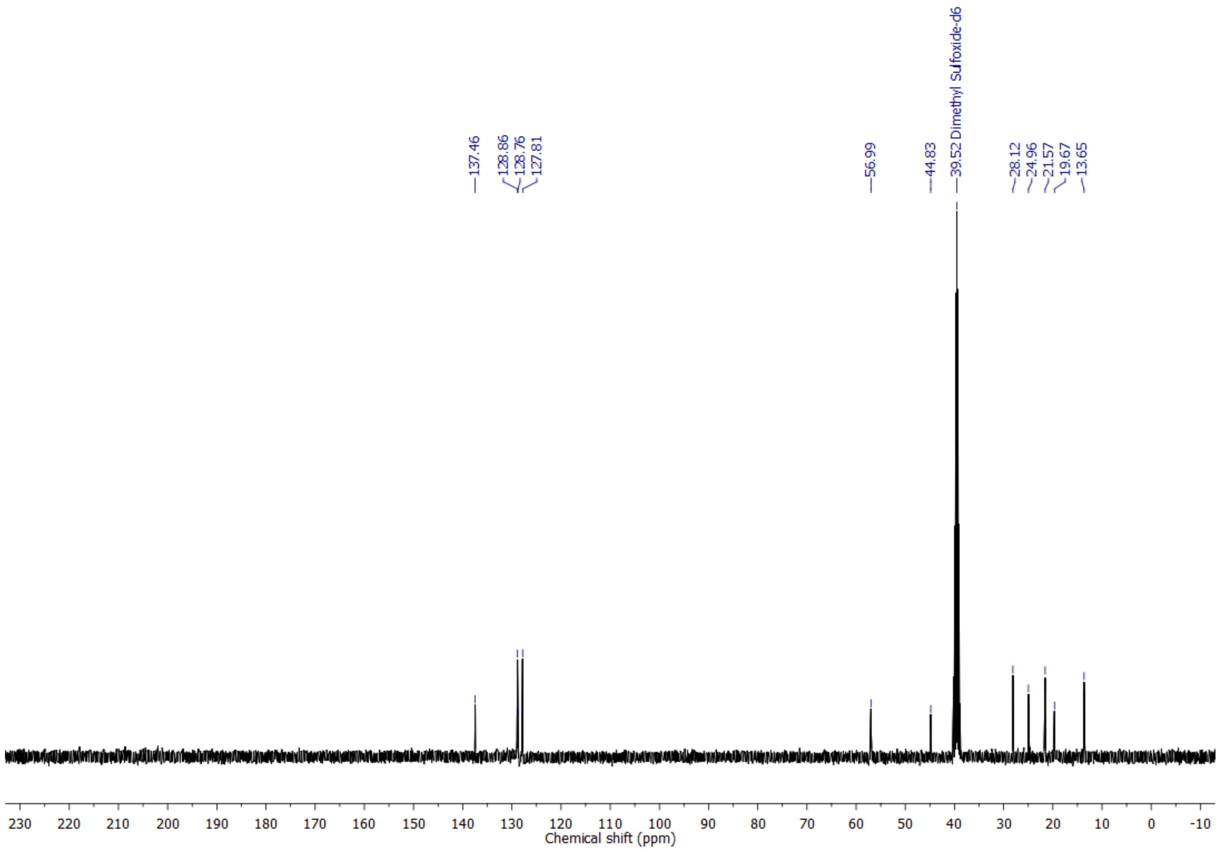

Figure S131: ^{13}C-NMR spectrum of **61**.

***N*-pentyloctan-2-aminium chloride (62)**

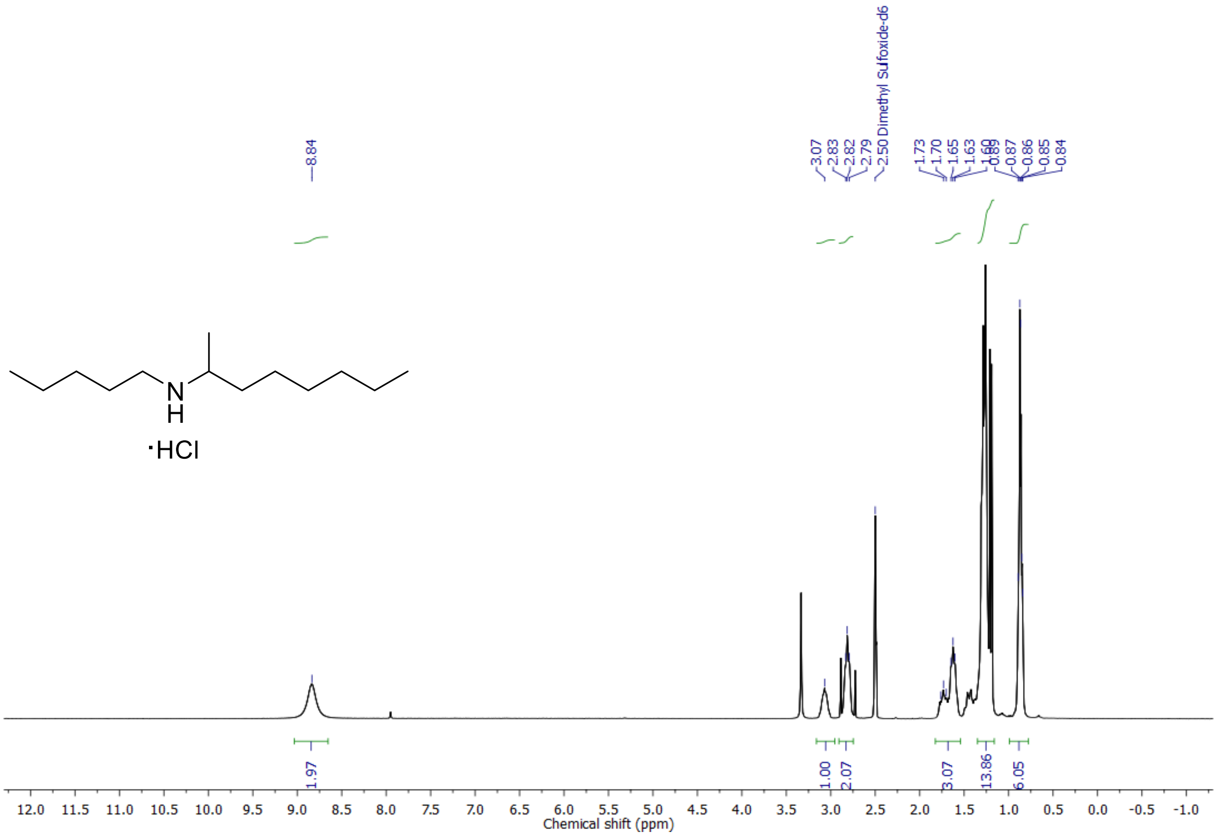

Figure S132: ^{1}H-NMR spectrum of **62**.

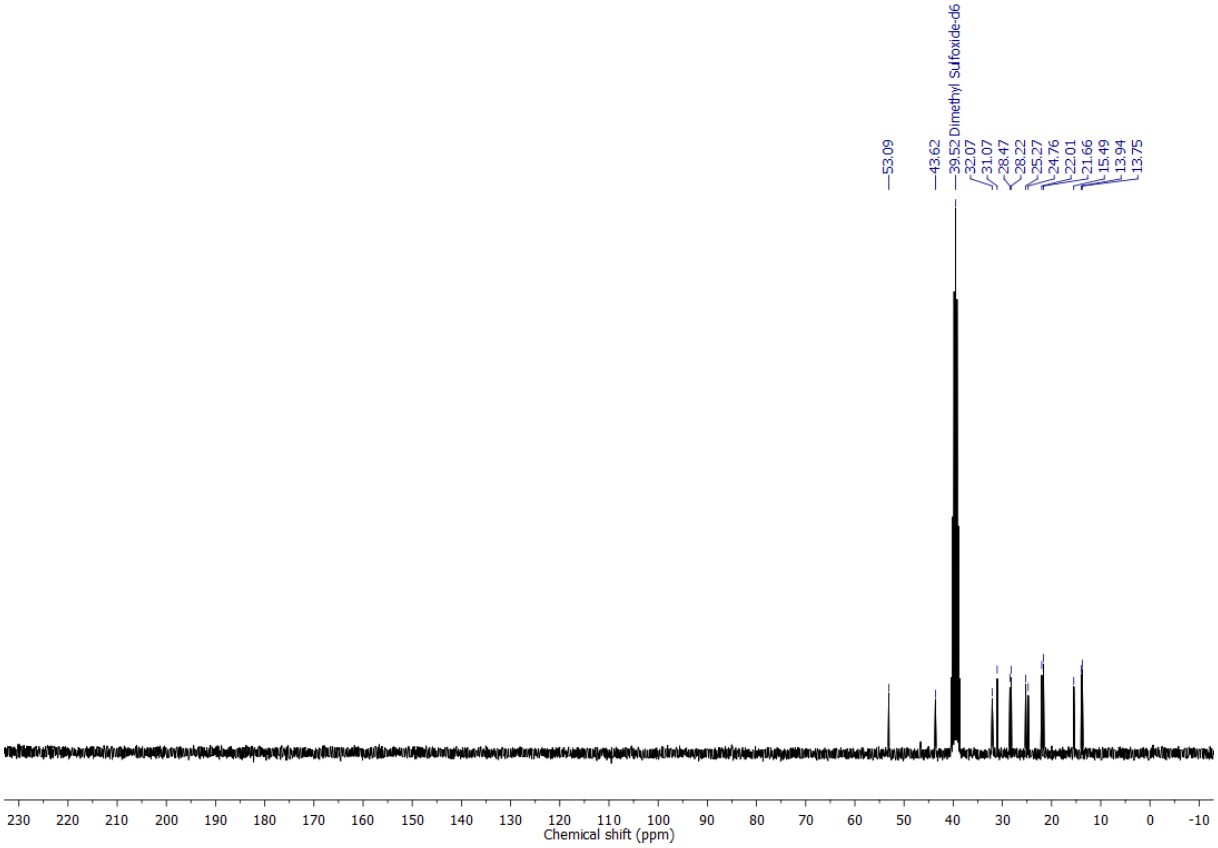

Figure S133: ^{13}C-NMR spectrum of **62**.

***N*-pentylcyclopentanammoniumchlorid (63)**

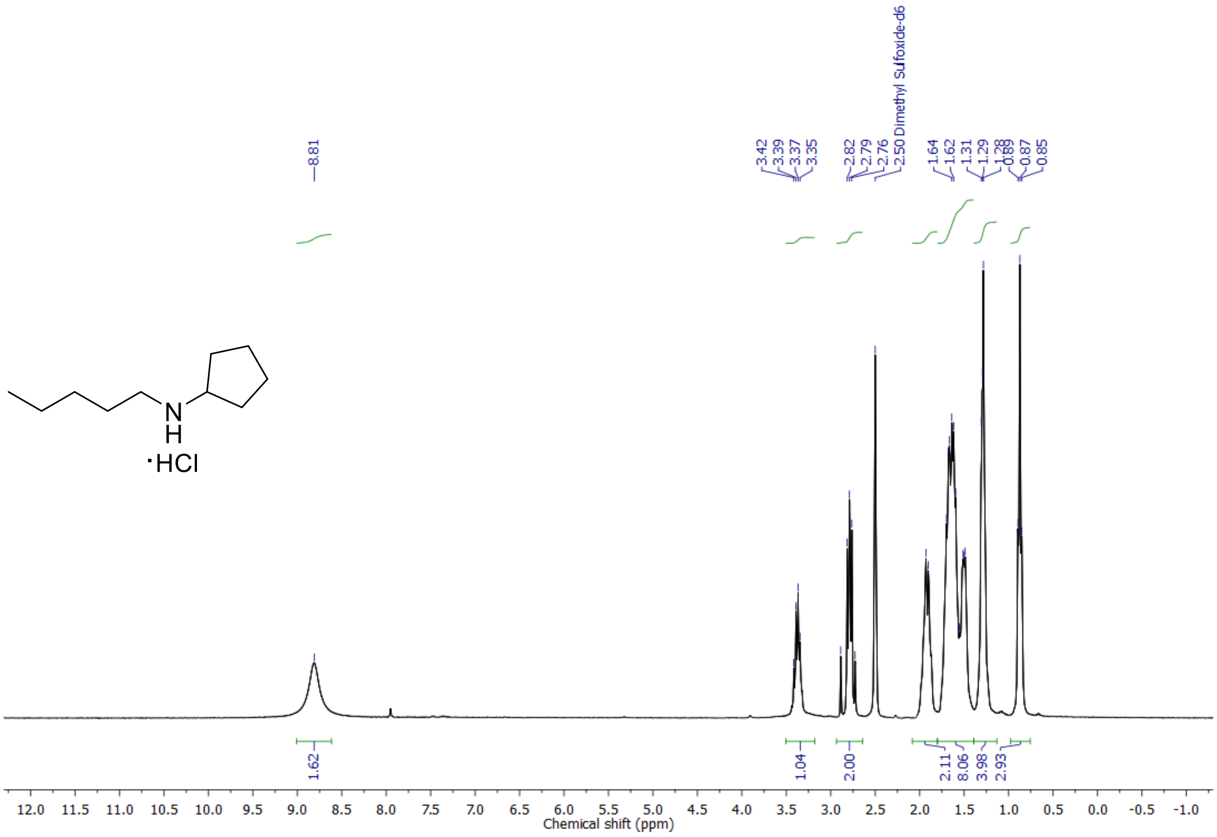

Figure S134: ^{1}H-NMR spectrum of **63**.

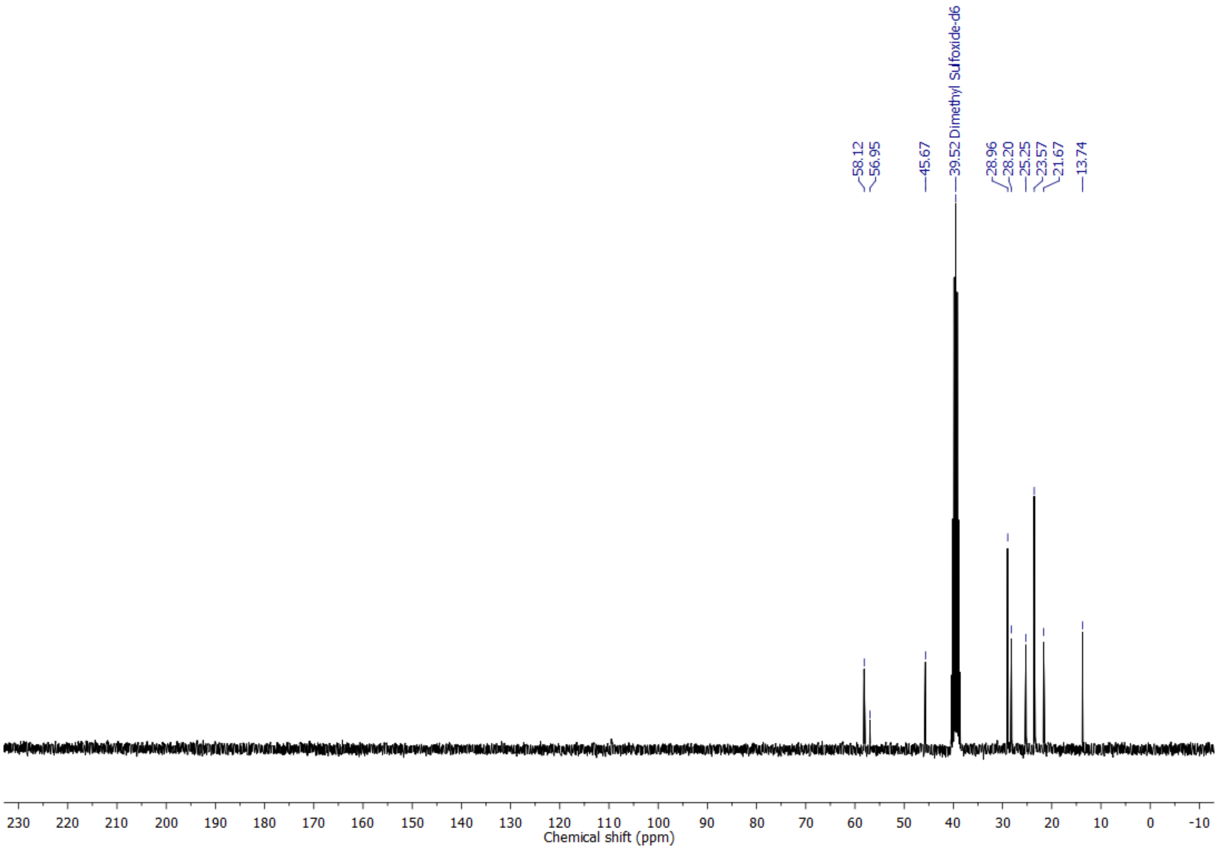

Figure S135: ^{13}C-NMR spectrum of **63**.

N-benzyl-4-(6-methoxynaphthalen-2-yl)butan-2-aminium chloride (64)

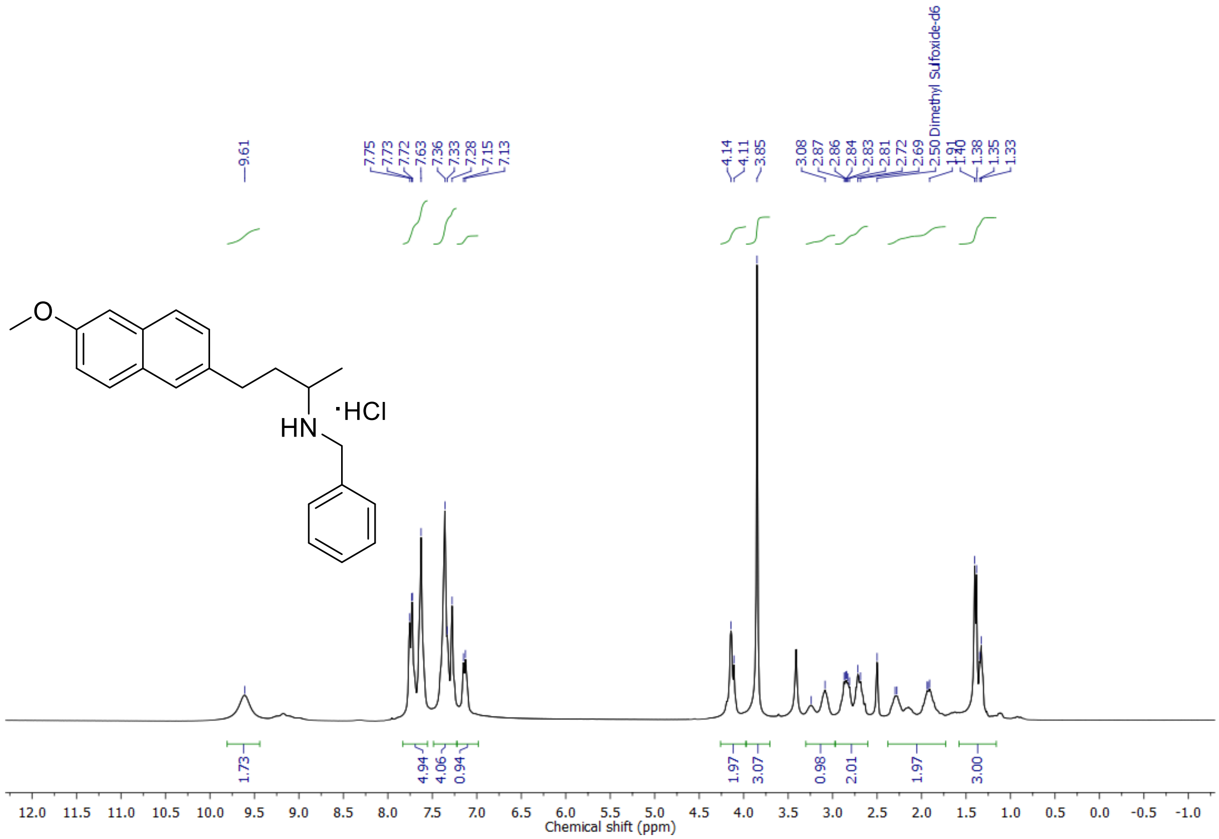

Figure S136: ^{1}H-NMR spectrum of **64**.

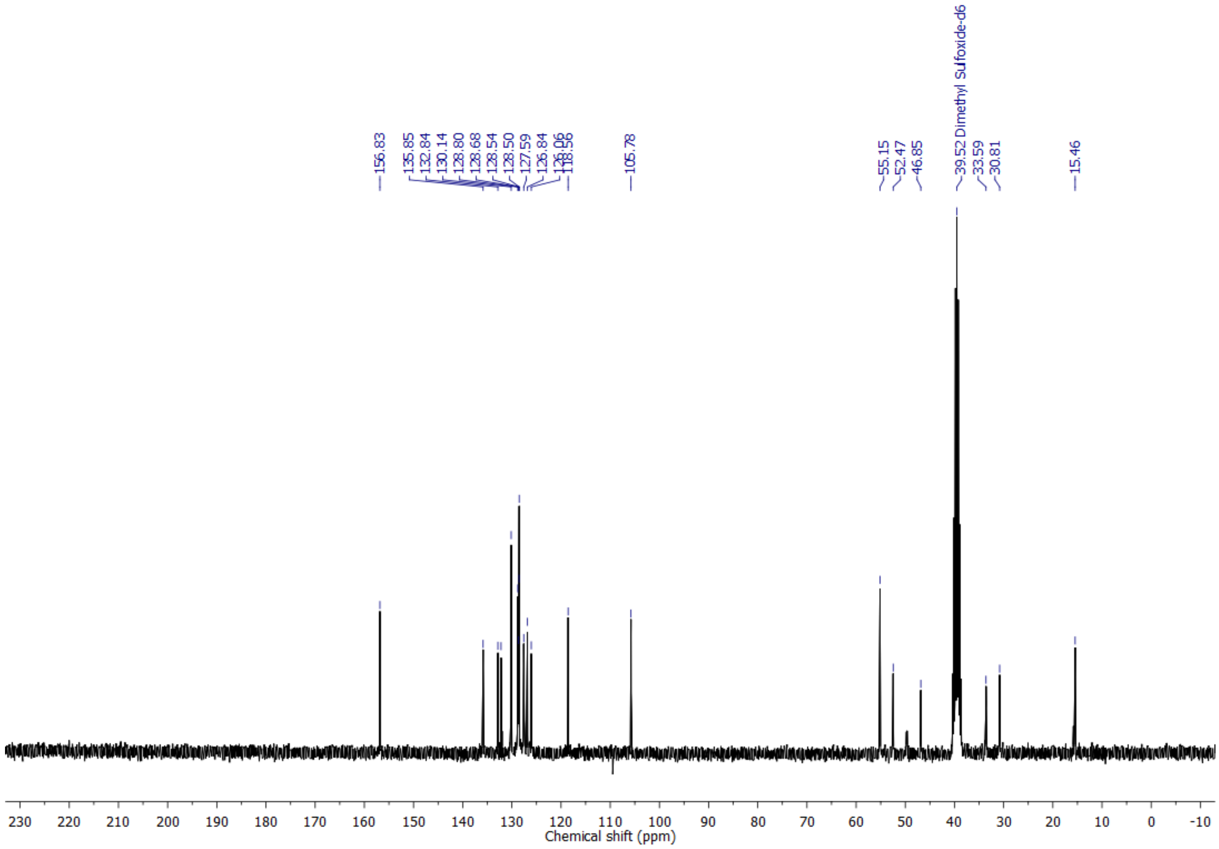

Figure S137: ^{13}C-NMR spectrum of **64**.

***N*-(4-(6-methoxynaphthalen-2-yl)butan-2-yl)pentan-1-aminium chloride (65)**

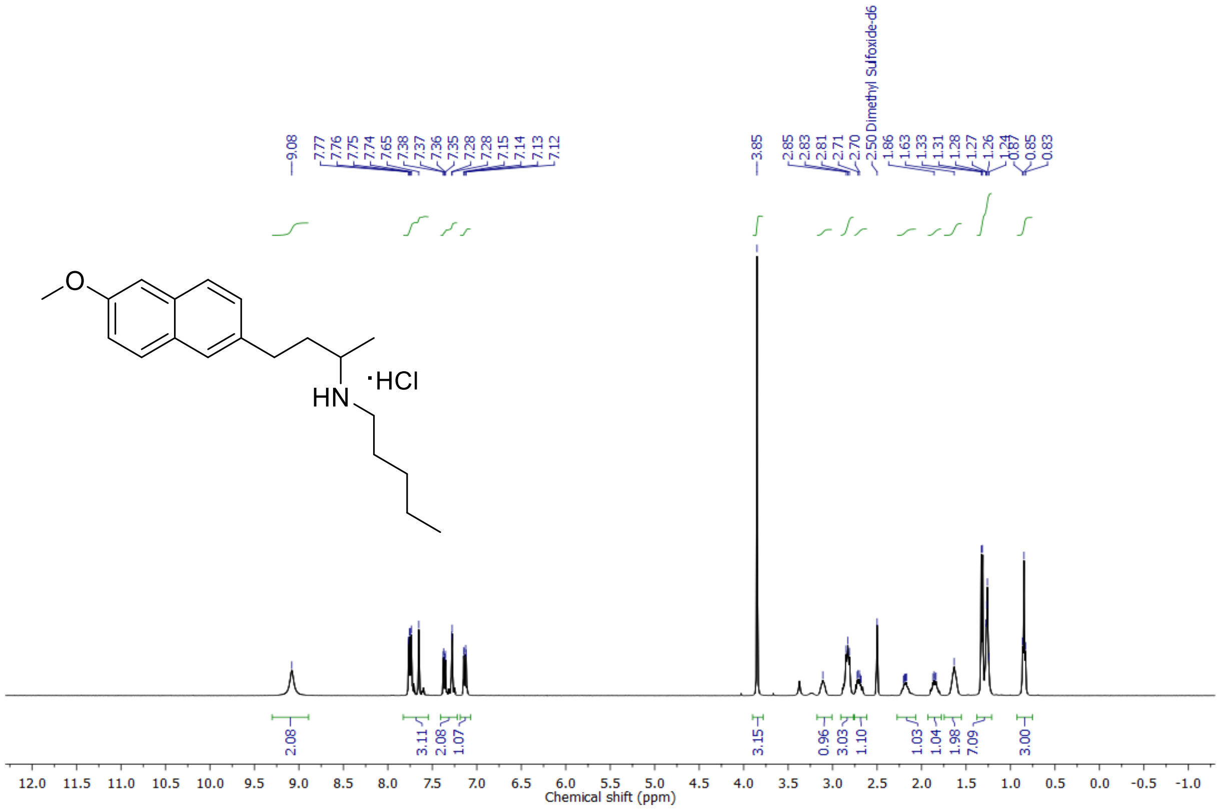

Figure S138: ^{1}H-NMR spectrum of **65**.

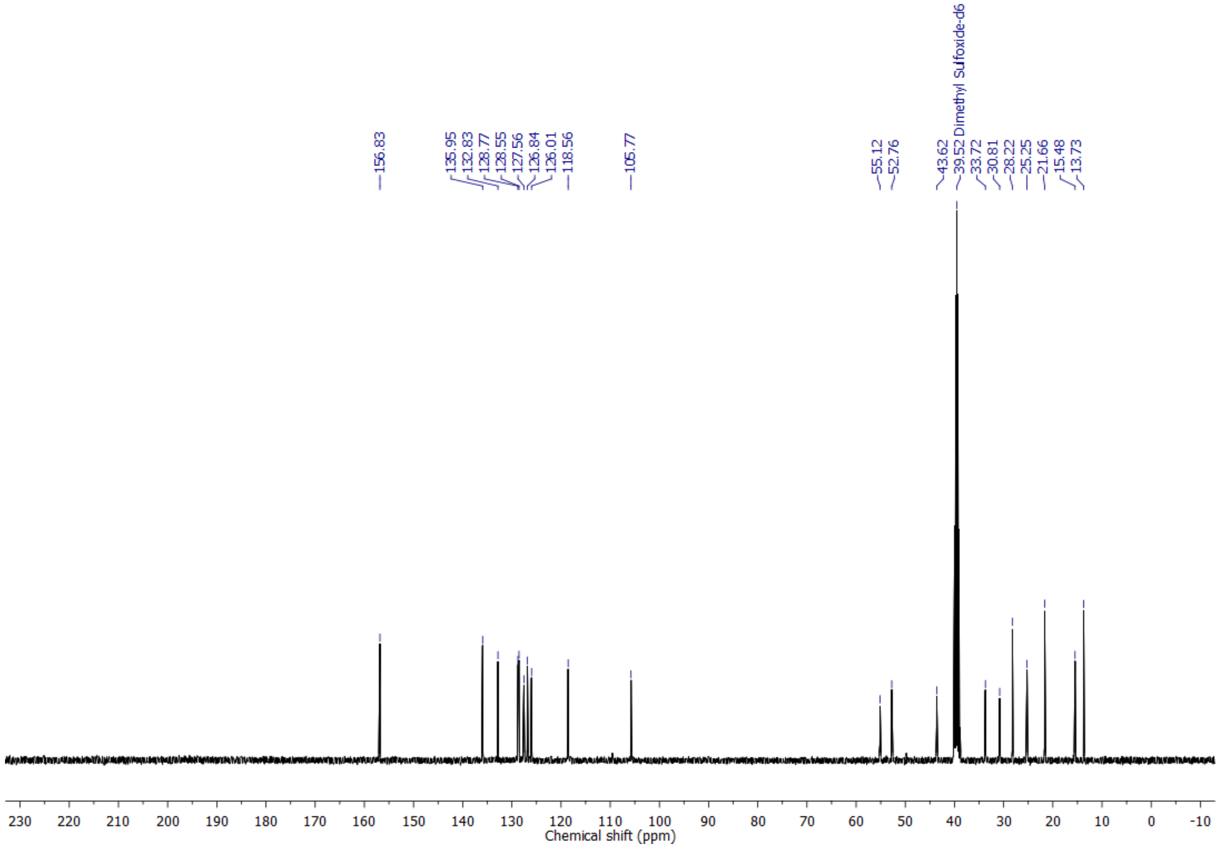

Figure S139: ^{13}C-NMR spectrum of **65**.

1-(5-(benzylamino)hexyl)-3,7-dimethyl-3,7-dihydro-1*H*-purine-2,6-dione hydrochloride (66)

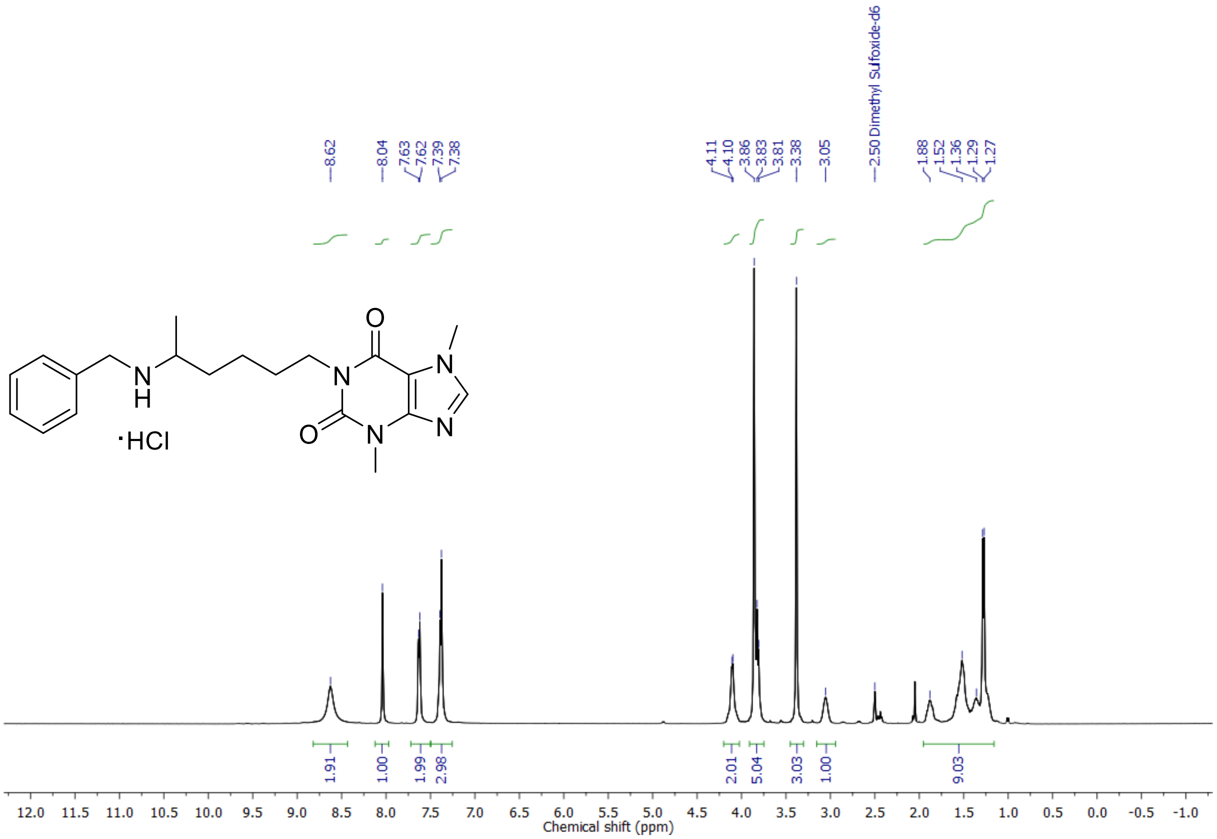

Figure S140: ^{1}H-NMR spectrum of **66**.

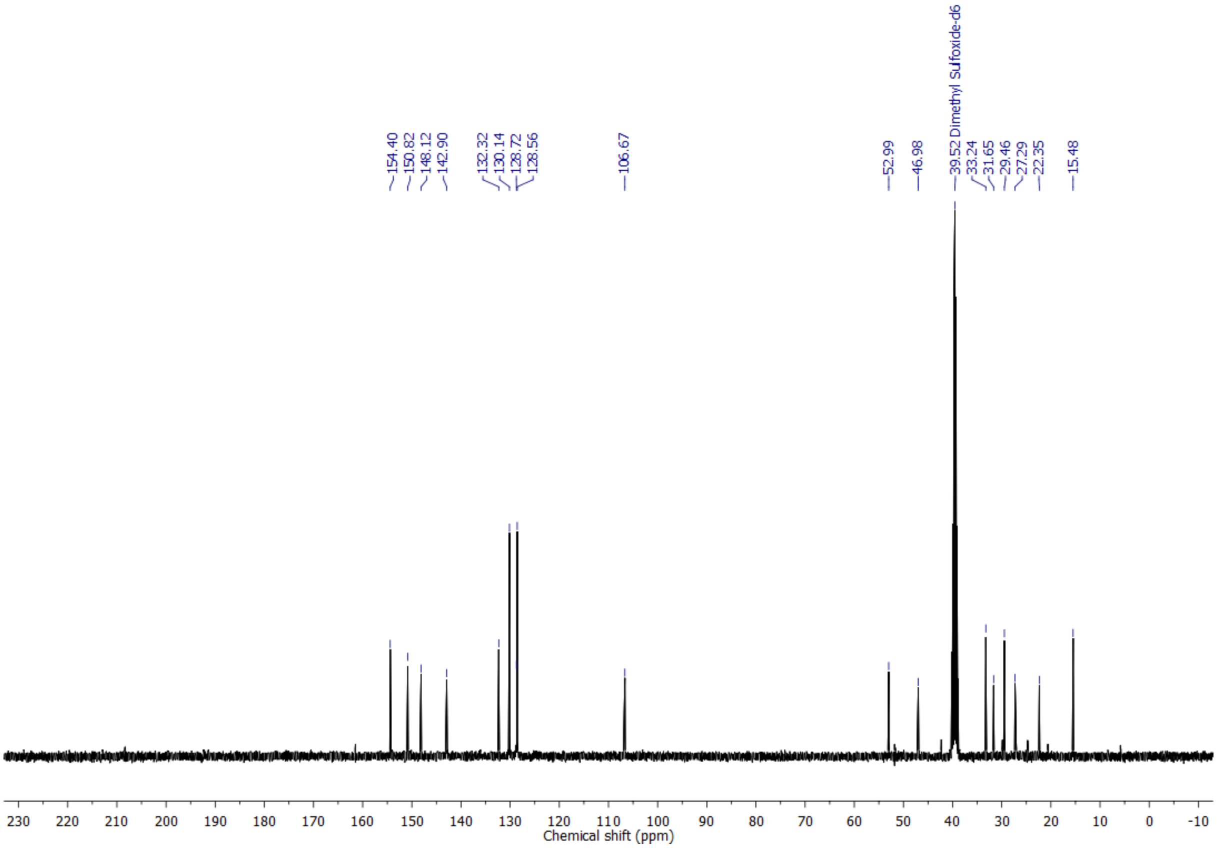

Figure S141: ^{13}C-NMR spectrum of **66**.

3,7-dimethyl-1-(5-(pentylamino)hexyl)-3,7-dihydro-1*H*-purine-2,6-dione hydrochloride (67)

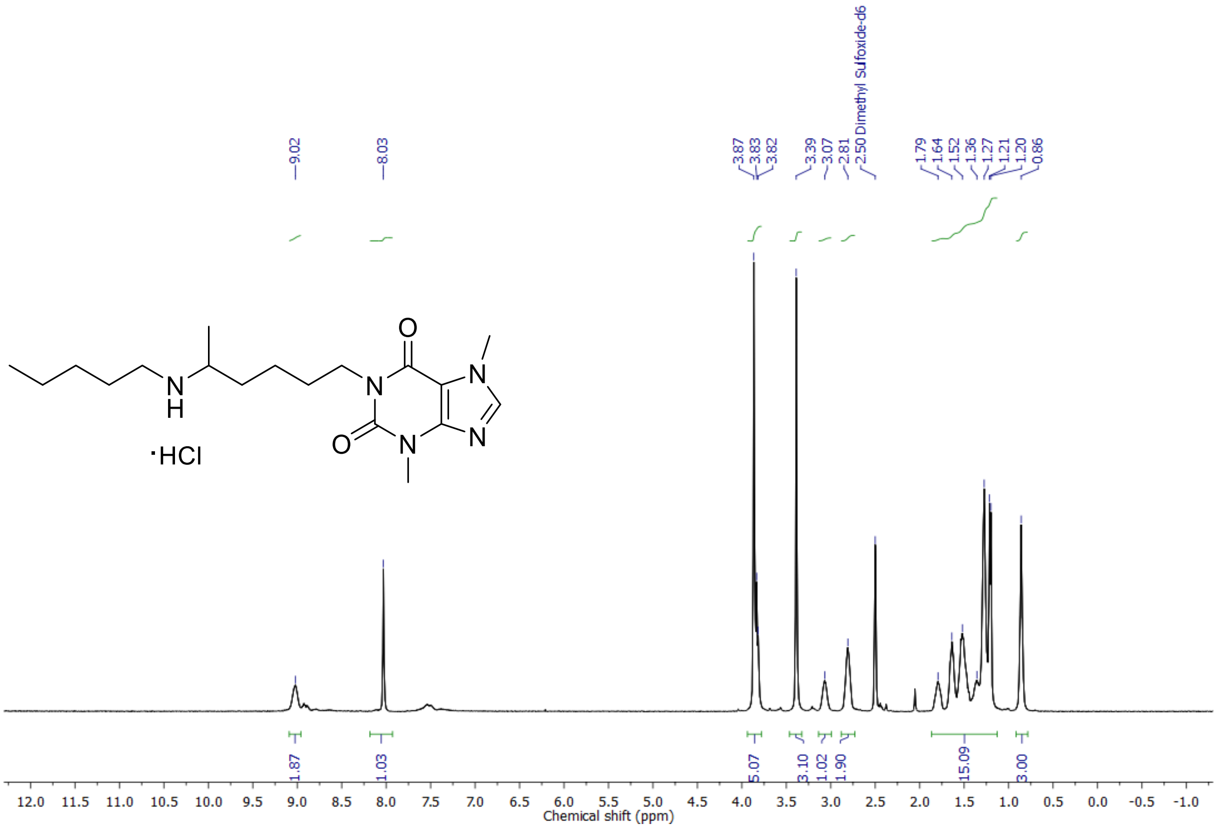

Figure S142: ^{1}H-NMR spectrum of **67**.

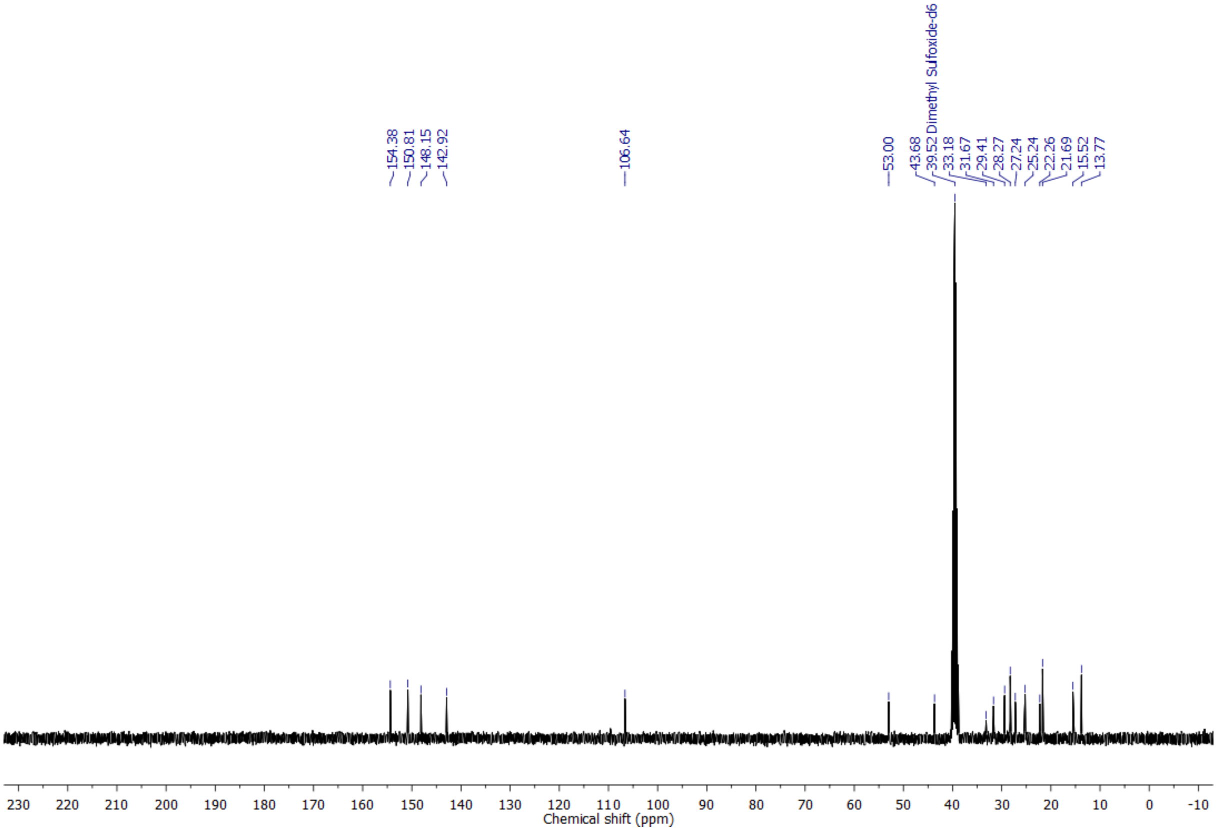

Figure S143: ^{13}C-NMR spectrum of **67**.

Tecalcet hydrochloride (68)

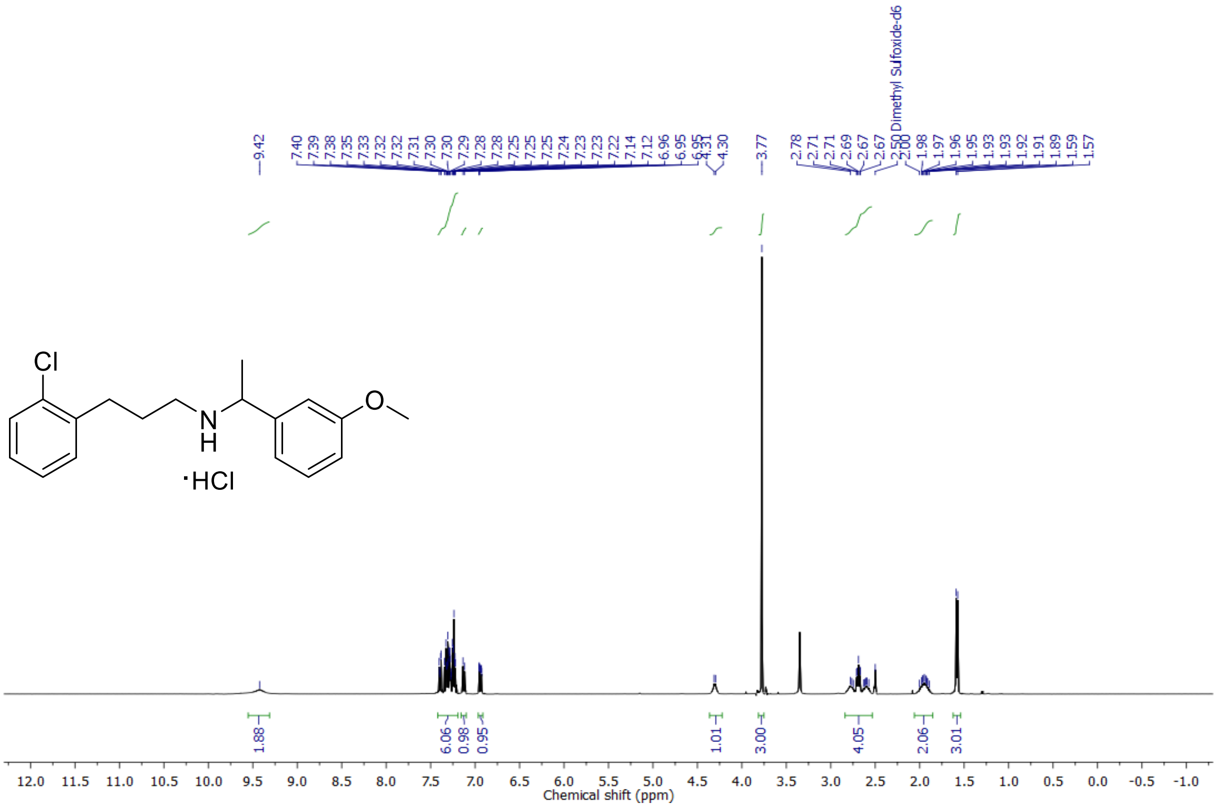

Figure S144: ^{1}H-NMR spectrum of **68**.

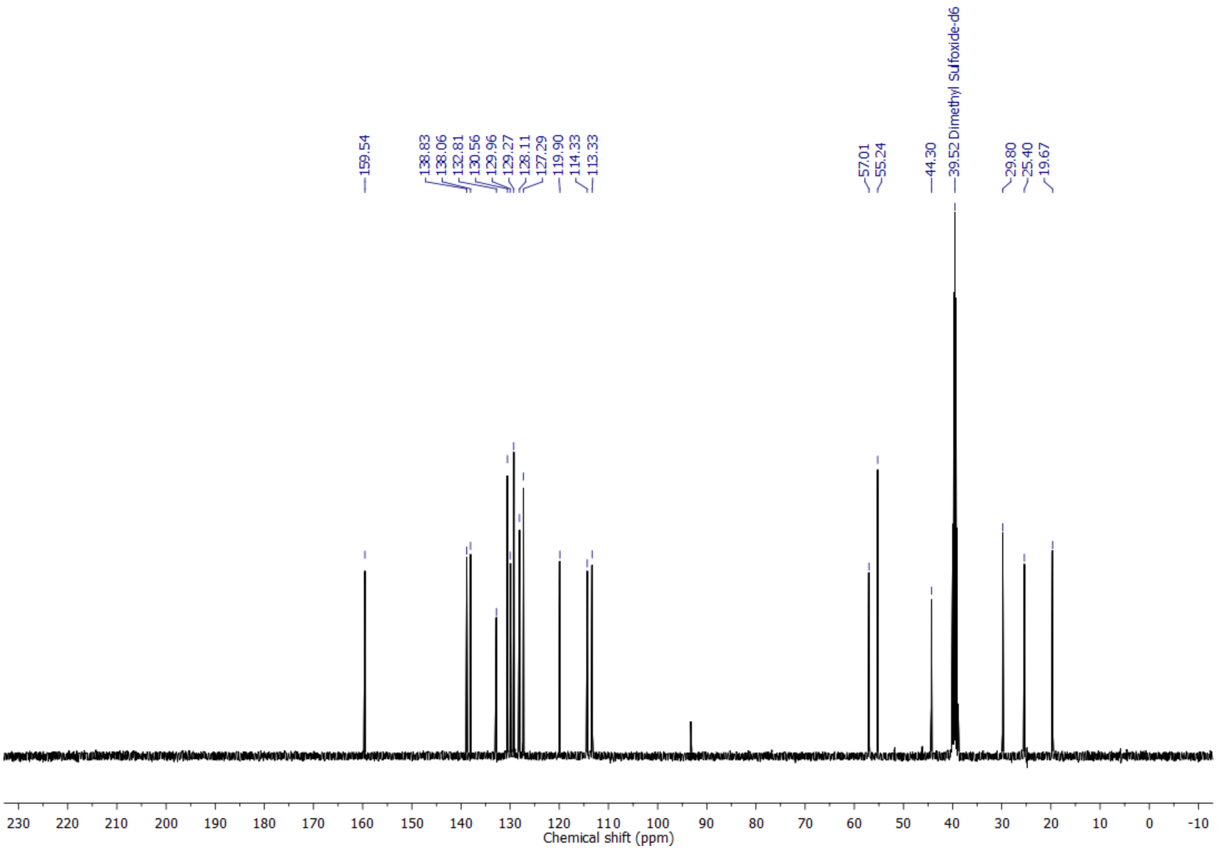

Figure S145: ^{13}C-NMR spectrum of **68**.

(8*R*,9*S*,13*S*,14*S*)-17-(benzylamino)-13-methyl-7,8,9,11,12,13,14,15,16,17-decahydro-6*H*-cyclopenta[a]phenanthren-3-ol hydrochloride (69)

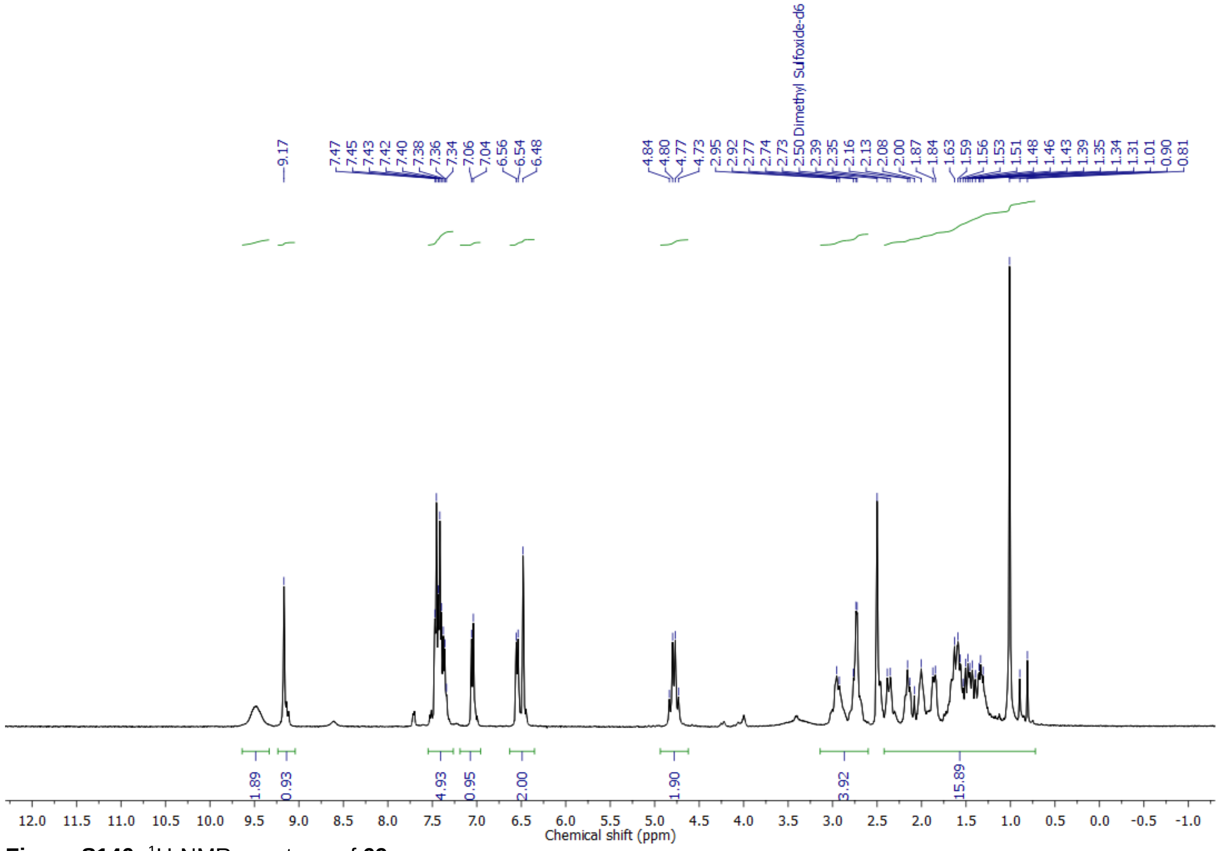

Figure S146: ^{1}H-NMR spectrum of **69**.

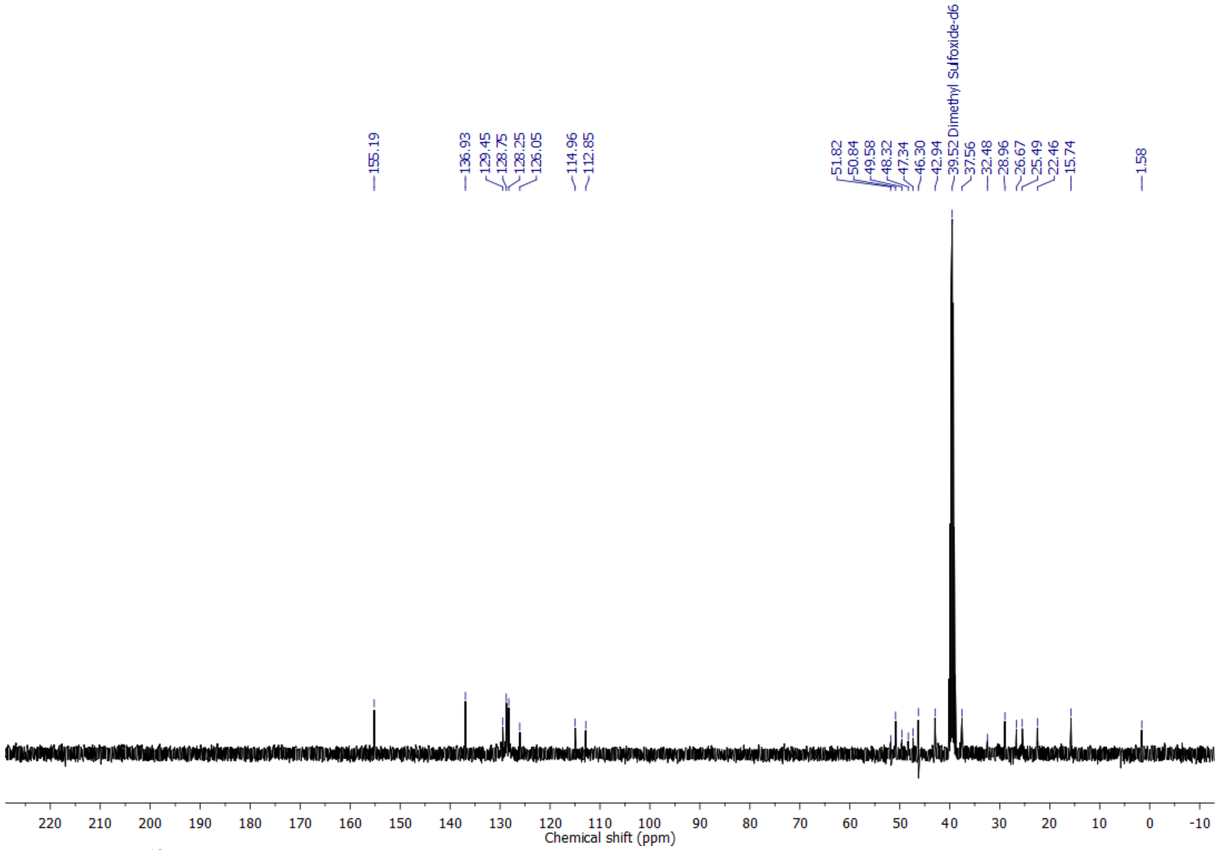

Figure S147: ^{13}C-NMR spectrum of **69**.

(8*R*,9*S*,13*S*,14*S*)-13-methyl-17-(pentylamino)-7,8,9,11,12,13,14,15,16,17-decahydro-6*H*-cyclopenta[a]phenanthren-3-ol hydrochloride (70)

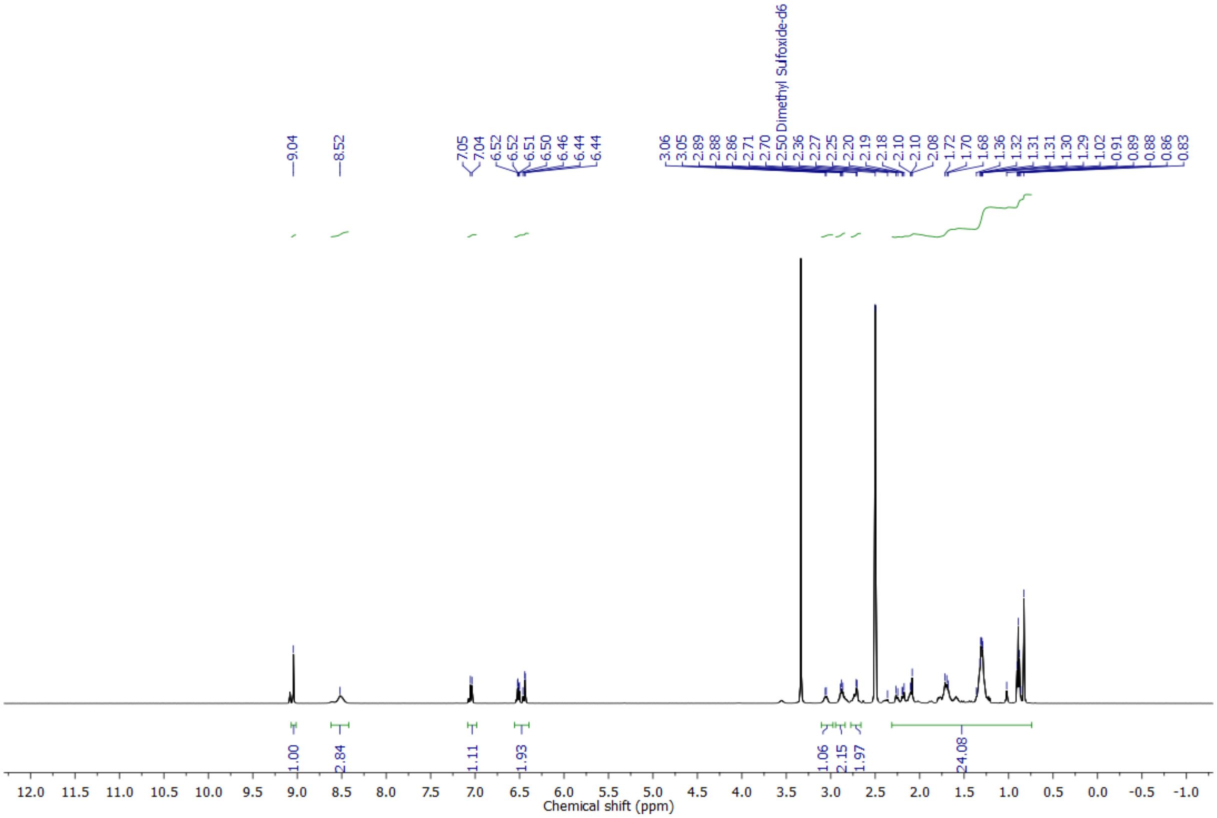

Figure S148: ^{1}H-NMR spectrum of **70**.

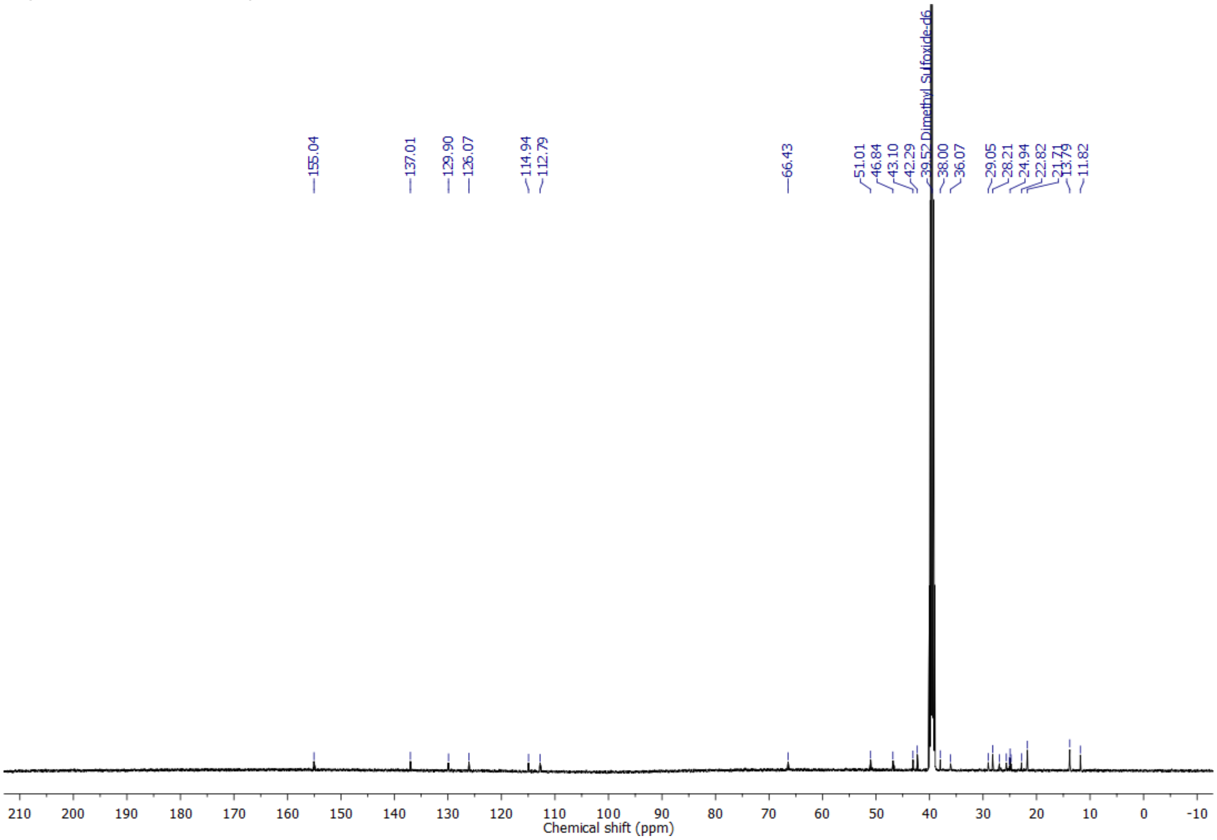

Figure S149: ^{13}C-NMR spectrum of **70**.

***N*-benzyl-1,1-diphenylmethanaminium chloride(8*R*,9*S*,10*S*,13*S*,14*S*,17*S*)-3-(benzylamino)-10,13-dimethylhexadecahydro-1H-cyclopenta[a]phenanthren-17-ol hydrochloride (71)**

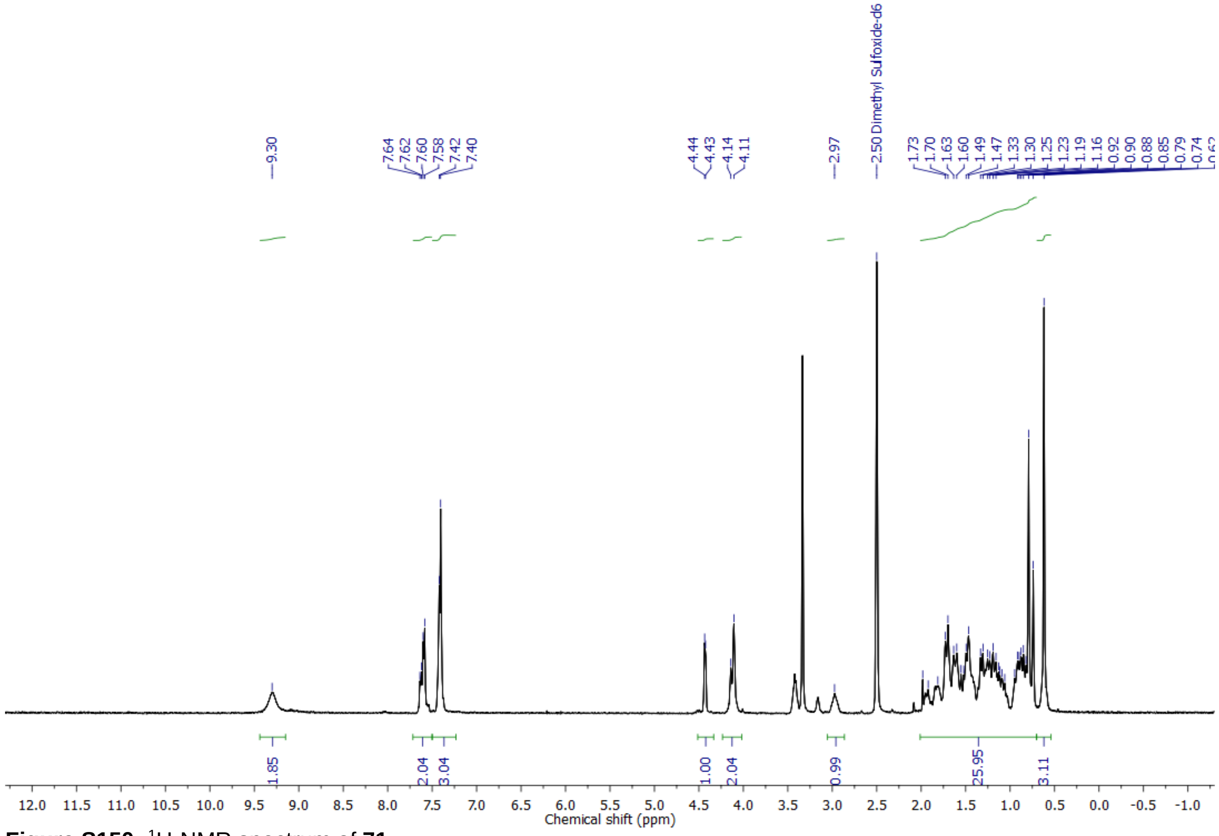

Figure S150: ^{1}H-NMR spectrum of **71**.

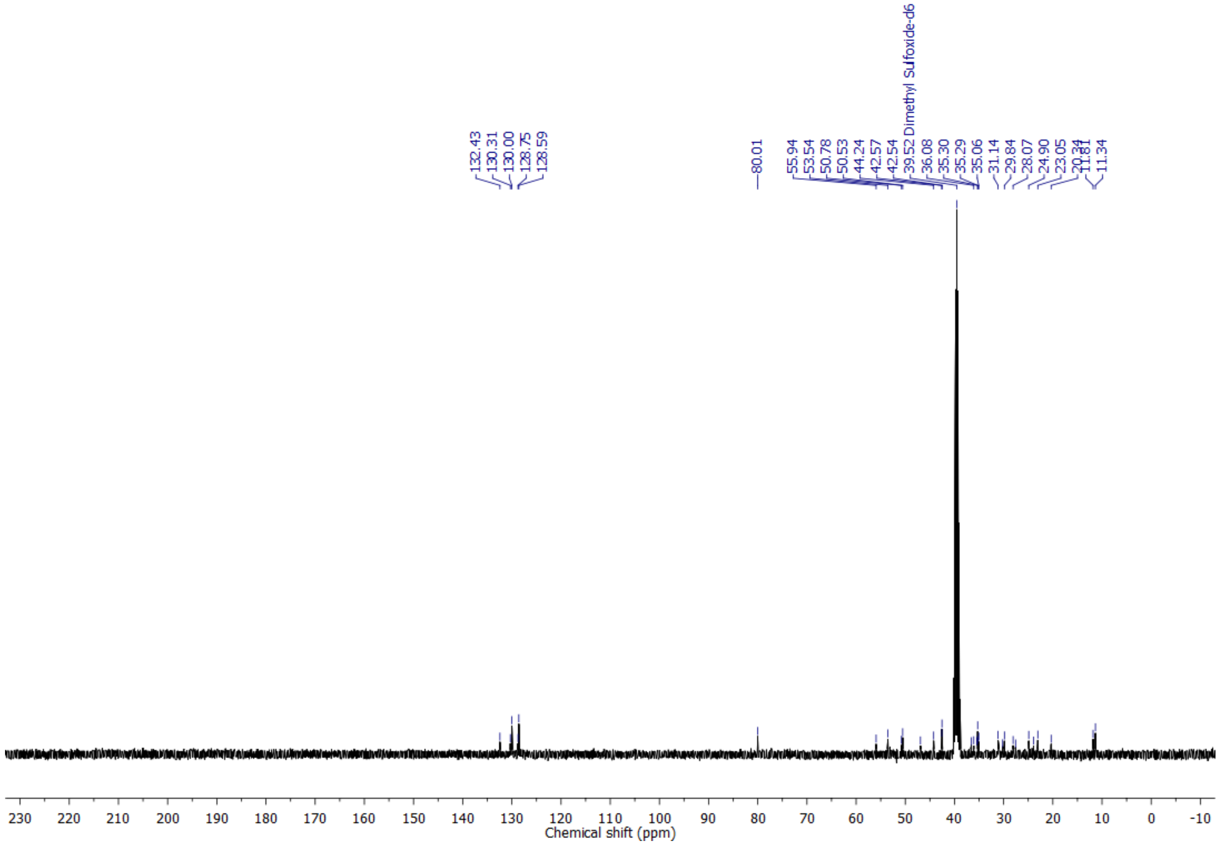

Figure S151: ^{13}C-NMR spectrum of **71**.

(8R,9S,10S,13S,14S,17S)-10,13-dimethyl-3-(pentylamino)hexadecahydro-1H-cyclopenta[a]phenanthren-17-ol hydrochloride (72)

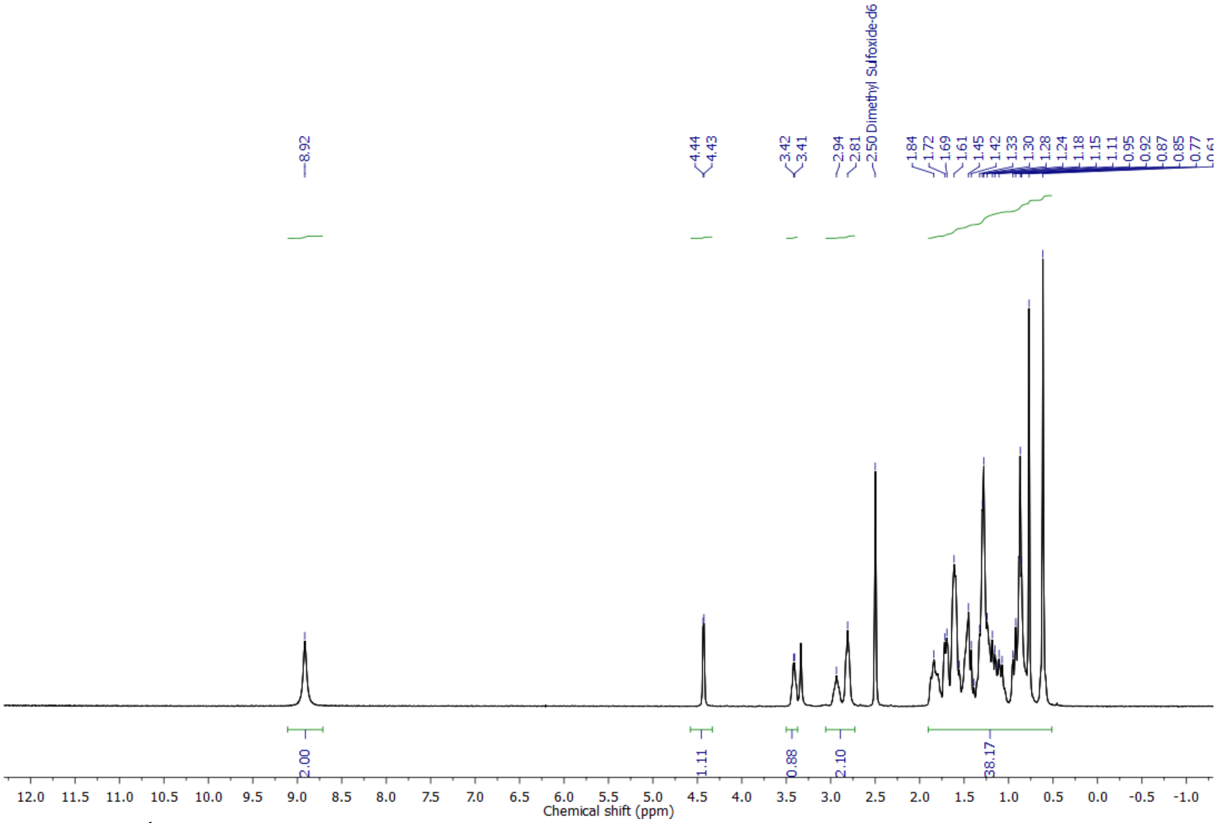

Figure S152: ^{1}H-NMR spectrum of **72**.

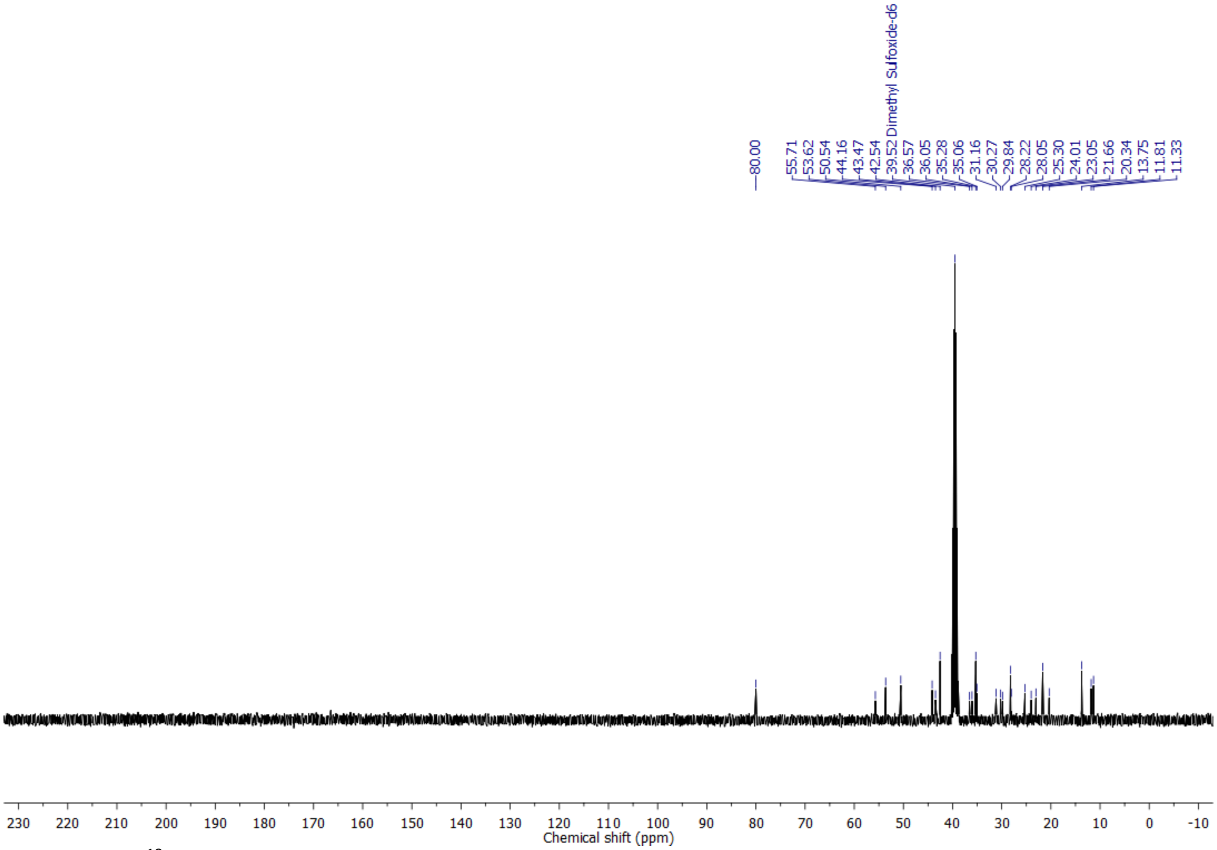

Figure S153: ^{13}C-NMR spectrum of **72**.

(8*R*,9*S*,10*R*,13*S*,14*S*,17*S*)-3-(benzylamino)-10,13-dimethyl-2,3,6,7,8,9,10,11,12,13,14,15,16,17-tetradecahydro-1H-cyclopenta[a]phenanthren-17-ol hydrochloride (73)

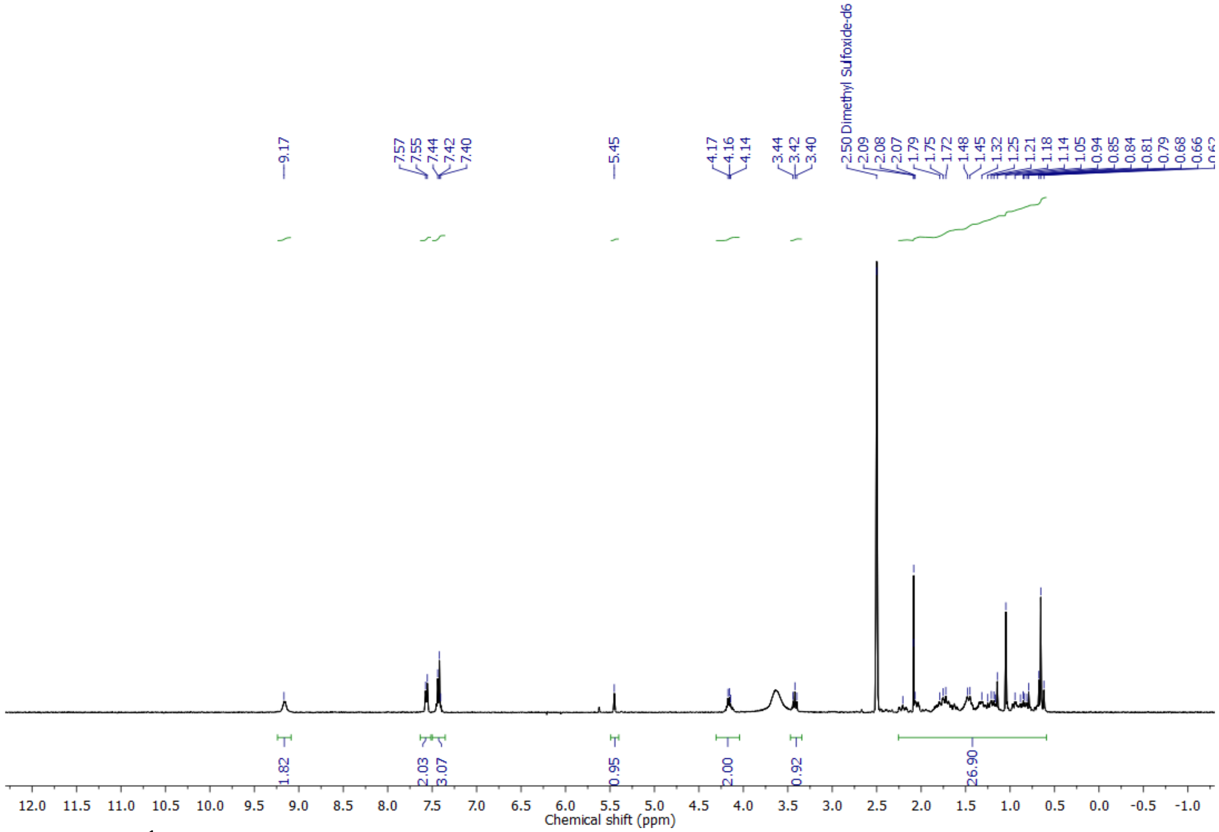

Figure S154: ^{1}H-NMR spectrum of **73**.

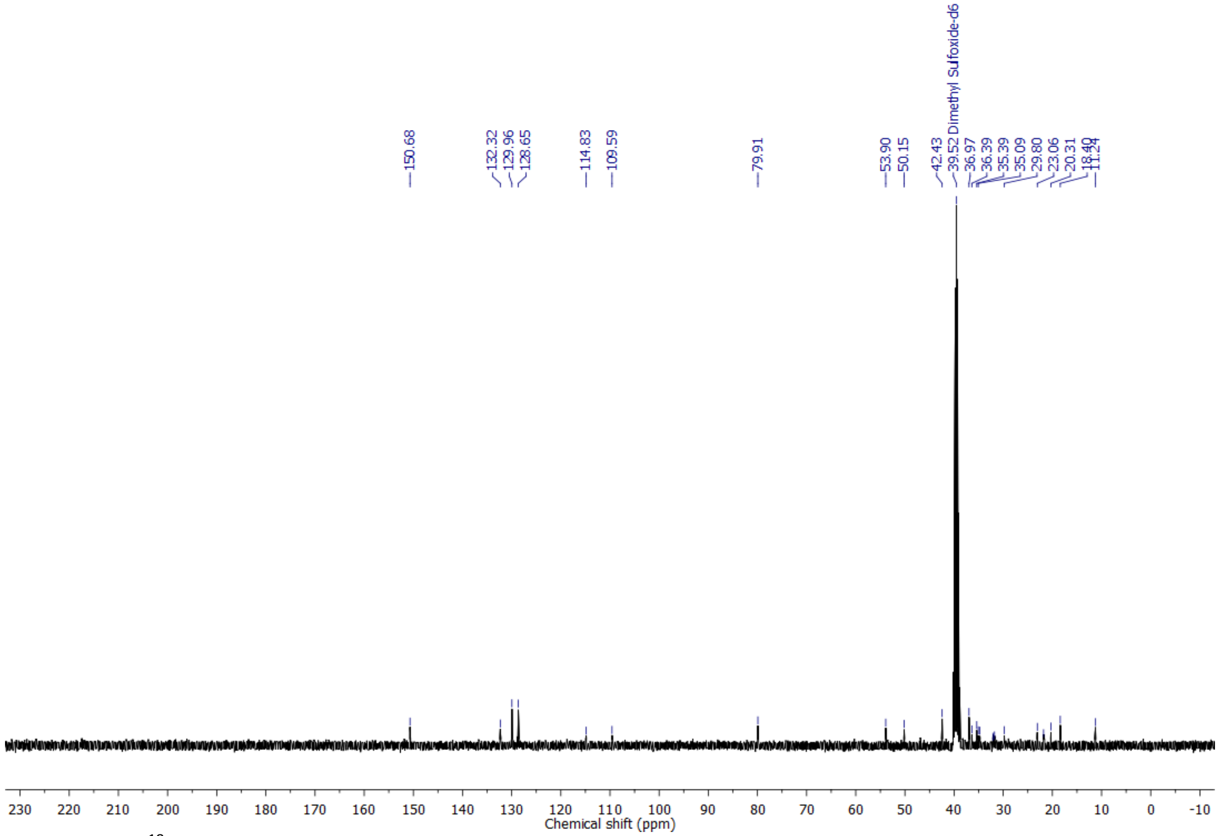

Figure S155: ^{13}C-NMR spectrum of **73**.

(8*R*,9*S*,10*R*,13*S*,14*S*,17*S*)-10,13-dimethyl-3-(pentylamino)-2,3,6,7,8,9,10,11,12,13,14,15,16,17-tetradecahydro-1*H*-cyclopenta[a]phenanthren-17-ol hydrochloride (74)

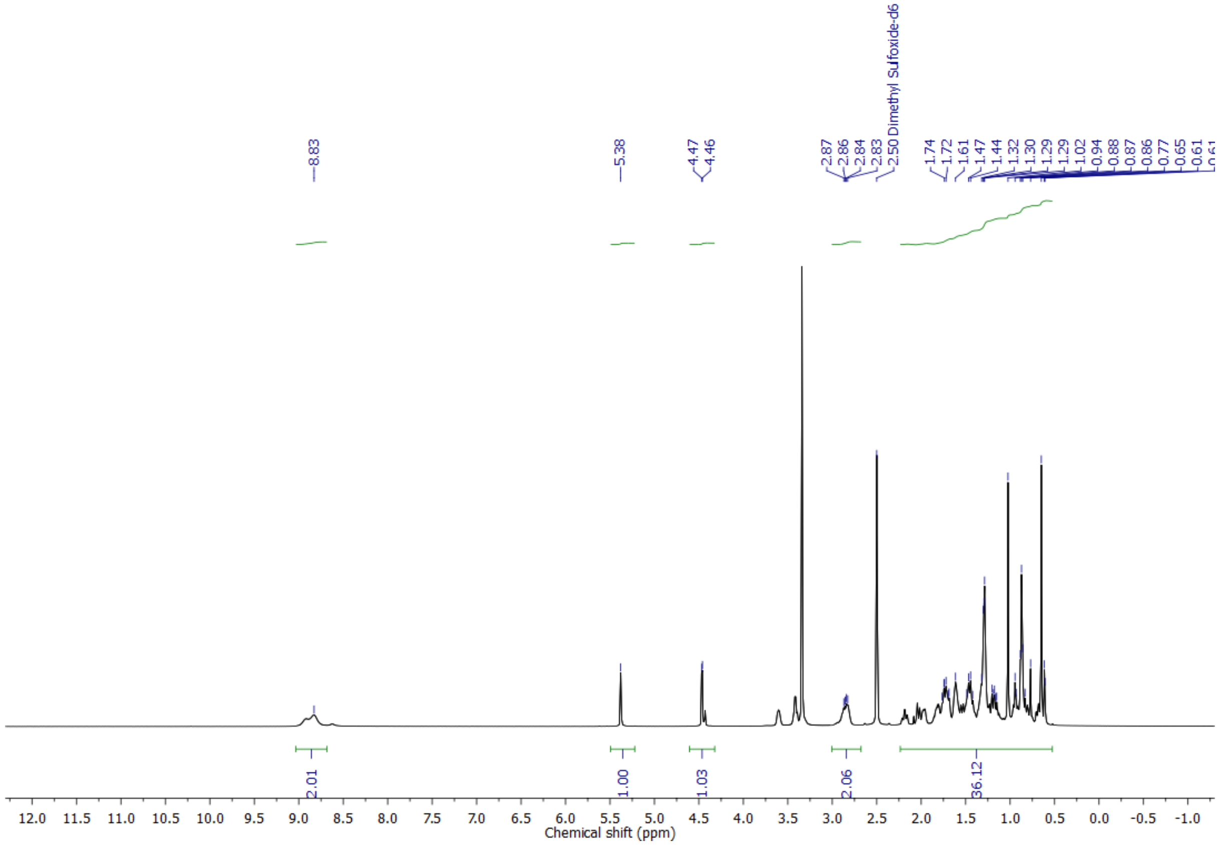

Figure S156: ^{1}H-NMR spectrum of **74**.

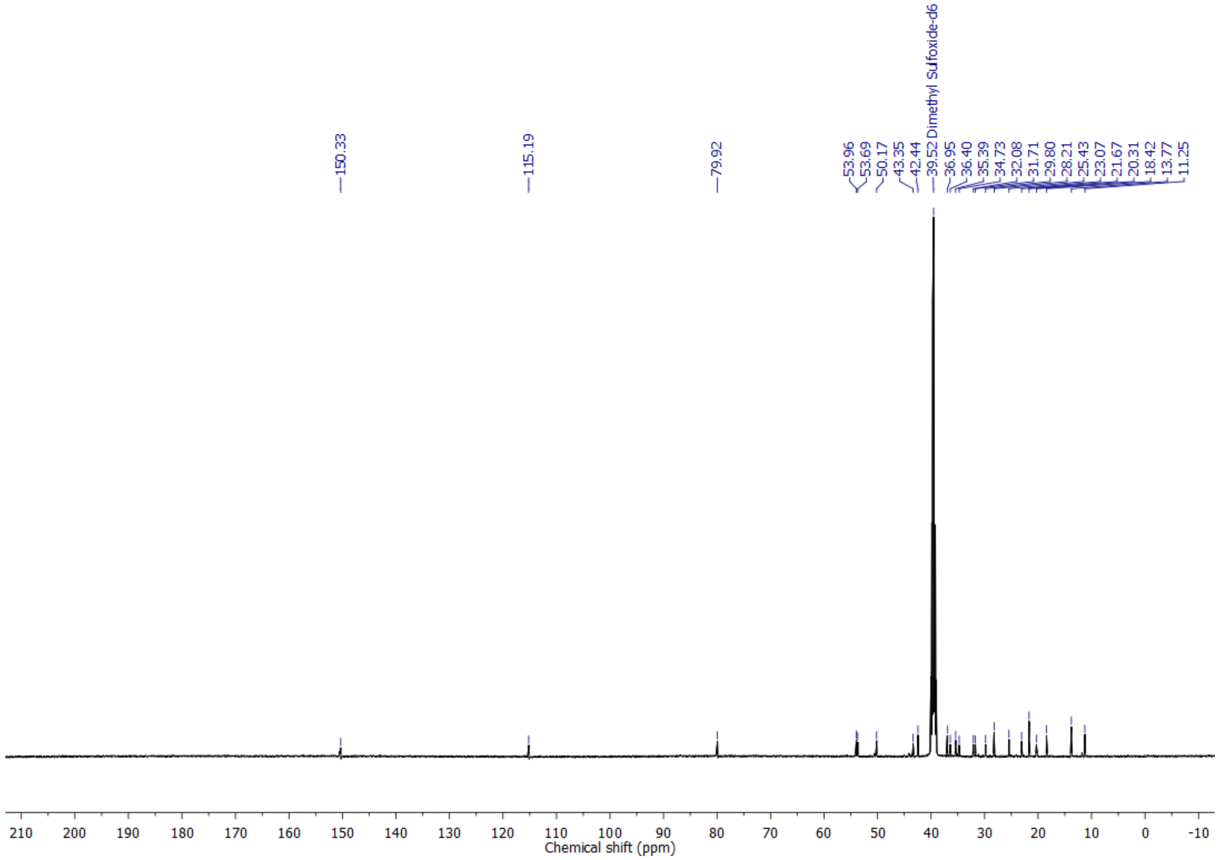

Figure S157: ^{13}C-NMR spectrum of **74**.

7. References

[1] S. L. J.Thomae, N. Prinz, T. Hartmann, M. Teck, S. Correll, M. Zobel, *Rev. Sci. Instrum.* **2019**, *90*, 043905.
[2] P. Juhás, T. Davis, C. L. Farrow, S. J. L. Billinge, *J. Appl. Crystallogr.* **2013***, 46*, 560-566.
[3] C. L. Farrow, P. Juhas, J. W. Liu, D. Bryndin, E. S. Bozin, J. Bloch, T. Proffen, S. J. L. Billinge, *Condens. Matter* **2007**, *19*, 335219.
[4] R. W. G. Wyckoff in *Crystal structures, Vol. 1*, Interscience Publishers, New York, **1963**.
[5] J. D. Hanawalt, H. W. Rinn, L. K. Frevel, *Ind. Eng. Chem. Anal. Ed.* **1938**, *10*, 457-512.
[6] L. F. B. Ribeiro, O. Flores, P. Furtat, C. Gervais, R. Kempe, R. A. F. Machadoa, G. Motz, *J. Mater. Chem. A.* **2017**, *5*, 720–729.
[7] M. C. dos Santos, F. Alvarez, *Phys. Rev. B* **1998**, *58*, 13918-13924.
[8] Z. H. Sheng, L. Shao, J. J. Chen, W. J. Bao, F. B. Wang, X. H. Xia, *ACS Nano* **2011**, *5*, 4350- 4358.
[9] P. Melinon, B. Maselli, F. Tournus, A. Perez, *Nat. Mater.* **2007**, *6*, 479-490.
[10] A. Poulain, C. Dupont, P. Martinez, C. Guizani, J. Drnec, *J. Appl. Cryst.* **2019**, *52*, 60-71.
[11] E. H. Kisi, C. J. Howard in *Applications of Neutron Powder Diffraction*, Oxford University Press, Oxford, **2012**, p.162.
[12] J. R. Cabrero-Antonino, R. Adam, V. Papa, M. Beller, *Nat. Commun.* **2020**, *11*, 3893-3911.
[13] M.-L.Yuan, J.-H. Xie, Q.-L. & Zhou, *ChemCatChem* **2016**, *8*, 3036–3040.
[14] L. Shi, X. Tan, H. Lv, J. Long, X. Zhang, *Chem. Eur. J.* **2017**, *23*, 546–548.
[15] J. M. John, S. H. Bergens, *Angew. Chem. Int. Ed.* **2011**, *50*, 10377–10380; *Angew. Chem.* **2011**, *123*, 10561-10564.
[16] J. Coetzee, D. L. Dodds, J. Klankermayer, S. Brosinski, W. Leitner, A. M. Z. Slawin, D. J. Cole-Hamilton, *Chem. Eur. J.* **2013**, *19*, 11039–11050.
[17] J. R. Cabrero-Antonino, E. Alberico, H.-J. Drexler, W. Baumann, K. Junge, H. Junge, M. Beller, *ACS Catal.* **2016**, *6*, 47–54.
[18] S. Kar, M. Rauch, A. Kumar, G. Leitus, Y. Ben-David, D. Milstein, *ACS Catal.* **2020**, *10*, 5511–5515.
[19] F. Schneck, M. Assmann, M. Balmer, K. Harms, R. Langer, *Organometallics* **2016**, *35*, 1931–1943.
[20] N. M. Rezayee, D. C. Samblanet, M. S. Sanford, *ACS Catal.* **2016**, *6*, 6377–6383.
[21] V. Papa, J. R. Cabrero-Antonino, E. Alberico, A. Spanneberg, K. Junge, H. Jungea, M. Beller, *Chem. Sci.* **2017**, *8*, 3576–3585.
[22] Y.-Q. Zou, S. Chakraborty, A. Nerush, D. Oren, Y. Diskin-Posner, Y. Ben-David, D. Milstein, *ACS Catal.* **2018**, *8*, 8014–8019.
[23] M. Stein, B. Breit, *Angew. Chem. Int. Ed.* **2013**, *52*, 2231–2234; *Angew. Chem.* **2013**, *128*, 2287-2290.
[24] T. Mitsudome, K. Miyagawa, Z. Maeno, T. Mizugaki, K. Jitsukawa, J. Yamasaki, Y. Kitagawa, K. Kaneda, *Angew. Chem. Int. Ed.* **2017**, *129*, 9509–9513; *Angew. Chem.* **2017**, *129*, 9509-9513.
[25] Y. Xie, P. Hu, T. Bendikov, D. Milstein, *Catal. Sci. Technol.* **2018**, *8*, 2784–2788.

Author Contributions

T. S. carried out the catalyst synthesis, catalyst characterization and catalytic reactions. S. L. J. T. and M. Z. performed XRD analyses and PDF refinement. L. K. developed the support material. T. S. and R. K. designed the experiments and co-wrote the manuscript.
